# Population structure of mitochondrial genomes in *Saccharomyces cerevisiae*

**DOI:** 10.1186/s12864-015-1664-4

**Published:** 2015-06-11

**Authors:** John F. Wolters, Kenneth Chiu, Heather L. Fiumera

**Affiliations:** Department of Biological Sciences, Binghamton University, Binghamton, NY USA; Computer Science Department, Binghamton University, Binghamton, NY USA

**Keywords:** *Saccharomyces cerevisiae* mitochondrial genome, mtDNA, Fungal genetics, Mitochondrial genetics, Intron, Mobile elements, Single-molecule sequencing

## Abstract

**Background:**

Rigorous study of mitochondrial functions and cell biology in the budding yeast, *Saccharomyces cerevisiae* has advanced our understanding of mitochondrial genetics. This yeast is now a powerful model for population genetics, owing to large genetic diversity and highly structured populations among wild isolates. Comparative mitochondrial genomic analyses between yeast species have revealed broad evolutionary changes in genome organization and architecture. A fine-scale view of recent evolutionary changes within *S. cerevisiae* has not been possible due to low numbers of complete mitochondrial sequences.

**Results:**

To address challenges of sequencing AT-rich and repetitive mitochondrial DNAs (mtDNAs), we sequenced two divergent *S. cerevisiae* mtDNAs using a single-molecule sequencing platform (PacBio RS). Using *de novo* assemblies, we generated highly accurate complete mtDNA sequences. These mtDNA sequences were compared with 98 additional mtDNA sequences gathered from various published collections. Phylogenies based on mitochondrial coding sequences and intron profiles revealed that intraspecific diversity in mitochondrial genomes generally recapitulated the population structure of nuclear genomes. Analysis of intergenic sequence indicated a recent expansion of mobile elements in certain populations. Additionally, our analyses revealed that certain populations lacked introns previously believed conserved throughout the species, as well as the presence of introns never before reported in *S. cerevisiae*.

**Conclusions:**

Our results revealed that the extensive variation in *S. cerevisiae* mtDNAs is often population specific, thus offering a window into the recent evolutionary processes shaping these genomes. In addition, we offer an effective strategy for sequencing these challenging AT-rich mitochondrial genomes for small scale projects.

**Electronic supplementary material:**

The online version of this article (doi:10.1186/s12864-015-1664-4) contains supplementary material, which is available to authorized users.

## Background

*Saccharomyces cerevisiae* has long been at the center of mitochondrial genetics, owing to a facultative anaerobic lifestyle and powerful genetic tools. Most mitochondrial research has focused on a limited number of laboratory strains, allowing for exacting functional studies of mitochondrial processes. Recently, this budding yeast has blossomed into a model for evolutionary biology [1–3]. Genome resequencing projects have revealed genetic diversity and natural population structures of *S. cerevisiae* [4–8]. The diversity in mitochondrial genomes has not been so thoroughly assessed.

Evolution of *S. cerevisiae* mitochondrial DNAs (mtDNAs) differs from nuclear genome evolution in multiple ways. Despite strong purifying selection on mtDNAs, intraspecific mitochondrial variation in *S. cerevisiae* is extensive, owing mainly to differences in intergenic sequences and mobile elements (reviewed in [9] and described below). Replication of mtDNA is not tied to the cell cycle [10], contributing to higher mutation rates in mtDNAs than in nuclear genomes [11]. In yeast, inheritance of mtDNAs is usually biparental [12], although the distribution of parental mitochondrial alleles in progeny is difficult to predict. This is due, in part, to different admixtures of parental mtDNAs in zygotes, mitochondrial recombination, and subsequent loss of heteroplasmy [13]. Additionally, mobile elements in mtDNA may move laterally within populations [14]. Together, these factors may cause mitochondrial sequences to diverge from nuclear population structure.

The mtDNAs of *S. cerevisiae* contain three subunits of the ATP synthase complex (*atp6, atp8* and *atp9*), apocytochrome *b* (*cob*), three subunits of the cytochrome *c* oxidase (*cox1*, *cox2*, and *cox3*), and one ribosomal protein (*rps3/VAR1*). The mitochondrial genome also encodes large and small rRNAs (*rnl* and *rns*), an RNA component of the mitochondrial RNAse P (*rnpB*) and 24 tRNAs [15]. In *S. cerevisiae*, these genes are separated by long AT-rich intergenic sequences and numerous introns. Repetitive GC-rich regions, known as GC clusters, intersperse the otherwise AT-rich mtDNAs [16, 17]. These clusters typically fall within non-coding sequences, but are also inserted into *rps3*/*VAR1* and ribosomal RNA genes, where they alter the size of the resulting gene products [18–20]. Their palindromic nature likely influences mtDNA structure, which may explain associations with mtDNA instability [21] and mitochondrial recombination [22]. It has been proposed, but never formally tested, that GC cluster-induced structural changes may affect gene regulation [23].

Optional group I and group II introns (differentiated by characteristic RNA secondary structures) also contribute to intraspecific mtDNA variation. Self-encoded homing endonucleases and reverse transcriptases facilitate intron mobility [24] and acquired maturase activities aid in their preservation [25]. In *S. cerevisiae,* mitochondrial introns are found within *cox1* (group 1: aI3α, aI3γ, aI4α, aI4β, aI5α, aI5β, aI5γ; group II: aI1, aI2, and aI5γ), *cob* (group I: bI2, bI3, bI4, bI5; group II: bI1β) and *rnl* (group I: Ω) [15]. Additional introns observed in other *Saccharomyces* species include the group I introns aI3β in *cox1* and bI1α in *cob* [26]. Incompatibilities between nuclear-encoded splicing factors and non-native introns provide credible support to theories that mitochondrial-nuclear coevolution have contributed to speciation of *Saccharomyces* yeasts through Dobzhansky-Muller-type incompatibilities [26–30]. However, some incompatibilities are strain-specific [26, 27] and highlight the importance of investigating mitochondrial diversity within, in addition to between, species.

The low number of available mtDNA sequences for *S. cerevisiae* yeasts has limited population genetic analyses. The mitochondrial genome of the reference strain was fully sequenced in 1998 [15], and until recently, very few additional mtDNAs were solved [31–33]. The lack of mitochondrial genomes produced by most high-throughput sequencing projects is most likely based on biases against the AT-rich and repetitive DNA during library preparation, sequencing and alignment [34, 35] and discussed in [27], but complete mtDNA sequence reconstruction is possible [32]. A particularly robust resequencing project recently released mtDNA sequences for 93 strains [6], thus providing substantial new resources for mtDNA population genetics. Despite these methodological advances in large-scale projects, sequencing these AT-rich and complex mtDNAs remains challenging, especially for smaller scale studies.

In this study, we sequenced two mitochondrial genomes using PacBio-RS. This single-molecule sequencing platform was successfully used for both chloroplast and microbial genomes [36, 37], suggesting it may be useful for solving *Saccharomyces* genomes for a small number of strains. We then compared these two newly generated sequences with 98 additional mtDNA sequences to provide a comprehensive picture of intraspecific mtDNA sequence variation in *S. cerevisiae*. Our analyses revealed population-specific genic and intergenic sequence structure including novel intron variation.

## Results

### *De Novo* assembly of *S. cerevisiae* mtDNAs

To assess the feasibility of resolving AT-rich yeast mitochondrial genomes utilizing single-molecule real time sequencing (SMRT), we first generated a complete mitochondrial genome for *S. cerevisiae* strain NCYC3594 [38], a haploid derivative of YJM975 [39]. The nuclear genome of this wine/European isolate has been sequenced numerous times using Illumina sequencing [4, 6, 8, 40]. Only recently was a complete mitochondrial sequence for YJM975 solved [6]. We sequenced an 800 bp library created from a sample enriched for mtDNA. The long read lengths (average = 606 bp) facilitated a *de novo* assembly that produced a single contig with length and GC content consistent with the S288C mitochondrial genome. In addition to the mitochondrial contig, the sequencing reaction produced numerous reads that assembled into shorter (<5 kb) contigs with GC contents more representative of the nuclear genome (>30 %). Following assembly quality improvements, the resulting mtDNA sequence for NCYC3594 was 78,917 bp with a GC content of 16.1 %.

We also sequenced an additional mtDNA from strain NCYC3585 [38], a haploid derivative of 273614N [4]. To reduce nuclear DNA contamination in the sequencing sample, intact mitochondria were treated with DNase prior to isolation of mtDNA. This increased the mtDNA:nDNA ratio in the DNA samples from 0.3:1 (for NCYC3594) to 776:1 (for NCYC3585). We used a longer insert size (6 kbp) in the sequencing reaction to obtain average read lengths of 2055 bp. Following *de novo* assembly and quality refinements, the mtDNA sequence for NCYC3585 was 76,596 bp with a GC content of 15.1 %.

To assess the quality of these assemblies, we aligned these sequences with mitochondrial sequences from the respective parental strains [6]. The differences between the mtDNA sequences for NCYC3594 and YJM975 were limited to 7 small indel polymorphisms, ranging from 1 to 25 bp (summing to 41 bp in total, <0.0006 % disagreement). The differences between NCYC3585 and YJM1450 (alias 273614N) were 3 indels, including 2 singletons and a 7 bp indel (<0.0002 % disagreement, Additional file 1: Table S1). All indels occurred within AT-rich intergenic regions. It is not known whether these small differences were due to strain specific polymorphisms or sequencing/assembly errors, but overall, the mtDNAs were nearly identical. Thus, single-molecule sequencing approaches generated highly accurate sequences of *Saccharomyces* AT-rich mtDNA.

### Intraspecific diversity of mitochondrial protein coding sequences

To explore intraspecific mitochondrial evolution in *S. cerevisiae*, we first investigated phylogenetic relationships among protein coding sequences from 99 unique strains. We compared coding sequences from complete mtDNA sequences including the newly obtained sequences presented here, with those from the reference strain S288C [15], industrial isolate NCIM3107 [32], sake strain Kyokai No. 7 [33], clinical isolate YJM789 [31], and 93 additional strains from a recently released dataset [6]. In sum, these strains include those from distinct ancestral populations, as previously described [4] including wine/European (n = 30), North American (n = 2), West African (n = 4), Malaysian (n = 1), and Sake (n = 5) lineages, as well as a large number of strains with admixed genetic backgrounds (n = 57). A complete strain list and accession numbers provided in Additional file 2: Table S2.

**Fig. 1:**
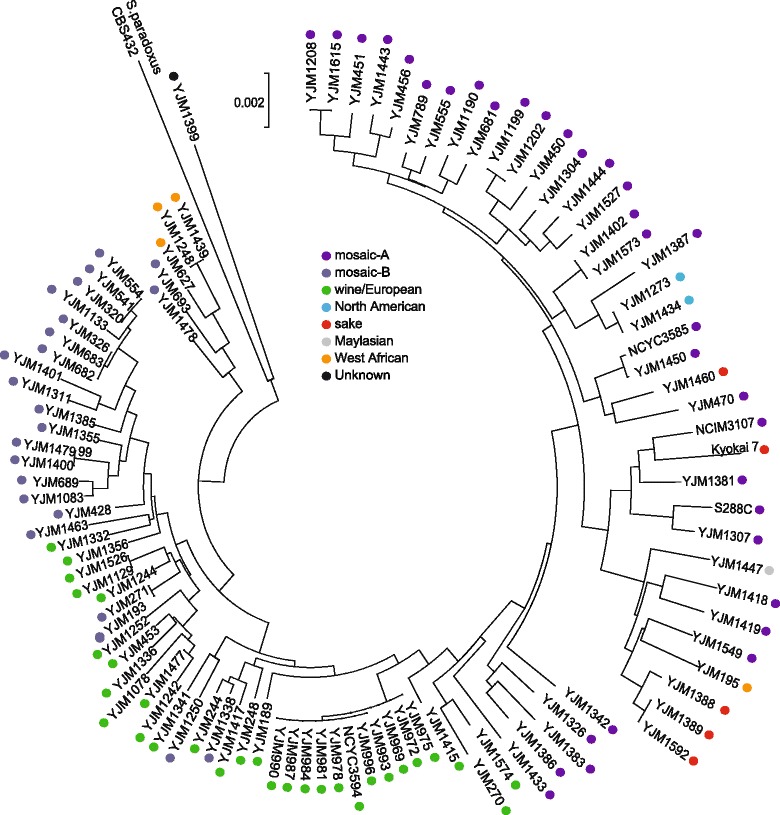
Phylogenetic relationship of mitochondrial coding sequences. A rooted phylogenetic tree of concatenated mitochondrial protein coding sequences from 99 *S. cerevisiae* isolates, including *S. paradoxus* strain CBS432 as an outgroup. Population designations are indicated.

A phylogenetic tree was built based on alignment of the concatenated coding sequences of *cox1-atp8-atp6-cob-atp9-rps3-cox2-cox3*, using sequences from *S. paradoxus* [27] as an outgroup (Fig. 1). Based on a total of 457 polymorphic positions across 6762 total aligned base pairs, mitochondrial sequences grouped into three broad clades. Mitochondrial genes from Asian strains (sake and Malaysian) and North American strains formed one large clade. Within this clade, sequences from the North American strains formed a distinct lineage from the Asian strains. A second distinct clade consisted mainly of wine/European strains. Sequences from three West African strains formed a third distinct clade. A single West African isolate grouped nearest to the sake lineage.

The strains with admixed (mosaic) ancestries had mtDNA sequences that mainly grouped within the larger Asian/North American clade, or within the European strains. We subdivided these mosaic strains into mosaic-A (Asian/North American), and mosaic-B (European) populations. Outliers in these groupings included two mosaic strains more closely related to the West African strains, and a single mosaic strain, YJM1399. Mitochondrial sequences from YJM1399 clustered more closely to *S. paradoxus* than to other *S. cerevisiae* strains. The nuclear background of this mosaic strain was also significantly diverged (particularly in the number of insertions and deletions) [6], and thus, was treated independently for subsequent analyses. Overall, the phylogenetic analysis of mitochondrial sequences largely recapitulated the population structures obtained by previous analysis of their nuclear genomes; wine/European, West African, sake/Asian, and North American strains were phylogenetically distinct from each other and from most strains with mosaic ancestry.

We assessed polymorphisms within the species in each gene separately (Additional file 3: Table S3). The genes *atp8* and *atp9* each contained no nonsynonymous mutations and only one synonymous site, and the lowest nucleotide diversities of all coding sequences (π = 0.0033 and 0.0017, respectively). Nonsynonymous variation was observed in all other genes, with the highest nonsynonymous/synonymous polymorphism ratio in *atp6* (pN/pS = 0.172). Consistent with the known intraspecific size variations in *rps3/VAR1* [19], most of the coding sequence polymorphism occurred in this gene (161 polymorphic sites). We obtained similar phylogenetic groupings using alignments of concatenated coding sequences omitting *rps3* or of individual genes (not shown). Despite the different degrees of variation between the coding sequences, the phylogeny was not overly sensitive to variation within any one gene.

### Divergent strains contain extensive indel variation across their mtDNAs

To assess whether patterns of mitochondrial variation are consistent between divergent populations, nine mtDNAs were chosen to reflect mitochondrial diversity across the species. These included the mtDNAs from NCYC3594 and YJM1078 (European), YJM1273 (North American), YJM1388 (sake), YJM1439 (West African), NCYC3585 and YJM789 (mosaic-A), YJM1401 (mosaic-B), and the reference strain, S288C. We performed a multiple genome alignment followed by extensive manual curation to properly align intron/exon boundaries and correct misalignments of large, repetitive intergenic sequences. A fully annotated and interactive alignment file is provided in Additional file 4: File S1.

**Fig. 2:**
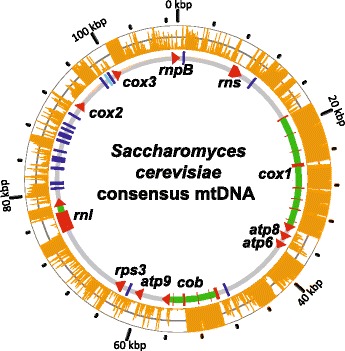
Consensus genome map of *S. cerevisiae* mtDNA. A consensus genome map based on the alignment of nine divergent mtDNAs illustrates the extensive polymorphisms across *S. cerevisiae* mtDNAs. The consensus sequence (~109 kbp) is substantially longer than the longest mtDNA in this alignment (~86 kbp) due to indel variation. Genes (red arrows), introns (green), and tRNAs (blue) are indicated. The light blue bar indicates a sole tRNA encoded on the light strand. The orange bars indicate the number of polymorphic sites within 100 bp windows, where the inner and outer edges of the circle represent 0 and 100, respectively. The grey line represents the genome-wide average of 51 polymorphic sites per window.

Consistent with known size variation, extensive indel polymorphism was observed across the genomes, particularly in intergenic and intronic regions (Fig. 2). These mtDNAs ranged from 76,596 bp (NCYC3585) to 86,214 bp (YJM789), with size differences due to intron content, many small indels (<100 bp), and a small number of large indels that generally corresponded to known variable hypothetical ORFs. Coding sequences, representing less than 8.6 % of mitochondrial genome, were conserved and syntenous. Nucleotide diversity of intergenic regions (π =0.1782) was significantly higher than for exons (π =0.0138), mainly due to indel variation. Excluding indels, nucleotide diversity of intergenic regions was significantly reduced (π = 0.0147) but was still twice that of exons (π = 0.007). Within the coding sequences, indels occurred in the hypervariable *rps3/VAR1* gene [19], and one instance of an in-frame 3 bp insertion in *cob*.

### Mobile GC clusters exhibit population specific patterns of variation

Mobile GC-clusters are a known source of indel varation in *Saccharomyces* mtDNAs [21]. These clusters range between 30–80 bp and have been characterized into distinct classes based on consensus sequences [16, 17]. Most GC clusters fall into the M1 and M2 classes (following the classifications defined in [17]) in both *S. cerevisiae* and *S. paradoxus* [21, 27, 41]. Subclasses of M1 and M2 clusters (M1’, M2’, and M2”) are similar to their parent classes but contain specific insertions or deletions. M3 and M4 classes are found within tandem arrays of GC cluster repeats. The G and V classes are optionally found in *ori* sequences and the *rps3* gene, respectively.

We first determined the number and classes of GC clusters in all 99 complete mitochondrial genomes (Additional file 5: Table S4). On average, each strain had 120 ± 22 classifiable GC-rich motifs. The majority of these GC clusters appeared as single elements, with an average of 21 ± 7 tandem arrays of 2 or more clusters per strain. Consistent with previous descriptions [15–17], the numbers of M1 (43 ± 10) and M2 (26 ± 6) clusters were highest. On average, M3 and M2’ clusters were equally represented (15 ± 4) and observed more frequently than the remaining classes (mean < 7). We also scanned each genome for GC-rich regions that did not fit a consensus sequence, identifying an average of 42 ± 5 additional positions per strain. These unclassified GC-rich regions were often associated with tandem arrays of GC clusters and are likely degenerate variants of the main classes.

**Fig. 3:**
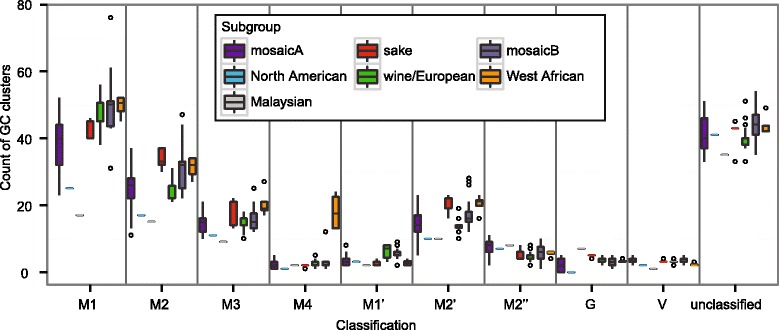
GC clusters by class and population. Box plots illustrate the distribution of each class of GC clusters within and between phylogenetic populations. Whiskers extend to the furthest value within 1.5 times the inter-quartile range from the 1st and 3rd quartiles. Outliers are plotted as empty circles. The number of strains analyzed for each population is provided in Table 1.

The variation in GC clusters demonstrated population specific patterns (Fig. 3 and Table 1). While the number of clusters varied between populations for each class (individual ANOVAs, *P* ≤ 1.0 x 10^−3^), the M4 clusters demonstrated the largest population specific effect (*P* ≤ 1.0 x 10^−26^). West African strains contained significantly more M4 clusters (18 ± 6) than any other population (2 ± 1).

**Table 1:** Mitochondrial GC clusters in *S. cerevisiae*

Population	n	M1	M2	M3	M4	M1’	M2’	M2”	G	V	total	U	T
All strains	98	43.3 ± 9.5	26.4 ± 6.4	15.2 ± 3.5	3.1 ± 3.5	4.7 ± 2.1	15.4 ± 4.0	6.1 ± 2.4	3.0 ± 1.6	3.2 ± 0.8	120.3 ± 22.1	41.7 ± 5.1	20.7 ± 6.6
mosaic-A	33	38.0 ± 7.8	25.2 ± 6.1	14.4 ± 3.0	2.2 ± 1.0	3.5 ± 1.6	14.6 ± 4.0	7.8 ± 2.0	2.4 ± 1.9	3.3 ± 0.7	111.4 ± 20.2	41.5 ± 5.4	19.2 ± 4.4
N. American	2	25.0	17.0	11.0	1.0	3.0	10.0	7.0	0.0	2.0	76.0	41.0	12.0
Malaysian	1	17.0	15.0	9.0	2.0	2.0	10.0	8.0	7.0	1.0	71.0	35.0	12.0
sake	5	43.2 ± 3.0	33.8 ± 3.1	18.2 ± 4.3	1.8 ± 0.5	2.6 ± 0.9	19.8 ± 2.8	5.2 ± 1.8	4.8 ± 0.5	3.2 ± 0.5	132.6 ± 8.1	41.4 ± 4.8	24.6 ± 3.0
wine/Euro	30	47.2 ± 4.5	23.7 ± 2.7	15.3 ± 2.0	2.7 ± 0.9	6.2 ± 1.9	13.9 ± 1.6	4.5 ± 1.3	3.5 ± 0.9	3.0 ± 0.6	119.9 ± 8.3	39.6 ± 3.3	19.0 ± 2.6
mosaic-B	23	48.7 ± 9.3	31.2 ± 6.2	15.8 ± 3.2	2.9 ± 2.1	5.3 ± 1.7	17.5 ± 4.4	6.2 ± 2.5	2.8 ± 1.2	3.5 ± 0.9	133.9 ± 20.8	44.2 ± 4.8	22.9 ± 6.5
West African	4	49.5 ± 3.3	31.3 ± 3.4	20.5 ± 4.4	18.0 ± 5.8	2.75 ± .96	20.3 ± 3.0	5.5 ± 1.0	3.3 ± 0.5	2.3 ± 0.5	153.3 ± 11.8	43.8 ± 3.5	40.0 ± 9.3
*S.paradoxus* YJM1399	1	14.0	1.0	6.0	0	1.0	0	1.0	0	4.0	27.0	37.0	4.0

The number of GC cluster for each classification is provided for all strains, and for each population. The number of strains within each population is given (n). Values are means ± 1 standard deviation. GC cluster counts were identical for North American strains. U indicates unclassified GC-rich regions. T indicates the number of tandem arrays.

To determine if GC clusters ever occurred in conserved positions, we examined the nine mtDNA multiple alignment. These mtDNAs contained a total of 1087 classifiable GC clusters that populated 282 unique positions. GC clusters were conserved at only 13 positions. These represented most classes (7 M1, 2 M2, and 1 each in M2”, M3, M4, and V classes) and were evenly distributed across the genome.

We more closely examined the apparent expansion of M4 clusters in the West African lineage by examining the West African representative in the multiple alignment. All 22 of the M4 clusters in YJM1439 (West Africa) were found in short tandem arrays containing the M4 cluster and an M1 cluster, including 2 of the 7 conserved M1 clusters. In nearly all identified cases (21/22), the M4 cluster was located upstream of its associated M1 cluster, suggesting that the M4 cluster may target the upstream TAG motif in conjunction with the 5′ region of the M1 cluster.

Additional repetitive characters across the genomes were identified in three mtDNAs using a k-mer counting method [42]. Highly repetitive short AT-rich sequences were observed only when using 15-mer scans (Additional file 6: File S2). These consisted of di- to penta-nucleotide repeats consistent with slippage during replication. Highly repetitive GC-rich sequences were only observed at k-mer scans under 50 bp.

### Novel mosaicism in intron patterns

Fifteen distinct introns have been described in *S. cerevisiae* mitochondrial genomes [15, 31]. We compared the mitochondrial intron profiles for 104 unique strains, including data from a deep sequencing project that did not provide complete mitochondrial genomes [40] but did provide read coverage for most coding sequences. The intron profiles generated were similar for strains appearing both in partial sequences from Bergstom et al. [40] and complete sequences from Strope et al. [6], thus providing a measure of confidence for deducing intron content from the partially sequenced mtDNAs.

Mitochondrial introns are remarkable variable, resulting in mosaic presence/absence patterns between individual strains. Seven of the nine introns in *cox1* are known as optional [15, 31]. In this analysis, the presence of these variable introns ranged from 25-91 % in the 104 mtDNAs analyzed (Table 2). We also observed variation in the *cox1-*ai5β and *cox1-*ai5γ intron. We could not find reports documenting absence of these introns within *S. cerevisiae*. However, inter- and intra-specific variation in other *Saccharomyces* species has been observed [26]; *cox1-*ai5β was prevalent throughout the genus but absent in *S. kudriavzevii* and the *cox1-*ai5γ was variable between *S. paradoxus* isolates. Five introns interrupt the *cob* gene in *S. cerevisiae* and “long” (containing all five introns) and “short” (containing just the last two introns) forms of this gene have been reported [15]. Consistent with these observations, the final two introns (*cob-*bi4 and -bi5) were invariably present in all *S. cerevisiae* strains. We also found strains lacking just *cob*-bi1β or both *cob*-bi1β and *cob*-bi2 introns, indicating that additional intron mosaics exist in natural populations.

**Table 2:** Intron content within and between *Saccharomyces* mtDNAs

Intron		*cox1*											*cob*						*rnl*
		ai1	ai2	ai3α	ai3β	ai3γ	ai4α	ai4β	ai4γ	ai5α	ai5β	ai5γ	bi1α	bi1β	bi2	bi3	bi4	bi5	Ω
		I1	I2	I3α	I3β	I3γ	I4α	I4β		I5α	I5β	I5α	I1α	I1β	I2	I3	I4	I5	
Group	(n)	II	II	I	I**	I	I	I	I**	I	I*	II*	I**	II	I	I	I*	I*	I
all *cerevisiae*	103	59	51	91	0	40	59	38	0	25	80	74	5	86	89	96	100	100	39
Mosaic-A	32	66	47	97	0	34	81	59	0	38	94	72	0	81	81	91	100	100	63
N. American	3	0	100	0	0	0	0	0	0	100	100	100	0	100	100	100	100	100	0
Malaysian	1	-	+	-	-	+	-	+	-	-	+	-	-	-	-	+	+	+	-
sake	5	100	80	100	0	40	40	0	0	20	100	100	0	20	20	80	100	100	0
wine/Euro	34	38	59	94	0	41	21	12	0	9	76	84	0	100	100	100	100	100	9
Mosaic-B	23	74	43	96	0	52	91	65	0	13	74	70	22	100	100	100	100	100	74
West African	5	100	20	80	0	20	100	0	0	80	0	0	0	40	100	100	100	100	0
Unknown	1	+	-	+	+	-	-	-	-	+	+	-	-	-	-	-	-	-	-
*paradoxus*	3	0	33	0	100	67	0	0	0	67	100	33	67	67	67	67	0	0	?
*mikatae*	1	+	-	+	+	-	+	-	-	+	+	+	+	+	+	+	+	+	?
*kudriavzevii*	1	-	-	+	+	-	+	-	-	+	-	+	+	+	+	+	-	-	?
*cariocanus*	1	-	-	-	+	-	-	-	-	-	+	+	+	+	+	-	-	-	?
*bayanus*	1	-	-	-	+	+	-	-	+	+	+	+	-	+	+	+	-	+	?
Insertion site		169	205	243	386	709	720	900	927	972	1107	1132	393	415	429	506	756	807	2716

The percentage of strains containing specific introns is provided for *S. cerevisiae* strains and related *Saccharomyces* species. The number of strains within each population is provided (n). When n = 1, intron presence/absence is indicated as +/−. Introns not known as variable (*) or observed (**) in *S. cerevisiae* are indicated next to their respective group heading. Insertion sites indicate the base pair in the CDS that precedes the intron insertion site.

Though mitochondrial intron content is known to vary in a strain-dependent manner, comparative studies have revealed that the occurrence of certain introns follows species divisions [26, 43]*.* The *cox1-*ai3β intron has never been found in *S. cerevisiae* species, and our findings were true to this classification. Similarly, we did not find evidence of a rare *cox1* intron (ai4γ) that has only been observed in *S. bayanus.* We did, however, find evidence of the *cob*-bI1α intron in a small number of strains (5 %). To our knowledge, this intron has not previously been reported in *S. cerevisiae* but has been observed in much of the *Saccharomyces* genus [26]*.* Interestingly, strains with this intron also contained the five common *S. cerevisiae* introns, and showed no evidence of introgression of *cob* exon sequences.

One exception to the species-specific intron structure was the mosaic strain, YJM1399. This strain contained the *cox1-*ai3β intron (inserted at the same location as in other species), and lacked the otherwise conserved *cob-*bi4 and –bi5 introns. The sequence of the *cox1-*ai3β intron most closely matched a sequence from the distantly related yeast *Lachancea meyersii* (Genbank: HE983614.1, 53 % coverage, 92 % identity) and shared little, if any, homology with the *cox1-*ai3β intron from *S. paradoxus.* Other portions of the mitochondrial genome were highly homologous to those of other *S. cerevisiae* strains.

To determine if intron profiles were related to population structure, we compared intron content between each population (Table 2). The highly prevalent *cob-*bi1β and *cob-*bi2 introns were nearly absent in the sake population and Malaysian strain. No West African strain contained the *cox1-*ai5β or -ai5γ intron (previously thought to be conserved). The nearly identical mtDNA sequences of the three North American isolates lacked the frequently observed *cox1-*ai3*α* intron. A phylogenetic tree based on character states of intron presence/absence (Additional file 7: Figure S3) was highly concordant with the phylogeny built from coding sequences (Figure 1); West African, sake/Malaysian, and North American strains were organized into distinct groups (with one exception within the sake strains). Intron patterns of wine/European and mosaic strains were more variable. Individual intron profiles for each strain are reported in Additional file 8: Table S5.

## Discussion and conclusions

Comparisons of mtDNAs between *Saccharomyces* species and other *Hemiascomycetes* yeasts have revealed broad evolutionary changes in mtDNA evolution, particularly in regards to genome organization [12, 44, 45]. Few population genetic investigations on intraspecific mtDNAs in yeasts exist [45–47], and none for *Saccharomyces* species. To provide a window onto recent evolutionary changes in the mtDNA, we compared the intraspecific genetic variation in mitochondrial genomes from 100 strains of *S. cerevisiae.*

Through phylogenetic analysis of coding sequences, we found that these mtDNAs were organized into three broad clades that shared remarkable resemblance to clades constructed using the thousands of SNPs across nuclear sequences [4, 6], albeit at lower resolution. Populations according to nuclear divergences organize strains into 5 non-mosaic populations (wine/European, West African, sake, Malaysian, and North American). From mitochondrial coding sequences alone, discrete populations for West African and wine/European ancestry were observed. Resolution of North American and Asian mtDNAs clades were more obscured, although each maintained distinct lineages within a broader clade. Strains with known mosaic ancestry grouped predominantly as two subclades peripheral to the wine/European or Asian/North American clades. Many of these mosaics are strains domesticated for human activities (or perhaps the result of admixing between wild and domesticated strains [48]). High prevalence of mitochondrial mosaic strains were intermixed among the wine/European and Asian strains suggests that mitochondrial genomes, like their nuclear counterparts, also contain mixed ancestry. This implies that mitochondrial recombination occurs frequently during admixture.

Mobile elements, including introns, also had population specific profiles. Intron pattern were not fixed within each population (wine/Euro, West African, etc.), but substantial trends between populations emerged. One example is in the optional *cox1-*ai1 intron, which was omnipresent in all sake and West African strains but only found in 38 % of wine/European strains and none of the North American isolates. Newly discovered variation in introns previously thought fixed in *S. cerevisiae* was also related to population structure; the *cob-*bi1β intron was only missing in sake and West African strains while being highly prevalent in all other populations. Similarly, the *cob-*bi1α intron, never previously reported within *S. cerevisiae* mtDNAs, was observed only in a small number of mosaic strains whose mtDNAs were most closely related to those of the wine/European strains (our “mosaic-B” group). While the ancestry of these mosaic strains are a subject of speculation, intron flanking sequences in their *cob* exons were more similar to the *cob* genes in *S. cerevisiae* than *S. paradoxus*, suggesting that this intron is not the result of introgression of a non-native *cob* allele. Intron analysis also revealed an interesting mitochondrial ancestry for mosaic strain, YJM1399. This strain contained an intron at the (non-*cerevisiae*) *cox1-*aI3β insertion site that most closely resembled a sequence from *Lachancea*, and contained an intron-less *cob* despite complete conservation of several *cob* introns throughout the rest of the *S. cerevisiae* strains. Several fixed substitutions in the coding sequences of this mosaic relative to *S. cerevisiae* and *S. paradoxus* suggested this was a non-*cerevisiae* mtDNA. Other regions of the mtDNA, however, were homologous to *S. cerevisiae* mtDNA. This mosaic strain likely provides an example of mitochondrial introgression and not replacement.

Genetic diversity in *S. cerevisiae* extends past what has been measured here [7], and it is likely that as larger genetic space is sampled, additional mitochondrial mosaics and intron variants will be revealed. Analysis of allelic variation within intron sequences may provide deeper insight into mitochondrial evolution, however, we found that a simple binary presence/absence analysis was sufficient to reconstruct the populations described here. Insertion mechanisms that occur during intron homing are mutagenic to residues in flanking sequences [49, 50]. Thus, the phylogenies created by coding sequences are likely influenced by population-specific intron profiles and the accompanying co-conversion of exon sequences.

Patterns of GC clusters also demonstrated population structure. The total numbers of clusters ranged from an average of ~76 in North American strains to ~153 clusters in West African strains. Each population had significantly different patterns in the numbers and types of GC clusters, however the West African strains appear to have undergone a recent expansion of the rare M4 cluster. While M4 clusters can be at the first position of a tandem GC cluster array [17], the M4 clusters in the West African strains were almost exclusively found following an M1 cluster. M2 clusters generally paired with M3 clusters in tandem arrays.

Phenotypic variation in wild yeasts is believed to follow population history [51]. The population specific genetic structure in mtDNAs may play a role in phenetic groupings. In *S. cerevisiae,* strains with a common nuclear background but harboring different mtDNAs had slightly different growth rates [52], demonstrating that naturally variation in mtDNAs can affect phenotype. It is easy to imagine how allelic differences in oxidative phosphorylation genes (and corresponding gene networks) could contribute to different efficiencies in mitochondrial respiration. Intergenic mtDNA sequences may also contribute to phenotypic differences, directly or indirectly. Mobile GC clusters affecting recombination [22] may influence mtDNA replication and inheritance or interfere with mitochondrial translation (as in the case of translational bypass elements that are believed to have evolved from GC clusters [53]). GC clusters are also correlated with petite frequency in *Saccharomyces* yeasts [21]. Possibly, the elevated petite frequency observed in the West African-related laboratory strain, SK1 [54], is due to an elevated number of GC clusters predicted in this phylogenetically distinct group. While *S. cerevisiae* introns are not essential for mitochondrial respiration [55], they may offer an underappreciated regulatory role [56–58]. Absence of introns in certain populations may relax selection on the nuclear encoded splicing factors, thus contributing to the creation of cytonuclear incompatibilities that play a role in post-zygotic speciation in these lower eukaryotes [26–30].

The whole-genome duplication preceding the evolution of the *Saccharomyces* genus is thought to have relaxed selection on mitochondrial functions, as evidenced by increased nonsynonymous mutations and relaxed codon bias in mitochondrially-targeted nuclear genes involved in respiration [59]. We observed more frequent nonsynonymous polymorphisms in *atp6*, consistent with relaxed purifying selection on this gene. The nonsynonymous to synonymous ratio of intraspecific polymorphisms for *atp6* (pN/pS = 0.172) was actually higher than that for the *rps3/VAR1* excluding indel variations (pN/pS = 0.142), a mitochondrial gene known for intraspecific diversity [19]. Interestingly, this mirrors intraspecific polymorphisms observed for the *atp6* gene in *L. kluyveri* [47], a yeast that evolved before the whole genome duplication. This gene has also been implicated in tests of positive selection in other organisms [60–62]. There were almost no nonsynonymous substitutions between *S. cerevisiae* and *S. paradoxus*, precluding formal tests of selection. Even if a distinct evolutionary pattern for *atp6* exists, it is likely that these closely related species are under very similar selection patterns.

In Saccharomyces, mitochondrial population genetics studies have been limited by low numbers of available mtDNA sequences. High coverage sequencing projects routinely produce only partial mitochondrial sequences [8, 40]. The recovery of complete mtDNAs proves that robust methodologies can be used to reconstruct these challenging genomes from Illumina-based datasets [6, 47]. Here, we have shown that high quality mtDNA sequences can be produced from single molecule sequencing data. While Illumina sequencing proves to be useful for large-scale mitochondrial genome sequencing, the PacBio RS platform offers a cost-efficient method when only a small number of mitochondrial genomes are required.

## Methods

### Isolation of mtDNA

Prior to DNA isolation, crudely purified mitochondria were prepared as previously described [63]. Strain NCYC3594 (a haploid derivative of YJM975 [38]) was grown overnight at 30 °C in 1.5 L YPD media (1 % yeast extract, 2 % peptone, 2 % dextrose). Late-log phase cells were harvested, washed, and incubated for 10 min at 30 °C in approximately 30 ml of 100 mM Tris-SO_4_ pH 9.4 containing 10 mM DTT. Cells were resuspended in 1.2 M sorbitol, 20 mM KH_2_PO_4_ pH 7.4 (7 ml/g wet weight cells) containing 0.5 mg/ml Zymolase 20T (Nacalai Tesque Inc.) and incubated while rocking at 30 °C until spheroplasts occurred (~45 min., determined by optical clearing of 50 μl cell suspension added to 0.5 ml H_2_0), followed by physical shearing of cells using a 40 ml tissue grinder. Mitochondria were separated from unbroken cells and cell debris through alternating rounds of centrifugation of 5 min at 5000 rpm and 12 min 12000 rpm in a Sorvall F21S-8x50Y rotor.

Mitochondrial fractions for strain NCYC3585 (a haploid derivative of 273614N [38] were collected as described above, except that cells were grown in YPEG media (1 % yeast extract, 2 % peptone, 3 % ethanol, 3 % glycerol). These mitochondrial enrichments were also subjected to a DNase treatment [64] by incubating in 1 ml 0.3 M sucrose, 5 mM MgCl_2_, 50 mM Tris–HCl pH 8.0, 10 mM CaCl_2_ containing 100 units of DNase (New England BioLabs) for 30 min. at 37 °C. DNases were inactivated by the addition of 0.5 M EDTA (pH 8.0) to a final concentration of 0.2 M. The mitochondria were washed to remove DNases through 3 repeated cycles of centrifugation (15000 rpm at 4 °C, 10 min) and resuspended in sucrose buffer.

The resulting mitochondria-enriched cell fractions were lysed in ~500 μl of 1 % Sarkosyl, 100 mM NaCl, 10 mM EDTA pH 8.0, Tris pH 8.0, and incubated at room temperature until optical clearing occurred (~30 min). DNA was isolated using phenol-chloroform extraction and ethanol precipitation. Purity of mtDNA (O.D. 260/280 = 1.8-2.0) was determined using a NanoDrop-1000 spectrophotometer.

### Real Time PCR

Real-time PCR was used to determine the relative abundance of nuclear and mitochondrial DNA. Primer sequences for *ACT1* (5′ GTATGTGTAAAGCCGGTTTTG and 5′CATGATACCTTGGTGTCTTGG) and *cox1* (5′ CTACAGATACAGCATTTCCAAGA and 5′ GTGCCTGAATAGATGATAATGGT) were obtained from Taylor, et. al. [65]. Each gene was amplified from dilutions of the purified mtDNAs using SYBR Green master mix (ABi Research) on an ABi 7300 Real Time PCR System. The ratio of mtDNA to nuclear DNA was determined as the logarithm of the difference in the cycle threshold values (log_2_ ΔCT), after correcting for genome size.

### Sequencing

Approximately 2 μg of purified mtDNAs were sequenced using the PacBio RS at the Yale Center for Genomic Analysis following circular consensus sequencing protocols. A library created from ~800 bp fragments of the mtDNA from NCYC3594 was sequenced using C1 chemistry, producing 38,681 reads. A library created from ~6 kbp fragments of fractionated mtDNA from NCYC3585 was sequenced using C2 chemistry, producing 7,360 reads. A single SMRT Cell was used for each library. Reads less than 50 bp were removed.

### Mitochondrial genome assembly and annotation

Circular consensus reads were assembled using MIRA v3.4.1 (NCYC3594) and MIRA 3.9.17 (NCYC3585) with parameters: denovo, genome, accurate [66]. The assembly for NCYC3585 produced two major contigs that overlapped (identified using MUMmer 3.0 [67]) by ~3000 bp and were manually joined to form a single scaffold. Consensus sequences and assembly qualities of the final scaffolds were improved by employing the Quiver consensus algorithm to map reads back to contigs and correct sequencing errors [37].

The average read quality for NCYC3594 was Q16.24, which improved to an average assembly quality of Q17.85 after Quiver. The average quality for the 273614N reads was Q15.95, which improved to Q45.83 after Quiver. Hand curation of each genome revealed a large duplicated sequence in an AT-rich intergenic region of NCYC3594 and was manually removed. Sequences were annotated using MFANNOT [24]. The annotations were verified with BLAST searches of features in the reference sequence [15]. Annotations included only tRNA sequences that were triply identified by MFANNOT, BLAST, and tRNAscan-SE [68]. Annotations were manually curated to ensure correct intron/exon boundaries.

The mitochondrial genome from *S. cerevisiae* is organized as a collection of linear concatemers that map to a circular genome [69, 70]. Consistent with circularity, we found reads that aligned to both ends of each linear scaffold (≥360 bp on both ends with > 80 % identity). The linear scaffolds were reorganized to match the S288C reference genome start position.

### Alignments and phylogenetics

Mitochondrial protein coding sequences were extracted from 99 strains. Strain names and accession numbers are provided in Additional file 2: Table S2. A 6765 bp alignment of the concatenated CDS was generated utilizing Clustal Omega [71]. A neighbor-joining [72] tree was constructed utilizing MEGA6 [73]. The *S. paradoxus* strain CBS432 was used as an outgroup. The proportion of synonymous to non-synonymous polymorphisms within *S. cerevisiae* (pN/pS) was calculated using PAML [74, 75].

An initial multiple alignment was constructed using the LAGAN [76] algorithm in the mVISTA suite of programs [77] using the mtDNA sequences from S288C, YJM789, NCYC3594, and NCYC3585. Sequences from YJM1078, YJM1273, YJM1388, YJM1401, and YJM1439 were added to the multiple alignment utilizing MAFTT [78]. Alignments were manually curated to fix exon/intron boundaries and indels. Nucleotide diversity was calculated to allow for multiple minor alleles using the per site summation method. Genome polymorphism was assessed by counting individual polymorphic sites in sliding windows of 100 bp in 50 bp steps across the multiple alignment.

### Additional analyses

GC clusters classifications were defined according to Weiller et al. [17], using consensus sequences (Additional file 9: Table S6). The GC clusters were identified using BLAST. GC-rich areas (>60 % GC) not matching consensus sequences were identified through 30 bp sliding window scans. Tandem GC cluster assays were defined as GC-rich regions that overlapped with at least two classified clusters. One-way ANOVAs were performed within each classification (count ~ population) and significance determined following a Bonferonni correction. Conserved GC clusters were identified as sites in the multiple alignment with overlapping GC-rich regions, followed by manual verification*.* Genome wide scans for repetitive elements in the mtDNAs from S288C, NCYC3594 and YJM789 were performed utilizing Jellyfish [79] with k-mer values of 15, 30, 50, and 100.

Intronic sequences were identified using BLAST with query sequences from S288C (c*ox1*-aI1α, aI2, aI3α, aI5α, aI5β, aI5γ, *rnl-*I1), YJM789 (*cox1-*aI3γ, aI4β) and *S. paradoxus* CBS432 (*cox1-*aI3β). Introns were classified as 0 or 1 based on clear presence of a homologous intron at that specific site and agreement with prior annotations (with the exception of aI3β in YJM1399, which shares the insertion site but no homology with *S. paradoxus*). Additional intron information was obtained from unassembled contigs from http://www.moseslab.csb.utoronto.ca/sgrp/ [40], based on identification of coding sequences in contigs and the presence of intervening sequence between exons. A distance matrix was calculated utilizing presence of an intron at the specific insertion sites as binary character trait, and a neighbor-joining tree was constructed with the ape package in R [80]. The strain NCIM3107 was omitted due to the presence of intervening sequences between exons atypical of the pattern observed in all other strains.

### Availability of supporting data

The annotated mtDNA sequences are available as Genbank accession numbers KR260476 (strain NCYC3594) and KR260477 (NCYC3585). Phylogenetic data has been deposited at TreeBase (http://purl.org/phylo/treebase/phylows/study/TB2:S17639). All scripts are available upon request.

## Additional files

Additional file 1: Table S1. Concordence of sequence data. Polymorphic sites between PacBio and the Illumina-generated datasets from [6]. Additional file 2: Table S2. Strain list. Strain names and accession numbers are provided. Additional file 3: Table S3. Nucleotide diversities. Polymorphic assessment, including nucleotide diversity (π), for each mitochondrial gene in *S. cerevisiae* (excluding YJM1399). Percent difference was calculated as (π*100). pN/pS indicates the ratio of nonsynonymous to synonymous polymorphism. Additional file 4: File S1. An interactive multiple alignment of the nine complete mtDNAs from S. cerevisiae. To move to a particular feature, select the feature name from the searchable list on the left. Nucleotide positions for each strain represent the ungapped genomes, while positions in the consensus sequence represent the position in the gapped multiple alignment. Additional file 5: Table S4. GC clusters. For each strain, the total counts of GC clusters in all classes are provided. Additional file 6: File S2. Repetitive elements. A k-mer analysis of repetitive sequences in strains S2883 and NCYC3594. Each square represents a single, potentially overlapping, k-mer. Additional file 7: Figure S3. Intron phylogenetics. A phylogenetic tree built from intron profiling, treated as binary character states. Any strain with a questionable intron profile was excluded. Additional file 8: Table S5. Mitochondrial introns. Individual intron profiles for each strain are provided, where 1 indicates the presence of the intron and a blank cell, the absence. Additional file 9: Table S6. GC cluster consensus sequences. Slightly modified from [17] to remove 5′ TAG motif.
