# Supplementary material for: Population structure of mitochondrial genomes in Saccharomyces cerevisiae
Source: BMC Genomics. 2015 Jun 11;16(1):451. doi: 10.1186/s12864-015-1664-4 (PMC4464245; doi:10.1186/s12864-015-1664-4)
Supplement: Additional file 4: File S1. — An interactive multiple alignment of the nine complete mtDNAs from S. cerevisiae. To move to a particular feature, select the feature name from the searchable list on the left. Nucleotide positions for each strain represent the ungapped genomes, while positions in the consensus sequence represent the position in the gapped multiple alignment. [file 12864_2015_1664_MOESM4_ESM.zip › 12864_2015_1664_MOESM4_ESM.html]

Multiple Sequence Alignment of Saccharomyces cerevisiae Mitochondrial Genomes


Toggle navigation

Multiple Sequence Alignment of Saccharomyces cerevisiae Mitochondrial Genomes

|  |
| --- |

| Sequences |
| --- |
| trnP(ugg) |
| rns |
| trnW(uca) |
| cox1-E1 |
| cox1-aI1 |
| cox1-E2 |
| cox1-aI2 |
| cox1-E3 |
| cox1-aI3alpha |
| cox1-E4 |
| cox1-E4.2 |
| cox1-aI4alpha |
| cox1-E5 |
| cox1-E5.2 |
| cox1-aI5alpha |
| cox1-E6 |
| cox1-aI5beta |
| cox1-E7 |
| cox1-aI5gamma |
| cox1-E8 |
| atp8 |
| atp6 |
| trnE(uuc) |
| cob-E1 |
| cob-bI1 |
| cob-E2 |
| cob-bI2 |
| cob-E3 |
| cob-bI3 |
| cob-E4 |
| cob-bI4 |
| cob-E5 |
| cob-bI5 |
| cob-E6 |
| atp9 |
| trnS(uga) |
| rps3 |
| rnl-E1 |
| rnl-I1 |
| rnl-E2 |
| trnT(ugu) |
| trnC(gca) |
| trnH(gug) |
| trnL(uaa) |
| trnQ(uug) |
| trnK(uuu) |
| trnR(ucu) |
| trnG(ucc) |
| trnD(guc) |
| trnS(gcu) |
| trnR(acg) |
| trnA(ugc) |
| trnI(gau) |
| trnY(gua)\_2 |
| trnN(guu) |
| trnM(cau)\_1 |
| cox2 |
| trnF(gaa) |
| trnT(uuag) |
| trnV(uac) |
| cox3 |
| trnM(cau)\_2 |
| rnpB |
| cox1-aI3gamma |
| cox1-aI4beta |
| trnY(gua) |
| cox1-aI4gamma |
| G-orf131 |
| cox1-aI3alpha-orf99 |
| G-trnP(ugg) |
| G-rns |
| G-trnW(uca) |
| G-cox1-E1 |
| G-cox1-aI1 |
| G-cox1-E2 |
| G-cox1-aI2 |
| G-cox1-E3 |
| G-cox1-aI3alpha |
| G-cox1-E4 |
| G-cox1-aI3gamma |
| G-cox1-E4.2 |
| G-cox1-aI4alpha |
| G-cox1-E5 |
| G-cox1-aI4beta |
| G-cox1-E5.2 |
| G-cox1-aI5alpha |
| G-cox1-E6 |
| G-cox1-aI5beta |
| G-cox1-E7 |
| G-cox1-aI5gamma |
| G-cox1-E8 |
| G-atp8 |
| G-atp6 |
| G-trnE(uuc) |
| G-cob-E1 |
| G-cob-bI1 |
| G-cob-E2 |
| G-cob-bI2 |
| G-cob-E3 |
| G-cob-bI3 |
| G-cob-E4 |
| G-cob-bI4 |
| G-cob-E5 |
| G-cob-bI5 |
| G-cob-E6 |
| G-atp9 |
| G-trnS(uga) |
| G-rps3 |
| G-rnl-E1 |
| G-rnl-I1 |
| G-rnl-E2 |
| G-trnT(ugu) |
| G-trnC(gca) |
| G-trnH(gug) |
| G-trnL(uaa) |
| G-trnQ(uug) |
| G-trnK(uuu) |
| G-trnR(ucu) |
| G-trnG(ucc) |
| G-trnD(guc) |
| G-trnS(gcu) |
| G-trnR(acg) |
| G-trnA(ugc) |
| G-trnI(gau) |
| G-trnY(gua)\_2 |
| G-trnN(guu) |
| G-trnM(cau)\_1 |
| G-cox2 |
| G-trnF(gaa) |
| G-trnT(uuag) |
| G-trnV(uac) |
| G-cox3 |
| G-trnM(cau)\_2 |
| G-rnpB |

| Sequence | Start | Alignment | End |
| --- | --- | --- | --- |
|
| S288C | 1 | TTCATAATTAA-TTTTTTATATATATATTATATTATAATATTAATTTATATTATAAAAATAATATTTATTATTAAAATATTTATTCTCCTTTCGGGGTTC | 99 |
| YJM1388 | 1 | TTCATAATTAA-TTTTTTATATATATATTATATTATAATATTAATTTATATTATAAAAATAATATTTATTATTAAAATATTTATTCTCCTTTAGGGGTTC | 99 |
| YJM789 | 1 | TTCATAATTAATTTTTTTATATATATATTATATTATAATATTAATTTATATTATAAAAATAATATTTATTATTAAAATATTTATTCTCCTTTCGGGGTTT | 100 |
| YJM1273 | 1 | TTCATAATTAA-TTTTTTATATATATATTATATTATAATATTAATTTATATTATAAAAATAATATTTATTATTAAAATATTTATTCTCCTTTCGGGGTTC | 99 |
| NCYC3585 | 1 | TTCATAATTAATTTTTTTATATATATATTATATTATAATATTAATTTATATTATAAAAATAATATTTATTATTAAAATATTTATTCTCCTTTCGGGGTTT | 100 |
| YJM1401 | 1 | TTCATAATTAA-TTTTTTATATATATATTATATTATAATATTAATTTATATTATAAAAATAATATTTATTATTAAAATATTTATTCTCCTTTAGGGGTTC | 99 |
| NCYC3594 | 1 | TTCATAATTAA-TTTTTTATATATATATTATATTATAATATTAAT-----TTATAAAAATAATATTTATTATTAAAATATTTATTCTCCTTTCGGGGTTC | 94 |
| YJM1078 | 1 | TTCATAATTAA-TTTTTTATATATATATTATATTATAATATTAATTTATATTATAAAAATAATATTTATTATTAAAATATTTATTCTCCTTTCGGGGTTC | 99 |
| YJM1439 | 1 | TTCATAATTAA-TTTTTTATATATATATTATATTATAATATTAATTTATATTATAAAAATAATATTTATTATTAAAATATTTATTCTCCTTTCGGGGTTC | 99 |
| consensus | 1 | \*\*\*\*\*\*\*\*\*\*\*-\*\*\*\*\*\*\*\*\*\*\*\*\*\*\*\*\*\*\*\*\*\*\*\*\*\*\*\*\*\*\*\*\*-----\*\*\*\*\*\*\*\*\*\*\*\*\*\*\*\*\*\*\*\*\*\*\*\*\*\*\*\*\*\*\*\*\*\*\*\*\*\*\*\*\*\*.\*\*\*\*\*\*. | 100 |
|
| S288C | 100 | CGGCTCCCGTGGCCGGGCCCCGGAATTATTAATTAATAATAAATTATTATTAATAATTATTTATTATTTTATCATTAAAATATATAAATA-------AAA | 192 |
| YJM1388 | 100 | CGGCTCCCGTGGCCGGCCCCCGGAACTATTAATTAATAATAAATTATTATTAATAATTATTTATTATTTTATTATTAAAATATATAAATAAAT----AAA | 195 |
| YJM789 | 101 | CGGCTCCCGTGGCCGGGCCCCGGAACTATTAATTAATAATAAATTATTATTAATAATTATTTATTATTTTATTATTAAAATATATAAATAAAT----AAA | 196 |
| YJM1273 | 100 | CGGCTCCCGTGGCCGGCCCCCGGAACTATTAATTAATAATAAATTATTATTAATAATTATTTATTATTTTATTATTAAAATATATAAATAAAT----AAA | 195 |
| NCYC3585 | 101 | CGGCTCCCGTGGCCGGGCCCCGGAACTATTAATTAATAATAAATTATTATTAATAATTATTTATTATTTTATTATTAAAATATATAAATAAAT----AAA | 196 |
| YJM1401 | 100 | CGGCTCCCGTGGCCGGCCCCCGGAACTATTAATTAATAATAAATTATTATTAATAATTATTTATTATTTTATTATTAAAATATATAAATAAAT----AAA | 195 |
| NCYC3594 | 95 | CGGCTCCCGTGGCCGGGCCCCGGAATTATTAATTAATAATAAATTATTATTAATAATTATTTATTATTTTATCATTAAAATATATAAATAAAT----AAA | 190 |
| YJM1078 | 100 | CGGCTCCCGTGGCCGGCCCCCGGAACTATTAATTAATAATAAATTATTATTAATAATTATTTATTATTTTATTATTAAAATATATAAATAAAT---AAAA | 196 |
| YJM1439 | 100 | CGGCTCCCGTGGCCGGGCCCCGGAACTATTAATTAATAATAAATTATTATTAATAATTATTTATTATTTTATTATTAAAATATATAAATAAATAAATAAA | 199 |
| consensus | 101 | \*\*\*\*\*\*\*\*\*\*\*\*\*\*\*\*.\*\*\*\*\*\*\*\*.\*\*\*\*\*\*\*\*\*\*\*\*\*\*\*\*\*\*\*\*\*\*\*\*\*\*\*\*\*\*\*\*\*\*\*\*\*\*\*\*\*\*\*\*\*\*.\*\*\*\*\*\*\*\*\*\*\*\*\*\*\*\*\*------.\*\*\* | 200 |
|
| S288C | 193 | AATATTAAAAAGAT-AAAAAAAATAATGTTTATTCTTTA-TATAAATTATATATATATATA--------TAATTAATTAATTAATTAATTAATTAATAAT | 282 |
| YJM1388 | 196 | AATATTAAAAAGAT-AAAAAAAATAATGTTTATTCTTTATTATAAATTATATATATATATATATATAATTAATTAATTAATTAATTAATTAATTAATAAT | 294 |
| YJM789 | 197 | AATATTAAAAAGAT--AAAAAAATAATGTTTATTCTTTATTATAAATTATATATATATATATATA----TAATTAATTAATTAATTAATTAATTAATAAT | 290 |
| YJM1273 | 196 | AATATTAAAAAGAT-AAAAAAAATAATGTTTATTCTTTATTATAAATTATATATATATATATATATAATTAATTAATTAATTAATTAATTAATTAATAAT | 294 |
| NCYC3585 | 197 | AATATTAAAAAGAT--AAAAAAATAATGTTTATTCTTTATTATAAATTATATATATATATATATA----TAATTAATTAATTAATTAATTAATTAATAAT | 290 |
| YJM1401 | 196 | AATATTAAAAAGAT-AAAAAAAATAATGTTTATTCTTTATTATAAATTATATATATATATATATATAATTAATTAATTAATTAATTAATTAATTAATAAT | 294 |
| NCYC3594 | 191 | AATATTAAAAAGATA-AAAAAAATAATGTTTATTCTTTA-TATAAATTATATATATATATATATAAT--TAATTAATTAATTAATTAATTAATTAATAAT | 286 |
| YJM1078 | 197 | AATATTAAAAAGAT--AAAAAAATAATGTTTATTCTTTATTATAAATTATATATATATATATATA----TAATTAATTAATTAATTAATTAATTAATAAT | 290 |
| YJM1439 | 200 | AATATTAAAAAGAT--AAAAAAATAATGTTTATTCTTTATTATAAATTATATATATATATATATATAATTAATTAATTAATTAATTAATTAATTAATAAT | 297 |
| consensus | 201 | \*\*\*\*\*\*\*\*\*\*\*\*\*\*--\*\*\*\*\*\*\*\*\*\*\*\*\*\*\*\*\*\*\*\*\*\*\*-\*\*\*\*\*\*\*\*\*\*\*\*\*\*\*\*\*\*\*\*\*----..--\*\*\*\*\*\*\*\*\*\*\*\*\*\*\*\*\*\*\*\*\*\*\*\*\*\*\*\*\*\*\* | 300 |
|
| S288C | 283 | AAAAATATAATTATAAATAATATAAATATTATTCTTTATTAATAAATATATATTTATATATTATAAAAGTATCTTAATTAATAAAAATAAACATTTAATA | 382 |
| YJM1388 | 295 | AAAAATATAATTATAAATAATATAAATATTATTCTTTATTAATAAATATATATTTATATATTATAAAAGTATCTTAATTAATAAAAATAAACATTTAATA | 394 |
| YJM789 | 291 | AAAAATATAATTATAAATAATATAAATATTATTCTTTATTAATAAATATATATTTATATATTATAAAAGTATCTTAATTAATAAAAATAAACATTTAATA | 390 |
| YJM1273 | 295 | AAAAATATAATTATAAATAATATAAATATTATTCTTTATTAATAAATATATATTTATATATTATAAAAGTATCTTAATTAATAAAAATAAACATTTAATA | 394 |
| NCYC3585 | 291 | AAAAATATAATTATAAATAATATAAATATTATTCTTTATTAATAAATATATATTTATATATTATAAAAGTATCTTAATTAATAAAAATAAACATTTAATA | 390 |
| YJM1401 | 295 | AAAAATATAATTATAAATAATATAAATATTATTCTTTATTAATAAATATATATTTATATATTATAAAAGTATCTTAATTAATAAAAATAAACATTTAATA | 394 |
| NCYC3594 | 287 | AAAAATATAATTATAAATAATATAAATATTATTCTTTATTAATAAATATATATTTATATATTATAAAAGTATCTTAATTAATAAAAATAAACATTTAATA | 386 |
| YJM1078 | 291 | AAAAATATAATTATAAATAATATAAATATTATTCTTTATTAATAAATATATATTTATATATTATAAAAGTATCTTAATTAATAAAAATAAACATTTAATA | 390 |
| YJM1439 | 298 | AAAAATATAATTATAAATAATATAAATATTATTCTTTATTAATAAATATATATTTATATATTATAAAAGTATCTTAATTAATAAAAATAAACATTTAATA | 397 |
| consensus | 301 | \*\*\*\*\*\*\*\*\*\*\*\*\*\*\*\*\*\*\*\*\*\*\*\*\*\*\*\*\*\*\*\*\*\*\*\*\*\*\*\*\*\*\*\*\*\*\*\*\*\*\*\*\*\*\*\*\*\*\*\*\*\*\*\*\*\*\*\*\*\*\*\*\*\*\*\*\*\*\*\*\*\*\*\*\*\*\*\*\*\*\*\*\*\*\*\*\*\*\*\* | 400 |
|
| S288C | 383 | ATATGAATTATATATTATTATTATTATTAATAAAATTATTAATAATAATCAATATGAAATTAATAAAAATCTTAT-AAAAAAGTAATGAAT--------- | 472 |
| YJM1388 | 395 | ATATGAATTATATATTATTATTATTATTTATAAAATTATTAATAATAATCAATATGAAATTAATAAAAATTTTAT-AAAAAAGTAATGAAT--------- | 484 |
| YJM789 | 391 | ATATGAATTATATATTATTATTATTATTTATAAAATTATTAATAATAATCAATATGAAATTAATAAAAATTTTAT-AAAAAAGTAATGAAT--------- | 480 |
| YJM1273 | 395 | ATATGAATTATATATTATTATTATTATTTATAAAATTATTAATAATAATCAATATGAAATTAATAAAAATTTTAT-AAAAAAGTAATGAAT--------- | 484 |
| NCYC3585 | 391 | ATATGAATTATATATTATTATTATTATTTATAAAATTATTAATAATAATCAATATGAAATTAATAAAAATTTTAT-AAAAAAGTAATGAAT--------- | 480 |
| YJM1401 | 395 | ATATGAATTATATATTATTATTATTATTTATAAAATTATTAATAATAATCAATATGAAATTAATAAAAATTTTAT-AAAAAAGTAATGAAT--------- | 484 |
| NCYC3594 | 387 | ATATGAATTATATATTATTATTATTATTTATAAAATTATTAATAATAATCAATATGAAATTAATAAAAATCTTAT-AAAAAAGTAATGAATACTCCTTCG | 485 |
| YJM1078 | 391 | ATATGAATTATATATTATTATTATTATTTATAAAATTATTAATAATAATCAATATGAAATTAATAAAAATTTTAT-AAAAAAGTAATGAAT--------- | 480 |
| YJM1439 | 398 | ATATGAATTATATATTATTATTATTATTTATAAAATTATTAATAATAATCAATATGAAATTAATAAAAATTTTATAAAAAAAGTAATGAAT--------- | 488 |
| consensus | 401 | \*\*\*\*\*\*\*\*\*\*\*\*\*\*\*\*\*\*\*\*\*\*\*\*\*\*\*\*.\*\*\*\*\*\*\*\*\*\*\*\*\*\*\*\*\*\*\*\*\*\*\*\*\*\*\*\*\*\*\*\*\*\*\*\*\*\*\*\*\*.\*\*\*\*-\*\*\*\*\*\*\*\*\*\*\*\*\*\*\*--------- | 500 |
|
| S288C | 473 | ------------------------ACTCCTTTTTAAAAATAAAAAGGGGTTCGGT--CCCCCCCCTTCCGTATACTTACGGGAGGGGGGTCCCTCACTCC | 546 |
| YJM1388 | 485 | ------------------------ATTCCTTTTTAAAAATA---------------------------------------------------------CC | 503 |
| YJM789 | 481 | ------------------------ATTCCTTTTTAAAAAT------------------------------------------------------------ | 496 |
| YJM1273 | 485 | ------------------------ATTCCTTTTTAAAAAT------------------------------------------------------------ | 500 |
| NCYC3585 | 481 | ------------------------ATTCCTTTTTAAAAAT------------------------------------------------------------ | 496 |
| YJM1401 | 485 | ------------------------ATTCCTTTTTAAAAAT------------------------------------------------------------ | 500 |
| NCYC3594 | 486 | GGGTTCGGTCCCCACGGGTCCCTCACTCCTTTTTAAAAATAAAAAGGGGTTCGGTCCCCCCCCCCTCCCGTATACTTACGGGAGGGGGGTCCCTCACTCC | 585 |
| YJM1078 | 481 | ------------------------ATTCCTTTTTAAAAAT------------------------------------------------------------ | 496 |
| YJM1439 | 489 | ------------------------ATTCCTTTTTAAAAAT------------------------------------------------------------ | 504 |
| consensus | 501 | ------------------------\*.\*\*\*\*\*\*\*\*\*\*\*\*\*\*--------------------------.--------------------------------- | 600 |
|
| S288C | 547 | TTCTTAATTAAATTATCTTAATTAAATTATCTTAATTAAATTATCTTAATTAAATTATCTTAATTAAATTATCTT-----AATTAAATTAAAAGGGGACT | 641 |
| YJM1388 | 504 | TTCTTAATTAAATA----------------------------------------------------------------------AAATAAAAAGGGGACT | 533 |
| YJM789 | 497 | -----------------------------------------------------ACCTTCTTAATTA--------------AATAAAATAAAAAGGGGACT | 529 |
| YJM1273 | 501 | -----------------------------------------------------ACCTTCTTAATTA--------------AATAAAATAAAAAGGGGACT | 533 |
| NCYC3585 | 497 | -----------------------------------------------------ACCTTCTTAATTA--------------AATAAAATAAAAAGGGGACT | 529 |
| YJM1401 | 501 | -----------------------------------------------------ACCTTCTTAATTA--------------AATAAAATAAAAAGGGGACT | 533 |
| NCYC3594 | 586 | TTCTTAATTAAATTATCTTAATTAAATTATCTTAATTAA--------------ATTATCTTAATTAAATTATCTTAATTAAATTAAATTAAAAGGGGACT | 671 |
| YJM1078 | 497 | -------------------------------------------------------------------ACCTTCTTAATTAAATAAAATAAAAAGGGGACT | 529 |
| YJM1439 | 505 | -----------------------------------------------------ACCTTCTTAATTA---------AATAAAATAAAATAAAAAGGGGACT | 542 |
| consensus | 601 | -------------.----------------------------------------...-----------...-------.----.\*\*\*\*.\*\*\*\*\*\*\*\*\*\*\* | 700 |
|
| S288C | 642 | TTATATTTATAAAGTAATTA------TATTAT------TATTATTATTATTATTTATTTATT---TTATTTTTATTATTTTATTATATATATTATATATT | 726 |
| YJM1388 | 534 | TTATATTTATAAAGTAATTA------TATTATTATTATTATTATTATTATTATTTATTTATTTA-TTTTTTTTATTATTTTATTATATATATTATATATT | 626 |
| YJM789 | 530 | TTATATTTATAAAGTAATTA------TATTAT------TATTATTATTATTATTTATTTATTTA-TTTTTTTTATTATTTTATTATATATATTATATATT | 616 |
| YJM1273 | 534 | TTATATTTATAAAGTAATTA------TATTAT------TATTATTATTATTATTTATTTATTTA-TTTTTTTTATTATTTTATTATATATATTATATATT | 620 |
| NCYC3585 | 530 | TTATATTTATAAAGTAATTA------TATTAT------TATTATTATTATTATTTATTTATTTA-TTTTTTTTATTATTTTATTATATATATTATATATT | 616 |
| YJM1401 | 534 | TTATATTTATAAAGTAATTATATTATTATTAT------TATTATTATTATTATTTATTTATTTA-TTTTTTTTATTATTTTATTATATATATTATATATT | 626 |
| NCYC3594 | 672 | TTATATTTATAAAGTAATTATATTTTTATTAT------TATTATTATTATTATTTATTTATTT---TTTTTTTATTATTTTATTATATATATTATATATT | 762 |
| YJM1078 | 530 | TTATATTTATAAAGTAATTATATTATTATTAT------TATTATTAT--TTATTTATTTATTT---TTTTTTTATTATTTTATTATATATATTATATATT | 618 |
| YJM1439 | 543 | TTATATTTATAAAGTAATTA------TATTAT------TATTATTATTATTATTTATTTATTTATTTTTTTTTATTATTTTATTATATATATTATATATT | 630 |
| consensus | 701 | \*\*\*\*\*\*\*\*\*\*\*\*\*\*\*\*\*\*\*\*----.-\*\*\*\*\*\*------\*\*\*\*\*\*\*\*\*--\*\*\*\*\*\*\*\*\*\*\*\*\*----\*.\*\*\*\*\*\*\*\*\*\*\*\*\*\*\*\*\*\*\*\*\*\*\*\*\*\*\*\*\*\*\*\* | 800 |
|
| S288C | 727 | AATACAGATAGAAGCCAAAAGGTCAGGCGCTTTCTTTGGGAGAAAGACCTAGTTAGTTCGAGTCTATCCTATCTGATAATAATTTAATTAACCATTAAAA | 826 |
| YJM1388 | 627 | AATACAGATAGAAGCCAAAAGGTCAGGCGCTTTCTTTGGGAGAAAGACCTAGTTAGTTCGAGTCTATCCTATCTGATAATAATTTAATTAAACATTAAAA | 726 |
| YJM789 | 617 | AATACAGATAGAAGCCAAAAGGTCAGGCGCTTTCTTTGGGAGAAAGACCTAGTTAGTTCGAGTCTATCCTATCTGATAATAATTTAATTAAACATTAAAA | 716 |
| YJM1273 | 621 | AATACAGATAGAAGCCAAAAGGTCAGGCGCTTTCTTTGGGAGAAAGACCTAGTTAGTTCGAGTCTATCCTATCTGATAATAATTTAATTAAACATTAAAA | 720 |
| NCYC3585 | 617 | AATACAGATAGAAGCCAAAAGGTCAGGCGCTTTCTTTGGGAGAAAGACCTAGTTAGTTCGAGTCTATCCTATCTGATAATAATTTAATTAAACATTAAAA | 716 |
| YJM1401 | 627 | AATACAGATAGAAGCCAAAAGGTCAGGCGCTTTCTTTGGGAGAAAGACCTAGTTAGTTCGAGTCTATCCTATCTGATAATAATTTAATTAAACATTAAAA | 726 |
| NCYC3594 | 763 | AATACAGATAGAAGCCAAAAGGTCAGGCGCTTTCTTTGGGAGAAAGACCTAGTTAGTTCGAGTCTATCCTATCTGATAATAATTTAATTAAACATTAAAA | 862 |
| YJM1078 | 619 | AATACAGATAGAAGCCAAAAGGTCAGGCGCTTTCTTTGGGAGAAAGACCTAGTTAGTTCGAGTCTATCCTATCTGATAATAATTTAATTAAACATTAAAA | 718 |
| YJM1439 | 631 | AATACAGATAGAAGCCAAAAGGTCAGGCGCTTTCTTTGGGAGAAAGACCTAGTTAGTTCGAGTCTATCCTATCTGATAATAATTTAATTAAACATTAAAA | 730 |
| consensus | 801 | \*\*\*\*\*\*\*\*\*\*\*\*\*\*\*\*\*\*\*\*\*\*\*\*\*\*\*\*\*\*\*\*\*\*\*\*\*\*\*\*\*\*\*\*\*\*\*\*\*\*\*\*\*\*\*\*\*\*\*\*\*\*\*\*\*\*\*\*\*\*\*\*\*\*\*\*\*\*\*\*\*\*\*\*\*\*\*\*\*\*\*.\*\*\*\*\*\*\*\* | 900 |
|
| S288C | 827 | AAAAGTATATATATTTATCATAATATATTAAATTTT-----ATTACAT-----TACAAATGAACACTTTTA-TTTATATTTATAAAAATATGA------- | 908 |
| YJM1388 | 727 | AAAAATATATATATTTATCATAATATATTAAATTTTATTACATTACAT-----TACAAATAAATACTTTTATTTTATATTTATAAAAATATGA------- | 814 |
| YJM789 | 717 | AAAAATATATATATTTATCATAATATATTAAATTTTATTACATTACAT-----TACAAATAAATACTTTTA-TTTATATTTATAAAAATATGA------- | 803 |
| YJM1273 | 721 | AAAAATATATATATTTATCATAATATATTAAATTTTATTACATTACAT-----TACAAATAAATACTTTTA-TTTATATTTATAAAAATATGA------- | 807 |
| NCYC3585 | 717 | AAAAATATATATATTTATCATAATATATTAAATTTTATTACATTACAT-----TACAAATAAATACTTTTATTTTATATTTATAAAAATATGA------- | 804 |
| YJM1401 | 727 | AAAAATATATATATTTATCATAATATATTAAATTTTATTACATTACAT-----TACAAATAAATACTTTTATTTTATATTTATAAAAATATGA------- | 814 |
| NCYC3594 | 863 | AAAAGTATATATATTTATCATAATATATTAAATTTTA-----TTACAT-----TACAAATGAACACTTTTA-TTTATATTTATAAAAATATGA------- | 944 |
| YJM1078 | 719 | AAAAATATATATATTTATCATAATATATTAAATTTTA-----TTACATTACATTACAAATAAATACTTTTATTTTATATTTATAAAAATATGA------- | 806 |
| YJM1439 | 731 | AAAAATATATATATTTATCATAATATATTAAATTTTATTACATTACATTACATTACAAATAAATACTTTTA-TTTATATTTATAAAAATATGAACTCCTT | 829 |
| consensus | 901 | \*\*\*\*.\*\*\*\*\*\*\*\*\*\*\*\*\*\*\*\*\*\*\*\*\*\*\*\*\*\*\*\*\*\*\*------\*\*\*\*\*\*-----\*\*\*\*\*\*\*.\*\*.\*\*\*\*\*\*\*-\*\*\*\*\*\*\*\*\*\*\*\*\*\*\*\*\*\*\*\*\*------- | 1000 |
|
| S288C | 909 | ---------------------------------------------------------------------------------------------------- | 908 |
| YJM1388 | 815 | ---------------------------------------------------------------------------------------------------- | 814 |
| YJM789 | 804 | ---------------------------------------------------------------------------------------------------- | 803 |
| YJM1273 | 808 | ---------------------------------------------------------------------------------------------------- | 807 |
| NCYC3585 | 805 | ---------------------------------------------------------------------------------------------------- | 804 |
| YJM1401 | 815 | -----------------------------------------------------------------------------------------ACTCCTTC--- | 822 |
| NCYC3594 | 945 | ---------------------------------------------------------------------------------------------------- | 944 |
| YJM1078 | 807 | ---------------------------------------------------------------------------------------------------- | 806 |
| YJM1439 | 830 | CTTAAATAGGGGTTCGGTCCCCCACGGGGTCCCTCACTCCTTCGGGGTTCGGTCCCCCCCTCCCGTTAGAATAGGGAGGGGGGTCCCTCACTCCTTCTTA | 929 |
| consensus | 1001 | ---------------------------------------------------------------------------------------------------- | 1100 |
|
| S288C | 909 | --------------------------------------------------------------ACTCCTTCGGGGTCCGCCCCGCGGGGGCGGGCCGGACT | 946 |
| YJM1388 | 815 | -------------------------------------------------------------------------------------------------ACT | 817 |
| YJM789 | 804 | -------------------------------------------------------------------------------------------------ACT | 806 |
| YJM1273 | 808 | -------------------------------------------------------------------------------------------------ACT | 810 |
| NCYC3585 | 805 | -------------------------------------------------------------------------------------------------ACT | 807 |
| YJM1401 | 823 | --------------GGGGTTCGGTCCCCCTCCCATTAGTATAGTATAGGGAGGGGTCCCTCACTCCTTCGGGGT---CCCCGCCGGGG---CGGGGACTC | 902 |
| NCYC3594 | 945 | -------------------------------------------------------------------------------------------------ACT | 947 |
| YJM1078 | 807 | -------------------------------------------------------------------------------------------------ACT | 809 |
| YJM1439 | 930 | ATTAAAGATAAAAAGGGGTTCGGTCCCCCTCCCGTTAGAATAAATATAGGGAGGGGTCCCTCACTCCTTCGGGGTCCGCCCCGCGGGGGCGGGCCGGACT | 1029 |
| consensus | 1101 | ---------------------------------.----.---......--..---..--......-.-..---.---.---..--------.-..-.... | 1200 |
|
| S288C | 947 | CCATATTATTATTATTATAATTATTATTATAATTATTATTATAATTATTATTATAATTATTATTATAATTAAAGAGTTTTGGATACCAATATGATATAAT | 1046 |
| YJM1388 | 818 | CCATATTATTATTATTATAATTATTATTATAATTATTATTATAATTATTATTATAATTATTATTATAATTAAAGAGTTTTGGATACCAATATGATATAAT | 917 |
| YJM789 | 807 | CCATATTATTATTATTATAATTATTATTATAATTATTATTATAATTATTATTATAATTATTATTATAATTAAAGAGTTTTGGATACCAATATGATATAAT | 906 |
| YJM1273 | 811 | CCATATTATTATTATTATAATTATTATTATAATTATTATTATAATTATTATTATAATTATTATTATAATTAAAGAGTTTTGGATACCAATATGATATAAT | 910 |
| NCYC3585 | 808 | CCATATTATTATTATTATAATTATTATTATAATTATTATTATAATTATTATTATAATTATTATTATAATTAAAGAGTTTTGGATACCAATATGATATAAT | 907 |
| YJM1401 | 903 | CATATTATTATTATTATAATTATTATTATAATTATTATTATAATTATTATTATAATTATTATTATAATTAAAGAGTTTTGGATACCAATATGATATAATA | 1002 |
| NCYC3594 | 948 | CCATATTATTATTATTATAATTATTATTATAATTATTATTATAATTATTATTATAATTATTATTATAATTAAAGAGTTTTGGATACCAATATGATATAAT | 1047 |
| YJM1078 | 810 | CCATATTATTATTATTATAATTATTATTATAATTATTATTATAATTATTATTATAATTATTATTATAATTAAAGAGTTTTGGATACCAATATGATATAAT | 909 |
| YJM1439 | 1030 | CCATATTATTATTATTATAATTATTATTATAATTATTATTATAATTATTATTATAATTATTATTATAATTAAAGAGTTTTGGATACCAATATGATATAAT | 1129 |
| consensus | 1201 | \*....\*..\*..\*..\*...\*.\*..\*..\*...\*.\*..\*..\*...\*.\*..\*..\*...\*.\*..\*..\*...\*.\*.\*\*....\*\*\*.\*....\*.\*.........\*.. | 1300 |
|
| S288C | 1047 | ATGATATAGGACCGAAACCCCTCATTTTATCATTTATTTATAATATTATAAATAAAAAAAAATATTATATATTATAATAAAATTAATATCATAATATATT | 1146 |
| YJM1388 | 918 | ATGATATAGGATCGAAACCCCTCATTTTATCATTTATTTATAATATTATAAAT-AAAAAAAATATTATATGTTATAATAAAATTAATATCATAATATATT | 1016 |
| YJM789 | 907 | ATGATATAGGATCGAAACCCCTCATTTTATCATTTATTTATAATATTATAAAT-AAAAAAAATATTATATGTTATAATAAAATTAATATCATAATATATT | 1005 |
| YJM1273 | 911 | ATGATATAGGATCGAAACCCCTCATTTTATCATTTATTTATAATATTATAAAT-AAAAAAAATATTATATGTTATAATAAAATTAATATCATAATATATT | 1009 |
| NCYC3585 | 908 | ATGATATAGGATCGAAACCCCTCATTTTATCATTTATTTATAATATTATAAAT-AAAAAAAATATTATATGTTATAATAAAATTAATATCATAATATATT | 1006 |
| YJM1401 | 1003 | TGATATAGGATCGAAACCCCTCATTTTATCATTTATTTATAATATTATAAATAAAAAAAAATATTATATGTTATAATAAAATTAATATCATAATATATTA | 1102 |
| NCYC3594 | 1048 | ATGATATAGGACCGAAACCCCTCATTTTATCATTTATTTATAATATTATAAAT-AAAAAAAATATTATATATTATAATAAAATTAATATCATAATATATT | 1146 |
| YJM1078 | 910 | ATGATATAGGACCGAAACCCCTCATTTTATCATTTATTTATAATATTATAAATAAAAAAAAATATTATATGTTATAATAAAATTAATATCATAATATATT | 1009 |
| YJM1439 | 1130 | ATGATATAGGATCGAAACCCCTCATTTTATCATTTATTTATAATATTATAAAT-AAAAAAAATATTATATGTTATAATAAAATTAATATCATAATATATT | 1228 |
| consensus | 1301 | ........\*.....\*\*.\*\*\*....\*\*\*.....\*\*..\*\*...\*...\*...\*\*..-\*\*\*\*\*\*\*...\*......\*...\*..\*\*\*.\*.\*.......\*.....\*. | 1400 |
|
| S288C | 1147 | ATATTATATATTATAT--TATATATATATATATATATATTCTTTTATAAAATTTATATTCTTCTTA---------------------TTAAAATTAAAAA | 1223 |
| YJM1388 | 1017 | ATATTATATATTATAT--TATATATATATATATATATATTCTTTTATAAAATTTATATTCTTCTTA--------------TTAAAATTTAAAATTTAAAA | 1100 |
| YJM789 | 1006 | ATATTATATATTATAT--TATATATATATATATATATATTCTTTTATAAAATTTATATTCTTCTTA--------------TTAAAATTTAAAATTAAAAA | 1089 |
| YJM1273 | 1010 | ATATTATATATTATAT--TATATATATATATATATATATTCTTTTATAAAATTTATATTCTTCTTATTAAAATTTAAAATTTAAAATTTAAAATTAAAAA | 1107 |
| NCYC3585 | 1007 | ATATTATATATTATAT--TATATATATATATATATATATTCTTTTATAAAATTTATATTCTTCTTATTAAAATTTAAAATTTAAAATTTAAAATTAAAAA | 1104 |
| YJM1401 | 1103 | TATTATATATTATATTATATATATATATATATATATATTCTTTTATAAAATTTATATTCTTCTTATTAAAATTTTAAATTTAAAATTTAAAATTAAAAA- | 1201 |
| NCYC3594 | 1147 | ATATTATATATTATAT--TATATATATATATATATATATTCTTTTATAAAATTTATATTCTTCTTA---------------------TTAAAATTAAAAA | 1223 |
| YJM1078 | 1010 | ATATTATATATTATAT--TATATATATATATATATATATTCTTTTATAAAATTTATATTCTTCTTATTAAAAT-------TTAAAATTTAAAATTAAAAA | 1100 |
| YJM1439 | 1229 | ATATTATATATTATAT--TATATATATATATATATATATTCTTTTATAAAATTTATATTCTTCTTATTAAAATTTAAAATTTAAAATTTAAAATTTAAAA | 1326 |
| consensus | 1401 | ...\*......\*....\*--....................\*..\*\*\*...\*\*\*.\*\*....\*..\*..\*..-.---.------.--.---.-\*.\*\*\*.\*..\*\*\*- | 1500 |
|
| S288C | 1224 | ---------------GGGAGCGGACTTTTAATTATATTTAATTATAGTTTTTAATCATTGGTTGAGATTTCAAAATAAGGTATAATA-TTTATATTATTC | 1307 |
| YJM1388 | 1101 | ---------------GGGAACGGACTTTTAATTATATTTAATTATAGTTTTTAATCATTGGTTGAGATTTCAAAATAAGGTATAATA-TTTATATTATTC | 1184 |
| YJM789 | 1090 | ---------------GGGAGCGGACTTTTAATTATATTTAATTATAGTTTTTAATCATTGGTTGAGATTTCAAAATAAGGTATAATATTTTATATTATTC | 1174 |
| YJM1273 | 1108 | ---------------GGGAGCGGACTTTTAATTATATTTAATTATAGTTTTTAATCATTGGTTGAGATTTCAAAATAAGGTATAATA-TTTATATTATTC | 1191 |
| NCYC3585 | 1105 | ---------------GGGAGCGGACTTTTAATTATATTTAATTATAGTTTTTAATCATTGGTTGAGATTTCAAAATAAGGTATAATA-TTTATATTATTC | 1188 |
| YJM1401 | 1202 | --------------GGGAGCGGACTTTTAATTATATTTAATTATAGTTTTTAATCATTGGTTGAGATTTCAAAATAAGGTATAATA--TTTATATTATTC | 1285 |
| NCYC3594 | 1224 | ---------------GGGAGCGGACTTTTAATTATATTTAATTATAGTTTTTAATCATTGGTTGAGATTTCAAAATAAGGTATAATA-TTTATATTATTC | 1307 |
| YJM1078 | 1101 | ---------------GGGAGCGGACTTTTAATTATATTTAATTATAGTTTTTAATCATTGGTTGAGATTTCAAAATAAGGTATAATA-TTTATATTATTC | 1184 |
| YJM1439 | 1327 | TTTAAAATTAAAAAGGGGAGCGGACTTTTAATTATATTTAATTATAGTTTTTAATCATTGGTTGAGATTTCAAAATAAGGTATAATA-TTTATATTATTC | 1425 |
| consensus | 1501 | ---------------\*\*....\*...\*\*\*.\*.\*....\*\*.\*.\*.....\*\*\*\*.\*....\*.\*.\*.....\*\*..\*\*\*..\*.\*....\*..--\*\*\*\*\*\*\*\*\*\*\*\* | 1600 |
|
| S288C | 1308 | TTTAACAAATATTATATTAT--------------AAAAAAAGATATAATATT--TATATTATTCTTTAACAAATATTATATTATAAAAAAGATATAATAT | 1391 |
| YJM1388 | 1185 | TTTAACAAATATTATATTAT---------------AAAAAAGATATAATATTTATATATTATTATT-AACATTATTTTTT-------------------- | 1248 |
| YJM789 | 1175 | TTTAACAAATATTATATTAT---------------AAAAAAGATATAATATTTATATATTATTATT-AACATTATTTTTTT------------------- | 1239 |
| YJM1273 | 1192 | TTTAACAAATATTATATTATATTAT----------AAAAAAGATATAATATTTATATATTATTATT-AATATTATTTTTT-------------------- | 1260 |
| NCYC3585 | 1189 | TTTAACAAATATTATATTATATTAT----------AAAAAAGATATAATATTTATATATTATTATT-AACATTATTTTTTTT------------------ | 1259 |
| YJM1401 | 1286 | TTTAACAAATATTATATTAT---------------AAAAAAGATATAATATTTATATATTATTATT-AACATTATTTTTT-------------------- | 1349 |
| NCYC3594 | 1308 | TTTAACAAATATTATATTATATTAT---------AAAAAAAGATATAATATTTATA--TTATTCTTTAACAAATATTATATTATAAAAAAGATATAATAT | 1396 |
| YJM1078 | 1185 | TTTAACAAATATTATATTATATTATATTATATTATAAAAAAGATATAATATTTATATATTATTATT-AACATTATTTTTTTT------------------ | 1265 |
| YJM1439 | 1426 | TTTAACAAATATTATATTAT---------------AAAAAAGATATAATATTTATATATTATTATT-AATATTATTTTTT-------------------- | 1489 |
| consensus | 1601 | \*\*\*\*\*\*\*\*\*\*\*\*\*\*\*\*\*\*\*\*--------------.\*\*\*\*\*\*\*\*\*\*\*\*\*\*\*\*\*--\*\*--\*\*\*\*\*.\*\*-\*\*.\*....\*\*.\*.-------------------- | 1700 |
|
| S288C | 1392 | TTATATATTATTATTAATATTATTTTTAAGTTCCG------------------------------------AAAGGAGAAACTTATAA-TTTTTATATCA | 1454 |
| YJM1388 | 1249 | -----------------------------------CAGTTCCGGGGCCCGGCCACGGGAGCCGGAACCCCGAAAGGAGAAACTTATAA-TTTTTATATCA | 1312 |
| YJM789 | 1240 | -----------------------------------CAGTTCCGGGGCCCGGCCACGGGAGCCGGAACCCCGAAAGGAGAAACTTATAA-TTTTTATATCA | 1303 |
| YJM1273 | 1261 | -----------------------------------CAGTTCCGGGGCCCGGCCACGGGAGCCGGAACCCCGAAAGGAGAAACTTATAA-TTTTTATATCA | 1324 |
| NCYC3585 | 1260 | -----------------------------------CAGTTCCGGGGCCCGGCCACGGGAGCCGGAACCCCGAAAGGAGAAACTTATAA-TTTTTATATCA | 1323 |
| YJM1401 | 1350 | -----------------------------------CAGTTCCGGGGCCCGGCCACGGGAGCCGGAACCCCGAAAGGAGAAACTTATAATTTTTATATCAT | 1414 |
| NCYC3594 | 1397 | TTATATATTATTATTAATATTATTTTTTAGTTCCG------------------------------------AAAGGAGAAACTTATAA-TTTTTATATCA | 1459 |
| YJM1078 | 1266 | -----------------------------------CAGTTCCGGGGCCCGGCCACGGGAGCCGGAACCCCGAAAGGAGAAACTTATAA-TTTTTATATCA | 1329 |
| YJM1439 | 1490 | ---------------------------------------------------------------------------CATAAACTTATAA-TTTTTATATCA | 1513 |
| consensus | 1701 | ---------------------------.-----------------------------------------------.\*.\*\*\*\*\*\*\*\*\*\*-\*\*\*\*....... | 1800 |
|
| S288C | 1455 | TTATTTATTATTATTTTTAATTTCAACTCCTTTTAGGTATTTCCATTTAACTTTCAGCAGAGACTTTCTAATTATAATTATATATATATAAATTTAAATA | 1554 |
| YJM1388 | 1313 | TTATTTATTATTATTTTTAATTTCAACTCCTTTTGGAGGTTTCCATTTCACCTTCAGCAGAGACTTTCTAATTATAATTATATATATATAAATTTAAATA | 1412 |
| YJM789 | 1304 | TTATTTATTATTATTTTTAATTTCAACTCCTTTTTGGGGTTTCTATTTTACCTTCAGCAGAGACTTTCTAATTATAATTATATATATATAAATTTAAATA | 1403 |
| YJM1273 | 1325 | TTATTTATTATTATTTTTAATTTCAACTCCTTTTTGGGGTTTCTATTTTACCTTCAGCAGAGACTTTCTAATTATAATTATATATATATAAATTTAAATA | 1424 |
| NCYC3585 | 1324 | TTATTTATTATTATTTTTAATTTCAACTCCTTTTGGAGGTTTCCATTTCACCTTCAGCAGAGACTTTCTAATTATAATTATATATATATAAATTTAAATA | 1423 |
| YJM1401 | 1415 | TATTTATTATTATTTTTAATTTCAACTCCTTTTGGGGGTTTCTATTTTACCTTCAGCAGAGACTTTCTAATTATAATTATATATATATAAATTTAAATAC | 1514 |
| NCYC3594 | 1460 | TTATTTATTATTATTTTTAATAATAACTCCTTTTAGGAATTTCCATTTAACCTTCAGCAGAGACTTTCTAATTATAATTATATATATATAAATTTAAATA | 1559 |
| YJM1078 | 1330 | TTATTTATTATTATTTTTAATTTCAACTCCTTTTGGGGGTTTCTATTTTACCTTCAGCAGAGACTTTCTAATTATAATTATATATATATAAATTTAAATA | 1429 |
| YJM1439 | 1514 | TTATTTATTATTATTTTTAATTTCAACTCCTTTTTGGGGTTTCTATTTTACCTTCAGCAGAGACTTTCTAATTATAATTATATATATATAAATTTAAATA | 1613 |
| consensus | 1801 | \*..\*\*..\*..\*..\*\*\*\*.\*.\*...\*...\*.\*\*\*..\*...\*\*....\*\*\*..\*.\*...........\*\*...\*.\*...\*.\*...........\*\*.\*\*.\*\*... | 1900 |
|
| S288C | 1555 | CATTTATAAAAAAGTATATAATATAATTATATTATATATAATAATATTATTAAATGAAGTATTCTTTATTATTAATTATAGGATATCTGGGGTCCATTAA | 1654 |
| YJM1388 | 1413 | CATTTATAAAAAAGTATATAATATAATTATATTATATATAATAATATTATTAAATGAAGTATTCTTTATTATTAATTATAGGATATCTGGGGTCCATTAA | 1512 |
| YJM789 | 1404 | CATTTATAAAAAAGTATATAATATAATTATATTATATATAATAATATTATTAAATGAAGTATTCTTTATTATTAATTATAGGATATCTGGGGTCCATTAA | 1503 |
| YJM1273 | 1425 | CATTTATAAAAAAGTATATAATATAATTATATTATATATAATAATATTATTAAATGAAGTATTCTTTATTATTAATTATAGGATATCTGGGGTCCATTAA | 1524 |
| NCYC3585 | 1424 | CATTTATAAAAAAGTATATAATATAATTATATTATATATAATAATATTATTAAATGAAGTATTCTTTATTATTAATTATAGGATATCTGGGGTCCATTAA | 1523 |
| YJM1401 | 1515 | ATTTATAAAAAAGTATATAATATAATTATATTATATATAATAATATTATTAAATGAAGTATTCTTTATTATTAATTATAGGATATCTGGGGTCCATTAAT | 1614 |
| NCYC3594 | 1560 | CATTTATAAAAAAGTATATAATATAATTATATTATATATAATAATATTATTAAATGAAGTATTCTTTATTATTAATTATAGGATATCTGGGGTCCATTAA | 1659 |
| YJM1078 | 1430 | CATTTATAAAAAAGTATATAATATAATTATATTATATATAATAATATTATTAAATGAAGTATTCTTTATTATTAATTATAGGATATCTGGGGTCCATTAA | 1529 |
| YJM1439 | 1614 | CATTTATAAAAAAGTATATAATATAATTATATTATATATAATAATATTATTAAATGAAGTATTCTTTATTATTAATTATAGGATATCTGGGGTCCATTAA | 1713 |
| consensus | 1901 | ..\*\*...\*\*\*\*\*.......\*....\*.\*....\*.......\*..\*...\*..\*.\*\*...\*....\*..\*\*..\*..\*.\*.\*....\*.......\*\*\*..\*..\*.\*. | 2000 |
|
| S288C | 1655 | TAATTATTATTGTAAATAATAATAAGGA-CCCCC-CCCATTATCTAATTAATAAATATATAAATAATCATTAATAAATATATTAATAATTATTAATAAAT | 1752 |
| YJM1388 | 1513 | TAATTATTATTGTAAATAATAATAGGGA-CCCCCACCCATTATCTAATTAATAAATATATAAATAATCATTAATAAATATATTAATAATTATTAATAAAT | 1611 |
| YJM789 | 1504 | TAATTATTATTGTAAATAATAATAGGGA--CCCC-CCCATTATCTAATTAATAAATATATAAATAATCATTAATAAATATATTAATAAATAATAATAAAT | 1600 |
| YJM1273 | 1525 | TAATTATTATTGTAAATAATAATAGGGA--CCCC-CCCATTATCTAATTAATAAATATATAAATAATCATTAATAAATATATTAATAAATAATAATAAAT | 1621 |
| NCYC3585 | 1524 | TAATTATTATTGTAAATAATAATAGGGACCCCCC-CCCATTATCTAATTAATAAATATATAAATAATCATTAATAAATATATTAATAATTATTAATAAAT | 1622 |
| YJM1401 | 1615 | AATTATTATTGTAAATAATAATAGGGACCCCCC-CCCATTATCTAATTAATAAATATATAAATAATCATTAATAAATATATTAATAAATAATAATAAATA | 1713 |
| NCYC3594 | 1660 | TAATTATTATTGTAAATAATAATAAGGACCCCCC-CCCATTATCTAATTAATAAATATATAAATAATCATTAATAAATATATTAATAATTATTAATAAAT | 1758 |
| YJM1078 | 1530 | TAATTATTATTGTAAATAATAATAGGGA-CCCCC-CCCATTATCTAATTAATAAATATATAAATAATCATTAATAAATATATTAATAATTATTAATAAAT | 1627 |
| YJM1439 | 1714 | TAATTATTATTGTAAATAATAATAGGGACCCCCC-CCCATTATCTAATTAATAAATATATAAATAATCATTAATAAATATATTAATAATTATTAATAAAT | 1812 |
| consensus | 2001 | .\*.\*..\*..\*...\*\*..\*..\*....\*..--\*\*\*-.\*\*..\*.....\*.\*.\*..\*\*......\*\*..\*....\*.\*..\*\*.....\*.\*..\*\*..\*..\*..\*\*.. | 2100 |
|
| S288C | 1753 | ATATAAATAATCATTAATAAATATATAAATAATATAATATATT-----ATAAAAATATAATAATAATAATTTATTATTAAAATATAATAATTTATTATAA | 1847 |
| YJM1388 | 1612 | ATATAAATAATCATTAATAAATATATAAATAATATATTATATTATATTATAAAAATATAATAATAATAATTTATTATTAAAATATAATAATTTATTATAA | 1711 |
| YJM789 | 1601 | ATATAAATAATCATTAATAAATATATAAATAATATATTATATT-----ATAAAAATATAATAATAATAATTTATTATTAAAATATAATAATTTATTATAA | 1695 |
| YJM1273 | 1622 | ATATAAATAATCATTAATAAATATATAAATAATATATTATATT-----ATAAAAATATAATAATAATAATTTATTATTAAAATATAATAATTTATTATAA | 1716 |
| NCYC3585 | 1623 | ATATAAATAATCATTAATAAATATATAAATAATATATTATATT-----ATAAAAATATAATAATAATAATTTATTATTAAAATATAATAATTTATTATAA | 1717 |
| YJM1401 | 1714 | TATAAATAATCATTAATAAATATATAAATAATATATTATATT------ATAAAAATATAATAATAATAATTTATTATTAAAATATAATAATTTATTATTA | 1807 |
| NCYC3594 | 1759 | ATATAAATAATCATTAATAAATATATAAATAATATATTATATT-----ATAAAAATATAATAATAATAATTTATTATTAAAATATAATAATTTATTATAA | 1853 |
| YJM1078 | 1628 | ATATAAATAATCATTAATAAATATATAAATAATATATTATATT-----ATAAAAATATAATAATAATAATTTATTATTAAAATATAATAATTTATTATAA | 1722 |
| YJM1439 | 1813 | ATATAAATAATCATTAATAAATATATAAATAATATATTATATT-----ATAAAAATATAATAATAATAATTTATTATTAAAATATAATAATTTATTATAA | 1907 |
| consensus | 2101 | ....\*\*..\*....\*.\*..\*\*......\*\*..\*..........\*------\*\*\*\*\*\*\*\*\*\*\*\*\*\*\*\*\*\*\*\*\*\*\*\*\*\*\*\*\*\*\*\*\*\*\*\*\*\*\*\*\*\*\*\*\*\*\*\*\*\*.\* | 2200 |
|
| S288C | 1848 | AAATATAATAATTTATTATAAAAATATAATAA---------------------TAACTCCTTTCGGGGTTCACACCTTTATAAATAAT------------ | 1914 |
| YJM1388 | 1712 | AAATATAATAATTTATTATTAAAATATAATAATTTATTATAAAAATATAATAATAACTCTTTTCGGGGTTCACACTTTTATAAATAATAAATAATAAATA | 1811 |
| YJM789 | 1696 | AAATATAATAATTTATTATTAAAATATAATAATTTATTATAAAAATATAATAATAACTCTTTTCGGGGTTCACACTTTTATAAATAATAAATAATAAATA | 1795 |
| YJM1273 | 1717 | AAATATAATAATTTATTATTAAAATATAATAATTTATTATAAAAATATAATAATAACTCTTTTCGGGGTTCACACTTTTATAAATAATAAATAATAAATA | 1816 |
| NCYC3585 | 1718 | AAATATAATAATTTATTATTAAAATATAATAATTTATTATAAAAATATAATAATAACTCTTTTTGGGGTTCACACTTTTATAAATAATAAATAATAAATA | 1817 |
| YJM1401 | 1808 | AAATATAATAATTTATTATAAAAATATAATAATTTATTATAAAAATATAATAATAACTCTTTTCGGGGTTCACACTTTTATAAATAATAAATAATAAATA | 1907 |
| NCYC3594 | 1854 | AAATATAATAATTTATTATAA---------------------AAATATAATAATAACTCCTTTCGGGGTTCACACCTTTATAAATAAT------------ | 1920 |
| YJM1078 | 1723 | AAATATAATAATTTATTATTAAAATATAATAATTTATTATAAAAATATAATAATAACTCTTTTCGGGGTTCACACTTTTATAAATAATAAATAATAAATA | 1822 |
| YJM1439 | 1908 | AAATATAATAATTTATTATTAAAATATAATAATTTATTATAAAAATATAATAATAACTCTTTTCGGGGTTCACACTTTTATAAATAATAAATAATAAATA | 2007 |
| consensus | 2201 | \*\*\*\*\*\*\*\*\*\*\*\*\*\*\*\*\*\*\*.\*--------------------------------\*\*\*\*\*\*.\*\*\*.\*\*\*\*\*\*\*\*\*\*\*.\*\*\*\*\*\*\*\*\*\*\*\*------------ | 2300 |
|
| S288C | 1915 | ------------------------------AAATAATAAATAATAAATAATAAATAATAAATATTAGTATTCACTA------------------------ | 1960 |
| YJM1388 | 1812 | ATAAATAAT---------------------AAATAATAAATAATAAATAATAAATAATAAATATTAGTGTTCACTAAT---------------------- | 1868 |
| YJM789 | 1796 | ATAAATAATAAATAATAAATAATAAATAATAAATAATAAATAATAAATAATAAATAATAAATATTAGTGTTTACTA------------------------ | 1871 |
| YJM1273 | 1817 | ATAAATAATAAATAAT--------------AAATAATAAATAATAAATAATAAATAATAAATATTAGTGTTCATTAAT---------------------- | 1880 |
| NCYC3585 | 1818 | ATAAATAATAAATAAT--------------AAATAATAAATAATAAATAATAAATAATAAATATTAGTGTTTACTA------------------------ | 1879 |
| YJM1401 | 1908 | ATAAATAATAAATAAT--------------AAATAATAAATAATAAATAATAAATAATAAATATTAGTGTTTACTA------------------------ | 1969 |
| NCYC3594 | 1921 | ------------------------------AAATAATAAATAATAAATAATAAATAATAAATATTAGTATTCACTA------------------------ | 1966 |
| YJM1078 | 1823 | ATAAATAATAAATAATAAATAATAAATAATAAATAATAAA---------------------TATTAGTGTTCACTAAT---------------------- | 1879 |
| YJM1439 | 2008 | ATAAATAAT---------------------AAATAATAAATAATAAATAATAAATAATAAATATTAGTGTTTACTCCTTCCGGGGTTCCGGCTCCCGTGG | 2086 |
| consensus | 2301 | ------------------------------\*\*\*\*\*\*\*\*\*\*---------------------\*\*\*\*\*\*\*.\*\*.\*.\*..----------------------- | 2400 |
|
| S288C | 1961 | ----------------ATATAAAATAATAATTATAAAAATAATCATTATTAAAAATATTATTAATTATTAAATTAAATACAATTAATATAATTTAGTTGT | 2044 |
| YJM1388 | 1869 | ------------AAATATATAAAATAATAATTATAAAAATAATCATTATTAAAAATATTATTAATTATTAAATTAAATACAATTAATATAATTTAGTTGT | 1956 |
| YJM789 | 1872 | ----------------ATATAAAATAATAATTATAAAAATAATCATTATTAAAAATATTATTAATTATTAAATTAAATACAATTAATATAATTTAGTTGT | 1955 |
| YJM1273 | 1881 | ------------AAATATATAAAATAATAATTATAAAAATAATCATTATTAAAAATATTATTAATTATTAAATTAAATACAATTAATATAATTTAGTTGT | 1968 |
| NCYC3585 | 1880 | ----------------ATATAAAATAATAATTATAAAAATAATCATTATTAAAAATATTATTAATTATTAAATTAAATACAATTAATATAATTTAGTTGT | 1963 |
| YJM1401 | 1970 | ----------------ATATAAAATAATAATTATAAAAATAATCATTATTAAAAATATTATTAATTATTAAATTAAATACAATTAATATAATTTAGTTGT | 2053 |
| NCYC3594 | 1967 | ----------------ATATAAAATAATAATTATAAAAATAATCATTATTAAAAATATTATTAATTATTAAATTAAATACAATTAATATAATTTAGTTGT | 2050 |
| YJM1078 | 1880 | ------------AAATATATAAAATAATAATTATAAAAATAATCATTATTAAAAATATTATTAATTATTAAATTAAATACAATTAATATAATTTAGTTGT | 1967 |
| YJM1439 | 2087 | CCGGGGCCCGGAACTAATATAAAATAATAATTATAAAAATAATCATTATTAAAAATATTATTAATTATTAAATTAAATACAATTAATATAATTTAGTTGT | 2186 |
| consensus | 2401 | -------------...\*\*\*\*\*\*\*\*\*\*\*\*\*\*\*\*\*\*\*\*\*\*\*\*\*\*\*\*\*\*\*\*\*\*\*\*\*\*\*\*\*\*\*\*\*\*\*\*\*\*\*\*\*\*\*\*\*\*\*\*\*\*\*\*\*\*\*\*\*\*\*\*\*\*\*\*\*\*\*\*\*\*\*\* | 2500 |
|
| S288C | 2045 | TTATATAATTTTAAATAATGTTTATATCAATTTAATAAAATTAAATTTAT-------------------------------------------------- | 2094 |
| YJM1388 | 1957 | TTATATAATTTTAAATAATGTTTATATCAATTTAATAAAATTAAATTTAT-------------------------------------------------- | 2006 |
| YJM789 | 1956 | TTATATAATTTTAAATAATGTTTATATCAATTTAATAAAATTAAATTTAT-------------------------------------------------- | 2005 |
| YJM1273 | 1969 | TTATATAATTTTAAATAATGTTTATATCAATTTAATAAAATTAAATTTAT-------------------------------------------------- | 2018 |
| NCYC3585 | 1964 | TTATATAATTTTAAATAATGTTTATATCAATTTAATAAAATTAAATTTAT-------------------------------------------------- | 2013 |
| YJM1401 | 2054 | TTATATAATTTTAAATAATGTTTATATCAATTTAATAAAATTAAATTTAT-------------------------------------------------- | 2103 |
| NCYC3594 | 2051 | TTATATAATTTTAAATAATGTTTATATCAATTTAATAAAATTAAATTTAT-------------------------------------------------- | 2100 |
| YJM1078 | 1968 | TTATATAATTTTAAATAATGTTTATATCAATTTAATAAAATTAAATTTAT-------------------------------------------------- | 2017 |
| YJM1439 | 2187 | TTATATAATTTTAAATAATGTTTATATCAATTTAATAAAATTAAATTTATAGTGAACACCTTTATTTAAAGGTGTGAACCAATCCCGCAAGGCAAGGGAG | 2286 |
| consensus | 2501 | \*\*\*\*\*\*\*\*\*\*\*\*\*\*\*\*\*\*\*\*\*\*\*\*\*\*\*\*\*\*\*\*\*\*\*\*\*\*\*\*\*\*\*\*\*\*\*\*\*\*-------------------------------------------------- | 2600 |
|
| S288C | 2095 | -AGTTCCGGGGCCCGGCCACGGGAGCCGGAACCCCGAAAGGAGTTTATCTATATATTATAATAACTATATGAATTTAATTATTAAAAATAATAAAAATAA | 2193 |
| YJM1388 | 2007 | -AGTTCCGGGGGCCGGCCACGGGAGCCGGAACCCCGAAAGGAGTTTATCTATATATTATAATAACTATATGAATTTCATTATTAAAAATAATAAAAATAA | 2105 |
| YJM789 | 2006 | -AGTTCCGGGGCCCGGCCACGGGAGCCGGAACCCCGAAAGGAGTTTATCTATATATTATAATAACTATATGAATTTAATTATTAAAAATAATAAAAATAA | 2104 |
| YJM1273 | 2019 | -AGTTCCGGGGCCCGGCCACGGGAGCCGGAACCCCGAAAGGAGTTTATCTATATATTATAATAACTATATGAATTTAATTATTAAAAATAATAAAAATAA | 2117 |
| NCYC3585 | 2014 | -AGTTCCGGGGCCCGGCCACGGGAGCCGGAACCCCGAAAGGAGTTTATCTATATATTATAATAACTATATGAATTTAATTATTAAAAATAATAAAAATAA | 2112 |
| YJM1401 | 2104 | -AGTTCCGGGGGCCGGCCACGGGAGCCGGAACCCCGAAAGGAGTTTATCTATATATTATAATAACTATATGAATTTCATTATTAAAAATAATAAAAATAA | 2202 |
| NCYC3594 | 2101 | -AGTTCCGGGGCCCGGCCACGGGAGCCGGAACCCCGAAAGGAGTTTATCTATATATTATAATAACTATATGAATTTCATTATTAAAAATAATAAAAATAA | 2199 |
| YJM1078 | 2018 | -AGTTCCGGGGCCCGGCCACGGGAGCCGGAACCCCGAAAGGAGTTTATCTATATATTATAATAACTATATGAATTTAATTATTAAAAATAATAAAAATAA | 2116 |
| YJM1439 | 2287 | GAGTTCCGGGGCCCGGCCACGGGAGCCGGAACCCCGAAAGGAGTTTATCTATATATTATAATAACTATATGAATTTAATTATTAAAAATAATAAAAATAA | 2386 |
| consensus | 2601 | -\*\*\*\*\*\*\*\*\*\*.\*\*\*\*\*\*\*\*\*\*\*\*\*\*\*\*\*\*\*\*\*\*\*\*\*\*\*\*\*\*\*\*\*\*\*\*\*\*\*\*\*\*\*\*\*\*\*\*\*\*\*\*\*\*\*\*\*\*\*\*\*\*\*\*.\*\*\*\*\*\*\*\*\*\*\*\*\*\*\*\*\*\*\*\*\*\*\* | 2700 |
|
| S288C | 2194 | GGAATTTTAATAAGAAGTAATATTTATTATATAATATATAAAAAAAATATATATATATATATAAAAATATATATAATAAGTTTTATTATAATATATATTA | 2293 |
| YJM1388 | 2106 | GGAATTTTAATAAGAAGTAATATTTATTATATAATATATAAAAAA-----ATATATATATATAAAAATATATATAATAAGTTTTATTATAATATATATTA | 2200 |
| YJM789 | 2105 | GGAATTTTAATAAGAAGTAATATTTATTATATAATATATAAAAAAA----ATATATATATATAAAAATATATATAATAAGTTTTATTATAATATATATTA | 2200 |
| YJM1273 | 2118 | GGAATTTTAATAAGAAGTAATATTTATTATATAATATATAAAAAAA----ATATATATATATAAAAATATATATAATAAGTTTTATTATAATATATATTA | 2213 |
| NCYC3585 | 2113 | GGAATTTTAATAAGAAGTAATATTTATTATATAATATATAAAAAAA----ATATATATATATAAAAATATATATAATAAGTTTTATTATAATATATATTA | 2208 |
| YJM1401 | 2203 | GGAATTTTAATAAGAAGTAATATTTATTATATAATATAT-AAAAAA----ATATATATATATAAAAATATATATAATAAGTTTTATTATAATATATATTA | 2297 |
| NCYC3594 | 2200 | GGAATTTTAATAAGAAGTAATATTTATTATATAATATATAAAAAAT----ATATATATATATAAAAATATATATAATAAGTTTTATTATAATATATATTA | 2295 |
| YJM1078 | 2117 | GGAATTTTAATAAGAAGTAATATTTATTATATAATATATAAAAAAT----ATATATATATATAAAAATATATATAATAAGTTTTATTATAATATATATTA | 2212 |
| YJM1439 | 2387 | GGAATTTTAATAAGAAGTAATATTTATTATATAATATATAAAAAAA----ATATATATATATAAAAATATATATAATAAGTTTTATTATAATATATATTA | 2482 |
| consensus | 2701 | \*\*\*\*\*\*\*\*\*\*\*\*\*\*\*\*\*\*\*\*\*\*\*\*\*\*\*\*\*\*\*\*\*\*\*\*\*\*\*-\*\*\*\*\*.----\*\*\*\*\*\*\*\*\*\*\*\*\*\*\*\*\*\*\*\*\*\*\*\*\*\*\*\*\*\*\*\*\*\*\*\*\*\*\*\*\*\*\*\*\*\*\*\*\*\* | 2800 |
|
| S288C | 2294 | AATTAATTATTATGAGGGGTTCGGTCCCTTTCCGGGCCCCAATTCATCTCATCTCATTTTATTTCATTTCAATATCATCTAATCTCATTTCTTTATAGAT | 2393 |
| YJM1388 | 2201 | AATTAATTATTATGAGGGGTTCGGTCCCTTTCCGGG-CCCAATTCATCTCATCTCATTTTATTTCATTTCAATATCATCTAATTTCATTTCTTTATAGAT | 2299 |
| YJM789 | 2201 | AATTAATTATTATGAGGGGTTCGGTCCCTTTCCGGGCCCCAATTCATCTCATCTCATTTTATTTCATTTCAATATCATCTAATCTCATTTCTTTATAGAT | 2300 |
| YJM1273 | 2214 | AATTAATTATTATGAGGGGTTCGGTCCCTTTCCGGGCCCCAATTCATCTCATCTCATTTTATTTCATTTCAATATCATCTAATCTCATTTCTTTATAGAT | 2313 |
| NCYC3585 | 2209 | AATTAATTATTATGAGGGGTTCGGTCCCTTTCCGGG-CCCAATTCATCTCATCTCATTTTATTTCATTTCAATATCATCTAATCTCATTTCTTTATAGAT | 2307 |
| YJM1401 | 2298 | AATTAATTATTATGAGGGGTTCGGTCCCTTTCCGGG-CCCAATTCATCTCATCTCATTTTATTTCATTTCAATATCATCTAATCTCATTTCTTTATAGAT | 2396 |
| NCYC3594 | 2296 | AATTAATTATTATGAGGGGTTCGGTTCCCTTCCGGACCCCAATTCATCTCATCTCATTTTATTTCATCTCAATATCATCTAATCTCATTTCTTTATAGAT | 2395 |
| YJM1078 | 2213 | AATTAATTATTATGAGGGGTTCGGTTCCCTTCCGGACCCCAATTCATCTCATCTCATTTTATTTCATCTCAATATCATCTAATCTCATTTCTTTATAGAT | 2312 |
| YJM1439 | 2483 | AATTAATTATTATGAGGGGTTCGGTCCCTTTCCGGGCCCCAATTCATCTCATCTCATTTTATTTCATTTCAATATCATCTAATCTCATTTCTTTATAGAT | 2582 |
| consensus | 2801 | \*\*\*\*\*\*\*\*\*\*\*\*\*\*\*\*\*\*\*\*\*\*\*\*\*.\*\*.\*\*\*\*\*\*.-\*\*\*\*\*\*\*\*\*\*\*\*\*\*\*\*\*\*\*\*\*\*\*\*\*\*\*\*\*\*.\*\*\*\*\*\*\*\*\*\*\*\*\*\*\*.\*\*\*\*\*\*\*\*\*\*\*\*\*\*\*\* | 2900 |
|
| S288C | 2394 | TTTACATATATATAAATATAAATATAAGATATTCACATTTATATATAATATAATATAATATAATAGATATTCATTCCTCTTTGATTAAAC-------TAA | 2486 |
| YJM1388 | 2300 | TTTACATATATATAAATATAAATATAAGATATTCACATTTATATATAATATAATATAATATAATAGATATTCATTCCTCTTTGATTAAAC-------TAA | 2392 |
| YJM789 | 2301 | TTTACATATATATAAATATAAATATAAGATATTCACATTTATATATAATATAATATAATATAATAGATATTCATTCCTCTTTGATTAAAC-------TAA | 2393 |
| YJM1273 | 2314 | TTTACATATATATAAATATAAATATAAGATATTCACATTTATATATAATATAATATAATATAATAGATATTCATTCCTCTTTGATTAAAC-------TAA | 2406 |
| NCYC3585 | 2308 | TTTACATATATATAAATATAAATATAAGATATTCACATTTATATATAATATAATATAATATAATAGATATTCATTCCTCTTTGATTAAAC-------TAA | 2400 |
| YJM1401 | 2397 | TTTACATATATATAAATATAAATATAAGATATTCACATTTATATATAATATAATATAATATAATAGATATTCATTCCTCTTTGATTAAAC-------TAA | 2489 |
| NCYC3594 | 2396 | TTTACATATATATAAATATAAATATAAGATATTCACATTTATATATAATATAATATAATATAATAGATATTTATTCCTTTTTGATTAAACTAATAATTAA | 2495 |
| YJM1078 | 2313 | TTTACATATATATAAATATAAATATAAGATATTCACATTTATATATAATATAATATAATATAATAGATATTTATTCCTTTTTGATTAAACTAATAATTAA | 2412 |
| YJM1439 | 2583 | TTTACATATATATAAATATAAATATAAGATATTCACATTTAT-----ATATAATATAATATAATAGATATTCATTCCTCTTTGATTAAAC-------TAA | 2670 |
| consensus | 2901 | \*\*\*\*\*\*\*\*\*\*\*\*\*\*\*\*\*\*\*\*\*\*\*\*\*\*\*\*\*\*\*\*\*\*\*\*\*\*\*\*\*\*-----\*\*\*\*\*\*\*\*\*\*\*\*\*\*\*\*\*\*\*\*\*\*\*\*.\*\*\*\*\*\*.\*\*\*\*\*\*\*\*\*\*\*-------\*\*\* | 3000 |
|
| S288C | 2487 | TAATTAATAATTAATAATTAATAATTAATAATTAATAATTATTCAGTAGAACTCCTTCTTAAAAAGGGGTTCGGTCCCCCTCCCATTAGTATAGTATAGG | 2586 |
| YJM1388 | 2393 | TAATTAATAATTAATAATTAATAATTAATAATTAATAATTATTCAGTAGA-------------------------------------------------- | 2442 |
| YJM789 | 2394 | TAATTAATAATTAATAATTAATAATTAATAATTAATAATTATTCAGTAGA-------------------------------------------------- | 2443 |
| YJM1273 | 2407 | TAATTAATAATTAATAATTAATAATTAATAATTAATAATTATTCAGTAGA-------------------------------------------------- | 2456 |
| NCYC3585 | 2401 | TAATTAATAATTAATAATTAATAATTAATAATTAATAATTATTCAGTAGA-------------------------------------------------- | 2450 |
| YJM1401 | 2490 | TAATTAATAATTAATAATTAATAATTAATAATTAATAATTATTCAGTAGA-------------------------------------------------- | 2539 |
| NCYC3594 | 2496 | TAATTAATAATTAATAATTAATAATTAATAATTAATAATTATTCGGTAGA-------------------------------------------------- | 2545 |
| YJM1078 | 2413 | TAATTAATAATTAATAATTAATAATTAATAATTAATAATTATTCGGTAGA-------------------------------------------------- | 2462 |
| YJM1439 | 2671 | TAATTAATAATTAATAATTAATAATTAATAATTAATAATTATTCAGTAGAACTCCTTCTTAAAAAGGGGTTCGGTCCCCCTCCCATTAG---------GG | 2761 |
| consensus | 3001 | \*\*\*\*\*\*\*\*\*\*\*\*\*\*\*\*\*\*\*\*\*\*\*\*\*\*\*\*\*\*\*\*\*\*\*\*\*\*\*\*\*\*\*\*.\*\*\*\*\*-------------------------------------------------- | 3100 |
|
| S288C | 2587 | GAGGGGTCCCTCACTCCTTCGGGGTCCGCCCCGCAGGGGGCGGGCCGGACTATTATTAAATAATTTATAATTTATTATTTATTAATATATTTATATAATA | 2686 |
| YJM1388 | 2443 | ------------------------------------------------ACTATTATTAAATAATTTATAATTTATTATTTATTAATATATTTATATAATA | 2494 |
| YJM789 | 2444 | ------------------------------------------------ACTATTATTAAATAATTTATAATTTATTATTTATTAATATATTTATATAATA | 2495 |
| YJM1273 | 2457 | ------------------------------------------------ACTATTATTAAATAATTTATAATTTATTATTTATTAATATATTTATATAATA | 2508 |
| NCYC3585 | 2451 | ------------------------------------------------ACTATTATTAAATAATTTATAATTTATTATTTATTAATATATTTATATAATA | 2502 |
| YJM1401 | 2540 | ------------------------------------------------ACTATTATTAAATAATTTATAATTTATTATTTATTAATATATTTATATAATA | 2591 |
| NCYC3594 | 2546 | ------------ACTCCTTCGGGGTCCGCCCCGCAGGGGGCGGGCCGGACTATTATTAAATAATTTATAATTTATTATTTATTAATATATTTATATAATA | 2633 |
| YJM1078 | 2463 | ------------ACTCCTTCGGGGTCCGCCCCGCAGGGGGCGGGCCGGACTATTATTAAATAATTTATAATTTATTATTTATTAATATATTTATATAATA | 2550 |
| YJM1439 | 2762 | AGGGGGACCCTCACTCCTTCGGGGTCCGCCCCGCAGGGGGCGGGCCGGACTATTATTAAATAATTTATAATTTATTATTTATTAATATATTTATATAATA | 2861 |
| consensus | 3101 | ..----.-----------------------------------------\*\*\*\*\*\*\*\*\*\*\*\*\*\*\*\*\*\*\*\*\*\*\*\*\*\*\*\*\*\*\*\*\*\*\*\*\*\*\*\*\*\*\*\*\*\*\*\*\*\*\*\* | 3200 |
|
| S288C | 2687 | TAATATAATATA-----ATATTATTCATACTTTTTAT-----TAATATAATATAATATAATATTATTAATACTTTCTCCTTTCGGGGTTCCGGCTCCCGT | 2776 |
| YJM1388 | 2495 | TAATATAATATA-----ATATTATTCATACTTTTTAT----------TAATATAATATAATATTATTAATACTTTCTCCTTTCGGGGTTCCGGCTCCCGT | 2579 |
| YJM789 | 2496 | TAATATAATATA-----ATATTATTCATACTTTTTAT-----T-----AATATAATATAATATTATTAATACTTTCTCCTTTCGGGGTTCCGGCTCCCGT | 2580 |
| YJM1273 | 2509 | TAATATAATATA-----ATATTATTCATACTTTTTATTAATAT-----AATATAATATAATATTATTCATACTTT------------------------- | 2573 |
| NCYC3585 | 2503 | TAATATAATATA-----ATATTATTCATACTTTTTAT---------------TAATATAATATTATTTATACTTTT------------------------ | 2558 |
| YJM1401 | 2592 | TAATATAATATA-----ATATTATTCATACTTTTTAT---------------TAATATAATATTATTTATACTTTCTCCTTTCGGGGTTCCGGCTCCCGT | 2671 |
| NCYC3594 | 2634 | TAATATAATATAATATTATATTATTCATACTTTTTAT-----TA-----ATATAATATAATATTATTCATACTTTTT----------------------- | 2700 |
| YJM1078 | 2551 | TAATATAATATAATATTATATTATTCATACTTTTTAT-----TA-----ATATAATATAATATTATTCATACTTTTT----------------------- | 2617 |
| YJM1439 | 2862 | TAATATAATATA-----ATATTATTCATACTTTTTAT----------TAATATAATATAATATTATTAATACTTTTT----------------------- | 2923 |
| consensus | 3201 | \*\*\*\*\*\*\*\*\*\*\*\*-----\*\*\*\*\*\*\*\*\*\*\*\*\*\*\*\*\*\*\*\*---------------\*\*\*\*\*\*\*\*\*\*\*\*\*\*\*.\*\*\*\*\*\*\*.------------------------ | 3300 |
|
| S288C | 2777 | GGCCGGGCCCCGGAACTATTAATATAAAGAAAAGAGTTTCAA--------TTATTTATTTATTTATTTATTTTTT----ATAAAAATAAGTCCCCGCCCC | 2864 |
| YJM1388 | 2580 | GGCCGGGCCCCGGAACTATTAATATAAAGAAAAGAGTTTCAA--------TTATTTATTTATTTATTTATTTTTT----ATAAAAATAAGTCCCCGCCCC | 2667 |
| YJM789 | 2581 | GGCCGGGCCCCGGAACTATTAATATAAAGAAAAGAGTTTCAA--------TTATTTATTTATTTATTTATTTATTTTTTATAAAAATAA----------- | 2661 |
| YJM1273 | 2574 | ---------------TTATTAATATAAAGAAAAGAGTTTCAA--------TTATTTATTTATTTATTTATTTATTTTTTATAAAAATA------------ | 2638 |
| NCYC3585 | 2559 | ----------------TATTAATATAAAGAAAAGAGTTTCAA--------TTATTTATTTATTTATTTATTTTTT----ATAAAAATA------------ | 2618 |
| YJM1401 | 2672 | GGCCGGGCCCCGGAACTATTAATATAAAGAAAAGAGTTTCAA--------TTATTTATTTATTTATTTATTTTTT----ATAAAAATAAGTCCCCGCCCC | 2759 |
| NCYC3594 | 2701 | -----------------ATTAATATAAAGAAAAGAGTTTCAATTATTTATTTATTTATTTATTTATTTATTTTTT----ATAAAAATA------------ | 2767 |
| YJM1078 | 2618 | -----------------ATTAATATAAAGAAAAGAGTTTCAATTATTTATTTATTTATTTATTTATTTATTTTTT----ATAAAAATA------------ | 2684 |
| YJM1439 | 2924 | -----------------ATTAATATAAAGAAAAGAGTTTCAA----TTATTTATTTATTTATTTATTTATTTTTT----ATAAAAATA------------ | 2986 |
| consensus | 3301 | ---------------.-\*\*\*\*\*\*\*\*\*\*\*\*\*\*\*\*\*\*\*\*\*\*\*\*\*--------\*\*\*\*\*\*\*\*\*\*\*\*\*\*\*\*\*\*\*\*\*\*.\*\*----\*\*\*\*\*\*\*\*\*------------ | 3400 |
|
| S288C | 2865 | GGCGGGGACCCCGAAGGAG---------------------------------------------------------TATTAATTTAAATAATTTATTTAA | 2907 |
| YJM1388 | 2668 | GGCGGGGACCCCGAAGGAGTGAGGGACCCCTCCCTATACTCTATACTAATGGGAGGGGGACCGAACCCCGAAGGAGTATTAATTTAAATAATTTATTTAA | 2767 |
| YJM789 | 2662 | ---------------------------------------------------------------------------------------------------- | 2661 |
| YJM1273 | 2639 | --------------------------------------------------------------------------AGAATTAAGTTAAATAATTTATTTAA | 2664 |
| NCYC3585 | 2619 | --------------------------------------------------------------------------AGAATTAATTTAAATAATTTATTTAA | 2644 |
| YJM1401 | 2760 | GGCGGGGACCCCGAAGGAGTGAGGGACCCCTCCCTATACTCTATACTAATGGGAGGGGGACCGAACCCCGAAGGAGTATTAATTTAAATAATTTATTTAA | 2859 |
| NCYC3594 | 2768 | --------------------------------------------------------------------------AGAATTAATTTAAATAATTTATTTAA | 2793 |
| YJM1078 | 2685 | --------------------------------------------------------------------------AGAATTAATTTAAATAATTTATTTAA | 2710 |
| YJM1439 | 2987 | --------------------------------------------------------------------------AGAATTAATTTAAATAATTTATTTAA | 3012 |
| consensus | 3401 | ----------------------------------------------------------------------------.-----.----------------- | 3500 |
|
| S288C | 2908 | TGAAATTATTAATTATAAATAAAAATAATAATTTTTAAAGATGTAATATAAAAATAAATATAATATAATTTAGGATAATTATATAAAATATTTATTATAT | 3007 |
| YJM1388 | 2768 | TGAAATTATTAATTATAAATAAAAATAATAATTTTTAAAGATGTAATATAAAAATAAATATAATATAATTTAGGATAATTATATAAAATATTTATTATAT | 2867 |
| YJM789 | 2662 | ---------------------------------------------------------ATATAATATAATTTAGGATAATTATATAAAATATTTATTATAT | 2704 |
| YJM1273 | 2665 | TGAAATTATTAATTATAAATAAAAATAATAATTTTTAAAGATGTAATATAAAAATAAATATAATATAATTTAGGATAATTATATAAAATATTTATTATAT | 2764 |
| NCYC3585 | 2645 | TGAAATTATTAATTATAAATAAGAATAATAATTTTTAAAGATGTAATATAAAAATAAATATAATATAATTTAGGATAATTATATAAAATATTTATTATAT | 2744 |
| YJM1401 | 2860 | TGAAATTATTAATTATAAATAAAAATAATAATTTTTAAAGATGTAATATAAAAATAAATATAATATAATTTAGGATAATTATATAAAATATTTATTATAT | 2959 |
| NCYC3594 | 2794 | TGAAATTATTAATTATAAATAAAAATAATAATTTTTAAAGATGTAATATAAAAATAAATATAATATAATTTAGGATAATTATATAAAATATTTATTATAT | 2893 |
| YJM1078 | 2711 | TGAAATTATTAATTATAAATAAAAATAATAATTTTTAAAGATGTAATATAAAAATAAATATAATATAATTTAGGATAATTATATAAAATATTTATTATAT | 2810 |
| YJM1439 | 3013 | TGAAATTATTAATTATAAATAAAAATAATAATTTTTAAAGATGTAATATAAAAATAAATATAATATAATTTAGGATAATTATATAAAATATTTATTATAT | 3112 |
| consensus | 3501 | ----------------------.----------------------------------\*\*\*\*\*\*\*\*\*\*\*\*\*\*\*\*\*\*\*\*\*\*\*\*\*\*\*\*\*\*\*\*\*\*\*\*\*\*\*\*\*\*\* | 3600 |
|
| S288C | 3008 | ATAGTTTT-TATAAAGAGTTTTAAAAGTGATAATATAATATATAATATTTATAAGTTCCGGGGCCCGGCCACGGGAGCCGGAACCCCGAAAGG------- | 3099 |
| YJM1388 | 2868 | ATAGTTTT-TATAAAGAGTTAAAAAAGTGATAATATAATATATAATATTTATAAGTTCCGGGGCCCGGCCACGGGAGCCGGAACCCCGAAAGG------- | 2959 |
| YJM789 | 2705 | ATAGTTTTCTATAAGGAGTTAAAAAAGTGATAATATAATATATAATATTTATA----------------------------------------------- | 2757 |
| YJM1273 | 2765 | ATAGTTTT-TATAAAGAGTTTTAAAAGTGATAATATAATATATAATATTTATA----------------------------------------------- | 2816 |
| NCYC3585 | 2745 | ATAGTTTT-TATAAAGAGTTTTAAAAGTGATAATATAATATATAATATTTATA----------------------------------------------- | 2796 |
| YJM1401 | 2960 | ATAGTTTT-TATAAAGAGTTAAAAAAGTGATAATATAATATATAATATTTATAAGTTCCGGGGCCCGGCCACGGGAGCCGGAACCCCGAAAGG------- | 3051 |
| NCYC3594 | 2894 | ATAGTTTT-TATAAGGAGTTTAAAAAGTGATAATATAATATATAATATTTATAA---------------------------------------------- | 2946 |
| YJM1078 | 2811 | ATAGTTTT-TATAAGGAGTTTAAAAAGTGATAATATAATATATAATATTTATAA---------------------------------------------- | 2863 |
| YJM1439 | 3113 | ATAGTTTTCTATAAAGAGTTAAAAAAGTGATAATATAATATATAATATTTATAA-TTCCGGGGCCCGGCCACGGGAGCCGGAACCCCGAAAGGAGTGAGG | 3211 |
| consensus | 3601 | \*\*\*\*\*\*\*\*-\*\*\*\*\*.\*\*\*\*\*..\*\*\*\*\*\*\*\*\*\*\*\*\*\*\*\*\*\*\*\*\*\*\*\*\*\*\*\*\*\*\*----------------------------------------------- | 3700 |
|
| S288C | 3100 | ------------------------------------AGTTATTTATATATATATAATTATAATCTTATTAATTATTTATATATATATTTAATATTA-TTT | 3162 |
| YJM1388 | 2960 | ------------------------------------AGTTATTTATATATATATAATTATAATCTTATTAATTATTTATATATATATTTAATATTA-TTT | 3022 |
| YJM789 | 2758 | ------------------------------------AGTTATTTATATATATATAATTATAATCTTATTAATTATTTATATATATATTTAATATTATTTT | 2821 |
| YJM1273 | 2817 | ------------------------------------AGTTATTTATATATATATAATTATAATCTTATTAATTATTTATATATATATTTAATATTA-TTT | 2879 |
| NCYC3585 | 2797 | ------------------------------------AGTTATTTATATATATATAATTATAATCTTATTAATTATTTATATATATATTTAATATTA-TTT | 2859 |
| YJM1401 | 3052 | ------------------------------------AGTTATTTATATATATATAATTATAATCTTATTAATTATTTATATATATATTTAATATTA-TTT | 3114 |
| NCYC3594 | 2947 | -------------------------------------GTTATTTATATATATATAATTATAATCTTATTAATTATTTATATATATATTTAATATTA-TTT | 3008 |
| YJM1078 | 2864 | -------------------------------------GTTATTTATATATATATAATTATAATCTTATTAATTATTTATATATATATTTAATATTA-TTT | 2925 |
| YJM1439 | 3212 | GACCCCCTCCCGTTAGGGAGGGGTGACCGAACCCCTCGTTATTTATATATATATAATTATAATCTTATTAATTATTTATATATATATTTAATATTATTTT | 3311 |
| consensus | 3701 | ------------------------------------.\*\*\*\*\*\*\*\*\*\*\*\*\*\*\*\*\*\*\*\*\*\*\*\*\*\*\*\*\*\*\*\*\*\*\*\*\*\*\*\*\*\*\*\*\*\*\*\*\*\*\*\*\*\*\*\*\*\*\*-\*\*\* | 3800 |
|
| S288C | 3163 | TTATATAATTTTATATTAAAGTATTATAATTATATATTTAATATTATTTTTATATAATTTTATATTATTTATTTATTTATTTATTTATTTAAAAATATTA | 3262 |
| YJM1388 | 3023 | TTATATAATTTTATATTAAAGTATTATAATTATATATTTAATATTATTTTTATATAATTTTATATTATTTATTTATTTATTTATTTATTTAAAAATATTA | 3122 |
| YJM789 | 2822 | TTATATAATTTTATATTAAAGTATTATAATTATATATTTAATATTATTTTTATATAATTTTATA----TTATTTATTTATTTATTTATTTAAAAATATTA | 2917 |
| YJM1273 | 2880 | TTATATAATTTTATATTAAAGTATTATAATTATATATTTAATATTATTTTTATATAATTTTATA----TTATTTATTTATTTATTTATTTAAAAATATTA | 2975 |
| NCYC3585 | 2860 | TTATATAATTTTATATTAAAGTATTATAATTATATATTTAATATTATTTTTATATAATTTTATATTATTTATTTATTTATTTATTTATTTAAAAATATTA | 2959 |
| YJM1401 | 3115 | TTATATAATTTTATATTAAAGTATTATAATTATATATTTAATATTATTTTTATATAATTTTATATTATTTATTTATTTATTTATTTATTTAAAAATATTA | 3214 |
| NCYC3594 | 3009 | TTATATAATTTTATATTAAAGTATTATAATTATATATTTAATATTTTTTTTATATAATTTTATAT----TATTTATTTATTTATTTATTTAAAAATATTA | 3104 |
| YJM1078 | 2926 | TTATATAATTTTATATTAAAGTATTATAATTATATATTTAATATTATTTTTATATAATTTTATAT----TATTTATTTATTTATTTATTTAAAAATATTA | 3021 |
| YJM1439 | 3312 | TTATATAATTTTATATTAAAGTATTATAATTATATATTTAATATTATTTTTATATAATTTTATAT----TATTTATTTATTTATTTATTTAAAAATATTA | 3407 |
| consensus | 3801 | \*\*\*\*\*\*\*\*\*\*\*\*\*\*\*\*\*\*\*\*\*\*\*\*\*\*\*\*\*\*\*\*\*\*\*\*\*\*\*\*\*\*\*\*\*.\*\*\*\*\*\*\*\*\*\*\*\*\*\*\*\*\*\*-----\*\*\*\*\*\*\*\*\*\*\*\*\*\*\*\*\*\*\*\*\*\*\*\*\*\*\*\*\*\*\* | 3900 |
|
| S288C | 3263 | TAATCATATATTTAATATTATTTAATATATTTTATATATTATATCTTTTATTGATTTATATATATATAGATTTAATAAATATATATATATATATATATAT | 3362 |
| YJM1388 | 3123 | TAATCATATATTTAATATTATTTAATATATTTTATATATTATATCTTTTATTGATTTATATATATATAGATTTAATAAATATATATATATATATATATAT | 3222 |
| YJM789 | 2918 | TAATCATATATTTAATATTATTTAATATATTTTATATATTATATCTTTTATTGATTTATATATATATAGATTTAATAAATATATATATATATATATATAT | 3017 |
| YJM1273 | 2976 | TAATCATATATTTAATATTATTTAATATATTTTATATATTATATCTTTTATTGATTTATATATATATAGATTTAATAAATATATATATATATATATATAT | 3075 |
| NCYC3585 | 2960 | TAATCATATATTTAATATTATTTAATATATTTTATATATTATATCTTTTATTGATTTATATATATATAGATTTAATAAATATATATATATATATATATAT | 3059 |
| YJM1401 | 3215 | TAATCATATATTTAATATTATTTAATATATTTTATATATTATATCTTTTATTGATTTATATATATATAGATTTAATAAATATATATATATATATATATAT | 3314 |
| NCYC3594 | 3105 | TAATCATATATTTAATATTATTTAATATATTTTATATATTATATCTTTTATTGATTTATATATATATAGATTTAATAAATATATATATATATATATATAT | 3204 |
| YJM1078 | 3022 | TAATCATATATTTAATATTATTTAATATATTTTATATATTATATCTTTTATTGATTTATATATATATAGATTTAATAAATATATATATATATATATATAT | 3121 |
| YJM1439 | 3408 | TAATCATATATTTAATATTATTTAATATATTTTATATATTATATCTTTTATTGATTTATATATATATAGATTTAATAAATATATATATATATATATATAT | 3507 |
| consensus | 3901 | \*\*\*\*\*\*\*\*\*\*\*\*\*\*\*\*\*\*\*\*\*\*\*\*\*\*\*\*\*\*\*\*\*\*\*\*\*\*\*\*\*\*\*\*\*\*\*\*\*\*\*\*\*\*\*\*\*\*\*\*\*\*\*\*\*\*\*\*\*\*\*\*\*\*\*\*\*\*\*\*\*\*\*\*\*\*\*\*\*\*\*\*\*\*\*\*\*\*\*\* | 4000 |
|
| S288C | 3363 | AAATATTCATTATATATTTATTATTATTATTATTATTTATTACT-------ATTTTT---TATTAT--ATATTAATAATATATATATTATTAGTTATGGG | 3450 |
| YJM1388 | 3223 | AAATATTCATTATATATTTATTATTATTATTATTATTTATTACT-------A-TTTT---TATTAT--ATATTAATAATATATATATTATTAGTTATGGG | 3309 |
| YJM789 | 3018 | AAATATTCATTATATATTTATTATTATTATTATTA-TTATTATTTATTACTATTTTT---TATTAT--ATATTAATAATATATATATTATTAGTTATGGG | 3111 |
| YJM1273 | 3076 | AAATATTCATTATATATTTATTATTATTATTATTATTTATTATTTATTATTATTTTT---ATATATTATTATTAATAATATATATATTATTAGTTATGGG | 3172 |
| NCYC3585 | 3060 | AAATATTCATTATATATTTATTATTATTATTATTATTTATTATTTATTACTA-TTTT---TATTAT--ATATTAATAATATATATATTATTAATTATGGG | 3153 |
| YJM1401 | 3315 | AAATATTCATTATATATTTATTATTATTA-------TTATTATTTATTACTA-TTTT---TATTAT--ATATTAATAATATATATATTATTAATTATGGG | 3401 |
| NCYC3594 | 3205 | AAATATTCATTATATATTTATTATTATTATTATTATTTATTATTTATTATTATTTTTATATATTAT---TATTAATAATATATATATTATTAATTATGGG | 3301 |
| YJM1078 | 3122 | AAATATTCATTATATATTTATTATTATTATTATTATTTATTATTTATTATTATTTTTAT---TATA---TATTAATAATATATATATTATTAATTATGGG | 3215 |
| YJM1439 | 3508 | AAATATTCATTATATATTTATTATTATTATTATTATTTATTACT-------ATTTTT---TATTAT--ATATTAATAATATATATATTATTAGTTATGGG | 3595 |
| consensus | 4001 | \*\*\*\*\*\*\*\*\*\*\*\*\*\*\*\*\*\*\*\*\*\*\*\*\*\*\*\*\*-------\*\*\*\*\*\*.\*-----.-\*-\*\*\*\*---......--.\*\*\*\*\*\*\*\*\*\*\*\*\*\*\*\*\*\*\*\*\*\*\*.\*\*\*\*\*\*\* | 4100 |
|
| S288C | 3451 | TATCCTAATAGTATATTATTATTTTTAATAATAATTTATGATTTATGTATAATAAATAAGTAGGGAATCGGTACGAATATCGAAAGGAGTTATATATTAT | 3550 |
| YJM1388 | 3310 | TATCCTAATAGTATATTATTATTTTTAATAATAATTTATGATTTATGTATAATAAATAAGTAGGGAATCGGTACGAATATCGAAAGGAGTTATATATTAT | 3409 |
| YJM789 | 3112 | TATCCTAATAGTATATTATTATTTTTAATAATAATTTATGATTTATGTATAATAAATAAGTAGGGAATCGGTACGAATATCGAAAGGAGTTATATATTAT | 3211 |
| YJM1273 | 3173 | TATCCTAATAGTATATTATTATTTTTAATAATAATTTATGATTTATGTATAATAAATAAGTAGGGAATCGGTACGAATATCGAAAGGAGTTATATATTAT | 3272 |
| NCYC3585 | 3154 | TATCCTAATAGTATATTATTATTTTTAATAATAATTTATGATTTATGAATAATAAATAAGTA-GGAATCGGTACGAATATCGAAAGGAGTTATATATTAT | 3252 |
| YJM1401 | 3402 | TATCCTAATAGTATATTATTATTTTTAATAATAATTTATGATTTATGAATAATAAATAAGTA-GGAATCGGTACGAATATCGAAAGGAGTTATATATTAT | 3500 |
| NCYC3594 | 3302 | TATCCTAATAGTATATTATTATTTTTAATAATAATTTATGATTTATGAATAATAAATAAGTAGGGAATCGGTATGAATATCGAAAGGAGTTATATATTAT | 3401 |
| YJM1078 | 3216 | TATCCTAATAGTATATTATTATTTTTAATAATAATTTATGATTTATGTATAATAAATAAGTAGGGAATCGGTACGAATATCGAAAGGAGTTATATATTAT | 3315 |
| YJM1439 | 3596 | TATCCTAATAGTATATTATTATTTTTAATAATAATTTATGATTTATGTATAATAAATAAGTAGGGAATCGGTACGAATATCGAAAGGAGTTATATATTAT | 3695 |
| consensus | 4101 | \*\*\*\*\*\*\*\*\*\*\*\*\*\*\*\*\*\*\*\*\*\*\*\*\*\*\*\*\*\*\*\*\*\*\*\*\*\*\*\*\*\*\*\*\*\*\*.\*\*\*\*\*\*\*\*\*\*\*\*\*\*-\*\*\*\*\*\*\*\*\*\*.\*\*\*\*\*\*\*\*\*\*\*\*\*\*\*\*\*\*\*\*\*\*\*\*\*\* | 4200 |
|
| S288C | 3551 | TAATTATTTATAATTATTTTATATATTATTAATTATTTATAATTATTTTATATATTTATAATTATTTTATATAGATAGGTTAGATAGGATAGATAGTATA | 3650 |
| YJM1388 | 3410 | TAATTATTTATAATTATTTTATATATTATTAATTATTTATAATTATTTTATATATTTATAATTATTTTATATAGATAGGATAGATAGGATAGATAGTATA | 3509 |
| YJM789 | 3212 | TAATTATTTATAATTATTTTATATATTATTAATTATTTATAATTATTTTATATATTTATAATTATTTTATATAGATAGGATAGATAGGATAGATAGTATA | 3311 |
| YJM1273 | 3273 | TAATTATTTATAATTATTTTATATATTATTAATTATTTATAATTATTTTATATATTTATAATTATTTTATATAGATAGGATAGATAGGATAGATAGTATA | 3372 |
| NCYC3585 | 3253 | TAATTATTTATAATTATTTTATATATTATTAATTATTTATAATTATTTTATATATTTATAATTATTTTATATAGATAGGATAGATAGGATAGATAGTATA | 3352 |
| YJM1401 | 3501 | TAATTATTTATAATTATTTTATATATTATTAATTATTTATAATTATTTTATATATTTATAATTATTTTATATAGATAGGATAGATAGGATAGATAGTATA | 3600 |
| NCYC3594 | 3402 | TAATTATTTATAATTATTTTATATATTATTAATTATTTATAATTATTTTATATATTTATAATTATTTTATATAGATAGGATAGATAGGATAGATAGGATA | 3501 |
| YJM1078 | 3316 | TAATTATTTATAATTATTTTATATATTATTAATTATTTATAATTATTTTATATATTTATAATTATTTTATATAGATAGGATAGATAGGATAGATAGTATA | 3415 |
| YJM1439 | 3696 | TAATTATTTATAATTATTTTATATATTATTAATTATTTATAATTATTTTATATATTTATAATTATTTTATATAGATAGGATAGATAGAATAGATAGTATA | 3795 |
| consensus | 4201 | \*\*\*\*\*\*\*\*\*\*\*\*\*\*\*\*\*\*\*\*\*\*\*\*\*\*\*\*\*\*\*\*\*\*\*\*\*\*\*\*\*\*\*\*\*\*\*\*\*\*\*\*\*\*\*\*\*\*\*\*\*\*\*\*\*\*\*\*\*\*\*\*\*\*\*\*\*\*\*.\*\*\*\*\*\*\*.\*\*\*\*\*\*\*\*.\*\*\* | 4300 |
|
| S288C | 3651 | GATA---------------------------------------------GGGGTCCCATTTATTATTTACAATAATAATTATTAATGGGACCCGGATATC | 3705 |
| YJM1388 | 3510 | GATA---------------------------------------------GGGGTCCCATTTATTATTTACAATAATAATTATTAATGGGACCCGGATATC | 3564 |
| YJM789 | 3312 | GATA---------------------------------------------GGGGTCCCATTTATTATTGAAAATAATAATTATTAATGGGACCCGGATATC | 3366 |
| YJM1273 | 3373 | TATA---------------------------------------------GGGGTCCCATTTATTATTTACAATAATAATTATTAATGGGACCCGGATATC | 3427 |
| NCYC3585 | 3353 | GATA---------------------------------------------GGGGTCCCATTTATTATTTACAATAATAATTATTAATGGGACCCGGATATC | 3407 |
| YJM1401 | 3601 | GATA---------------------------------------------GGGGTCCCATTTATTATTTACAATAATAATTATTAATGGGACCCGGATATC | 3655 |
| NCYC3594 | 3502 | GATAGGATAGATAGGATAGATAGGATAGATAGGATAGATAGGATAGATAGGGGTCCCATTTATTATTTACAATAATAATTATTAATGGGACCCGGATATC | 3601 |
| YJM1078 | 3416 | GATA---------------------------------------------GGGGTCCCATTTATTATTTACAATAATAATTATTAATGGGACCCGGATATC | 3470 |
| YJM1439 | 3796 | GATA---------------------------------------------GGGGTCCCATTTATTATTTACAATAATAATTATTAATGGGACCCGGATATC | 3850 |
| consensus | 4301 | .\*\*\*---------------------------------------------\*\*\*\*\*\*\*\*\*\*\*\*\*\*\*\*\*\*.\*.\*\*\*\*\*\*\*\*\*\*\*\*\*\*\*\*\*\*\*\*\*\*\*\*\*\*\*\*\*\* | 4400 |
|
| S288C | 3706 | TTATTGTTATTAATTTATATATTATTCATTATTATTAATATATATTTAATATAATTAAATATTATATTATATTATATTATATTATTTATTAAAAAAAAAT | 3805 |
| YJM1388 | 3565 | TTATTGTTATTAATTTATATATTATTCATTATTATTAATATATATTTAATATAATTAA-----ATATTATATTATATTATATTATTTATTAAAAAAAAAT | 3659 |
| YJM789 | 3367 | TTATTGTTATTAATTTATATATTATTCATTATTATTAATATATATTTAATATAATTAA----------ATATTATATTATATTATTTATT-AAAAAAAAT | 3455 |
| YJM1273 | 3428 | TTATTGTTATTAATTTATATATTATTCATTATTATTAATATATATTTAATATAATTAA-----ATATTATATTATATTATATTATTTATT-AAAAAAAAT | 3521 |
| NCYC3585 | 3408 | TTATTGTTATTAATTTATATATTATTCATTATTATTAATATATATTTAATATAATTAA----------ATATTATATTATATTATTTATT-AAAAAAAAT | 3496 |
| YJM1401 | 3656 | TTATTGTTATTAATTTATATATTATTCATTATTATTAATATATATTTAATATAATTAA-----ATATTATATTATATTACATTATTTATTAAAAAAAAAT | 3750 |
| NCYC3594 | 3602 | TTATTGTTATTAATTTATATATTATTCATTATTATTAATATATATTTAATATAATTAAA-----TATTATATTATATTATATTATTTATTAAAAAAAAAT | 3696 |
| YJM1078 | 3471 | TTATTGTTATTAATTTATATATTATTCATTATTATTAATATATATTTAATATAATTAAA-----TATTATATTATATTATATTATTTATT-AAAAAAAAT | 3564 |
| YJM1439 | 3851 | TTATTGTTATTAATTTATATATTATTCATTATTATTAATATATATTTAATATAATTAA-----ATATTATATTATATTATATTATTTATT-AAAAAAAAT | 3944 |
| consensus | 4401 | \*\*\*\*\*\*\*\*\*\*\*\*\*\*\*\*\*\*\*\*\*\*\*\*\*\*\*\*\*\*\*\*\*\*\*\*\*\*\*\*\*\*\*\*\*\*\*\*\*\*\*\*\*\*\*\*\*\*----------\*\*\*\*\*\*\*\*\*\*\*.\*\*\*\*\*\*\*\*\*\*-\*\*\*\*\*\*\*\*\* | 4500 |
|
| S288C | 3806 | CTATTACTTATTTTTTTTATTAATATATAAATTATTTATATAATTTATCATTTTTATTT----------------------------------------- | 3864 |
| YJM1388 | 3660 | CTATTACTTATTTTTTTTATTAATATATAAATTATTTATATAATTTATCATTTTTATTT----------------------------------------- | 3718 |
| YJM789 | 3456 | CTATTACTTATTTTTTTTATTAATATATAAATTATTTATATAATTTATCATTTTTATTT----------------------------------------- | 3514 |
| YJM1273 | 3522 | CTATTACTTATTTTTTTTATTAATATATAAATTATTTATATAATTTATCATTTTTATTT----------------------------------------- | 3580 |
| NCYC3585 | 3497 | CTATTACTTATTTTTTTTATTAATATATAAATTATTTATATAATTTATCATTTTTATTT----------------------------------------- | 3555 |
| YJM1401 | 3751 | CTATTACTTA-TTTTTTTATTAATATATAAATTATTTATATAATTTATCATTTTTATTT----------------------------------------- | 3808 |
| NCYC3594 | 3697 | CTATTACTTATTTTTTTTATTAATATATAAATTATTTATATAATTTATCATTTTTATTT----------------------------------------- | 3755 |
| YJM1078 | 3565 | CTATTACTTA-TTTTTTTATTAATATATAAATTATTTATATAATTTATCATTTTTATTT----------------------------------------- | 3622 |
| YJM1439 | 3945 | CTATTACTTA-TTTTTTTATTAATATATAAATTATTTATATAATTTATCATTTTTATTTCTCCTTTCGGGGTTCCGGCTCCCGTGGCCGGGCCCCGGAAC | 4043 |
| consensus | 4501 | \*\*\*\*\*\*\*\*\*\*-\*\*\*\*\*\*\*\*\*\*\*\*\*\*\*\*\*\*\*\*\*\*\*\*\*\*\*\*\*\*\*\*\*\*\*\*\*\*\*\*\*\*\*\*\*\*\*\*----------------------------------------- | 4600 |
|
| S288C | 3865 | -----------------------------------------------ATATATTATTATTTTTTATATATAAATTAATATATATATAT----ATTATATA | 3913 |
| YJM1388 | 3719 | -----------------------------------------------ATATATTATTATTTTTTATATATAAATTAATATATATATAT----ATTATATA | 3767 |
| YJM789 | 3515 | -----------------------------------------------ATATATTATTATTTTTTATATATAAATTAATATATATATAT----TATATATA | 3563 |
| YJM1273 | 3581 | -----------------------------------------------ATATATTATTATTTTTTATATATAAATTAATATATATATAT----TATATATA | 3629 |
| NCYC3585 | 3556 | -----------------------------------------------ATATATTATTATTTTTTATATATAAATTAATATATATATAT----TATATATA | 3604 |
| YJM1401 | 3809 | -----------------------------------------------ATATATTATTATTTTTTATATATAAATTAATATATATATAT----ATTATATA | 3857 |
| NCYC3594 | 3756 | -----------------------------------------------ATATATTATTATTTTTTATATATAAATTAATATATATATAT----ATTATATA | 3804 |
| YJM1078 | 3623 | -----------------------------------------------ATATATTATTATTTTTTATATATAAATTAATATATATATATTATATATATATA | 3675 |
| YJM1439 | 4044 | TCCTCCCTTGCGGGGTTCACACCTTTATAATTAAATAAAGGTGTTCACTATATTATTATTTTTTATATATAAATTAATATATATATAT----TATATATA | 4139 |
| consensus | 4601 | -----------------------------------------------.\*\*\*\*\*\*\*\*\*\*\*\*\*\*\*\*\*\*\*\*\*\*\*\*\*\*\*\*\*\*\*\*\*\*\*\*\*\*\*\*----..\*\*\*\*\*\* | 4700 |
|
| S288C | 3914 | TA-----C-TTTTTTTTTTATAATATATCTATATATATAAATAAA-----TATATTATATTATATTTTTATATAATATATTATTAATTATTATTTTAATT | 4002 |
| YJM1388 | 3768 | TA-----C-TTTTTTTTTTATAATATATCTATATATATAAATTAA-----TATATTATATTATATTTTTATATAATATATTATTAATTATTATTTTAATT | 3856 |
| YJM789 | 3564 | TATATACTTTTTTTTTTTTATAATATATCTATATATATAAATTAA-----TATATTATA-----TTTTTATATAATATATTATTAATTATTATTTTAATT | 3653 |
| YJM1273 | 3630 | TATATAC--TTTTTTTTTTATAATATATCTATATATATAAATTAATATATTATATTATA-----TTTTTATATAATATATTATTAATTATTATTTTAATT | 3722 |
| NCYC3585 | 3605 | TATATAC--TTTTTTTTTAATAATATATCTATATATATAAATTAA-----TATATTATA-----TTTTTATATAATATATTATTAATTATTATTTTAATT | 3692 |
| YJM1401 | 3858 | TAC------TTTTTTTTTTATAATATATCTATATATATAAATTAATATATTATATTATA-----TTTTTATATAATATATTATTAATTATTATTTTAATT | 3946 |
| NCYC3594 | 3805 | TACT-----TTTTTTTTTAATAATATATCTATATATATAAATTAA-----TATATTATAT-----TTTTATATAATATATTATTAATTATTATTTTAATT | 3889 |
| YJM1078 | 3676 | TACT-----TTTTTTTTTAATAATATATCTATATATATAAATTAA-----TATATTATAT-----TTTTATATAATATATTATTAATTATTATTTTAATT | 3760 |
| YJM1439 | 4140 | TATATAC--TTTTTTTTTAATAATATATCTATATATATAAATTAA-----TATATTATATTATATTTTTATATAATATATTATTAATTATTATTTTAATT | 4232 |
| consensus | 4701 | \*\*..---.-\*\*\*\*\*\*\*\*\*.\*\*\*\*\*\*\*\*\*\*\*\*\*\*\*\*\*\*\*\*\*\*\*.\*\*-----\*\*\*\*\*\*\*\*\*------\*\*\*\*\*\*\*\*\*\*\*\*\*\*\*\*\*\*\*\*\*\*\*\*\*\*\*\*\*\*\*\*\*\*\* | 4800 |
|
| S288C | 4003 | TTCTATTCTATTGT---GGGGGTCCCAATTATTATTTTCAATAATAATTATTATTGGGACCCGGATATCTTCTTGTTTATCATTTATTATTTTATTAAAT | 4099 |
| YJM1388 | 3857 | TTCTATTCTATTGT---GGGGGTCCCAATTATTATTTTCAATAATAATTATTATTGGGACCCGGATATCTTCTTGTTTATCATTTATTATTTTATTAAAT | 3953 |
| YJM789 | 3654 | TTCTATTCTATTGT---GGGGGTCCCAATTATTATTTTCAATAATAATTATTATTGGGACCCGGATATCTTCTTGTTTATCATTTATTATTTTATTAAAT | 3750 |
| YJM1273 | 3723 | TTCTATTCTATTGT---GGGGGTCCCAATTATTATTTTCAATAATAATTATTATTGGGACCCGGATATCTTCTTGTTTATCATTTATTATTTTATTAAAT | 3819 |
| NCYC3585 | 3693 | TTCTATTCTATTGT---GGGGGTCCCAATTATTATTTTCAATAATAATTATTATTGGGACCCGGATATCTTCTTGTTTATCATTTATTATTTTATTTAAT | 3789 |
| YJM1401 | 3947 | TTCTATTCTATTGT---GGGGGTCCCAATTATTATTTTCAATAATAATTATTATTGGGACCCGGATATCTTCTTGTTTATCATTTATTATTTTATTAAAT | 4043 |
| NCYC3594 | 3890 | TTCTATTCTATTGTGGGGGGGGTCCCAATTATTATTTTCAATAATAATTATTATTGGGACCCGGATATCTTCTTGTTTATCATTTATTATTTTATTAAAT | 3989 |
| YJM1078 | 3761 | TTCTATTCTATTGT---GGGGGTCCCAATTATTATTTTCAATAATAATTATTATTGGGACCCGGATATCTTCTTGTTTATCATTTATTATTTTATTAAAT | 3857 |
| YJM1439 | 4233 | TTCTATTCTATTGT---GGGGGTCCCAATTATTATTTTCAATAATAATTATTATTGGGACCCGGATATCTTCTTGTTTATCATTTATTATTTTATTAAAT | 4329 |
| consensus | 4801 | \*\*\*\*\*\*\*\*\*\*\*\*\*\*---\*\*\*\*\*\*\*\*\*\*\*\*\*\*\*\*\*\*\*\*\*\*\*\*\*\*\*\*\*\*\*\*\*\*\*\*\*\*\*\*\*\*\*\*\*\*\*\*\*\*\*\*\*\*\*\*\*\*\*\*\*\*\*\*\*\*\*\*\*\*\*\*\*\*\*\*\*\*\*.\*\*\* | 4900 |
|
| S288C | 4100 | TTATTATTATTTTTAATTTATATTTATATTATATAATTAATTATATCGTTTATACTCCTTC--------------------------------------- | 4160 |
| YJM1388 | 3954 | TTATTATTATTTTTAATTTATATTTATATTATATCATTAATTATATCGTTTAT----------------------------------------------- | 4006 |
| YJM789 | 3751 | TTATTATTATTTTTAATTTATATTTATATTATATCATTAATTATATCGTTTAT----------------------------------------------- | 3803 |
| YJM1273 | 3820 | TTATTATTATTTTTAATTTATATTTATATTATATAATTAATTATATCGTTTAT----------------------------------------------- | 3872 |
| NCYC3585 | 3790 | TTATTATTATTTTTAATTTATATTTATATTATATAATTAATTATATCGTTTAT----------------------------------------------- | 3842 |
| YJM1401 | 4044 | TTATTATTATTTTTAATTTATATTTATATTATATAATTAATTATATCGTTTATACTCCTTC---------------GGGGTTCGGTCCCCCTCCCATTAG | 4128 |
| NCYC3594 | 3990 | TTATTATTATTTTTAATTTATATTTATATTATATAATTAATTATATGGTTTATACTCCTTTTTTTTTTAATAAAAAGGGGTTCGGTCCCCCTCCC----- | 4084 |
| YJM1078 | 3858 | TTATTATTATTTTTAATTTATATTTATATTATATAATTAATTATATCGTT-------------------------------------------------- | 3907 |
| YJM1439 | 4330 | TTATTATTATTTTTAATTTATATTTATATTATATAATTAATTATATCGTTTAT----------------------------------------------- | 4382 |
| consensus | 4901 | \*\*\*\*\*\*\*\*\*\*\*\*\*\*\*\*\*\*\*\*\*\*\*\*\*\*\*\*\*\*\*\*\*\*.\*\*\*\*\*\*\*\*\*\*\*.\*\*\*----------.--------------------------------------- | 5000 |
|
| S288C | 4161 | -------------------------------GGGGTCCCCGCCGGGGCGGGG-ACTTTATATTTTATTATATAATATATTATATTCTTATAATATATTTA | 4228 |
| YJM1388 | 4007 | -----------------------------------------------------ACTTTATATTTTATTATATAATATATTATATTATTATAATATATTTA | 4053 |
| YJM789 | 3804 | -----------------------------------------------------ACTTTATATTTTATTATATAATATATTATATTCTTATAATATATTTA | 3850 |
| YJM1273 | 3873 | -----------------------------------------------------ACTTTATATTTTATTATATAATATATTATATTCTTATAATATATTTA | 3919 |
| NCYC3585 | 3843 | -----------------------------------------------------ACTTTATATTTTATTATATAATATATTATATTCTTATAATATATTTA | 3889 |
| YJM1401 | 4129 | TATAGTATAGGGAGGGGTCCCTCACTCCTTCGGGGTCCCCGCCGGGGCGGGG-ACTTTATATTTTATTATATAATATATTATATTCTTATAATATATTTA | 4227 |
| NCYC3594 | 4085 | -----TAACGGGAG----------------GGGGACCCTCACTCCTTCGGGGGACTTTATATTTTATTATATAATATATTATATTCTTATAATATATTTA | 4163 |
| YJM1078 | 3908 | ---------------------------------------------------ATACTTTATATTTTATTATATAATATATTATATTCTTATAATATATTTA | 3956 |
| YJM1439 | 4383 | -----------------------------------------------------ACCTTAT-TATTATTATATAATATATTATATTATTATAATATATTTA | 4428 |
| consensus | 5001 | -------..---------------------.---..--.-.-.....----..\*\*.\*\*\*\*-\*.\*\*\*\*\*\*\*\*\*\*\*\*\*\*\*\*\*\*\*\*\*\*.\*\*\*\*\*\*\*\*\*\*\*\*\*\* | 5100 |
|
| S288C | 4229 | TTGATTATGTTATAAAATTTATTCTATGTGTGCTCTATATATATTTAATATTCTGGTTATT-ATCACCCACCCCCTCCCCCTATTACGTCTCCGAGGTCC | 4327 |
| YJM1388 | 4054 | TTGATTATATTATAAAATTTATTCTATGTGTGCTCTATATATATTTAATATTCTGGTTATT-ATCACCCACCCCCTCCCCCTATTACGTCTCCGAGGTCC | 4152 |
| YJM789 | 3851 | TTGATTATATTATAAAATTTATTCTATGTGTGCTCTATATATATTTAATATTCTGGTTATT-ATCACCCACCCCCTCCCCCTATT--------------- | 3934 |
| YJM1273 | 3920 | TTGATTATATTATAAAATTTATTCTATGTGTGCTCTATATATATTTAATATTCTGGTTATT-ATCACCCACCCCCTCCCCCTATT--------------- | 4003 |
| NCYC3585 | 3890 | TTGATTATATTATAAAATTTATTCTATGTGTGCTCTATATATATTTAATATTCTGGTTATT-ATCACCCACCCCCTCCCCCTATT--------------- | 3973 |
| YJM1401 | 4228 | TTGATTATATTATAAAATTTATTCTATGTGTGCTCTATATATATTTAATATTCTGGTTATT-ATCACCCACCCCCTCCCCCTATTACGTCTCCGAGGTCC | 4326 |
| NCYC3594 | 4164 | TTGATTATATTATAAAATTTATTTTATGTGTGCTCTATATATATTTAATATTCTGGTTATT-ATCAACCA-------CCCCTATTACGTCTCCGAGGTCC | 4255 |
| YJM1078 | 3957 | TTGATTATGTTATAAAATTTATTCTATGTGTGCTCTATATATATTTAATATTCTGGTTATT-ATCAACCA-------CCCCTATTACGTCTCCGAGGTCC | 4048 |
| YJM1439 | 4429 | TTGATTATATTATAAAATTTATTCTATGTGTGTTCTATATATATTTAATATTCTGGTTATTGATCACCCACCCCCTCCCCCTATAAAACTT--------- | 4519 |
| consensus | 5101 | \*\*\*\*\*\*\*\*.\*\*\*\*\*\*\*\*\*\*\*\*\*\*.\*\*\*\*\*\*\*\*.\*\*\*\*\*\*\*\*\*\*\*\*\*\*\*\*\*\*\*\*\*\*\*\*\*\*\*\*-\*\*\*\*.\*\*\*-------\*\*\*\*\*\*\*.-....---------- | 5200 |
|
| S288C | 4328 | CGGTTTCGTAAGAAACCGGGACTTATATATTT-ATAAATATAAATCTAACTTAATTAATAATTTAAATAATATACTTTATATTTTATAAATAAAAATAAT | 4426 |
| YJM1388 | 4153 | CGGTTTCGTAAGAAACCGGGACTTATATATTT-ATAAATATAAATCTAACTTAATTAATAATTTAAATAATATACTTTATATTTTATAAATAAAAATAAT | 4251 |
| YJM789 | 3935 | --------------------ACTTATATATTT-ATAAATATAAATCTAACTTAATTAATAATTTAAATAATATACTTAATATTTTATAAATAAAAATAAT | 4013 |
| YJM1273 | 4004 | --------------------ACTTATATATTT-ATAAATATAAATCTAACTTAATTAATAATTTAAATAATATACTTTATATTTTATAAATAAAAATAAT | 4082 |
| NCYC3585 | 3974 | --------------------ACTTATATATTT-ATAAATATAAATCTAACTTAATTAATAATTTAAATAATATACTTAATATTTTATAAATAAAAATAAT | 4052 |
| YJM1401 | 4327 | CGGTTTCGTAAGAAACCGGGACTTATATATTTGATAAATATAAATCTAACTTAATTAATAATTTAAATAATATACTTTATATTTTATAAATATAAATAAT | 4426 |
| NCYC3594 | 4256 | CGGTTTCGTAAGAAACCGGGACTTATATATTT-ATAAATATAAATCTAACTTAATTAATAATTTAAATAATATACTTTATATTTTATAAATAAAAATAAT | 4354 |
| YJM1078 | 4049 | CGGTTTCGTAAGAAACCGGGACTTATATATTT-ATAAATATAAATCTAACTTAATTAATAATTTAAATAATATACTTTATATTTTATAAATAAAAATAAT | 4147 |
| YJM1439 | 4520 | ------------AATTTATTACTTATATATTT-ATAAATATAAATCTAACTTAATTAATAATTTAAATAATATACTTAATATTTAATAAATAAAAATAAT | 4606 |
| consensus | 5201 | --------------......\*\*\*\*\*\*\*\*\*\*\*\*-\*\*\*\*\*\*\*\*\*\*\*\*\*\*\*\*\*\*\*\*\*\*\*\*\*\*\*\*\*\*\*\*\*\*\*\*\*\*\*\*\*\*\*\*.\*\*\*\*\*\*.\*\*\*\*\*\*\*.\*\*\*\*\*\*\* | 5300 |
|
| S288C | 4427 | TATAA-CCTTTTTTATAATTATATATAATAATAATATATATTATCAAATAATTATTATTTCTTTTTTTTCT-------------------TTAAT----- | 4501 |
| YJM1388 | 4252 | TATAATCTTTTTTTATAATTATATATAATAATAATATATATTATCAAATAATTATTATTTCTTTTTTTTCTTTAATTAATAATTAATTAATTAAT----- | 4346 |
| YJM789 | 4014 | TATAA-TCTTTTTTATAATTATATATAATAATAATATATATTATCAAATAATTATTATTTCTTTTTTTTCT-----------------------T----- | 4084 |
| YJM1273 | 4083 | TATAA-TCTTTTTTATAATTATATATAATAATAATATATATTATCAAATAATTATTATTTCTTTTTTTTCT-----------------------TTAATT | 4158 |
| NCYC3585 | 4053 | TATAA-TCTTTTTTATAATTATATATAATAATAATATATATTATCAAATAATTATTATTTCTTTTTTTTCTT---TAATTAA---------TAAT----- | 4134 |
| YJM1401 | 4427 | TATAA-TCTTTTTTATAATTATATATAATAATAATATATATTATCAAATAATTATTATTTCTTTTTTTTTCTT--TAATTAA---------TAAT----- | 4509 |
| NCYC3594 | 4355 | TATAA-TCTTTTTTATAATTATATATAATAATAATATATATTATCAAATAATTATTATTTCTTTTTTTTTCT---TTAATTAATAATT----AAT----- | 4441 |
| YJM1078 | 4148 | TATAA-TCTTTTTTATAATTATATATAATAATAATATATATTATCAAATAATTATTATTTCTTTTTTTTTCT---TTAATTAATAATT----AAT----- | 4234 |
| YJM1439 | 4607 | TATAA-CCTTTTTTATAATTATATATAATAATAATATATATTATCAAATAATTATTATTTCTTTTTTTTCT----TTAATTAATAATT----AAT----- | 4692 |
| consensus | 5301 | \*\*\*\*\*-..\*\*\*\*\*\*\*\*\*\*\*\*\*\*\*\*\*\*\*\*\*\*\*\*\*\*\*\*\*\*\*\*\*\*\*\*\*\*\*\*\*\*\*\*\*\*\*\*\*\*\*\*\*\*\*\*\*\*\*\*\*..-----.-.-.-.-----------\*----- | 5400 |
|
| S288C | 4502 | --TAATTAATTAATTAATATTTTAT------AAAAATATATTTCTCCTTACGGGGTTCCGGCTCCCGTAGCCGGGGCCCGAAACTAAATAAAATATATTA | 4593 |
| YJM1388 | 4347 | --TAATTAATTAATTAATATTTTAT-----AAAAAATATATTT----------------------------------------CTAAATAAAATATATTA | 4399 |
| YJM789 | 4085 | --TAATTAATTAATTAATATTTTAT------AAAAATATATTT----------------------------------------ATAAATAAAATATATTA | 4136 |
| YJM1273 | 4159 | AATAATTAATTAATTAATATTTTAT-----AAAAAATATATTT----------------------------------------CTAAATAAAATATATTA | 4213 |
| NCYC3585 | 4135 | --TAATTAATTAATTAATATTTTAT-----AAAAAATATATTT----------------------------------------CTAAATAAAATATATTA | 4187 |
| YJM1401 | 4510 | --TAATTAATTAATTAATATTTTAT-----AAAAAATATATTT----------------------------------------CTAAATAAAATATATTA | 4562 |
| NCYC3594 | 4442 | --TAATTAATTAATTAATATTTTAT-----AAAAAATATATTTCTCCTTACGGGGTTCCGGCTCCCGTAGCCGGGGCCCGAAACTAAATAAAATATATTA | 4534 |
| YJM1078 | 4235 | --TAATTAATTAATTAATATTTTAT-----AAAAAATATATTTCTCCTTACGGGGTTCCGGCTCCCGTAGCCGGGGCCCGAAACTAAATAAAATATATTA | 4327 |
| YJM1439 | 4693 | --TAATTAATTAATTAATATTTTATTTTATAAAAAATATATTTCTCCTTACGGGGTTCCGGCTCCCGTAGCGGGGGCCCGAAACTAAATAAAATATATTA | 4790 |
| consensus | 5401 | --\*\*\*\*\*\*\*\*\*\*\*\*\*\*\*\*\*\*\*\*\*\*\*------\*\*\*\*\*\*\*\*\*\*\*\*----------------------------.-----------.\*\*\*\*\*\*\*\*\*\*\*\*\*\*\*\* | 5500 |
|
| S288C | 4594 | TTAATAATATTATATAATATAATAATAATATAATAATTTTATATAAATATATATTTATATATTAAATTAAATTATAATTTTATTATGAAAATTATATCTT | 4693 |
| YJM1388 | 4400 | TTAATAATATTATATAATATAATAATAATATAATAATTTTATATAAATATATATTTATATATTAAATTAAATTATAATTTTATTATGAAAATTATATCTT | 4499 |
| YJM789 | 4137 | TTAATAATATTATATAATATAATAATAATATAATAATTTTATATAAATATATATTTATATATTAAATTAAATTATAATTTTATTATGAAAATTATATCTT | 4236 |
| YJM1273 | 4214 | TTAATAATATTATATAATATAATAATAATATAATAATTTTATATAAATATATATTTATATATTAAATTAAATTATAATTTTATTATGAAAATTATATCTT | 4313 |
| NCYC3585 | 4188 | TTAATAATATTATATAATATAATAATAATATAATAATTTTATATAAATATATATTTATATATTAAATTAAATTATAATTTTATTATGAAAATTATATCTT | 4287 |
| YJM1401 | 4563 | TTAATAATATTATATAATATAATAATAATATAATAATTTTATATAAATATATATTTATATATTAAATTAAATTATAATTTTATTATGAAAATTATATCTT | 4662 |
| NCYC3594 | 4535 | TTAATAATATTATATAATATAATAATAATATAATAATTTTATATAAATATATATTTATATATTAAATTAAATTATAATTTTATTATGAAAATTATATCTT | 4634 |
| YJM1078 | 4328 | TTAATAATATTATATAATATAATAATAATATAATAATTTTATATAAATATATATTTATATATTAAATTAAATTATAATTTTATTATGAAAATTATATCTT | 4427 |
| YJM1439 | 4791 | TTAATAATATTATATAATATAATAATAATATAATAATTTTATATAAATATATATTTATATATTAAATTAAATTATAATTTTATTATGAAAATTATATCTT | 4890 |
| consensus | 5501 | \*\*\*\*\*\*\*\*\*\*\*\*\*\*\*\*\*\*\*\*\*\*\*\*\*\*\*\*\*\*\*\*\*\*\*\*\*\*\*\*\*\*\*\*\*\*\*\*\*\*\*\*\*\*\*\*\*\*\*\*\*\*\*\*\*\*\*\*\*\*\*\*\*\*\*\*\*\*\*\*\*\*\*\*\*\*\*\*\*\*\*\*\*\*\*\*\*\*\*\* | 5600 |
|
| S288C | 4694 | TTTTT-TATA------------------------------------------------------------------------------------TTTTTA | 4708 |
| YJM1388 | 4500 | TTTTT---AT------------------------------------------------------------------------------------ATTCTA | 4512 |
| YJM789 | 4237 | TTTTTTTATA------------------------------------------------------------------------------------TTTTTA | 4252 |
| YJM1273 | 4314 | TTTATATATA------------------------------------------------------------------------------------TTTTTA | 4329 |
| NCYC3585 | 4288 | TTTTTATA---------------------------------------------------------------------------------------TTCTA | 4300 |
| YJM1401 | 4663 | TTTTTATA---------------------------------------------------------------------------------------TTCTA | 4675 |
| NCYC3594 | 4635 | TTTTTATAT---------------------------------------------------------------------------------------TCTA | 4647 |
| YJM1078 | 4428 | TTTTTATAT---------------------------------------------------------------------------------------TCTA | 4440 |
| YJM1439 | 4891 | TTATTATATTCTCCTTTCGGGGTTCCGGCTCCCGTGGCCGGGCCCCGGAACTCCTCCCTTGCCTTGCGGGGTTCACACCTTTAAATAAAGGTGTTCACTA | 4990 |
| consensus | 5601 | \*\*..\*.--..------------------------------------------------------------------------------------....\*\* | 5700 |
|
| S288C | 4709 | TATAATAAAAATATGTTATATATATATTAATAAT-AAAAGGTAGTGAGGATTAAATAAATTATATAATAATTATAACTCTTAATTATAAAATAA--ATAT | 4805 |
| YJM1388 | 4513 | TATAATAAAAATATGTTATATATATATTAATAATAAAAAAGTAGTGAGGATTAAATAAATTATATAATAATTATAACTCTTAATTATAAAATAA--ATAT | 4610 |
| YJM789 | 4253 | TATAATAAAAATATGTTATATATATATTAATAAT-AAAAGGTAGTGAGGATTAAATAAATTATATAATAATTATAACTCTTAATTATAAAATAA--ATAT | 4349 |
| YJM1273 | 4330 | TATAATAAAAATATGTTATATATATATTAATAAT-AAAAGGTAGTGAGGATTAAATAAATTATATAATAATTATAACTCTTAATTATAAAATAA--ATAT | 4426 |
| NCYC3585 | 4301 | TATAATAAAAATATGTTATATATATATTAATAATAAAAAAATAGTGAGGATTAAATAAATTGTATAATAATTATAACTCTTAATTATAAAATAAATATAT | 4400 |
| YJM1401 | 4676 | TATAATAAAAATATGTTATATATATATTAATAAT-AAAAAGTAGTGAGGATTAAATAAATTATATAATAATTATAACTCTTAATTATAAAATAA--ATAT | 4772 |
| NCYC3594 | 4648 | TATAATAAAAATATGTTATATATATATTAATAATAAAAAAGTAGTGAGGATTAAATAAATTGTATAATAATTATAACTCTTAATTATAAAATAA--ATAT | 4745 |
| YJM1078 | 4441 | TATAATAAAAATATGTTATATATATATTAATAATAAAAAAGTAGTGAGGATTAAATAAATTGTATAATAATTATAACTCTTAATTATAAAATAA--ATAT | 4538 |
| YJM1439 | 4991 | TATAATAAAAATATGTTATATATATATTAATAATAAAAAAGTAGTGAGGATTAAATAAATTATATAATAATTATAACTCTTAATTATAAAATAA--ATAT | 5088 |
| consensus | 5701 | \*\*\*\*\*\*\*\*\*\*\*\*\*\*\*\*\*\*\*\*\*\*\*\*\*\*\*\*\*\*\*\*\*\*-\*\*\*\*..\*\*\*\*\*\*\*\*\*\*\*\*\*\*\*\*\*\*\*\*.\*\*\*\*\*\*\*\*\*\*\*\*\*\*\*\*\*\*\*\*\*\*\*\*\*\*\*\*\*\*\*\*--\*\*\*\* | 5800 |
|
| S288C | 4806 | ATATATATATATAAGTATCCATTTCCATATAATCTTTTAATAAATA-TTAATAAATATTAAAAAAAAATAATATTATAATA-TTTTAGTATATAATTCAA | 4903 |
| YJM1388 | 4611 | ATATATATATATAAGTATTCATTTCCATAAAATCTTTTAATAAATA-TTAATAAATATT--AAAAAAATAATATTATAATA-TTTTTGTATATAATTCAA | 4706 |
| YJM789 | 4350 | ATATATATATATAAGTATCCATTTTCATAAAATCTTTTAATAAATA-TTAATAAATATT--AAAAAAATAATATTATAATA-TTTTTGTATATAATTCAA | 4445 |
| YJM1273 | 4427 | ATATATATATATAAGTATCCATTTCCATAAAATCTTTTAATAAATA-TTAATAAATATT--AAAAAAATAATATTATAATA-TTTTTGTATATAATTCAA | 4522 |
| NCYC3585 | 4401 | ATATATATATATAAGTATCCATTTCTATAAAATCTTTTAATAAATA-TTAATAAATATT--AAAAAAATAATATTATAATA-TTTTAGTATATAATTCAA | 4496 |
| YJM1401 | 4773 | ATATATATATATAAGTATCCATTTCTATAAAATCTTTTAATAAATA-TTAATAAATATT--AAAAAAATAATATTATAATATTTTTTGTATATAATTCAA | 4869 |
| NCYC3594 | 4746 | ATATATATATATAAGTATCCATTTCTATAAAATCTTTTAATAAATATTTAATAAATATT--AAAAAAATAATATTATAATA-TTTTTGTATATAATTCAA | 4842 |
| YJM1078 | 4539 | ATATATATATATAAGTATCCATTTCTATAAAATCTTTTAATAAATATTTAATAAATATT--AAAAAAATAATATTATAATA-TTTTTGTATATAATTCAA | 4635 |
| YJM1439 | 5089 | ATATATATATATAAGTATTCATTTCCATAAAATCTTTTAATAAATA-TTAATAAATATA--AAAAAAATAATATTATAATA-TTTTTGTATATAATTCAA | 5184 |
| consensus | 5801 | \*\*\*\*\*\*\*\*\*\*\*\*\*\*\*\*\*\*.\*\*\*\*\*..\*\*\*.\*\*\*\*\*\*\*\*\*\*\*\*\*\*\*\*-\*\*\*\*\*\*\*\*\*\*\*.--\*\*\*\*\*\*\*\*\*\*\*\*\*\*\*\*\*\*\*\*-\*\*\*\*.\*\*\*\*\*\*\*\*\*\*\*\*\* | 5900 |
|
| S288C | 4904 | TAAAATTCATTGGAGGGGTAAATAATAATAATTTACTAATGGCAAGTTATAGTCTTAAAGGTTTTTA----------TTTT-TTTTATTAAATTAATAAA | 4992 |
| YJM1388 | 4707 | TAAAATTCATTGGAGGGGTAAATAATAATAATTTACTAATGGCAAGTTATAGTCTTAAAGGTTTTTA----------TTTT-TTTTATTAAATTAATAAA | 4795 |
| YJM789 | 4446 | TAAAATTCATTGGAGGGGTAAATAATAATAATTTACTAATGGCAAATTATAGTCTTAAAGGTTTTTA---------------TTTTATTAAATTAATAAA | 4530 |
| YJM1273 | 4523 | TAAAATTCATTGGAGGGGTAAATAATAATAATTTACTAATGGCAAATTATAGTCTTAAAGGTTTTTA---------------TTTTATTAAATTAATAAA | 4607 |
| NCYC3585 | 4497 | TAAAATTCATTGGAGGGGTAAATAATAATAATTTACTAATGGCAAGTTATAGTCTTAAAGGTTTTTA----------TTTTTTTTTATTAAATTAATAAA | 4586 |
| YJM1401 | 4870 | TAAAATTCATTGGAGGGGTAAATAATAATAATTTACTAATGGCAAGTTATAGTCTTAAAGGTTTTTA------------TTTTTTTATTAAATTAAAAAA | 4957 |
| NCYC3594 | 4843 | TAAAATTCATTGGAGGGGTAAATAATAATAATTTACTAATGGCAAGTTATAGTCTTAAAGGTTTTTA----------TTTTTTTTTATTAAATTAATAAA | 4932 |
| YJM1078 | 4636 | TAAAATTCATTGGAGGGGTAAATAATAATAATTTACTAATGGCAAGTTATAGTCTTAAAGGTTTTTAT-----TTTTTTTTTTTTTATTAAATTAATAAA | 4730 |
| YJM1439 | 5185 | TAAAATTCATTGGAGGGGTAAATAATAATAATTTACTAATGGCAAGTTATAGTCTTAAAGGTTTTTATTTTTTTTTATTTTTTTTTATTAAATAAATAAA | 5284 |
| consensus | 5901 | \*\*\*\*\*\*\*\*\*\*\*\*\*\*\*\*\*\*\*\*\*\*\*\*\*\*\*\*\*\*\*\*\*\*\*\*\*\*\*\*\*\*\*\*\*.\*\*\*\*\*\*\*\*\*\*\*\*\*\*\*\*\*\*\*\*\*---------.-----\*\*\*\*\*\*\*\*\*\*\*.\*\*.\*\*\* | 6000 |
|
| S288C | 4993 | ATAATAATACCATTTATATATTCCATTATATATATATATTTAATAAAAATAATAATATCATTTATATATTTTATTATATATTATATATATTTTATATAAA | 5092 |
| YJM1388 | 4796 | ATAATAATACCATTTATATATTCCATTATATATATATATTTAATAAAAATAATAATATCATTTATATATTTTATTATATATTATATATATTTTATATAAA | 4895 |
| YJM789 | 4531 | ATAATAATACCATTTATATATTCCATTATATATATATATTTAATAAAAATAATAATATTATTTATATATTTTATTATATATTATATATATTTTATATAAA | 4630 |
| YJM1273 | 4608 | ATAATAATACCATTTATATATTCCATTATATATATATATTTAATAAAAATAATAATATTATTTATATATTTTATTATATATTATATATATTTTATATAAA | 4707 |
| NCYC3585 | 4587 | ATAATAATACCATTTATATATTCCATTATATATATATATTTAATAAAAATAATAATATCATTTATATATTTTATTATATATTATATATATTTTATATAAA | 4686 |
| YJM1401 | 4958 | ATAATAATACCATTTATATATTCCATTATATATATATATTTAATAAAAATAATAATATCATTTATATATTTTATTATATATTATATATATTTTATATAAA | 5057 |
| NCYC3594 | 4933 | ATAATAATACCATTTATATATTCCATTATATATATATATTTAATAAAAATAATAATATCATTTATATATTTTATTATATATTATATATATTTTATATAAA | 5032 |
| YJM1078 | 4731 | ATAATAATACCATTTATATATTCCATTATATATATATATTTAATAAAAATAATAATATCATTTATATATTTTATTATATATTATATATATTTTATATAAA | 4830 |
| YJM1439 | 5285 | ATAATAATACCATTTATATATTCCAT--TATATATATATTTAATAAAAATAATAATATCATTTATATATTTTATTATATATTATATATATTTTATATAAA | 5382 |
| consensus | 6001 | \*\*\*\*\*\*\*\*\*\*\*\*\*\*\*\*\*\*\*\*\*\*\*\*\*\*--\*\*\*\*\*\*\*\*\*\*\*\*\*\*\*\*\*\*\*\*\*\*\*\*\*\*\*\*\*\*.\*\*\*\*\*\*\*\*\*\*\*\*\*\*\*\*\*\*\*\*\*\*\*\*\*\*\*\*\*\*\*\*\*\*\*\*\*\*\*\*\* | 6100 |
|
| S288C | 5093 | ATAATAATAATAAATTTATATTTTTATATATTA-TTATTAAAT---AATAATAATATAAATAACTCCTTC--------------GGGGTTCGGTCCCCA- | 5173 |
| YJM1388 | 4896 | ATAATAATAATAAATTTATATTTTTATATATTA-TTATTAAAT---AATAATAATATAAATAACTCCTTCTTTAAATAAAAAGGGGGGTTCGGTTCCCA- | 4990 |
| YJM789 | 4631 | ATAATAATAATAAATTTATATTTTTATATATTA-TTATTAAAT---AATAATAATATAAATA-------------------------------------- | 4688 |
| YJM1273 | 4708 | ATAATAATAATAAATTTATATTTTTATATATTA-TTATTAAAT---AATAATAATATAAATA-------------------------------------- | 4765 |
| NCYC3585 | 4687 | ATAATAATAATAAATTTATATTTTTATATATTA-TTATTAAAT---AATAATAATATAAATAACTCCTTC--------------GGGGTTCGGTCCCCA- | 4767 |
| YJM1401 | 5058 | ATAATAATAATAAATTTATATTTTTATATATTATTTATTAAAT---AATAATAATATAAATA-------------------------------------- | 5116 |
| NCYC3594 | 5033 | ATAATAATAATAAATTTATATTTTTATATATTA-TTATTAAAT---AATAATAATATAAATAACTCCTTC--------------GGGGTTCGGTCCCCCT | 5114 |
| YJM1078 | 4831 | ATAATAATAATAAATTTATATTTTTATATATTA-TTATTAAAT---AATAATAATATAAATAACTCCTTC--------------GGGGTTCGGTCCCCCT | 4912 |
| YJM1439 | 5383 | ATAATAATAATAAATTTATATTTTTATATATTA-TTATTAAATAATAATAATAATATAAATAATTCCTTC------------------------------ | 5451 |
| consensus | 6101 | \*\*\*\*\*\*\*\*\*\*\*\*\*\*\*\*\*\*\*\*\*\*\*\*\*\*\*\*\*\*\*\*\*-\*\*\*\*\*\*\*\*\*---\*\*\*\*\*\*\*\*\*\*\*\*\*\*\*\*-.------------------------------.---.- | 6200 |
|
| S288C | 5174 | ----------------------C-GGGTCCCTCACTCCTTC----TTAAGAATAAAAAGGGGTTCGGT----CCCCCTCCCGTTAGTACACGGGA-GGGG | 5241 |
| YJM1388 | 4991 | ----------------------CGGGGTCCCGCACTCCTTT------------------GGGTTCGGT----CCCCCTCCCGTTAGTACACGGGAGGGGG | 5046 |
| YJM789 | 4689 | ---------------------------------ACTCCTTC----TTAAGAATAAAAAGGGGTTCGGT----CCCCCTCCCGTTAGTACACGGGA--GGG | 4745 |
| YJM1273 | 4766 | ---------------------------------ACTCCTTC----TTAAGAATAAAAAGGGGTTCGGT----CCCCCTCCCGTTAGTACACGGGA--GGG | 4822 |
| NCYC3585 | 4768 | ----------------------C-GGGTCCCTCACTCCTTC----TTAAGAATAAAAAGGGGTTCGGT----CCCCCTCCCGTTAGTACACGGGA-GGGG | 4835 |
| YJM1401 | 5117 | ---------------------------------ATTCCTTC-----------------GGGGTTCGGTCCCCCCCCCTCCCGTTAGTACACGGGA-GGGG | 5165 |
| NCYC3594 | 5115 | CCCATTAGTATAAGTATAGGGAG-GGGTCCCTCACTCCTTCTTAATTAAGAATAAAAAGGGGTTCGGT----CCCCCTCCCGTAAGTACACGGGA-GAGG | 5208 |
| YJM1078 | 4913 | CCCATTAGTATAAGTATAGGGAG-GGGTCCCTCACTCCTTCTTAATTAAGAATAAAAAGGGGTTCGGT----CCCCCTCCCGTTAGTACACGGGA-GAGG | 5006 |
| YJM1439 | 5452 | ----------------------------------------------------------GGGGTTCGGT---CCCCCCTCCCGTTAGTACACGGGA-GGGG | 5489 |
| consensus | 6201 | ----------------------.--------.--.-----.------------------\*\*\*\*\*\*\*\*\*----\*\*\*\*\*\*\*\*\*\*\*.\*\*\*\*\*\*\*\*\*\*\*--.\*\* | 6300 |
|
| S288C | 5242 | GTCTCTCACTCCTTCTTAAAAAATAAAAAGGTGGAAGGACTAATATAATTTTAAATAATAATTAATACTTTAATAATAATTTGTATTTCTTTATTATTAA | 5341 |
| YJM1388 | 5047 | GTCTCTCACTCCTTCTTAAAAAATAAAAAGGTGGAAGGACTAATATAATTTTAAATAATAATTAATACTTTAATAATAATTTGTATTTCTTTATTATTAA | 5146 |
| YJM789 | 4746 | GTCTCTCACTCCTTCTTAAAAAATAAAAAGGTGGAAGGACTAATATAATTTTAAATAATAATTAATACTTTAATAATAATTTGTATTTCTTTATTATTAA | 4845 |
| YJM1273 | 4823 | GTCTCTCACTCCTTCTAAAAAAATAAAAAGGTGGAAGGACTAATATAATTTTAAATAATAATTAATACTTTAATAATAATTTGTATTTCTTTATTATTAA | 4922 |
| NCYC3585 | 4836 | GTCTCTCACTCCTTCTTAAAAAATAAAAAGGTGGAAGGACTAATATAATTTTTAATAATAATTAATACTTTAATAATAATTTGTATTTCTTTATTATTAA | 4935 |
| YJM1401 | 5166 | GTCTCTCACTCCTTCTTAAAAAATAAAAAGGTGGAAGGACTAATATAATTTTAAATAATAATTAATACTTTAATAATAATTTGTATTTCTTTATTATTAA | 5265 |
| NCYC3594 | 5209 | GTCTCTCACTCCTTCTTAAAAAATAAAAAGGTGGAAGGACTAATATAATTTTAAATAATAATTAATACTTTAATAATAATTTGTATTTCTTTATTATTAA | 5308 |
| YJM1078 | 5007 | GTCTCTCACTCCTTCTTAAAAAATAAAAAGGTGGAAGGACTAATATAATTTTAAATAATAATTAATACTTTAATAATAATTTGTATTTCTTTATTATTAA | 5106 |
| YJM1439 | 5490 | GTCTCTCACTCCTTCTAAAAAAATAAAAAGGTGGGAGGACTAATATAATTTTAAATAATAATTAATACTTTAATAATAATTTGTATTTCTTTATTATTAA | 5589 |
| consensus | 6301 | \*\*\*\*\*\*\*\*\*\*\*\*\*\*\*\*.\*\*\*\*\*\*\*\*\*\*\*\*\*\*\*\*\*.\*\*\*\*\*\*\*\*\*\*\*\*\*\*\*\*\*.\*\*\*\*\*\*\*\*\*\*\*\*\*\*\*\*\*\*\*\*\*\*\*\*\*\*\*\*\*\*\*\*\*\*\*\*\*\*\*\*\*\*\*\*\*\*\* | 6400 |
|
| S288C | 5342 | TATATTAAATATAATAATAATTAATATAATTACAATATATTAATATTATCAAATATTAATAAATATACTTTTTTATATAATTTATTTATTTATTTATT-- | 5439 |
| YJM1388 | 5147 | TATATTAAATATAATAATAATTAATATAATTACAATATATTAATATTATCAAATATTAATAAATATACTTTTTTATATAATTTATTTATTTATTTA-T-- | 5243 |
| YJM789 | 4846 | TATATTAAATATAATAATAATTAATATAATTACAATATATTAATATTATCAAATATTAATAAATATACTTTTTTATATAATTTATTTATTTATTTA---- | 4941 |
| YJM1273 | 4923 | TATATTAAATATAATAATAATTAATATAATTACAATATATTAATATTATCAAATATTAATAAATATACTTTTTTATATAATTTATTTATTTATTTA---- | 5018 |
| NCYC3585 | 4936 | TATATTAAATATAATAATAATTAATATAATTACAATATATTAATATTATCAAATATTAATAAATATACTTTTTTATATAATTTATTTATTTATTTAT--- | 5032 |
| YJM1401 | 5266 | TATATTAAATATAATAATAATTAATATAATTACAATATATTAATATTATCAAATATTAATAAATATA--TTTTTATATAATTTATTTATTTATTT----- | 5358 |
| NCYC3594 | 5309 | TATATTAAATATAATAATAATTAATATAATTACAATATATTAATATTATCAAATATTAATAAATATAT-TTTTTATATAATTTATTTATTTATTTATTTA | 5407 |
| YJM1078 | 5107 | TATATTAAATATAATAATAATTAATATAATTACAATATATTAATATTATCAAATATTAATAAATATAT-TTTTTATATAATTTATTTATTTATTTATTTA | 5205 |
| YJM1439 | 5590 | TATATTAAATATAATAATAATTAATATAATTACAATATATTAATATTATCAAATATTAATAAATATA--TTTTTATATAATTTATTTATTTATTTTTTTT | 5687 |
| consensus | 6401 | \*\*\*\*\*\*\*\*\*\*\*\*\*\*\*\*\*\*\*\*\*\*\*\*\*\*\*\*\*\*\*\*\*\*\*\*\*\*\*\*\*\*\*\*\*\*\*\*\*\*\*\*\*\*\*\*\*\*\*\*\*\*\*\*\*\*\*.-\*\*\*\*\*\*\*\*\*\*\*\*\*\*\*\*\*\*\*\*\*\*\*\*\*\*.---. | 6500 |
|
| S288C | 5440 | -TTTTTTTTATTAAACTAATTA------TAATTGTAATTTCGAAAA------GGGGGTGGGAGTAAACATATATAATTTATAATC--TATATATATATAT | 5524 |
| YJM1388 | 5244 | -TTTTTTTTATTAAACTAATTA------TAATTGTAATTTCGAAAA------GGGGGTGGGAGTAAACATATATAATTTATAATC--TATATATATATAT | 5328 |
| YJM789 | 4942 | -TTTTTTTTATTAAACTAATTATAATTGTAATTGTAATTTCGAAAA--GG--GGGGGTGGGAGTAAATATATATAATTTATAATC--TATATATATATAT | 5034 |
| YJM1273 | 5019 | -TTTTTTTTATTAAACTAATTATAATTGTAATTGTAATTTCGAAAA---G--GGGGGTGGGAGTAAATATATATAATTTATAATC--TATATATATATAT | 5110 |
| NCYC3585 | 5033 | -TTTTTTTTATTAAACTAATTA------TAATTGTAATTTCGAAAA------GGGGGTGGGAGTAAACATATATAATTTATAATC--TATATATATATAT | 5117 |
| YJM1401 | 5359 | -TTTTTATTATTAAACTAATTA------TAATTGTAATTTCGTAAA------GGGGGTGGGAGTAAATATATATAATTTATAATC----TATATATATAT | 5441 |
| NCYC3594 | 5408 | TTTTTTTTTATTAAACTAATTATAATTGTAATTGTAATTTCGAAAA--GGGGGGGGGTGGGAGTAAATATATATAATTTATAATCTATATATATATATAT | 5505 |
| YJM1078 | 5206 | TTTTTTTTTATTAAACTAATTATAATTGTAATTGTAATTTCGAAAAGGGGGGGGGGGTGGTAGTAAATATATATAATTTATAATC--TATATATATATAT | 5303 |
| YJM1439 | 5688 | -TTTTTATTATTAAACTAATTA------TAATTGTAATTTCGTAAA--GGGGGGGGGTGGGAGTAAATATATATAATTTATAATC----TATATATATAT | 5774 |
| consensus | 6501 | -\*\*\*\*\*.\*\*\*\*\*\*\*\*\*\*\*\*\*\*\*------\*\*\*\*\*\*\*\*\*\*\*\*\*\*.\*\*\*------\*\*\*\*\*\*\*\*.\*\*\*\*\*\*.\*\*\*\*\*\*\*\*\*\*\*\*\*\*\*\*\*----\*\*\*\*\*\*\*\*\*\*\* | 6600 |
|
| S288C | 5525 | ATATAATTTTTTAATAAATATTAATAAATATTTAT----------------------------------AAAAAGAATAATTTATATTTATAATATATAA | 5590 |
| YJM1388 | 5329 | ATATAATTTCTTAATAAATATTAATAAATATTTCT---------------------------------AAAAAAGAATAATTTATATTTATAATATATAA | 5395 |
| YJM789 | 5035 | ATATAATTTCTTAATAAATATTAATAAATATTTAT----------------------------------AAAAAGAATAATTTATATTTATAATATATAA | 5100 |
| YJM1273 | 5111 | ATATAATTTCTTAATAAATATTAATAAATATTTAT----------------------------------AAAAAGAATAATTTATATTTATAATATATAA | 5176 |
| NCYC3585 | 5118 | ATATAATTTCTTAATAAATATTAATAAATATTTAT----------------------------------AAAAAGAATAATTTATATTTATAATATATAA | 5183 |
| YJM1401 | 5442 | ATATAATTTCTTAATAAATATTAATAAATATTTCT---------------------------------AAAAAAGAATAATTTATATTTATAATATATAA | 5508 |
| NCYC3594 | 5506 | ATATAATTTCTTAATAAATATTAATAAATATTTAT----------------------------------AAAAAGAATAATTTATATTTATAATATATAA | 5571 |
| YJM1078 | 5304 | ATATAATTTCTTAATAAATATTAATAAATATTTAT----------------------------------AAAAAGAATAATTTATATTTATAATATATAA | 5369 |
| YJM1439 | 5775 | ATATAATTTCTTAATAAATATTAATAAATATTTCTCCTTCGGCGTCCGCCCCGCGGGCGGGCCGGACTAAAAAAGAATAATTTATATTTATAATATATAA | 5874 |
| consensus | 6601 | \*\*\*\*\*\*\*\*\*.\*\*\*\*\*\*\*\*\*\*\*\*\*\*\*\*\*\*\*\*\*\*\*.\*----------------------------------\*\*\*\*\*\*\*\*\*\*\*\*\*\*\*\*\*\*\*\*\*\*\*\*\*\*\*\*\*\*\* | 6700 |
|
| S288C | 5591 | TTTATATATTTTATTTTTATTATACAATTAATATAAAATATAAAATATTAAATATTAAATATTAAATA-------TTAAATATTAAATATTAATTTTTAT | 5683 |
| YJM1388 | 5396 | TTTATATATTTTATTTTTATTATACAATTAATATAAAATATAAAATATAAAATATTAAATATTAAATA-------TTAAATATTAAATATTAATTTTTAT | 5488 |
| YJM789 | 5101 | TTTATATATTTTATTTTTATTATACAATTAATATAAAATATA--------------AAATATAAAATA-------TTAAATATTAAATATTAATTTTTAT | 5179 |
| YJM1273 | 5177 | TTTATATATTTTATTTTTATTATACAATTAATATAAAATATA--------------AAATATAAAATATTAAATATTAAATATTAAATATTAATTTTTAT | 5262 |
| NCYC3585 | 5184 | TTTATATATTTTATTTTTATTATACAATTAATATAAAATATAAAATATTAAATATTAAATATTAAATA-------TTAAATATTAAATATTAATTTTTAT | 5276 |
| YJM1401 | 5509 | TTTATATATTTTATTTTTATTATACAATTAATATAAAATATAAAATATAAAATATTAAATATTAAATA-------TTAAATATTAAATATTAATTTTTAT | 5601 |
| NCYC3594 | 5572 | TTTATATATTTTATTTTTATTATACAATTAATATAAAATATAAAATATAAAATATTAAATATTAAATA-------TTAAATATTAAATATTTATTTTTAT | 5664 |
| YJM1078 | 5370 | TTTATATATTTTATTTTTATTATACAATTAATATAAAATATAAAATATAAAATATTAAATATTAAATA-------TTAAATATTA-------ATTTTTAT | 5455 |
| YJM1439 | 5875 | TTTATATATTTTATTTTTATTATACAATTAATATAAAATATAAAATATAAAATATTAAATATTAAATA---------------------TTAATTTTTAT | 5953 |
| consensus | 6701 | \*\*\*\*\*\*\*\*\*\*\*\*\*\*\*\*\*\*\*\*\*\*\*\*\*\*\*\*\*\*\*\*\*\*\*\*\*\*\*\*\*\*------.-------\*\*\*\*\*\*.\*\*\*\*\*-----------------------.\*\*\*\*\*\*\*\* | 6800 |
|
| S288C | 5684 | AGGGGTTATATAATAATTATATTTATAATTATATAATATTAAAAAGGGTATTTTTATAATTATTACATTTTTATTTTATTTATAAAAATATTAATTTTAA | 5783 |
| YJM1388 | 5489 | AGGGGTTATATAATAATTATATTTATAATTATATAATATTAAAAA-GGTATTTTTATAATTATTACATTTTTATTTTATTTATAAAAATATTAATTTTAA | 5587 |
| YJM789 | 5180 | AGGGGTTATATAATAATTATATTTATAATTATATAATATTAAAAAGGGTATTTTTATAATTATTACATTTTTATTTTATTTATAAAAATATTAATTTTAA | 5279 |
| YJM1273 | 5263 | AGGGGTTATATAATAATTATATTTATAATTATATAATATTAAAAAGGGTATTTTTATAATTATTACATTTTTATTTTATTTATAAAAATATTAATTTTAA | 5362 |
| NCYC3585 | 5277 | AGGGGTTATATAATAATTATATTTATAATTATATAATATTAAAAA-GGTATTTTTATAATTATTACATTTTTATTTTATTTATAAAAATATTAATTTTAA | 5375 |
| YJM1401 | 5602 | AGGGGTTATATAATAATTATATTTATAATTATATAATATTAAAAAGGGTATTTTTATAATTATTACA-TTTTATTTTATTTATAAAAATATTAATTTTAA | 5700 |
| NCYC3594 | 5665 | AGGGGTTATATAATAATTATATTTATAATTATATAATATTAAAAAGGGTATTTTTATAATTATTACATTTTTATTTTATTTATAAAAATATTAATTTTAA | 5764 |
| YJM1078 | 5456 | AGGGGTTATATAATAATTATATTTATAATTATATAATATTAAAAAGGGTATTTTTATAATTATTACATTTTTATTTTATTTATAAAAATATTAATTTTAA | 5555 |
| YJM1439 | 5954 | AGGGGTTATATAATAATTATATTTATAATTATTATATATTAAAAAGGGTATTTTTATAATTATTACATTTTTATTTTATTTATAAAAATATTAATTTTAA | 6053 |
| consensus | 6801 | \*\*\*\*\*\*\*\*\*\*\*\*\*\*\*\*\*\*\*\*\*\*\*\*\*\*\*\*\*\*\*\*...\*\*\*\*\*\*\*\*\*\*-\*\*\*\*\*\*\*\*\*\*\*\*\*\*\*\*\*\*\*\*\*-\*\*\*\*\*\*\*\*\*\*\*\*\*\*\*\*\*\*\*\*\*\*\*\*\*\*\*\*\*\*\*\* | 6900 |
|
| S288C | 5784 | TAAGTATTGAATACTTTATATAATATAAATATTAATTACATAATTAATAATTAAATAATATTTAATAATATTATTTAAATTTATTATTTATAATTATTTA | 5883 |
| YJM1388 | 5588 | TAAGTATTGAATACTTTATATAATATAAATATTAATTACATAATTAATAATTAAATAATATTTAATAATATTATTTAAATTTATTATTTATAATTATTTA | 5687 |
| YJM789 | 5280 | TAAGTATTGAATACTTTATATAATATAAATATTAATTACATAATTAATAATTAAATAATATTTAATAATATTATTTAAATTTATTATTTATAATTATTTA | 5379 |
| YJM1273 | 5363 | TAAGTATTGAATACTTTATATAATATAAATATTAATTACATAATTAATAATTAAATAATATTTAATAATATTATTTAAATTTATTATTTATAATTATTTA | 5462 |
| NCYC3585 | 5376 | TAAGTATTGAATACTTTATATAATATAAATATTAATTACATAATTAATAATTAAATAATATTTAATAATATTATTTAAATTTATTATTTATAATTATTTA | 5475 |
| YJM1401 | 5701 | TAAGTATTGAATACTTTATATAATATAAATATTAATTACATAATTAATAATTAAATAATATTTAATAATATTATTTAAATTTATTATTTATAATTATTTA | 5800 |
| NCYC3594 | 5765 | TAAGTATTGAATACTTTATATAATATAAATATTAATTACATAATTAATAATTAAATAATATTTAATAATATTATTTAAATTTATTATTTATAATTATTTA | 5864 |
| YJM1078 | 5556 | TAAGTATTGAATACTTTATATAATATAAATATTAATTACATAATTAATAATTAAATAATATTTAATAATATTATTTAAATTTATTATTTATAATTATTTA | 5655 |
| YJM1439 | 6054 | TAAGTATTGAATACTTTATATAATATAAATATTAATTACATAATTAATAATTAAATAATATTAAATAATATTATTTAAATTAATTATTTATAATTATTTA | 6153 |
| consensus | 6901 | \*\*\*\*\*\*\*\*\*\*\*\*\*\*\*\*\*\*\*\*\*\*\*\*\*\*\*\*\*\*\*\*\*\*\*\*\*\*\*\*\*\*\*\*\*\*\*\*\*\*\*\*\*\*\*\*\*\*\*\*\*\*.\*\*\*\*\*\*\*\*\*\*\*\*\*\*\*\*\*\*.\*\*\*\*\*\*\*\*\*\*\*\*\*\*\*\*\*\* | 7000 |
|
| S288C | 5884 | TTTATAAAATTCTATTTTTATTATTATTA---TTTTTATTTTATTATTAAAGATTAATATAATAATTATTAATATATTAAAAATCTTTTATTATATTAAT | 5980 |
| YJM1388 | 5688 | TTTATAAAATTCTATTTTTATTATTATTA------TTATTTTATTATTAAAGATTAATATAATAATTATTAATATATTAAAAATCTTTTATTATATTAAT | 5781 |
| YJM789 | 5380 | TTTATAAAATTCTATTTTTATTATTATTA---TTTTTATTTTATTATTAAAAATTAATATAATAATTATTAATATATTAAAAATCTTTTATTATATTAAT | 5476 |
| YJM1273 | 5463 | TTTATAAAATTCTATTTTTATTATTATTA------TTATTTTATTATTAAAGATTAATATAATAATTATTAATATATTAAAAATCTTTTATTATATTAAT | 5556 |
| NCYC3585 | 5476 | TTTATAAAATTCTATTTTTATTATTATTA---TTTTTATTTTATTATTAAAGATTAATATAATAATTATTAATATATTAAAAATCTTTTATTATATTAAT | 5572 |
| YJM1401 | 5801 | TTTATAAAATTATATTTTTATTATTATTA---TTTTTATTTTATTATTAAAAATTAATATAATAATTATTAATATATTAAAAATCTTTTATTATATTAAT | 5897 |
| NCYC3594 | 5865 | TTTATAAAATTCTATTTTTATTATTATTA---T---TATTTTATTATTAAAGATTAATATAATAATTATTAATATATTAAAAATCTTTTATTATATTAAT | 5958 |
| YJM1078 | 5656 | TTTATAAAATTCTATTTTTATTATTATTATTTT---TAATTTATTATTAAAGATTAATATAATAATTATTAATATATTAAAAATCTTTTATTATATTAAT | 5752 |
| YJM1439 | 6154 | TTTATAAAATTCTATTTTTATTATTATTA---TTTTTATTTTATTATTAAAAATTAATATAATAATTATTAATATATTAAAAATCTTTTATTATATTAAT | 6250 |
| consensus | 7001 | \*\*\*\*\*\*\*\*\*\*\*.\*\*\*\*\*\*\*\*\*\*\*\*\*\*\*\*\*-------\*\*.\*\*\*\*\*\*\*\*\*\*\*\*.\*\*\*\*\*\*\*\*\*\*\*\*\*\*\*\*\*\*\*\*\*\*\*\*\*\*\*\*\*\*\*\*\*\*\*\*\*\*\*\*\*\*\*\*\*\*\*\* | 7100 |
|
| S288C | 5981 | ATTTATAAAAAAGTATTTAAT-AAAAAAGATGTATAAATTTATAAATTATATAATATTATTAATTTATATAATAATAATATTATAACTTTGTGATTGTCA | 6079 |
| YJM1388 | 5782 | ATTTATAAAAAAGTATTTAAT-AAAAAAGATGTATAAATTTATAAATTATATAATATTATTAATTTATATAATAATAATATTATAACTTTGTGATTGTCA | 5880 |
| YJM789 | 5477 | ATTTATAAAAAAGTATTTAATAAAAAAAGATGTCTAAATTTATAAATTATATAATATTATTAATTTATATAATAATAATATTATAACTTTGTGATTGTCA | 5576 |
| YJM1273 | 5557 | ATTTATAAAAAAGTATTTAAT-AAAAAAGATGTCTAAATTTATAAATTATATAATATTATTAATTTATATAATAATAATATTATAACTTTGTGATTGTCA | 5655 |
| NCYC3585 | 5573 | ATTTATAAAAAAGTATTTAAT-AAAAAAGATGTATAAATTTATAAATTATATAATATTATTAATTTATATAATAATAATATTATAACTTTGTGATTGTCA | 5671 |
| YJM1401 | 5898 | ATTTATAAAAAAGTATTTAATAAAAAAAGATGTCTAAATTTATAAATTATATAATATTATTAATTTATATAATAATAATATTATAACTTTGTGATTGTCA | 5997 |
| NCYC3594 | 5959 | ATTTATAAAAAAGTATTTAAT-AAAAAAGATGTATAAATTTATAAATTATATAATATTATTAATTTATATAATAATAATATTATAACTTTGTGATTGTCA | 6057 |
| YJM1078 | 5753 | ATTTATAAAAAAGTATTTAAT-AAAAAAGATGTATAAATTTATAAATTATATAATATTATTAATTTATATAATAATAATATTATAACTTTGTGATTGTCA | 5851 |
| YJM1439 | 6251 | ATTTATAAAAAAGTATTTAAT-AAAAAAGATGTCTAAATTTATAAATTATATAATATTATTAATTTATATAATAATAATATTATAACTTTGTGATTGTCA | 6349 |
| consensus | 7101 | \*\*\*\*\*\*\*\*\*\*\*\*\*\*\*\*\*\*\*\*\*-\*\*\*\*\*\*\*\*\*\*\*.\*\*\*\*\*\*\*\*\*\*\*\*\*\*\*\*\*\*\*\*\*\*\*\*\*\*\*\*\*\*\*\*\*\*\*\*\*\*\*\*\*\*\*\*\*\*\*\*\*\*\*\*\*\*\*\*\*\*\*\*\*\*\*\*\*\* | 7200 |
|
| S288C | 6080 | ATTTAGTTAATCATTGTTATTAATAAAGGAAAGATATAAAAAATATTCTCCTTCTTAAAAA------GGGGTTCGGTTCCCCCCCGTAAG---------- | 6163 |
| YJM1388 | 5881 | ATTTAGTTAATCATTGTTATTAATAAAGGAAAGATATAAAAAATATTCTCCTTCTTAAAAA------GGGGTTCGGTTCCCCCCCGTAAGTG-------- | 5966 |
| YJM789 | 5577 | ATTTAGTTAATCATTGTTATTAATAAAGGAAAGATATAAAAAATATTCTCCTTCTTAAAAA------GGGGTTCGGT-CCCCCC-GTAAGTATAAGTATA | 5668 |
| YJM1273 | 5656 | ATTTAGTTAATCATTGTTATTAATAAAGGAAAGATATAAAAAATATTCTCCTTCTTAAGAATAAAAAGGGGTTCGGTTCCCCCCCGTAAG---------- | 5745 |
| NCYC3585 | 5672 | ATTTAGTTAATCATTGTTATTAATAAAGGAAAGATATAAAAAATATTCTCCTTCTTAAAAA-----GGGGTTCGGTT-CCCCCCCGTAAG---------- | 5755 |
| YJM1401 | 5998 | ATTTAGTTAATCATTGTTATTAATAAAGGAAAGATATAAAAAATATTCTCCTTC------------GGGGTTCGGT---CCCCCCGTAAGTATA------ | 6076 |
| NCYC3594 | 6058 | ATTTAGTTAATCATTGTTATTAATAAAGGAAAGATATAAAAAATATTCTCCTTCTTAAAAA-----GGGGTTCGGTT-CCCCCCGTAAGGG--------- | 6142 |
| YJM1078 | 5852 | ATTTAGTTAATCATTGTTATTAATAAAGGAAAGATATAAAAAATATTCTCCTTCTTAAAAA-----GGGGTTCGGTT-CCCCCCGTAAG----------- | 5934 |
| YJM1439 | 6350 | ATTTAGTTAATCATTGTTATTAATAAAGGAAAGATATAAAAAATATTCTCCTTC------------GGGGTTCGGTCCCCCTCCCGTTAGAATAAATATA | 6437 |
| consensus | 7201 | \*\*\*\*\*\*\*\*\*\*\*\*\*\*\*\*\*\*\*\*\*\*\*\*\*\*\*\*\*\*\*\*\*\*\*\*\*\*\*\*\*\*\*\*\*\*\*\*\*\*\*\*\*\*----.-------.\*\*\*.\*..\*...-\*\*.\*\*.....-..---.---- | 7300 |
|
| S288C | 6164 | --GGGGGGGTCCCTCACTCCTTTGGTCGGACTCCTT--------------------------------------------------------------CG | 6199 |
| YJM1388 | 5967 | ----GGGGGTCCCTCACTCCTTTGGTCGGACTCCTT--------------------------------------------------------------CG | 6000 |
| YJM789 | 5669 | -CGGGGGGGTCCCTCACTCCTTC----------------------------------------------------------------------------- | 5690 |
| YJM1273 | 5746 | --GGGGGGGTCCCTCACTGCTTTGGTCGGAC--------------------------------------------------------------------- | 5774 |
| NCYC3585 | 5756 | --GGGGGGGTCCCTCACTCCTTTGGTCGGACTCCTT--------------------------------------------------------------CG | 5791 |
| YJM1401 | 6077 | ---CGGGGGTCCCTCACTCCTTCGGCCGGACTCCTTCTTTAAAAGGGGTTCGGTCCCCCTCCCATTAGTATAGTATAGGGAGGGGTCCCTCACTCCTTCG | 6173 |
| NCYC3594 | 6143 | ----GGGGGTCCCTCACTCCTTTGGTCGGACTCCTT--------------------------------------------------------------CG | 6176 |
| YJM1078 | 5935 | --GGGGGGGTCCCTCACTCCTTTGGTCGGACTCCTT--------------------------------------------------------------CG | 5970 |
| YJM1439 | 6438 | GGGAGGGGGTCCCTCACTCCTT------------------------------------------------------------------------------ | 6459 |
| consensus | 7301 | -.-.\*\*\*\*\*\*\*\*\*\*\*\*\*\*.\*\*\*.--.-------------------------------------------------------------------------- | 7400 |
|
| S288C | 6200 | GGGTCCGCCCCGCGGGGGCGGGCCGGACTAATTTAACTTTTAATATTAATATTAATATTATTTATATTTTTAATATATAAAAATAAATAATTTTATTTTT | 6299 |
| YJM1388 | 6001 | GGGTCCGCCCCGCGGGGGCGGGCCGGACTAATTTAACTTTTAATATTAATATTAATATTATTTATATTTTTAATATATAAAAATAAATAATTTTATTTTT | 6100 |
| YJM789 | 5691 | --------------------GGCCGGACTAATTTAACTTTTAATATTAATATTAATATTATTTATATTTTTAATATATAAAAATAAATAATTTTATTTTT | 5770 |
| YJM1273 | 5775 | ----------------------------TAATTTAACTTTTAATATTAATATTAATATTATTTATATTTTTAATATATAAAAATAAATAATTTTATTTTT | 5846 |
| NCYC3585 | 5792 | GGGTCCGCCCCGCGGGGGCGGGCCGGACTAATTTAACTTTTAATATTAATATTAATATTATTTATATTTTTAATATATAAAAATAAATAATTTTATTTTT | 5891 |
| YJM1401 | 6174 | GGGTCCGCCCCGCGGGGGCGGGCCGGACTAATTTAACTTTTAATATTAATATTAATATTATTTATATTTTTAATATATAAAAATAAATAATTTTATTTTT | 6273 |
| NCYC3594 | 6177 | GGGTCCGCCCCGCGGGGGCGGGCCGGACTAATTTAACTTTTAATATTAATATTAATATTATTTATATTTTTAATATATAAAAATAAATAATTTTATTTTT | 6276 |
| YJM1078 | 5971 | GGGTCCGCCCCGCGGGGGCGGGCCGGACTAATTTAACTTTTAATATTAATATTAATATTATTTATATTTTTAATATATAAAAATAAATAATTTTATTTTT | 6070 |
| YJM1439 | 6460 | ---------------------------------------------------------------------------------------------------- | 6459 |
| consensus | 7401 | ---------------------------------------------------------------------------------------------------- | 7500 |
|
| S288C | 6300 | ATTAA----------------------------------------------------------------------------------------TAGT--- | 6308 |
| YJM1388 | 6101 | ATTAA----------------------------------------------------------------------------------------TAGT--- | 6109 |
| YJM789 | 5771 | ATTAA----------------------------------------------------------------------------------------TAGT--- | 5779 |
| YJM1273 | 5847 | ATTAA----------------------------------------------------------------------------------------TAGT--- | 5855 |
| NCYC3585 | 5892 | ATTAA----------------------------------------------------------------------------------------TAGTTCC | 5903 |
| YJM1401 | 6274 | ATTAA----------------------------------------------------------------------------------------TAGT--- | 6282 |
| NCYC3594 | 6277 | ATTAA----------------------------------------------------------------------------------------TAGT--- | 6285 |
| YJM1078 | 6071 | ATTAA----------------------------------------------------------------------------------------TAGTTCC | 6082 |
| YJM1439 | 6460 | -----CTTAAAAAGGAGAAATAAATAATTTTATTTTTATTAATAGTGAACACCTTTATTTAAAGGTGTGAACCAATCCCGCAAGGCAAGGGAGGAGTTCC | 6554 |
| consensus | 7501 | ---------------------------------------------------------------------------------------------.\*\*\*--- | 7600 |
|
| S288C | 6309 | --------------------------------------ATATTATATAAACAATAAAATAGTATTAATTATATAAAATTTATATAAAATATATATAAATT | 6370 |
| YJM1388 | 6110 | --------------------------------------ATATTATATAAACAATAAAATAGTATTAATTATATAAAATTTATATAAAATATATATAAATT | 6171 |
| YJM789 | 5780 | --------------------------------------ATATTATATAAACAATAAAATAGTATTAATTATAAAAAATTTATATAAAATATATATAAATT | 5841 |
| YJM1273 | 5856 | --------------------------------------ATATTATATAAACAATAAAATAGTATTAATTATATAAAATTTATATAAAATATATATAAATT | 5917 |
| NCYC3585 | 5904 | GGGCCCCGGCCACGGGAGCCGGAACCCCGGAAGGAGTAATATTATATAAACAATAAAATAGTATTAATTATATAAAATTTATATAAAATATATATAAATT | 6003 |
| YJM1401 | 6283 | --------------------------------------ATATTATATAAACAATAAAATAGTATTAATTATATAAAATTTATATAAAATATATATAAATT | 6344 |
| NCYC3594 | 6286 | --------------------------------------ATATTATATAAACAATAAAATAGTATTAATTATATAAAATTTATATAAAATATATATAAATT | 6347 |
| YJM1078 | 6083 | GGGCCCCGGCCACGGGAGCCGGAACCCCGGAAGGAGTAATATTATATAAACAATAAAATAGTATTAATTATATAAAATTTATATAAAATATATATAAATT | 6182 |
| YJM1439 | 6555 | GGGGCCCGGCCACGGGAGCCGGAACCCCGGAAGGAGTAATATTATATAAACAATAAAATAGTATTAATTATATAAAATTTATATAAAATATATATAAATT | 6654 |
| consensus | 7601 | ---.----------------------------------\*\*\*\*\*\*\*\*\*\*\*\*\*\*\*\*\*\*\*\*\*\*\*\*\*\*\*\*\*\*\*\*\*\*.\*\*\*\*\*\*\*\*\*\*\*\*\*\*\*\*\*\*\*\*\*\*\*\*\*\*\* | 7700 |
|
| S288C | 6371 | TATTATATATATATATATTAATATTTTAATAAAG----TTTTTATTATAAATTTATTTATTTAT-----------TTAT--------------------- | 6434 |
| YJM1388 | 6172 | TATTATATATATATATATTAATATTTTAATAAAG---TTTTTTATTATAAATTTATTTATTTATTTATTTATTAATTAT--------------------- | 6247 |
| YJM789 | 5842 | TATTATATATATATATATTAATATTTTAATAAAG----TTTTTATTATAAATTTATTTATTTAT-----------TTAT--------------------- | 5905 |
| YJM1273 | 5918 | TATTATATATATATATATTAATATTTTAATAAAGTTTTTTTTTATTATAAATTTATTTATTTAT-----------TTATTTATTTATTTATTAATTATTA | 6006 |
| NCYC3585 | 6004 | TATTATATATATATATATTAATATTTTAATAAAG----TTTTTATTATAAATTTATTTATTTAT-----------TTAT--------------------- | 6067 |
| YJM1401 | 6345 | TATTATATATATATATATTAATATTTTAATAAAG----TTTTTATTATAAATTTATTTATTTAT-----------TTAT--------------------- | 6408 |
| NCYC3594 | 6348 | TATTATATATATATATATTAATATTTTAATAAAG----TTTTTATTATAAATTTATTTATTTAT-----------TTAT--------------------- | 6411 |
| YJM1078 | 6183 | TATTATATATATATATATTAATATTTTAATAAAG----TTTTTATTATAAATTTATTTATTTAT-----------TTAT--------------------- | 6246 |
| YJM1439 | 6655 | TATTATATATATATATATTAATATTTTAATAAAG---TTTTTTATTATAAATTTATTTATTTAT-----------TTAT--------------------- | 6719 |
| consensus | 7701 | \*\*\*\*\*\*\*\*\*\*\*\*\*\*\*\*\*\*\*\*\*\*\*\*\*\*\*\*\*\*\*\*\*\*----\*\*\*\*\*\*\*\*\*\*\*\*\*\*\*\*\*\*\*\*\*\*\*\*\*\*-----------\*\*\*\*--------------------- | 7800 |
|
| S288C | 6435 | --TATAATATTAATAATTTATTTATTATTATATAAGTAATAAATAATAGTTTTATATAATAATAATA--ATATATATATATATATATTATTATATTAGTT | 6530 |
| YJM1388 | 6248 | --TATATTTTAATTTATTAATTTATTATTATATAAGTAATAAATAATAGTTTTATATAATAATAATAATATATATATATATATATATAATTATATTAGTT | 6345 |
| YJM789 | 5906 | --TATAATATTAATAATTTATTTATTATTATATAAGTAATAAATAATAGTTTTATATAATAATAATAATATATATATATATATATATAATTATATTAGTT | 6003 |
| YJM1273 | 6007 | TATTTTAATTTATTTATTTATTTATTATTATATAAGTAATAAATAATAGTTTTATATAATAATAATAATATATATATATATATATATAATTATATTAGTT | 6106 |
| NCYC3585 | 6068 | --TATAATATTAATAATTTATTTATTATTATATAAGTAATAAATAATAGTTTTATATAATAATAATAATATATATATATATATATATAATTATATTAGTT | 6165 |
| YJM1401 | 6409 | --TATAATATTAATAATTTATTTATTATTATATAAGTAATAAATAATAGTTTTATATAATAATAATAATATATATATATATATATATAATTATATTAGTT | 6506 |
| NCYC3594 | 6412 | --TATAATATTAATAATTTATTTATTATTATATAAGTAATAAATAATAGTTTTATATAATAATAATAA--TATATATATATATATATTATTATATTAGTT | 6507 |
| YJM1078 | 6247 | --TATAATATTAATAATTTATTTATTATTATATAAGTAATAAATAATAGTTTTATATAATAATAATAA--TATATATATATATATATTATTATATTAGTT | 6342 |
| YJM1439 | 6720 | --TATAATATTAATAATTTATTTATTATTATATAAGTAATAAATAATAGTTTTATATAATAATAATAATATATATATATATATATATAATTATATTAGTT | 6817 |
| consensus | 7801 | --\*.\*....\*.\*.\*.\*\*\*.\*\*\*\*\*\*\*\*\*\*\*\*\*\*\*\*\*\*\*\*\*\*\*\*\*\*\*\*\*\*\*\*\*\*\*\*\*\*\*\*\*\*\*\*\*\*\*\*---\*\*\*\*\*\*\*\*\*\*\*\*\*\*\*\*\*.\*\*\*\*\*\*\*\*\*\*\*\* | 7900 |
|
| S288C | 6531 | ATATAATAAGGAAAAGTAAAAAATTTATAAGAATATGATGTTGGTTCAGATTAAGCGCTAAATAAGGACATGACACATGCGAATCATACGTTTATTATTG | 6630 |
| YJM1388 | 6346 | ATATAATAAGGAAAAGTAAAAATTTTATAAGAATATGATGTTGGTTCAGATTAAGCGCTAAATAAGGACATGACACATGCGAATCATACGTTTATTATTG | 6445 |
| YJM789 | 6004 | ATATAATAAGGAAAAGTAAAAAATTTATAAGAATATGATGTTGGTTCAGATTAAGCGCTAAATAAGGACATGACACATGCGAATCATACGTTTATTATTG | 6103 |
| YJM1273 | 6107 | ATATAATAAGGAAAAGTAAAAAATTTATAAGAATATGATGTTGGTTCAGATTAAGCGCTAAATAAGGACATGACACATGCGAATCATACGTTTATTATTG | 6206 |
| NCYC3585 | 6166 | ATATAATAAGGAAAAGTAAAAAATTTATAAGAATATGATGTTGGTTCAGATTAAGCGCTAAATAAGGACATGACACATGCGAATCATACGTTTATTATTG | 6265 |
| YJM1401 | 6507 | ATATAATAAGGAAAAGTAAAAAATTTATAAGAATATGATGTTGGTTCAGATTAAGCGCTAAATAAGGACATGACACATGCGAATCATACGTTTATTATTG | 6606 |
| NCYC3594 | 6508 | ATATAATAAGGAAAAGTAAAAATTTTATAAGAATATGATGTTGGTTCAGATTAAGCGCTAAATAAGGACATGACACATGCGAATCATACGTTTATTATTG | 6607 |
| YJM1078 | 6343 | ATATAATAAGGAAAAGTAAAAATTTTATAAGAATATGATGTTGGTTCAGATTAAGCGCTAAATAAGGACATGACACATGCGAATCATACGTTTATTATTG | 6442 |
| YJM1439 | 6818 | ATATAATAAGGTAAAGTAAAAAATTTATAAGAATATGATGTTGGTTCAGATTAAGCGCTAAATAAGGACATGACACATGCGAATCATACGTTTATTATTG | 6917 |
| consensus | 7901 | \*\*\*\*\*\*\*\*\*\*\*.\*\*\*\*\*\*\*\*\*\*.\*\*\*\*\*\*\*\*\*\*\*\*\*\*\*\*\*\*\*\*\*\*\*\*\*\*\*\*\*\*\*\*\*\*\*\*\*\*\*\*\*\*\*\*\*\*\*\*\*\*\*\*\*\*\*\*\*\*\*\*\*\*\*\*\*\*\*\*\*\*\*\*\*\*\*\*\* | 8000 |
|
| S288C | 6631 | ATAAGATAATAAATATGTGGTGTAAACGTGAGTAATTTTATTAGGAATTAATGAACTATAGAATAAGCTAAATACTTAATATATTATTATATAAAAATAA | 6730 |
| YJM1388 | 6446 | ATAAGATAATAAATATGTGGTGTAAACGTGAGTAATTTTATTAGGAATTAATGAACTATAGAATAAGCTAAATACTTAATATATTATTATATAAAAATAA | 6545 |
| YJM789 | 6104 | ATAAGATAATAAATATGTGGTGTAAACGTGAGTAATTTTATTAGGAATTAATGAACTATAGAATAAGCTAAATACTTAATATATTATTATATAAAAATAA | 6203 |
| YJM1273 | 6207 | ATAAGATAATAAATATGTGGTGTAAACGTGAGTAATTTTATTAGGAATTAATGAACTATAGAATAAGCTAAATACTTAATATATTATTATATAAAAATAA | 6306 |
| NCYC3585 | 6266 | ATAAGATAATAAATATGTGGTGTAAACGTGAGTAATTTTATTAGGAATTAATGAACTATAGAATAAGCTAAATACTTAATATATTATTATATAAAAATAA | 6365 |
| YJM1401 | 6607 | ATAAGATAATAAATATGTGGTGTAAACGTGAGTAATTTTATTAGGAATTAATGAACTATAGAATAAGCTAAATACTTAATATATTATTATATAAAAATAA | 6706 |
| NCYC3594 | 6608 | ATAAGATAATAAATATGTGGTGTAAACGTGAGTAATTTTATTAGGAATTAATGAACTATAGAATAAGCTAAATACTTAATATATTATTATATAAAAATAA | 6707 |
| YJM1078 | 6443 | ATAAGATAATAAATATGTGGTGTAAACGTGAGTAATTTTATTAGGAATTAATGAACTATAGAATAAGCTAAATACTTAATATATTATTATATAAAAATAA | 6542 |
| YJM1439 | 6918 | ATAAGATAATAAATATGTGGTGTAAACGTGAGTAATTTTATTAGGAATTAATGAACTATAGAATAAGCTAAATACTTAATATATTATTATATAAAAATAA | 7017 |
| consensus | 8001 | \*\*\*\*\*\*\*\*\*\*\*\*\*\*\*\*\*\*\*\*\*\*\*\*\*\*\*\*\*\*\*\*\*\*\*\*\*\*\*\*\*\*\*\*\*\*\*\*\*\*\*\*\*\*\*\*\*\*\*\*\*\*\*\*\*\*\*\*\*\*\*\*\*\*\*\*\*\*\*\*\*\*\*\*\*\*\*\*\*\*\*\*\*\*\*\*\*\*\*\* | 8100 |
|
| S288C | 6731 | TTTATATAATAAAAAGGATATATATATAATATATATTTATCTATAGTCAAGCCAATAATGGTTTAGGTAGTAGGTTTATTAAGAGTTAAACCTAGCCAAC | 6830 |
| YJM1388 | 6546 | TTTATATAATAAAAAGGATATATATATAATATATATTTATCTATAGTCAAGCCAATAATGGTTTAGGTAGTAGGTTTATTAAGAGTTAAACCTAGCCAAC | 6645 |
| YJM789 | 6204 | TTTATATAATAAAAAGGATATATATATAATATATATTTATCTATAGTCAAGCCAATAATGGTTTAGGTAGTAGGTTTATTAAGAGTTAAACCTAGCCAAC | 6303 |
| YJM1273 | 6307 | TTTATATAATAAAAAGGATATATATATAATATATATTTATCTATAGTCAAGCCAATAATGGTTTAGGTAGTAGGTTTATTAAGAGTTAAACCTAGCCAAC | 6406 |
| NCYC3585 | 6366 | TTTATATAATAAAAAGGATATATATATAATATATATTTATCTATAGTCAAGCCAATAATGGTTTAGGTAGTAGGTTTATTAAGAGTTAAACCTAGCCAAC | 6465 |
| YJM1401 | 6707 | TTTATATAATAAAAAGGATATATATATAATATATATTTATCTATAGTCAAGCCAATAATGGTTTAGGTAGTAGGTTTATTAAGAGTTAAACCTAGCCAAC | 6806 |
| NCYC3594 | 6708 | TTTATATAATAAAAAGGATATATATATAATATATATTTATCTATAGTCAAGCCAATAATGGTTTAGGTAGTAGGTTTATTAAGAGTTAAACCTAGCCAAC | 6807 |
| YJM1078 | 6543 | TTTATATAATAAAAAGGATATATATATAATATATATTTATCTATAGTCAAGCCAATAATGGTTTAGGTAGTAGGTTTATTAAGAGTTAAACCTAGCCAAC | 6642 |
| YJM1439 | 7018 | TTTATATAATAAAAAGGATATATATATAATATATATTTATCTATAGTCAAGCCAATAATGGTTTAGGTAGTAGGTTTATTAAGAGTTAAACCTAGCCAAC | 7117 |
| consensus | 8101 | \*\*\*\*\*\*\*\*\*\*\*\*\*\*\*\*\*\*\*\*\*\*\*\*\*\*\*\*\*\*\*\*\*\*\*\*\*\*\*\*\*\*\*\*\*\*\*\*\*\*\*\*\*\*\*\*\*\*\*\*\*\*\*\*\*\*\*\*\*\*\*\*\*\*\*\*\*\*\*\*\*\*\*\*\*\*\*\*\*\*\*\*\*\*\*\*\*\*\*\* | 8200 |
|
| S288C | 6831 | GATCCATAATCGATAATGAAAGTTAGAACGATCACGTTGACTCTGAAATATAGTCAATATCTATAAGATACAGCAGTGAGGAATATTGGACAATGATCGA | 6930 |
| YJM1388 | 6646 | GATCCATAATCGATAATGAAAGTTAGAACGATCACGTTGACTCTGAAATATAGTCAATATCTATAAGATACAGCAGTGAGGAATATTGGACAATGATCGA | 6745 |
| YJM789 | 6304 | GATCCATAATCGATAATGAAAGTTAGAACGATCACGTTGACTCTGAAATATAGTCAATATCTATAAGATACAGCAGTGAGGAATATTGGACAATGATCGA | 6403 |
| YJM1273 | 6407 | GATCCATAATCGATAATGAAAGTTAGAACGATCACGTTGACTCTGAAATATAGTCAATATCTATAAGATACAGCAGTGAGGAATATTGGACAATGATCGA | 6506 |
| NCYC3585 | 6466 | GATCCATAATCGATAATGAAAGTTAGAACGATCACGTTGACTCTGAAATATAGTCAATATCTATAAGATACAGCAGTGAGGAATATTGGACAATGATCGA | 6565 |
| YJM1401 | 6807 | GATCCATAATCGATAATGAAAGTTAGAACGATCACGTTGACTCTGAAATATAGTCAATATCTATAAGATACAGCAGTGAGGAATATTGGACAATGATCGA | 6906 |
| NCYC3594 | 6808 | GATCCATAATCGATAATGAAAGTTAGAACGATCACGTTGACTCTGAAATATAGTCAATATCTATAAGATACAGCAGTGAGGAATATTGGACAATGATCGA | 6907 |
| YJM1078 | 6643 | GATCCATAATCGATAATGAAAGTTAGAACGATCACGTTGACTCTGAAATATAGTCAATATCTATAAGATACAGCAGTGAGGAATATTGGACAATGATCGA | 6742 |
| YJM1439 | 7118 | GATCCATAATCGATAATGAAAGTTAGAACGATCACGTTGACTCTGAAATATAGTCAATATCTATAAGATACAGCAGTGAGGAATATTGGACAATGATCGA | 7217 |
| consensus | 8201 | \*\*\*\*\*\*\*\*\*\*\*\*\*\*\*\*\*\*\*\*\*\*\*\*\*\*\*\*\*\*\*\*\*\*\*\*\*\*\*\*\*\*\*\*\*\*\*\*\*\*\*\*\*\*\*\*\*\*\*\*\*\*\*\*\*\*\*\*\*\*\*\*\*\*\*\*\*\*\*\*\*\*\*\*\*\*\*\*\*\*\*\*\*\*\*\*\*\*\*\* | 8300 |
|
| S288C | 6931 | AAGATTGATCCAGTTACTTATTAGGATGATATATAAAAATA-TTTTATTTTATTT----------------------------------------ATAAA | 6989 |
| YJM1388 | 6746 | AAGATTGATCCAGTTACTTATTAGGATGATATATAAAAATA-TTTTATTTTATTT----------------------------------------ATAAA | 6804 |
| YJM789 | 6404 | AAGATTGATCCAGTTACTTATTAGGATGATATATAAAAATA-TTTTATTTTATTT----------------------------------------ATAAA | 6462 |
| YJM1273 | 6507 | AAGATTGATCCAGTTACTTATTAGGATGATATATAAAAATA-TTTTATTTTATTT----------------------------------------ATAAA | 6565 |
| NCYC3585 | 6566 | AAGATTGATCCAGTTACTTATTAGGATGATATATAAAAATA-TTTTATTTTATTTAGTTCCGGGGCCCGGCCACGGGAGCCGGAACCCCGAAAGGAGAAA | 6664 |
| YJM1401 | 6907 | AAGATTGATCCAGTTACTTATTAGGATGATATATAAAAATA-TTTTATTTTATTT----------------------------------------ATAAA | 6965 |
| NCYC3594 | 6908 | AAGATTGATCCAGTTACTTATTAGGATGATATATAAAAATA-TTTTATTTTATTT----------------------------------------ATAAA | 6966 |
| YJM1078 | 6743 | AAGATTGATCCAGTTACTTATTAGGATGATATATAAAAATATTTTTATTTTATTTAGTTCCGGGGCCCGGCCACGGGAGCCGGAACCCCGAAAGGAGAAA | 6842 |
| YJM1439 | 7218 | AAGATTGATCCAGTTACTTATTAGGATGATATATAAAAATA-TTTTATTTTATTT----------------------------------------ATAAA | 7276 |
| consensus | 8301 | \*\*\*\*\*\*\*\*\*\*\*\*\*\*\*\*\*\*\*\*\*\*\*\*\*\*\*\*\*\*\*\*\*\*\*\*\*\*\*\*\*-\*\*\*\*\*\*\*\*\*\*\*\*\*----------------------------------------\*.\*\*\* | 8400 |
|
| S288C | 6990 | TATTAAATATTTATAATAATAATAATAATAATATATATATATAAATTGATTAAAAATAAAATCCATAAATAATTAAAATAATGATATTAATTACCATATA | 7089 |
| YJM1388 | 6805 | TATTAAATATTTATAATAATAATAATAATAATATATATATATAAATTGATTAAAAATAAAATCCATAAATAATTAAAATAATGATATTAATTACCATATA | 6904 |
| YJM789 | 6463 | TATTTAATATTTATAATAATAATAATAATAATATATATATATAAATTGATTAAAAATAAAATCCATAAATAATTAAAATAATGATATTAATTACCATATA | 6562 |
| YJM1273 | 6566 | TATTAAATATTTATAATAATAATAATAATAATATATATATATAAATTGATTAAAAATAAAATCCATAAATAATTAAAATAATGATATTAATTACCATATA | 6665 |
| NCYC3585 | 6665 | TATTTAATATTTATAATAATAATAATAATAATATATATATATAAATTGATTAAAAATAAAATCCATAAATAATTAAAATAATGATATTAATTACCATATA | 6764 |
| YJM1401 | 6966 | TATTAAATATTTATAATAATAATAATAATAATATATATATATAAATTGATTAAAAATAAAATCCATAAATAATTAAAATAATGATATTAATTACCATATA | 7065 |
| NCYC3594 | 6967 | TATTAAATATTTATAATAATAATAATAATAATATATATATATAAATTGATTAAAAATAAAATCCATAAATAATTAAAATAATGATATTAATTACCATATA | 7066 |
| YJM1078 | 6843 | TATTAAATATTT---ATAATAATAATAATAATATATATATATAAATTGATTAAAAATAAAATCCATAAATAATTAAAATAATGATATTAATTACCATATA | 6939 |
| YJM1439 | 7277 | TATTAAATATTTATAATAATAATAATAATAATATATATATATAAATTGATTAAAAATAAAATCCATAAATAATTAAAATAATGATATTAATTACCATATA | 7376 |
| consensus | 8401 | \*\*\*\*.\*\*\*\*\*\*\*---\*\*\*\*\*\*\*\*\*\*\*\*\*\*\*\*\*\*\*\*\*\*\*\*\*\*\*\*\*\*\*\*\*\*\*\*\*\*\*\*\*\*\*\*\*\*\*\*\*\*\*\*\*\*\*\*\*\*\*\*\*\*\*\*\*\*\*\*\*\*\*\*\*\*\*\*\*\*\*\*\*\*\*\*\* | 8500 |
|
| S288C | 7090 | TA-TTTTTATATGGATATATATATTAATAATAATATTAATTTTATTATTATTAATAATATATTTTAATAGTCCTGACTAATATTTGTGCCAGCAGTCGCG | 7188 |
| YJM1388 | 6905 | TA-TTTTTATATGGATATATATATTAATAATAATATTAA-TTTATTATTATTAATAATATATTTTAATAGTCCTGACTAATATTTGTGCCAGCAGTCGCG | 7002 |
| YJM789 | 6563 | TA-TTTTTATATGGATATATATATTAATAATAATATTAATTTTATTATTATTAATAATATATTTTAATAGTCCTGACTAATATTTGTGCCAGCAGTCGCG | 6661 |
| YJM1273 | 6666 | TA-TTTTTATATGGATATATATATTAATAATAATATTAATTTTATTATTATTAATAATATATTTTAATAGTCCTGACTAATATTTGTGCCAGCAGTCGCG | 6764 |
| NCYC3585 | 6765 | TA-TTTTTATATGGATATATATATTAATAATAATATTAATTTTATTATTATTAATAATATATTTTAATAGTCCTGACTAATATTTGTGCCAGCAGTCGCG | 6863 |
| YJM1401 | 7066 | TA-TTTTTATATGGATATATATATTAATAATAATATTAATTTTATTATTATTAATAATATATTTTAATAGTCCTGACTAATATTTGTGCCAGCAGTCGCG | 7164 |
| NCYC3594 | 7067 | TA-TTTTTATATGGATATATATATTAATAATAATATTAATTTTATTATTATTAATAATATATTTTAATAGTCCTGACTAATATTTGTGCCAGCAGTCGCG | 7165 |
| YJM1078 | 6940 | TA-TTTTTATATGGATATATATATTAATAATAAAATTAA-TTTATTATTATTAATAATATATTTTAATAGTCCTGACTAATATTTGTGCCAGCAGTCGCG | 7037 |
| YJM1439 | 7377 | TATTTTTTATATGGATATATATATTAATAATAATATTAATTTTATTATTATTAATAATATATTTTAATAGTCCTGACTAATATTTGTGCCAGCAGTCGCG | 7476 |
| consensus | 8501 | \*\*-\*\*\*\*\*\*\*\*\*\*\*\*\*\*\*\*\*\*\*\*\*\*\*\*\*\*\*\*\*\*.\*\*\*\*\*-\*\*\*\*\*\*\*\*\*\*\*\*\*\*\*\*\*\*\*\*\*\*\*\*\*\*\*\*\*\*\*\*\*\*\*\*\*\*\*\*\*\*\*\*\*\*\*\*\*\*\*\*\*\*\*\*\*\*\*\* | 8600 |
|
| S288C | 7189 | GTAACACAAAGAGGGCGAGCGTTAATCATAATGGTTTAAAGGATCCGTAGAATGAATTATATATTATAATTTAGAGTTAAT-AAAATATAATTAAAGAAT | 7287 |
| YJM1388 | 7003 | GTAACACAAAGAGGGCGAGCGTTAATCATAATGGTTTAAAGGATCCGTAGAATGAATTATATATTATAATTTAGAGTTAAT-AAAAT-TAATTAAAGAAT | 7100 |
| YJM789 | 6662 | GTAACACAAAGAGGGCGAGCGTTAATCATAATGGTTTAAAGGATCCGTAGAATGAATTATATATTATAATTTAGAGTTAAT-AAAAT-TAATTAAAGAAT | 6759 |
| YJM1273 | 6765 | GTAACACAAAGAGGGCGAGCGTTAATCATAATGGTTTAAAGGATCCGTAGAATGAATTATATATTATAATTTAGAGTTAAT-AAAAT-TAATTAAAGAAT | 6862 |
| NCYC3585 | 6864 | GTAACACAAAGAGGGCGAGCGTTAATCATAATGGTTTAAAGGATCCGTAGAATGAATTATATATTATAATTTAGAGTTAAT-AAAAT-TAATTAAAGAAT | 6961 |
| YJM1401 | 7165 | GTAACACAAAGAGGGCGAGCGTTAATCATAATGGTTTAAAGGATCCGTAGAATGAATTATATATTATAATTTAGAGTTAAT-AAAAT-TAATTAAAGAAT | 7262 |
| NCYC3594 | 7166 | GTAACACAAAGAGGGCGAGCGTTAATCATAATGGTTTAAAGGATCCGTAGAATGAATTATATATTATAATTTAGAGTTAAT-AAATA-TAATTAAAGAAT | 7263 |
| YJM1078 | 7038 | GTAACACAAAGAGGGCGAGCGTTAATCATAATGGTTTAAAGGATCCGTAGAATGAATTATATATTATAATTTAGAGTTAATAAAATA-TAATTAAAGAAT | 7136 |
| YJM1439 | 7477 | GTAACACAAAGAGGGCGAGCGTTAATCATAATGGTTTAAAGGATCCGTAGAATGAATTATATATTATAATTTAGAGTTAAT-AAAATATAATTAAAGAAT | 7575 |
| consensus | 8601 | \*\*\*\*\*\*\*\*\*\*\*\*\*\*\*\*\*\*\*\*\*\*\*\*\*\*\*\*\*\*\*\*\*\*\*\*\*\*\*\*\*\*\*\*\*\*\*\*\*\*\*\*\*\*\*\*\*\*\*\*\*\*\*\*\*\*\*\*\*\*\*\*\*\*\*\*\*\*\*\*\*-\*\*\*..-\*\*\*\*\*\*\*\*\*\*\*\* | 8700 |
|
| S288C | 7288 | TATAATAGTAAAGATGAAATAATAATAATAATTATAAGACTAATATATGTGAAAATATTAATTAAATATTAACTGACATTGAGGGATTAAAACTAGAGTA | 7387 |
| YJM1388 | 7101 | TATAATAGTAAAGATGAAATAATAATAATAATTATAAGACTAATATATGTGAAAATATTAATTAAATATTAACTGACATTGAGGGATTAAAACTAGAGTA | 7200 |
| YJM789 | 6760 | TATAATAGTAAAGATGAAATAATAATAATAATTATAAGACTAATATATGTGAAAATATTAATTAAATATTAACTGACATTGAGGGATTAAAACTAGAGTA | 6859 |
| YJM1273 | 6863 | TATAATAGTAAAGATGAAATAATAATAATAATTATAAGACTAATATATGTGAAAATATTAATTAAATATTAACTGACATTGAGGGATTAAAACTAGAGTA | 6962 |
| NCYC3585 | 6962 | TATAATAGTAAAGATGAAATAATAATAATAATTATAAGACTAATATATGTGAAAATATTAATTAAATATTAACTGACATTGAGGGATTAAAACTAGAGTA | 7061 |
| YJM1401 | 7263 | TATAATAGTAAAGATGAAATAATAATAATAATTATAAGACTAATATATGTGAAAATATTAATTAAATATTAACTGACATTGAGGGATTAAAACTAGAGTA | 7362 |
| NCYC3594 | 7264 | TATAATAGTAAAGATGAAATAATAATAATAATTATAAGACTAATATATGTGAAAATATTAATTAAATATTAACTGACATTGAGGGATTAAAACTAGAGTA | 7363 |
| YJM1078 | 7137 | TATAATAGTAAAGATGAAATAATAATAATAATTATAAGACTAATATATGTGAAAATATTAATTAAATATTAACTGACATTGAGGGATTAAAACTAGAGTA | 7236 |
| YJM1439 | 7576 | TATAATAGTAAAGATGAAATAATAATAATAATTATAAGACTAATATATGTGAAAATATTAATTAAATATTAACTGACATTGAGGGATTAAAACTAGAGTA | 7675 |
| consensus | 8701 | \*\*\*\*\*\*\*\*\*\*\*\*\*\*\*\*\*\*\*\*\*\*\*\*\*\*\*\*\*\*\*\*\*\*\*\*\*\*\*\*\*\*\*\*\*\*\*\*\*\*\*\*\*\*\*\*\*\*\*\*\*\*\*\*\*\*\*\*\*\*\*\*\*\*\*\*\*\*\*\*\*\*\*\*\*\*\*\*\*\*\*\*\*\*\*\*\*\*\*\* | 8800 |
|
| S288C | 7388 | GCGAAACGGATTCGATACCCGTGTAGTTCTAGTAGTAAACTATGAATACAATTATTTATAATATATATTATATATAAATAATAAATGAAAATGAAAGTAT | 7487 |
| YJM1388 | 7201 | GCGAAACGGATTCGATACCCGTGTAGTTCTAGTAGTAAACTATGAATACAATTATTTATAATATATATTATATATAAATAATAAATGAAAATGAAAGTAT | 7300 |
| YJM789 | 6860 | GCGAAACGGATTCGATACCCGTGTAGTTCTAGTAGTAAACTATGAATACAATTATTTATAATATATATTATATATAAATAATAAATGAAAATGAAAGTAT | 6959 |
| YJM1273 | 6963 | GCGAAACGGATTCGATACCCGTGTAGTTCTAGTAGTAAACTATGAATACAATTATTTATAATATATATTATATATAAATAATAAATGAAAATGAAAGTAT | 7062 |
| NCYC3585 | 7062 | GCGAAACGGATTCGATACCCGTGTAGTTCTAGTAGTAAACTATGAATACAATTATTTATAATATATATTATATATAAATAATAAATGAAAATGAAAGTAT | 7161 |
| YJM1401 | 7363 | GCGAAACGGATTCGATACCCGTGTAGTTCTAGTAGTAAACTATGAATACAATTATTTATAATATATATTATATATAAATAATAAATGAAAATGAAAGTAT | 7462 |
| NCYC3594 | 7364 | GCGAAACGGATTCGATACCCGTGTAGTTCTAGTAGTAAACTATGAATACAATTATTTATAATATATATTATATATAAATAATAAATGAAAATGAAAGTAT | 7463 |
| YJM1078 | 7237 | GCGAAACGGATTCGATACCCGTGTAGTTCTAGTAGTAAACTATGAATACAATTATTTATAATATATATTATATATAAATAATAAATGAAAATGAAAGTAT | 7336 |
| YJM1439 | 7676 | GCGAAACGGATTCGATACCCGTGTAGTTCTAGTAGTAAACTATGAATACAATTATTTATAATATATATTATATATAAATAATAAATGAAAATGAAAGTAT | 7775 |
| consensus | 8801 | \*\*\*\*\*\*\*\*\*\*\*\*\*\*\*\*\*\*\*\*\*\*\*\*\*\*\*\*\*\*\*\*\*\*\*\*\*\*\*\*\*\*\*\*\*\*\*\*\*\*\*\*\*\*\*\*\*\*\*\*\*\*\*\*\*\*\*\*\*\*\*\*\*\*\*\*\*\*\*\*\*\*\*\*\*\*\*\*\*\*\*\*\*\*\*\*\*\*\*\* | 8900 |
|
| S288C | 7488 | TCCACCTGAAGAGTACGTTAGCAATAATGAAACTCAAAACAATAGACGGTTACAGACTTAAGCAGTGGAGCATGTTATTTAATTCGATAATCCACGACTA | 7587 |
| YJM1388 | 7301 | TCCACCTGAAGAGTACGTTAGCAATAATGAAACTCAAAACAATAGACGGTTACAGACTTAAGCAGTGGAGCATGTTATTTAATTCGATAATCCACGACTA | 7400 |
| YJM789 | 6960 | TCCACCTGAAGAGTACGTTAGCAATAATGAAACTCAAAACAATAGACGGTTACAGACTTAAGCAGTGGAGCATGTTATTTAATTCGATAATCCACGACTA | 7059 |
| YJM1273 | 7063 | TCCACCTGAAGAGTACGTTAGCAATAATGAAACTCAAAACAATAGACGGTTACAGACTTAAGCAGTGGAGCATGTTATTTAATTCGATAATCCACGACTA | 7162 |
| NCYC3585 | 7162 | TCCACCTGAAGAGTACGTTAGCAATAATGAAACTCAAAACAATAGACGGTTACAGACTTAAGCAGTGGAGCATGTTATTTAATTCGATAATCCACGACTA | 7261 |
| YJM1401 | 7463 | TCCACCTGAAGAGTACGTTAGCAATAATGAAACTCAAAACAATAGACGGTTACAGACTTAAGCAGTGGAGCATGTTATTTAATTCGATAATCCACGACTA | 7562 |
| NCYC3594 | 7464 | TCCACCTGAAGAGTACGTTAGCAATAATGAAACTCAAAACAATAGACGGTTACAGACTTAAGCAGTGGAGCATGTTATTTAATTCGATAATCCACGACTA | 7563 |
| YJM1078 | 7337 | TCCACCTGAAGAGTACGTTAGCAATAATGAAACTCAAAACAATAGACGGTTACAGACTTAAGCAGTGGAGCATGTTATTTAATTCGATAATCCACGACTA | 7436 |
| YJM1439 | 7776 | TCCACCTGAAGAGTACGTTAGCAATAATGAAACTCAAAACAATAGACGGTTACAGACTTAAGCAGTGGAGCATGTTATTTAATTCGATAATCCACGACTA | 7875 |
| consensus | 8901 | \*\*\*\*\*\*\*\*\*\*\*\*\*\*\*\*\*\*\*\*\*\*\*\*\*\*\*\*\*\*\*\*\*\*\*\*\*\*\*\*\*\*\*\*\*\*\*\*\*\*\*\*\*\*\*\*\*\*\*\*\*\*\*\*\*\*\*\*\*\*\*\*\*\*\*\*\*\*\*\*\*\*\*\*\*\*\*\*\*\*\*\*\*\*\*\*\*\*\*\* | 9000 |
|
| S288C | 7588 | ACCTTACCATATTTTGAATATTATAATAATTATTATAATTATTATATTACAGGCGTTACATTGTTGTCTTTAGTTCGTGCTGCAAAGTTTTAGATTAAGT | 7687 |
| YJM1388 | 7401 | ACCTTACCATATTTTGAATATTATAATAATTATTATAATTATTATATTACAGGCGTTACATTGTTGTCTTTAGTTCGTGCTGCAAAGTTTTAGATTAAGT | 7500 |
| YJM789 | 7060 | ACCTTACCATATTTTGAATATTATAATAATTATTATAATTATTATATTACAGGCGTTACATTGTTGTCTTTAGTTCGTGCTGCAAAGTTTTAGATTAAGT | 7159 |
| YJM1273 | 7163 | ACCTTACCATATTTTGAATATTATAATAATTATTATAATTATTATATTACAGGCGTTACATTGTTGTCTTTAGTTCGTGCTGCAAAGTTTTAGATTAAGT | 7262 |
| NCYC3585 | 7262 | ACCTTACCATATTTTGAATATTATAATAATTATTATAATTATTATATTACAGGCGTTACATTGTTGTCTTTAGTTCGTGCTGCAAAGTTTTAGATTAAGT | 7361 |
| YJM1401 | 7563 | ACCTTACCATATTTTGAATATTATAATAATTATTATAATTATTATATTACAGGCGTTACATTGTTGTCTTTAGTTCGTGCTGCAAAGTTTTAGATTAAGT | 7662 |
| NCYC3594 | 7564 | ACCTTACCATATTTTGAATATTATAATAATTATTATAATTATTATATTACAGGCGTTACATTGTTGTCTTTAGTTCGTGCTGCAAAGTTTTAGATTAAGT | 7663 |
| YJM1078 | 7437 | ACCTTACCATATTTTGAATATTATAATAATTATTATAATTATTATATTACAGGCGTTACATTGTTGTCTTTAGTTCGTGCTGCAAAGTTTTAGATTAAGT | 7536 |
| YJM1439 | 7876 | ACCTTACCATATTTTGAATATTATAATAATTATTATAATTATTATATTACAGGCGTTACATTGTTGTCTTTAGTTCGTGCTGCAAAGTTTTAGATTAAGT | 7975 |
| consensus | 9001 | \*\*\*\*\*\*\*\*\*\*\*\*\*\*\*\*\*\*\*\*\*\*\*\*\*\*\*\*\*\*\*\*\*\*\*\*\*\*\*\*\*\*\*\*\*\*\*\*\*\*\*\*\*\*\*\*\*\*\*\*\*\*\*\*\*\*\*\*\*\*\*\*\*\*\*\*\*\*\*\*\*\*\*\*\*\*\*\*\*\*\*\*\*\*\*\*\*\*\*\* | 9100 |
|
| S288C | 7688 | TCATAAACGAACAAAACTCCATATATATAATTTTAATTATATATAATTTTATATTATTTATTAATATAAAGAAAGGAATTAAGACAAATCATAATGATCC | 7787 |
| YJM1388 | 7501 | TCATAAACGAACAAAACTCCATATATATAATTTTAATTATATATAATTTTATATTATTTATTAATATAAAGAAAGGAATTAAGACAAATCATAATGATCC | 7600 |
| YJM789 | 7160 | TCATAAACGAACAAAACTCCATATATATAATTTTAATTATATATAATTTTATATTATTTATTAATATAAAGAAAGGAATTAAGACAAATCATAATGATCC | 7259 |
| YJM1273 | 7263 | TCATAAACGAACAAAACTCCATATATATAATTTTAATTATATATAATTTTATATTATTTATTAATATAAAGAAAGGAATTAAGACAAATCATAATGATCC | 7362 |
| NCYC3585 | 7362 | TCATAAACGAACAAAACTCCATATATATAATTTTAATTATATATAATTTTATATTATTTATTAATATAAAGAAAGGAATTAAGACAAATCATAATGATCC | 7461 |
| YJM1401 | 7663 | TCATAAACGAACAAAACTCCATATATATAATTTTAATTATATATAATTTTATATTATTTATTAATATAAAGAAAGGAATTAAGACAAATCATAATGATCC | 7762 |
| NCYC3594 | 7664 | TCATAAACGAACAAAACTCCATATATATAATTTTAATTATATATAATTTTATATTATTTATTAATATAAAGAAAGGAATTAAGACAAATCATAATGATCC | 7763 |
| YJM1078 | 7537 | TCATAAACGAACAAAACTCCATATATATAATTTTAATTATATATAATTTTATATTATTTATTAATATAAAGAAAGGAATTAAGACAAATCATAATGATCC | 7636 |
| YJM1439 | 7976 | TCATAAACGAACAAAACTCCATATATATAATTTTAATTATATATAATTTTATATTATTTATTAATATAAAGAAAGGAATTAAGACAAATCATAATGATCC | 8075 |
| consensus | 9101 | \*\*\*\*\*\*\*\*\*\*\*\*\*\*\*\*\*\*\*\*\*\*\*\*\*\*\*\*\*\*\*\*\*\*\*\*\*\*\*\*\*\*\*\*\*\*\*\*\*\*\*\*\*\*\*\*\*\*\*\*\*\*\*\*\*\*\*\*\*\*\*\*\*\*\*\*\*\*\*\*\*\*\*\*\*\*\*\*\*\*\*\*\*\*\*\*\*\*\*\* | 9200 |
|
| S288C | 7788 | TTATAATATGGGTAATAGACGTGCTATAATAAAATGATAATAAAATTATATAAAATATATTTAATTATATTTAATTAATAATATAAAACATTTTAATTTT | 7887 |
| YJM1388 | 7601 | TTATAATATGGGTAATAGACGTGCTATAATAAAATGATAATAAAATTATATAAAATATATTTAATTATATTTAATTAATAATATAAAACATTTTAATTTT | 7700 |
| YJM789 | 7260 | TTATAATATGGGTAATAGACGTGCTATAATAAAATGATAATAAAATTATATAAAATATATTTAATTATATTTAATTAATAATATAAAACATTTTAATTTT | 7359 |
| YJM1273 | 7363 | TTATAATATGGGTAATAGACGTGCTATAATAAAATGATAATAAAATTATATAAAATATATTTAATTATATTTAATTAATAATATAAAACATTTTAATTTT | 7462 |
| NCYC3585 | 7462 | TTATAATATGGGTAATAGACGTGCTATAATAAAATGATAATAAAATTATATAAAATATATTTAATTATATTTAATTAATAATATAAAACATTTTAATTTT | 7561 |
| YJM1401 | 7763 | TTATAATATGGGTAATAGACGTGCTATAATAAAATGATAATAAAATTATATAAAATATATTTAATTATATTTAATTAATAATATAAAACATTTTAATTTT | 7862 |
| NCYC3594 | 7764 | TTATAATATGGGTAATAGACGTGCTATAATAAAATGATAATAAAATTATATAAAATATATTTAATTATATTTAATTAATAATATAAAACATTTTAATTTT | 7863 |
| YJM1078 | 7637 | TTATAATATGGGTAATAGACGTGCTATAATAAAATGATAATAAAATTATATAAAATATATTTAATTATATTTAATTAATAATATAAAACATTTTAATTTT | 7736 |
| YJM1439 | 8076 | TTATAATATGGGTAATAGACGTGCTATAATAAAATGATAATAAAATTATATAAAATATATTTAATTATATTTAATTAATAATATAAAACATTTTAATTTT | 8175 |
| consensus | 9201 | \*\*\*\*\*\*\*\*\*\*\*\*\*\*\*\*\*\*\*\*\*\*\*\*\*\*\*\*\*\*\*\*\*\*\*\*\*\*\*\*\*\*\*\*\*\*\*\*\*\*\*\*\*\*\*\*\*\*\*\*\*\*\*\*\*\*\*\*\*\*\*\*\*\*\*\*\*\*\*\*\*\*\*\*\*\*\*\*\*\*\*\*\*\*\*\*\*\*\*\* | 9300 |
|
| S288C | 7888 | TAATATATTTTTTTATTATATATTAATATGAATTATAATCTGAAATTCGATTATATGAAAAAAGAATTGCTAGTAATACGTAAATTAGTATGTTACGGTG | 7987 |
| YJM1388 | 7701 | TAATATATTTTTTTATTATATATTAATATGAATTATAATCTGAAATTCGATTATATGAAAAAAGAATTGCTAGTAATACGTAAATTAGTATGTTACGGTG | 7800 |
| YJM789 | 7360 | TAATATATTTTTTTATTATATATTAATATGAATTATAATCTGAAATTCGATTATATGAAAAAAGAATTGCTAGTAATACGTAAATTAGTATGTTACGGTG | 7459 |
| YJM1273 | 7463 | TAATATATTTTTTTATTATATATTAATATGAATTATAATCTGAAATTCGATTATATGAAAAAAGAATTGCTAGTAATACGTAAATTAGTATGTTACGGTG | 7562 |
| NCYC3585 | 7562 | TAATATATTTTTTTATTATATATTAATATGAATTATAATCTGAAATTCGATTATATGAAAAAAGAATTGCTAGTAATACGTAAATTAGTATGTTACGGTG | 7661 |
| YJM1401 | 7863 | TAATATATTTTTTTATTATATATTAATATGAATTATAATCTGAAATTCGATTATATGAAAAAAGAATTGCTAGTAATACGTAAATTAGTATGTTACGGTG | 7962 |
| NCYC3594 | 7864 | TAATATATTTTTTTATTATATATTAATATGAATTATAATCTGAAATTCGATTATATGAAAAAAGAATTGCTAGTAATACGTAAATTAGTATGTTACGGTG | 7963 |
| YJM1078 | 7737 | TAATATATTTTTTTATTATATATTAATATGAATTATAATCTGAAATTCGATTATATGAAAAAAGAATTGCTAGTAATACGTAAATTAGTATGTTACGGTG | 7836 |
| YJM1439 | 8176 | TAATATATTTTTTTATTATATATTAATATGAATTATAATCTGAAATTCGATTATATGAAAAAAGAATTGCTAGTAATACGTAAATTAGTATGTTACGGTG | 8275 |
| consensus | 9301 | \*\*\*\*\*\*\*\*\*\*\*\*\*\*\*\*\*\*\*\*\*\*\*\*\*\*\*\*\*\*\*\*\*\*\*\*\*\*\*\*\*\*\*\*\*\*\*\*\*\*\*\*\*\*\*\*\*\*\*\*\*\*\*\*\*\*\*\*\*\*\*\*\*\*\*\*\*\*\*\*\*\*\*\*\*\*\*\*\*\*\*\*\*\*\*\*\*\*\*\* | 9400 |
|
| S288C | 7988 | AATATTCTAACTGTTTCGCACTAATCACTCATCACGCGTTGAAACATATTATTATCTTATTATTTATATAATATTTTTTAATAAATATTAATAATTATTA | 8087 |
| YJM1388 | 7801 | AATATTCTAACTGTTTCGCACTAATCACTCATCACGCGTTGAAACATATTATTATCTTATTATTTATATAATATTTTTTAATAAATATTAATAATTATTA | 7900 |
| YJM789 | 7460 | AATATTCTAACTGTTTCGCACTAATCACTCATCACGCGTTGAAACATATTATTATCTTATTATTTATATAATATTTTTTAATAAATATTAATAATTATTA | 7559 |
| YJM1273 | 7563 | AATATTCTAACTGTTTCGCACTAATCACTCATCACGCGTTGAAACATATTATTATCTTATTATTTATATAATATTTTTTAATAAATATTAATAATTATTA | 7662 |
| NCYC3585 | 7662 | AATATTCTAACTGTTTCGCACTAATCACTCATCACGCGTTGAAACATATTATTATCTTATTATTTATATAATATTTTTTAATAAATATTAATAATTATTA | 7761 |
| YJM1401 | 7963 | AATATTCTAACTGTTTCGCACTAATCACTCATCACGCGTTGAAACATATTATTATCTTATTATTTATATAATATTTTTTAATAAATATTAATAATTATTA | 8062 |
| NCYC3594 | 7964 | AATATTCTAACTGTTTCGCACTAATCACTCATCACGCGTTGAAACATATTATTATCTTATTATTTATATAATATTTTTTAATAAATATTAATAATTATTA | 8063 |
| YJM1078 | 7837 | AATATTCTAACTGTTTCGCACTAATCACTCATCACGCGTTGAAACATATTATTATCTTATTATTTATATAATATTTTTTAATAAATATTAATAATTATTA | 7936 |
| YJM1439 | 8276 | AATATTCTAACTGTTTCGCACTAATCACTCATCACGCGTTGAAACATATTATTATCTTATTATTTATATAATATTTTTTAATAAATATTAATAATTATTA | 8375 |
| consensus | 9401 | \*\*\*\*\*\*\*\*\*\*\*\*\*\*\*\*\*\*\*\*\*\*\*\*\*\*\*\*\*\*\*\*\*\*\*\*\*\*\*\*\*\*\*\*\*\*\*\*\*\*\*\*\*\*\*\*\*\*\*\*\*\*\*\*\*\*\*\*\*\*\*\*\*\*\*\*\*\*\*\*\*\*\*\*\*\*\*\*\*\*\*\*\*\*\*\*\*\*\*\* | 9500 |
|
| S288C | 8088 | ATTTATATTTATTTATATCAGAAATAATATGAATTAATGCGAAGTTGAAATACAGTTACCGTAGGGGAACCTGCGGTGGGCTTATAAATATCTTAAATAT | 8187 |
| YJM1388 | 7901 | ATTTATATTTATTTATATCAGAAATAATATGAATTAATGCGAAGTTGAAATACAGTTACCGTAGGGGAACCTGCGGTGGGCTTATAAATATCTTAAATAT | 8000 |
| YJM789 | 7560 | ATTTATATTTATTTATATCAGAAATAATATGAATTAATGCGAAGTTGAAATACAGTTACCGTAGGGGAACCTGCGGTGGGCTTATAAATATCTTAAATAT | 7659 |
| YJM1273 | 7663 | ATTTATATTTATTTATATCAGAAATAATATGAATTAATGCGAAGTTGAAATACAGTTACCGTAGGGGAACCTGCGGTGGGCTTATAAATATCTTAAATAT | 7762 |
| NCYC3585 | 7762 | ATTTATATTTATTTATATCAGAAATAATATGAATTAATGCGAAGTTGAAATACAGTTACCGTAGGGGAACCTGCGGTGGGCTTATAAATATCTTAAATAT | 7861 |
| YJM1401 | 8063 | ATTTATATTTATTTATATCAGAAATAATATGAATTAATGCGAAGTTGAAATACAGTTACCGTAGGGGAACCTGCGGTGGGCTTATAAATATCTTAAATAT | 8162 |
| NCYC3594 | 8064 | ATTTATATTTATTTATATCAGAAATAATATGAATTAATGCGAAGTTGAAATACAGTTACCGTAGGGGAACCTGCGGTGGGCTTATAAATATCTTAAATAT | 8163 |
| YJM1078 | 7937 | ATTTATATTTATTTATATCAGAAATAATATGAATTAATGCGAAGTTGAAATACAGTTACCGTAGGGGAACCTGCGGTGGGCTTATAAATATCTTAAATAT | 8036 |
| YJM1439 | 8376 | ATTTATATTTATTTATATCAGAAATAATATGAATTAATGCGAAGTTGAAATACAGTTACCGTAGGGGAACCTGCGGTGGGCTTATAAATATCTTAAATAT | 8475 |
| consensus | 9501 | \*\*\*\*\*\*\*\*\*\*\*\*\*\*\*\*\*\*\*\*\*\*\*\*\*\*\*\*\*\*\*\*\*\*\*\*\*\*\*\*\*\*\*\*\*\*\*\*\*\*\*\*\*\*\*\*\*\*\*\*\*\*\*\*\*\*\*\*\*\*\*\*\*\*\*\*\*\*\*\*\*\*\*\*\*\*\*\*\*\*\*\*\*\*\*\*\*\*\*\* | 9600 |
|
| S288C | 8188 | TCTTACATAAATATTAATCTAAATATTAATATA-------------------------------AATATTAATATT------------------------ | 8232 |
| YJM1388 | 8001 | TCTTACATAAATATTAATCTAAATATTAATATA-------------------------------AATATTAATATT------------------------ | 8045 |
| YJM789 | 7660 | TCTTACATAAATATTAATCTAAATATTAATATA-------------------------------AATAT------------------------------- | 7697 |
| YJM1273 | 7763 | TCTTACATAAATATTATTATAAATATAAATATAAATATTTATATAAATATTAATATAAAATATAAATAT------------------------------- | 7831 |
| NCYC3585 | 7862 | TCTTACATAAATATTAATCTAAATATTAATATA-------------------------------AATAT------------------------------- | 7899 |
| YJM1401 | 8163 | TCTTACATAAATATTAATCTAAATATTAATATA-------------------------------AATAT------------------------------- | 8200 |
| NCYC3594 | 8164 | TCTTACATAAATATTAATCTAAATATTAATATA-------------------------------AATATTAATATT------------------------ | 8208 |
| YJM1078 | 8037 | TCTTACATAAATATTAATCTAAATATTAATATA-------------------------------AATATTAATATT------------------------ | 8081 |
| YJM1439 | 8476 | TCTTACATAAATATTATTATAAATATTAATATA-------------------------------AATATTTATAGTGAACACCTTTATTTAATTATAAAG | 8544 |
| consensus | 9601 | \*\*\*\*\*\*\*\*\*\*\*\*\*\*\*\*.\*.\*\*\*\*\*\*\*.\*\*\*\*\*\*-------------------------------\*\*\*\*\*-.---.------------------------- | 9700 |
|
| S288C | 8233 | ------------------AATAGTTCCGGGGCCCGGCCACGGGAGCCGGAACCCCGAAAGGAGAAATATTAATATAAAT---ATAAATATTAATAT---- | 8307 |
| YJM1388 | 8046 | ------------------AATAGTTCCGGGGCCCGGCCACGGGAGCCGGAACCCCGAAAGGAGAAATATAAATATAAAT---AT---------------- | 8108 |
| YJM789 | 7698 | ------------------------------------------------------------------------------------AAATATTAATAGTTCC | 7713 |
| YJM1273 | 7832 | ------------------------------------------------------------------------------------AAATATTAATAGTTCC | 7847 |
| NCYC3585 | 7900 | ------------------------------------------------------------------------------------AAATATTAATAT---- | 7911 |
| YJM1401 | 8201 | ------------------------------------------------------------------------------------TAATATTAATAGTTCC | 8216 |
| NCYC3594 | 8209 | ------------------AATAGTTCCGGGGCCCGGCCACGGGAGCCGGAACCCCGAAAGGAG------------------------------------- | 8253 |
| YJM1078 | 8082 | ------------------AATAGTTCCGGGGCCCGGCCACGGGAGCCGGAACCCCGAAAGGAG------------------------------------- | 8126 |
| YJM1439 | 8545 | GTGTGAACCCCGCAAGGGAGGAGTTCCGGGGCCCGGCCACGGGAGCCGGAACCCCGAAAGGAGAAATATAAATATAAATATAATATATTTTAATA----- | 8639 |
| consensus | 9701 | -------------------..------------------------------------------------.--------------..--.------.---- | 9800 |
|
| S288C | 8308 | ------------------------------AAATATAAATATAAATATAAATATATTT-----TAATATAATATAATATAATATATAATATATTATATAA | 8372 |
| YJM1388 | 8109 | -------------------------------------------------AATATATTT-----TAATATAATATAATATAATATATAATATATTATATAA | 8154 |
| YJM789 | 7714 | GGGGCCCGGCCACGGGAGCCGGAACCCCGAAAGGAGAAATATAAATAT-AATATATTTTAATATAATATAATATAATATAATATATAATATATTATATAA | 7812 |
| YJM1273 | 7848 | GGGGCCCGGCCACGGGAGCCGGAACCCCGAAAGGAGAAATATAAATATAAATATAATATATTTTAATATAATATAATATAATATATAATATATTATATAA | 7947 |
| NCYC3585 | 7912 | ------------------------------TAATATAAATATAAATAT-AATATATTTTAATATAATATAATATAATATAATATATAATATATTATATAA | 7980 |
| YJM1401 | 8217 | GGGGCCCGGCCACGGGAGCCGGAACCCCGAAAGGAGAAATATAAATATAAATATAATATATTTTAATATAATATAATATAATATATAATATATTATATAA | 8316 |
| NCYC3594 | 8254 | ------------------------------AAATATAAATATAAATATAAATATATTT-----TAATATAATATAATATAATATATAATATATTATATAA | 8318 |
| YJM1078 | 8127 | ------------------------------AAATATAAATATAAATATAAATATATTT-----TAATATAATATAATATAATATATAATATATTATATAA | 8191 |
| YJM1439 | 8640 | ------------------------------TAATATAAATATAAATAT-AATATATTT-----TAATATAATATAATATAATATATAATATATTATATAA | 8703 |
| consensus | 9801 | ------------------------------.-..-.-------------\*\*\*\*\*\*.\*.--.-.\*\*\*\*\*\*\*\*\*\*\*\*\*\*\*\*\*\*\*\*\*\*\*\*\*\*\*\*\*\*\*\*\*\*\*\*\* | 9900 |
|
| S288C | 8373 | ATATAATATATAAATAATATAATAAAATATTTTAATATATATATAATATAATATAATTATTATTATAATTTAATATAAATTATTATTATAATTTAATATA | 8472 |
| YJM1388 | 8155 | ATATAATATATAAATAATATAATAAAATATTTTAATATATATATAATATAATATAATTATTATTATAATTTAATATAAATTATTATTATAATTTAATATA | 8254 |
| YJM789 | 7813 | ATATAATATATAAATAATATAATAAAATATTTTAATATATATATAATATAATATAATTATTATTATAATTTAATATAAATTATTATTATAATTTAATATA | 7912 |
| YJM1273 | 7948 | ATATAATATATAAATAATATAATAAAATATTTTAATATATATATAATATAATATAATTATTATTATAATTTAATATAAATTATTATTATAATTTAATATA | 8047 |
| NCYC3585 | 7981 | ATATAATATATAAATAATATAATAAAATATTTTAATATATATATAATATAATATAATTATTATTATAATTTAATATAAATTATTATTATAATTTAATATA | 8080 |
| YJM1401 | 8317 | ATATAATATATAAATAATATAATAAAATATTTTAATATATATATAATATAATATAATTATTATTATAATTTAATATAAATTATTATTATAATTTAATATA | 8416 |
| NCYC3594 | 8319 | ATATAATATATAAATAATATAATAAAATATTTTAATATATATATAATATAATATAATTATTATTATAATTTAATATAAATTATTATTATAATTTAATATA | 8418 |
| YJM1078 | 8192 | ATATAATATATAAATAATATAATAAAATATTTTAATATATATATAATATAATATAATTATTATTATAATTTAATATAAATTATTATTATAATTTAATATA | 8291 |
| YJM1439 | 8704 | ATATAATATATAAATAATATAATAAAATATTTTAATATAT-----ATATAATATAATTATTATTATAATTTAATAT-AATTATTATTATAATTTAATATA | 8797 |
| consensus | 9901 | \*\*\*\*\*\*\*\*\*\*\*\*\*\*\*\*\*\*\*\*\*\*\*\*\*\*\*\*\*\*\*\*\*\*\*\*\*\*\*\*-----\*\*\*\*\*\*\*\*\*\*\*\*\*\*\*\*\*\*\*\*\*\*\*\*\*\*\*\*\*\*\*-\*\*\*\*\*\*\*\*\*\*\*\*\*\*\*\*\*\*\*\*\*\*\* | 10000 |
|
| S288C | 8473 | ATAAATAAATAAATAATTATAATTATAATTATAATTATAATCTCAATATATAAATGATAAATTATTATAAATACAAAGGAAATAATTGATTTTTAAAATA | 8572 |
| YJM1388 | 8255 | ATAAATAAATAAATAATTATAATTATAATTATAATTATAATCTCAATATATAAATGATAAATTATTATAAATACAAAGGAAATAATTGATTTTTAAAATA | 8354 |
| YJM789 | 7913 | ATAAATAAATAAATAATTATAATTATAATTATAATTATAATCTCAATATATAAATGATAAATTATTATAAATACGAAGGAAATAATTGATTTTTAAAATA | 8012 |
| YJM1273 | 8048 | ATAAATAAATAAATAATTATAATTATAATTATAATTATAATCTCAATATATAAATGATAAATTATTATAAATACGAAGGAAATAATTGATTTTT-AAACA | 8146 |
| NCYC3585 | 8081 | ATAAATAAATAAATAATTATAATTATAATTATAATTATAATCTCAATATATAAATGATAAATTATTATAAATACGAAGAAAATAATTGATTTTT-AAACA | 8179 |
| YJM1401 | 8417 | ATAAATAAATAA------ATAATTATAATTATAATTATAATCTCAATATATAAATGATAAATTATTATAAATACAAAGGAAATAATTGATTTTTAAAATA | 8510 |
| NCYC3594 | 8419 | ATAAATAAATAAATAATTATAATTATAATTATAATTATAATCTCAATATATAAATGATAAATTATTATAAATACGAAGGAAATAATTGATTTTTAAAATA | 8518 |
| YJM1078 | 8292 | ATAAATAAATAAATAATTATAATTATAATTATAATTATAATCTCAATATATAAATGATAAATTATTATAAATACGAAGGAAATAATTGATTTTTAAAATA | 8391 |
| YJM1439 | 8798 | ATAAATAAATAAATAA--ATAATTATAATTATAATTATAATCTCAATATATAAATGATAAATTATTATAAATACGAAGGAAATAATTGATTTTT-AAACA | 8894 |
| consensus | 10001 | \*\*\*\*\*\*\*\*\*\*\*\*------\*\*\*\*\*\*\*\*\*\*\*\*\*\*\*\*\*\*\*\*\*\*\*\*\*\*\*\*\*\*\*\*\*\*\*\*\*\*\*\*\*\*\*\*\*\*\*\*\*\*\*\*\*\*\*\*.\*\*\*.\*\*\*\*\*\*\*\*\*\*\*\*\*\*\*-\*\*\*.\* | 10100 |
|
| S288C | 8573 | TATTTAATAAAATATATAATATAAATTATACTTTTTTTGTTATTATATAATAATTATATTAATATATTTAATAGAATTAAACTCCTTC------------ | 8660 |
| YJM1388 | 8355 | TATTTAATAAAATATATAATATAAATTATAC-TTTTTTGTTATTATATAATAATTATATTAATATATTTAATAGAATTAAATTCCTTCGACCGGACTCCT | 8453 |
| YJM789 | 8013 | TATTTAATAAAATATATAATATAAATTATAC-TTTTTTGTTATTATATAATAATTATATTAATATATTTAATAGAATTAAACTCCTTC------------ | 8099 |
| YJM1273 | 8147 | TATTTAATAAAATATATAATATAAATTATAC-TTTTTTGTTATTATATAATAATTATATTAATATATTTAATAGAATTAAACTCCTTC------------ | 8233 |
| NCYC3585 | 8180 | TATTTAATAAAATATATAATATAAATTATAC-TTTTTTGTTATTATATAATAATTATATTAATATATTTAATAGAATTAAACTCCTTC------------ | 8266 |
| YJM1401 | 8511 | TATTTAATAAAATATATAATATAAATTATAC-TTTTTTGTTATTATATAATAATTATATTAATATATTTAATAGAATTAAATTCCTTCGACCGGACTCCT | 8609 |
| NCYC3594 | 8519 | TATTTAATAAAATATATAATATAAATTATAC--TTTTTGTTATTATATAATAATTATATTAATATATTTAATAGA------------------------- | 8591 |
| YJM1078 | 8392 | TATTTAATAAAATATATAATATAAATTATAC--TTTTTGTTATTATATAATAATTATATTAATATATTTAATAGA------------------------- | 8464 |
| YJM1439 | 8895 | TATTTAATAAAATATATAATATAAATTATAC-TTTTTTGTTATTATATAATAATTATATTAATATATTTAATAGAATTAAACTCCTTCGACCGGACTCCT | 8993 |
| consensus | 10101 | \*\*\*\*\*\*\*\*\*\*\*\*\*\*\*\*\*\*\*\*\*\*\*\*\*\*\*\*\*\*\*--\*\*\*\*\*\*\*\*\*\*\*\*\*\*\*\*\*\*\*\*\*\*\*\*\*\*\*\*\*\*\*\*\*\*\*\*\*\*\*\*\*\*------.------------------ | 10200 |
|
| S288C | 8661 | --------------------------------------------------------------------------------------GG------------ | 8662 |
| YJM1388 | 8454 | TCTTTAAAAGGGGTTCGGTCCCCCTCCCATTAGTATAGAGTATAGGGAGGGGTCCCTCACTCCTTCGGGGTCCGCCCCGCGGGGCGGG------------ | 8541 |
| YJM789 | 8100 | --------------------------------------------------------------------------------------GG---CCGGACTCC | 8110 |
| YJM1273 | 8234 | --------------------------------------------------------------------------------------GGGGTTCGGTCCCC | 8247 |
| NCYC3585 | 8267 | --------------------------------------------------------------------------------------GGGGTTCGGTCCCC | 8280 |
| YJM1401 | 8610 | TCTTTAAA-----------------------------------------------------------------------------AGGGGTTCGGTCCCC | 8632 |
| NCYC3594 | 8592 | ---------------------------------------------------------------------------------------------------- | 8591 |
| YJM1078 | 8465 | ---------------------------------------------------------------------------------------------------- | 8464 |
| YJM1439 | 8994 | TC-------------------------------------------------------------------------------------------------- | 8995 |
| consensus | 10201 | -------------------------------------------------------------------------------------.-----.---.-.-- | 10300 |
|
| S288C | 8663 | ----------------------------------------------------------------CCGGACTATTATTCATTTTATATATTAATGATAAAT | 8698 |
| YJM1388 | 8542 | ----------------------------------------------------------------CCGGACTATTATTCATTTTATATATTAATGATAAAT | 8577 |
| YJM789 | 8111 | TTC----------------------GGGGTCCGCCCCGC-----------------GGGGCGGGCCGGACTATTATTCATTTTATATATTAATGATAAAT | 8171 |
| YJM1273 | 8248 | ACG----------------------GGGGTCCCTCACTCCTTCTTAAATTAAAAAGGGGTTCGGCCGGACTATTATTCATTTTATATATTAATGATAAAT | 8325 |
| NCYC3585 | 8281 | CTCCCGT------------TAGGGAGGGGTC--CCTCAC-----------------TCCTTCGACCGGACTATTATTCATTTTATATATTAATGATAAAT | 8349 |
| YJM1401 | 8633 | CTCCCATTAGTATAGAGTATAGGGAGGGGTC--CCTCAC-----------------TCCTTCGACCGGACTATTATTCATTTTATATATTAATGATAAAT | 8713 |
| NCYC3594 | 8592 | ---------------------------------------------------------------------------------------------------- | 8591 |
| YJM1078 | 8465 | ---------------------------------------------------------------------------------------------------- | 8464 |
| YJM1439 | 8996 | -------------------------GGGGTACGCCCCGC----------------GGGGGCGGGCCGGACTATTATTCATTTTATATATTAATGATAAAT | 9054 |
| consensus | 10301 | ...--.------------------------.-..-.-.------------------......-.------------------------------------ | 10400 |
|
| S288C | 8699 | CATTAATTATTATTAATAAATTTATTTATAATATTTAATTTTATATATTATTATTTATAAT--AAAAAAAATTATATTATAACAA------TTTAATTTT | 8790 |
| YJM1388 | 8578 | CATTAATTATTATTAATAAATTTATTTATAATATTTAATTTTATATATTATTATTTATAATAAAAAAAAAATTATATTATAACAA------TTTAATTTT | 8671 |
| YJM789 | 8172 | CATTAATTATTATTAATAAATTTATTTATAATATTTAATTTTATATATTATTATTTATAAT--AAAAAAAATTATATTATAACAATTTAATTTTAATTTT | 8269 |
| YJM1273 | 8326 | CATTAATTATTATTAATAAATTTATTTATAATATTTAATTTTATATATTATTATTTATAAT---AAAAAAATTATATTATAACAA------TTTAATTTT | 8416 |
| NCYC3585 | 8350 | CATTAATTATTATTAATAAATTTATTTATAATATTTAATTTTATATATTATTATTTATAAT--AAAAAAAATTATATTATAACAA------TTTAATTTT | 8441 |
| YJM1401 | 8714 | CATTAATTATTATTAATAAATTTATTTATAATATTTAATTTTATATATTATTATTTATAAT---AAAAAAATTATATTATAACAA------TTTAATTTT | 8804 |
| NCYC3594 | 8592 | ---------------------------------------------------------------------------------------------------- | 8591 |
| YJM1078 | 8465 | ---------------------------------------------------------------------------------------------------- | 8464 |
| YJM1439 | 9055 | CATTAATTATTATTAATAAATTTATTTATAATATTTAATTTTATATATTATTATTTATAAT--AAAAAAAATTATATTATAACAA------TTTAATTTT | 9146 |
| consensus | 10401 | ---------------------------------------------------------------------------------------------------- | 10500 |
|
| S288C | 8791 | AATTTTTATTTTTAAATTATAAAATTAATAATTTATTTGTTTAAATAAAATTTATAACTCCTTC------------GGGGTTC----------------- | 8861 |
| YJM1388 | 8672 | AATTTTTATTTTTAAATTATAAAATTAATAATTTATTTATTTATATAATAATTTTAACTCCTTC-----TTTAAAAGGGGTTCGGTCCCCCTCCCATTAG | 8766 |
| YJM789 | 8270 | AATTTTTATTTTTAAATTATAAAATTAATAATTTATTTGTTTAAATAAAATTTATAACTCCTTC------------GGGGTTCGGTCCCCCTCCC----- | 8352 |
| YJM1273 | 8417 | AATTTTTATTTTTAAATTATAAAATTAATAATTTATTTATTTATATAATAATTTAAACTCCTTC------------GGGGTTCGGTCCCC---------- | 8494 |
| NCYC3585 | 8442 | AATTTTTATTTTTAAATTATAAAATTAATAATTTATTTATTTATATAATAATTTAAACTCCTTC------------GGGGTTCGGTCCCCAC-------- | 8521 |
| YJM1401 | 8805 | AATTTTTATTTTTAAATTATAAAATTAATAATTTATTTATTTATATAATAATTTAAACTCCTTCTTAAATTAAATAGGGGTTCGGTCCCCCTCCC----- | 8899 |
| NCYC3594 | 8592 | ---------------------------------------------------------------------------------------------------- | 8591 |
| YJM1078 | 8465 | ---------------------------------------------------------------------------------------------------- | 8464 |
| YJM1439 | 9147 | AATTTTTATTTTTAAATTATAAAATTAATAATTTATTTATTTATATAATAATTTTAACTCCTTC------------GGGGTTCGGTCCCCCTCCCATTAG | 9234 |
| consensus | 10501 | --------------------------------------.----.----.-.--..----------------.--.---------------..-------- | 10600 |
|
| S288C | 8862 | ------------------------------------------------------------------------------------GGCCGGACTATTAATA | 8877 |
| YJM1388 | 8767 | TATAGAGTATA--------------GGGAGGGGTCCCTCACTCCTTT-----------------GGGGTCCGGCCCGCGGGGCGGGCCGAACTATTAATA | 8835 |
| YJM789 | 8353 | -----------AT-----TAGTATAGGGAGGGGTCCTTCACTCCTTT-----------------GGGGTCCGGCCCGCGGGGCGGGCCGAACTATTAATA | 8419 |
| YJM1273 | 8495 | --------------------------ACGGGGGTCCCTCACTCCTTT-----------------GGGGTCCGGCCCGCCCCGCGGGCCGAACTATTAATA | 8551 |
| NCYC3585 | 8522 | ----------------------------GGGGGTCCCTCACTCCTTT-----------------GGGGTCCGGCCCGCGGGGCGGGCCGAACTATTAATA | 8576 |
| YJM1401 | 8900 | -----------ATTAGTATAGTATAGGGAGGGGTCCCTCACTCCTTT-----------------GGGGTCCGGCCCGCGGGGCGGGCCGAACTATTAATA | 8971 |
| NCYC3594 | 8592 | ---------------------------------------------------------------------------------------------ATTAATA | 8598 |
| YJM1078 | 8465 | ---------------------------------------------------------------------------------------------ATTAATA | 8471 |
| YJM1439 | 9235 | TATA------------------GGGAGGGGGTCCCTCACTCCTTCTTAAAAAGGGGTACGGTTC----------CCGCGGGGCGGGCCGGACTATTAATA | 9306 |
| consensus | 10601 | ----------------------.......--...-...-.-....---------------------------------...--------.---\*\*\*\*\*\*\* | 10700 |
|
| S288C | 8878 | TAAATAAATAATAAATATTTATAATAAAATAATATACATCTTCTTTAAATA-AAAAAAGGGGACATTATAAATAGTATATAAATATATTATATCTTTTTT | 8976 |
| YJM1388 | 8836 | TAAATAAATAATAAATATTTATAATAAAATAATATACATCTTCTTTAAAT--AAATAAGGGGACATTATAAATAGTATATAAATATATTATATCTTTTTT | 8933 |
| YJM789 | 8420 | TAAATAAATAATAAATATTTATAATAAAATAATATACATCTTCTTTAAAT--AAATAAGGGGACATTATAAATAGTATATAAATATATTATATCTTTTTT | 8517 |
| YJM1273 | 8552 | TAAATAAATAATAAATATTTATAATAAAATAATATACATCTTCTTTAAATA-AAAAAAGGGGACATTATAAATAGTATATAAATATATTATATCTTTTTT | 8650 |
| NCYC3585 | 8577 | TAAATAAATAATAAATATTTATAATAAAATAATATACATCTTCTTTAAATA-AAAAAAGGGGACATTATAAATAGTATATAAATATATTATATCTTTTTT | 8675 |
| YJM1401 | 8972 | TAAATAAATAATAAATATTTATAAT-AAATAATATACATTTTCTTTAAAT--AAATAAGGGAACATTATAAATAGTATATAAATATATTATATCTTTTTT | 9068 |
| NCYC3594 | 8599 | TAAATAAATAATAAATATTTATAATAAAATAATATACATCTTCTTTAAATAAAAAAAAGGGGACATTATAAATAGTATATAAATATATTATATCTTTTTT | 8698 |
| YJM1078 | 8472 | TAAATAAATAATAAATATTTATAATAAAATAATATACATCTTCTTTAAATAAAAAAAAGGGGACATTATAAATAGTATATAAATATATTATATCTTTTTT | 8571 |
| YJM1439 | 9307 | TAAATAAATAATAAATATTTATAATAAAATAATATACATCTTCTTTAAAT--AAATAAGGGGACATTATAAATAGTATATAAATATATTATATCTTTTTT | 9404 |
| consensus | 10701 | \*\*\*\*\*\*\*\*\*\*\*\*\*\*\*\*\*\*\*\*\*\*\*\*\*-\*\*\*\*\*\*\*\*\*\*\*\*\*.\*\*\*\*\*\*\*\*\*\*--\*\*\*.\*\*\*\*\*.\*\*\*\*\*\*\*\*\*\*\*\*\*\*\*\*\*\*\*\*\*\*\*\*\*\*\*\*\*\*\*\*\*\*\*\*\*\* | 10800 |
|
| S288C | 8977 | ATTATTATTATTAATAAATAATAATAATAATTTATATATTTATAATATATTTAATAGTTCCGGGGCCCGGCCACGGGAGCCGGAACCCCGAAAGGAGAAT | 9076 |
| YJM1388 | 8934 | ATTATTATTATTAATAAATAATAATAATAATTTATATATTTATAATATATTTAATAGTTCCGGGGCCCGGCCACGGGAGCCGGAACCCCGAAAGGAGAAT | 9033 |
| YJM789 | 8518 | ATTATTATTATTAATAAATAATAATAATAATTTATATATTTATAATATATTTAAT----------------------------------------AGAAT | 8577 |
| YJM1273 | 8651 | ATTATTATTATTAATAAATAATAATAATAATTTATATATTTATAATATATTTAAT----------------------------------------AGAAT | 8710 |
| NCYC3585 | 8676 | ATTATTATTATTAATAAATAATAATAATAATTTATATATTTATAATATATTTAAT----------------------------------------AGAAT | 8735 |
| YJM1401 | 9069 | ATTATTATTATTAATAAATAATAATAATAATTTATATATTTATAATATATTTAATAGTTCCGGGGCCCGGCCACGGGAGCCGGAACCCCGAAAGGAGAAT | 9168 |
| NCYC3594 | 8699 | ATTATTATTATTAATAAATAATAATAATAATTTATATATTTATAATATATTTAATAGTTCCGGGGCCCGGCCACGGGAGCCGGAACCCCGAAAGGAGAAT | 8798 |
| YJM1078 | 8572 | ATTATTATTATTAATAAATAATAATAATAATTTATATATTTATAATATATTTAATAGTTCCGGGGCCCGGCCACGGGAGCCGGAACCCCGAAAGGAGAAT | 8671 |
| YJM1439 | 9405 | ATTATTATTATTAATAAATAATAATAATAATTTATATATTTATAATATATTTAAT----------------------------------------AGAAT | 9464 |
| consensus | 10801 | \*\*\*\*\*\*\*\*\*\*\*\*\*\*\*\*\*\*\*\*\*\*\*\*\*\*\*\*\*\*\*\*\*\*\*\*\*\*\*\*\*\*\*\*\*\*\*\*\*\*\*\*\*\*\*----------------------------------------\*\*\*\*\* | 10900 |
|
| S288C | 9077 | GTATTATAATTATTACATATAATTATTATTATTCACTTCTTATTAAAAATAAT----------------------------------------------- | 9129 |
| YJM1388 | 9034 | GTATTATAATTATTACATATAATTATTATTATTCACTTCTTATTAAAAATAAT----------------------------------------------- | 9086 |
| YJM789 | 8578 | GTATTATAATTATTACATATAATTATTATTATTCACTTCTTATTAAAAATAAT----------------------------------------------- | 8630 |
| YJM1273 | 8711 | GTATTATAATTATTACATATAATTATTATTATTCACTTCTTATTAAAAATAAT----------------------------------------------- | 8763 |
| NCYC3585 | 8736 | GTATTATAATTATTACATATAATTATTATTATTCACTTCTTATTAAAAATAAT----------------------------------------------- | 8788 |
| YJM1401 | 9169 | GTATTATAATTATTACATATAATTATTATTATTCACTTCTTATTAAAAATAAT----------------------------------------------- | 9221 |
| NCYC3594 | 8799 | GTATTATAATTATTACATATAATTATTATTATTCACTTCTTATTAAAAATAATACTCCTTCTTAAAAAGGGGTTCGGTCCCCCTCCCATTAGTATAATAT | 8898 |
| YJM1078 | 8672 | GTATTATAATTATTACATATAATTATTATTATTCACTTCTTATTAAAAATAATACTCCTTCTTAAAAAGGGGTTCGGTCCCCCTCCCATTAGTATAATAT | 8771 |
| YJM1439 | 9465 | GTATTATAATTATTACATATAATTATTATTATTCACTTCTTATTAAAAATAAT----------------------------------------------- | 9517 |
| consensus | 10901 | \*\*\*\*\*\*\*\*\*\*\*\*\*\*\*\*\*\*\*\*\*\*\*\*\*\*\*\*\*\*\*\*\*\*\*\*\*\*\*\*\*\*\*\*\*\*\*\*\*\*\*\*\*----------------------------------------------- | 11000 |
|
| S288C | 9130 | ---------------------------------------------------------------------------------------------------- | 9129 |
| YJM1388 | 9087 | ---------------------------------------------------------------------------------------------------- | 9086 |
| YJM789 | 8631 | ---------------------------------------------------------------------------------------------------- | 8630 |
| YJM1273 | 8764 | ---------------------------------------------------------------------------------------------------- | 8763 |
| NCYC3585 | 8789 | ---------------------------------------------------------------------------------------------------- | 8788 |
| YJM1401 | 9222 | ---------------------------------------------------------------------------------------------------- | 9221 |
| NCYC3594 | 8899 | ACATTAGTATAATATACATTAGTATAATATACATTAGTATAATATACATTAGTATAATATACATTAGTATAATATACATTAGTATAATATACGGGAGGGG | 8998 |
| YJM1078 | 8772 | ACATTAGTATAATATACATTAGTATAATATACATTAGTATAATATACATTAGTATAATATACATTAGTATAATATACATTAGTATAATATACGGGAGGGG | 8871 |
| YJM1439 | 9518 | ---------------------------------------------------------------------------------------------------- | 9517 |
| consensus | 11001 | ---------------------------------------------------------------------------------------------------- | 11100 |
|
| S288C | 9130 | ------------------------------------ACTCTATATAATTTATATAATTTATTTTAATATATATATATTTATATATAATATAATATATATA | 9193 |
| YJM1388 | 9087 | ------------------------------------ACTCTATATAATTTATATAATTTATTTTAATATATATATATTTATAT-----ATAATATATATA | 9145 |
| YJM789 | 8631 | ------------------------------------ACTCTATATAATTTATATAATTTATTTTAATATATATATATTTATATATAATATAATATATATA | 8694 |
| YJM1273 | 8764 | ------------------------------------ACTCTATATAATTTATATAATTTATTTTAATATATATATATTTATATATAATATAATATATATA | 8827 |
| NCYC3585 | 8789 | ------------------------------------ACTCTATATAATTTATATAATTTATTTTAATATATATATATTTATATATAATATAATATATATA | 8852 |
| YJM1401 | 9222 | ------------------------------------ACTCTATATAATTTATATAATTTATTTTAATATATATATATTTATATATAATATAATATATATA | 9285 |
| NCYC3594 | 8999 | TCCCTCACTCCTTCGGGGTCCCCGCTGGGGGCGGAGACTCTATATAATTTATATAATTTATTTTAATATATATATATTTATATATAATATAATATATATA | 9098 |
| YJM1078 | 8872 | TCCCTCACTCCTTCGGGGTCCCCGCTGGGGGCGGAGACTCTATATAATTTATATAATTTATTTTAATATATATATATTTATATATAATATAATATATATA | 8971 |
| YJM1439 | 9518 | ------ACTCCTTCGGGGTCCCCACTGGGGGTGGGGACTCTATATAATTTATATAATTTATTTTAATATATATATATTTATATATAATATAATATATATA | 9611 |
| consensus | 11101 | -----------------------.-------.--.-\*\*\*\*\*\*\*\*\*\*\*\*\*\*\*\*\*\*\*\*\*\*\*\*\*\*\*\*\*\*\*\*\*\*\*\*\*\*\*\*\*\*\*\*\*\*\*-----\*\*\*\*\*\*\*\*\*\*\*\* | 11200 |
|
| S288C | 9194 | TTTATTTATTATAATCATTTTTTTTTAACTTAAAATAAAACTTATTATAATTTATATAATTTATAATTTTTATAT------AAAAATAATTATATAA-TT | 9286 |
| YJM1388 | 9146 | TTTATTTATTATAATCATTTTTTTT--ACTTAAAATAAAACTTATTATAATAAATATAATTTATAATTTTTATAT------AAAAAGAATTATATAA-TT | 9236 |
| YJM789 | 8695 | TTTATTTATTATAATCATTTTTTTTT-ACTTAAAATAAAACTTATTATAATTTATATAATTTATAATTTTTATATA---AAAAAAATAATTATATAATTT | 8790 |
| YJM1273 | 8828 | TTTATTTATTATAATCATTTATTTTTAACTTAAAATAAAACTTATTATAATTTATATAATTTATAATTTTTATAT------AAAAATAATTATATAA-TT | 8920 |
| NCYC3585 | 8853 | TTTATTTATTATAATCATTTTTTTTTAACTTAAAATAAAACTTATTATAATTTATATAATTTATAATTTTTATAT------AAAAATAATTATATAATTT | 8946 |
| YJM1401 | 9286 | TTTATTTATTATAATCATTTATTTTTAACTTAAAATAAAACTTATTATAATTTATATAATTTATAATTTTTCTCCTTTCTTAAAAATAATTATATAA-TT | 9384 |
| NCYC3594 | 9099 | TTTATTTATTATAATCATTTTTTTTTA-CTTAAAATAAAACTTATTATAATTTATATAATTTATAATTTTTATATA---AAAAAAATAATTATATAA-TT | 9193 |
| YJM1078 | 8972 | TTTATTTATTATAATCATTTTTTTTTA-CTTAAAATAAAACTTATTATAATTTATATAATTTATAATTTTTATATA---AAAAAAATAATTATATAATTT | 9067 |
| YJM1439 | 9612 | TTTATTTATTATAATCATTTTTTTTTTACTTAAAATAAAACTTATTATAATTTATATAATTTATAATTTTTATATA---AAAAAAATAATTATATAA-TT | 9707 |
| consensus | 11201 | \*\*\*\*\*\*\*\*\*\*\*\*\*\*\*\*\*\*\*\*.\*\*\*\*-.-\*\*\*\*\*\*\*\*\*\*\*\*\*\*\*\*\*\*\*\*\*\*\*..\*\*\*\*\*\*\*\*\*\*\*\*\*\*\*\*\*\*.\*...---..\*\*\*\*\*.\*\*\*\*\*\*\*\*\*\*-\*\* | 11300 |
|
| S288C | 9287 | TTTATTTATTTATATAATAATAATATTATTTGTTATATATTATATATTATATATATAATAAATAAATAAATAATAAATAATAATAATAAGGATATAGTTT | 9386 |
| YJM1388 | 9237 | T---ATTATTTATATAATAATAATATTATTTGTTATATAATATATATTATATATATAAT-AATAAATAAATAAT-AATAATAATAATAAGGATATAGTTT | 9331 |
| YJM789 | 8791 | TTTTTTTATTTATATAATAATAATATTATTTG-------TTATATATTATATATATAAT-AATAAATAAATAAT-AATAATAATAATAAGGATATAGTTT | 8881 |
| YJM1273 | 8921 | TTTTTTTATTTATATAATAATAATATTATTTG-------TTATATATTATATATATAAT-AATAAATAAATAAT-AATAATAATAATAAGGATATAGTTT | 9011 |
| NCYC3585 | 8947 | TTTTTTTATTTATATAATAATAATATTATTTGTTATATATTATATATTATATATATAATAAATAAATAAATAATAAATAATAATAATAAGGATATAGTTT | 9046 |
| YJM1401 | 9385 | TTTTTTTATTTATATAATAATAATATTATTTGTTATATATTATATATTATATATATAATAAATAAATAAATAATAAATAATAATAATAAGGATATAGTTT | 9484 |
| NCYC3594 | 9194 | TTTTTTTATTTATATAATAATAATATTATTTGT-------TATATATTATATATATAATA-ATAAATAAATAATA-ATAATAATAATAAGGATATAGTTT | 9284 |
| YJM1078 | 9068 | TTTTTTTATTTATATAATAATAATATTATTTGT-------TATATATTATATATATAATA-ATAAATAAATAATA-ATAATAATAATAAGGATATAGTTT | 9158 |
| YJM1439 | 9708 | TTTTTTTATTTATATAATAATAATATTATTTGT-------TATATATTATATATATAATA-ATAAATAAATAATA-ATAATAATAATAAGGATATAGTTT | 9798 |
| consensus | 11301 | \*--..\*\*\*\*\*\*\*\*\*\*\*\*\*\*\*\*\*\*\*\*\*\*\*\*\*\*\*-------.\*\*\*\*\*\*\*\*\*\*\*\*\*\*\*\*\*\*\*--\*\*\*\*\*\*\*\*\*\*\*\*\*--\*\*\*\*\*\*\*\*\*\*\*\*\*\*\*\*\*\*\*\*\*\*\*\* | 11400 |
|
| S288C | 9387 | AATGGTAAAACAGTTGATTTCAAATCAATCATTAGGAGTTCGAATCTCTTTATCCTTGATAATAATAATAAAAATATGTATTTATTTAATTATTTTAATA | 9486 |
| YJM1388 | 9332 | AATGGTAAAACAGTTGATTTCAAATCAATCATTAGGAGTTCGAATCTCTTTATCCTTGATAATAATAATAAAAATATGTATTTATTTAATTATTTTAATA | 9431 |
| YJM789 | 8882 | AATGGTAAAACAGTTGATTTCAAATCAATCATTAGGAGTTCGAATCTCTTTATCCTTGATAATAATAATAAAAATATGTATTTATTTAATTATTTTAATA | 8981 |
| YJM1273 | 9012 | AATGGTAAAACAGTTGATTTCAAATCAATCATTAGGAGTTCGAATCTCTTTATCCTTGATAATAATAATAAAAATATGTATTTATTTAATTATTTTAATA | 9111 |
| NCYC3585 | 9047 | AATGGTAAAACAGTTGATTTCAAATCAATCATTAGGAGTTCGAATCTCTTTATCCTTGATAATAATAATAAAAATATGTATTTATTTAATTATTTTAATA | 9146 |
| YJM1401 | 9485 | AATGGTAAAACAGTTGATTTCAAATCAATCATTAGGAGTTCGAATCTCTTTATCCTTGATAATAATAATAAAAATATGTATTTATTTAATTATTTTAATA | 9584 |
| NCYC3594 | 9285 | AATGGTAAAACAGTTGATTTCAAATCAATCATTAGGAGTTCGAATCTCTTTATCCTTGATAATAATAATAAAAATATGTATTTATTTAATTATTTTAATA | 9384 |
| YJM1078 | 9159 | AATGGTAAAACAGTTGATTTCAAATCAATCATTAGGAGTTCGAATCTCTTTATCCTTGATAATAATAATAAAAATATGTATTTATTTAATTATTTTTATA | 9258 |
| YJM1439 | 9799 | AATGGTAAAACAGTTGATTTCAAATCAATCATTAGGAGTTCGAATCTCTTTATCCTTGATAATAAT------AATATGTATTTATTTAATTATTTTAATA | 9892 |
| consensus | 11401 | \*\*\*\*\*\*\*\*\*\*\*\*\*\*\*\*\*\*\*\*\*\*\*\*\*\*\*\*\*\*\*\*\*\*\*\*\*\*\*\*\*\*\*\*\*\*\*\*\*\*\*\*\*\*\*\*\*\*\*\*\*\*\*\*\*\*------\*\*\*\*\*\*\*\*\*\*\*\*\*\*\*\*\*\*\*\*\*\*\*\*.\*\*\* | 11500 |
|
| S288C | 9487 | TTTCTCCT-----------------------------------------TTCGGGGTTCCGGCTCCC--------------GTGGCCGGGCCCCGGAACT | 9531 |
| YJM1388 | 9432 | TTTCTCCT-----------------------------------------TTCGGGGTTCCGGCTCCC--------------GTGGCCGGGCCCCGGAACT | 9476 |
| YJM789 | 8982 | TTTCTCCT-----------------------------------------TTCGGGGTTCCGGCTCCC--------------GTGGCCGGCCCCCGGAACT | 9026 |
| YJM1273 | 9112 | TTTCTCCT-----------------------------------------TTCGGGGTTCCGGCTCCC--------------GTGGCCGGCCCCCGGAACT | 9156 |
| NCYC3585 | 9147 | TTTCTCCT-----------------------------------------TTCGGGGTTCCGGCTCCC--------------GTGGCCGGGCCCCGGAACT | 9191 |
| YJM1401 | 9585 | TTTCTCCT-----------------------------------------TTCGGGGTTCCGGCTCCC--------------GTGGCCGGGCCCCGGAACT | 9629 |
| NCYC3594 | 9385 | TTTCTCCT-----------------------------------------TTCGGGGTTCCGGCTCCC--------------GTGGCCGGGCCCCGGAACT | 9429 |
| YJM1078 | 9259 | TTTCTCCT-----------------------------------------TTCGGGGTTCCGGCTCCC--------------GTGGCCGGGCCCCGGAACT | 9303 |
| YJM1439 | 9893 | TTTCTCCTTCTTAAAAAGGGGTTCGGTCCCCCACGGGGTCCCTCACTCCTTCGGGGTTCGGTCCCCCTCCCGTTAGGGAGGGGGTCCCTCACTCCTTTCT | 9992 |
| consensus | 11501 | \*\*\*\*\*\*\*\*-----------------------------------------\*\*\*\*\*\*\*\*\*\*.\*.\*.\*\*\*--------------\*.\*.\*\*....\*.\*....\*\* | 11600 |
|
| S288C | 9532 | ATTAATATAATATAATATAATATAAATATTCATTTATC-TTTTTTTTAATATTCTTAATTAATTAATTAATTAATATATTAATTAT-AAAAAATATATTA | 9629 |
| YJM1388 | 9477 | AT-----TAATATAATATAATATAAATATTCATTTATCTTTTTTTTTAATATTCTTAATTAATTAATTAATTAATATATTAATTATAAAAAAATATATTA | 9571 |
| YJM789 | 9027 | AT-----TAATATAATATAATATAAATATTCATTTATCTTTTTTTTTAATATTCT----TAATTAATTAATTAATATATTAATTAT-AAAAAATATATTA | 9116 |
| YJM1273 | 9157 | AT-----TAATATAATATAATCTAAATATTCATTTATCTTTTTTTTTAATATTCT----TAATTAATTAATTAATATATTAATTAT-AAAAAATATATTA | 9246 |
| NCYC3585 | 9192 | AT-----TAATATAATATAATATAAATATTCATTTATCTTTTTTTTTAATATTC--------TTAATTAATTAATATATTAATTAT-AAAAAATATATTA | 9277 |
| YJM1401 | 9630 | AT-----TAATATAATATAATATAAATATTCATTTATC-TTTTTTTTAATATTCTTAATTAATTAATTAATTAATATATTAATTAT-AAAAAATATATTA | 9722 |
| NCYC3594 | 9430 | AT----------TAATATAATATAAATATTCATTTATCTTTTTTTTTTATATTCTTAATTAATTAATTAATTAATATATTAATTAT-AAAAAATATATTA | 9518 |
| YJM1078 | 9304 | AT----------TAATATAATATAAATATTCATTTATCTTTTTTTTTTATATTCTTAATTAATTAATTAATTAATATATTAATTAT-AAAAAATATATTA | 9392 |
| YJM1439 | 9993 | ATTAATATAATATAATATAATATAAATATTCATTTATC-TTTTTTTTAATATTCTTAATTAATTAATTAATTAATATATTAATTAT-AAAAAATATATTA | 10090 |
| consensus | 11601 | \*\*----------\*\*\*\*\*\*\*\*\*.\*\*\*\*\*\*\*\*\*\*\*\*\*\*\*\*-\*\*\*\*\*\*\*\*.\*\*\*\*\*\*--------\*\*\*\*\*\*\*\*\*\*\*\*\*\*\*\*\*\*\*\*\*\*\*\*-\*\*\*\*\*\*\*\*\*\*\*\*\* | 11700 |
|
| S288C | 9630 | TAATTTTATTATTAATAAGTATAAATATATTATTAATAATAATTTATTAAAAATATATTATTATAATATATTAATATATCATAATTATAATCAATATTAT | 9729 |
| YJM1388 | 9572 | TAATTTTATTATTAATAAGTATAAATATATTATTAATAATAATTTATTAAAAATATATTATTATAATATATTAATATATCATAATTATAATCAATATTAT | 9671 |
| YJM789 | 9117 | TAATTTTATTATTAATAAGTATAAATATATTATTAATAATAATTTATTAAAAATATATTATTATAATATATTAATATATCATAATTATAATCAATATTAT | 9216 |
| YJM1273 | 9247 | TAATTTTATTATTAATAAGTATAAATATATTATTAATAATAATTTATTAAAAATATATTATTATAATATATTAATATATCATAATTATAATCAATATTAT | 9346 |
| NCYC3585 | 9278 | TAATTTTATTATTAATAAGTATAAATATATTATTAATAATAATTTATTAAAAATATATTATTATAATATATTAATATATCATAATTATAATCAATATTAT | 9377 |
| YJM1401 | 9723 | TAATTTTATTATTAATAAGTATAAATATATTATTAATAATAATTTATTAAAAATATATTATTATAATATATTAATATATCATAATTATAATCAATATTAT | 9822 |
| NCYC3594 | 9519 | TAATTTTATTATTAATAAGTATAAATATATTATTAATAATAATTTATTAAAAATATATTATTATAATATATTAATATATCATAATTATAATCAATATTAT | 9618 |
| YJM1078 | 9393 | TAATTTTATTATTAATAAGTATAAATATATTATTAATAATAATTTATTAAAAATATATTATTATAATATATTAATATATCATAATTATAATCAATATTAT | 9492 |
| YJM1439 | 10091 | TAATTTTATTATTAATAAGTATAAATATATTATTAATAATAATTTATTAATAATATATTATTATAATATATTAATATATCATAATTATAATCAATATTAT | 10190 |
| consensus | 11701 | \*\*\*\*\*\*\*\*\*\*\*\*\*\*\*\*\*\*\*\*\*\*\*\*\*\*\*\*\*\*\*\*\*\*\*\*\*\*\*\*\*\*\*\*\*\*\*\*\*\*.\*\*\*\*\*\*\*\*\*\*\*\*\*\*\*\*\*\*\*\*\*\*\*\*\*\*\*\*\*\*\*\*\*\*\*\*\*\*\*\*\*\*\*\*\*\*\*\*\* | 11800 |
|
| S288C | 9730 | ATTATTTAATTTTATAATACT----------------------------------TAATTATTAATATATTATTC--ATATATATATAAATTAAATTAAA | 9793 |
| YJM1388 | 9672 | ATTATTTAATTTTATAATACT----------------------------------TAATTATTAATATATTATTC--ATATATATATAAATTAAATTAAA | 9735 |
| YJM789 | 9217 | ATTATTTAATTTTATAATACT----------------------------------TAATTATTAATATATTATTC--ATATATATATAAATTAAATTAAA | 9280 |
| YJM1273 | 9347 | ATTATTTAATTTTATAATACT----------------------------------TAATTATTAATATATTATTC--ATATATATATAAATTAAATTAAA | 9410 |
| NCYC3585 | 9378 | ATTATTTAATTTTATAATACT----------------------------------TAATTATTAATATATTATTC--ATATATATATAAATTAAATTAAA | 9441 |
| YJM1401 | 9823 | ATTATTTAATTTTATAATACT----------------------------------TAATTATTAATATATTATTC--ATATATATATAAATTAAATTAAA | 9886 |
| NCYC3594 | 9619 | ATTATTTAATTTTATAATACT----------------------------------TAATTATTAATATATTATTCATATATATATATAAATTAAATTAAA | 9684 |
| YJM1078 | 9493 | ATTATTTAATTTTATAATACT----------------------------------TAATTATTAATATATTATTCATATATATATATAAATTAAATTAAA | 9558 |
| YJM1439 | 10191 | ATTATTTAATTTTATAATACTCCTTCGGGGTCCGCCCCGCGGGGGCGGGCCGGACTATTTATTAATATATTATTC--ATATATATATAAATTAAATTAAA | 10288 |
| consensus | 11801 | \*\*\*\*\*\*\*\*\*\*\*\*\*\*\*\*\*\*\*\*\*----------------------------------\*\*.\*\*\*\*\*\*\*\*\*\*\*\*\*\*\*\*\*--\*\*\*\*\*\*\*\*\*\*\*\*\*\*\*\*\*\*\*\*\*\*\* | 11900 |
|
| S288C | 9794 | TTAATTATATTGAATATATAAATATATATATATATAAATATAT-AAAAAATTATATAAATTATTTTAAG------------------------------- | 9861 |
| YJM1388 | 9736 | TTAATTATATTGAATATATAAATATATATATATATAAATATATAAAAAAATTATATAAATTATTTTAAGAAAGGAGAAATATTATAATATATTTAATATT | 9835 |
| YJM789 | 9281 | TTAATTATATTGAATATATAAATATATATATATATAAATATAT-AAAAAATTATATAAATTATTTTAAG------------------------------- | 9348 |
| YJM1273 | 9411 | TTAATTATATTGAATATATAAATATATATATATATAAATATAT-AAAAAATTATATAAATTATTTTAAG------------------------------- | 9478 |
| NCYC3585 | 9442 | TTAATTATATTGAATATATAAATATATATATATATAAATATAT-AAAAAATTATATAAATTATTTTAAG------------------------------- | 9509 |
| YJM1401 | 9887 | TTAATTATATTGAATATATAAATATATATATATATAAATATAT-AAAAAATTATATAAATTATTTTAAG------------------------------- | 9954 |
| NCYC3594 | 9685 | TTAATTATATTGAATATATAAATATATATATATATAAATATAT-AAAAAATTATATAAATTATTTTAAG------------------------------- | 9752 |
| YJM1078 | 9559 | TTAATTATATTGAATATATAAATATATATATATATAAATATAT-AAAAAATTATATAAATTATTTTAAG------------------------------- | 9626 |
| YJM1439 | 10289 | TTAATTATATTGAATATATAAATATATATATATATAAATATAT-AAAAAATTATATAAATTATTTTAAG------------------------------- | 10356 |
| consensus | 11901 | \*\*\*\*\*\*\*\*\*\*\*\*\*\*\*\*\*\*\*\*\*\*\*\*\*\*\*\*\*\*\*\*\*\*\*\*\*\*\*\*\*\*\*-\*\*\*\*\*\*\*\*\*\*\*\*\*\*\*\*\*\*\*\*\*\*\*\*\*------------------------------- | 12000 |
|
| S288C | 9862 | ---------------------------------------------------------------------------------------------------- | 9861 |
| YJM1388 | 9836 | TTATTTTTTTTTTTTATTTTAATTTATTAAATAATTTAATAATTAAATAAATTAATGATTTGATTTTATTCTATTATTATTATATTTTATTATATAAAAA | 9935 |
| YJM789 | 9349 | ---------------------------------------------------------------------------------------------------- | 9348 |
| YJM1273 | 9479 | ---------------------------------------------------------------------------------------------------- | 9478 |
| NCYC3585 | 9510 | ---------------------------------------------------------------------------------------------------- | 9509 |
| YJM1401 | 9955 | ---------------------------------------------------------------------------------------------------- | 9954 |
| NCYC3594 | 9753 | ---------------------------------------------------------------------------------------------------- | 9752 |
| YJM1078 | 9627 | ---------------------------------------------------------------------------------------------------- | 9626 |
| YJM1439 | 10357 | ---------------------------------------------------------------------------------------------------- | 10356 |
| consensus | 12001 | ---------------------------------------------------------------------------------------------------- | 12100 |
|
| S288C | 9862 | ---------------------------------------------------------------------------------------------------- | 9861 |
| YJM1388 | 9936 | TAAATAATAAATAATAAATAGTATGATACAATTTATTAATAATATAATTATAAATTATAAATAATAATTTATTTTAATAATTCTCCTTTCGGGGTTCCGG | 10035 |
| YJM789 | 9349 | ---------------------------------------------------------------------------------------------------- | 9348 |
| YJM1273 | 9479 | ---------------------------------------------------------------------------------------------------- | 9478 |
| NCYC3585 | 9510 | ---------------------------------------------------------------------------------------------------- | 9509 |
| YJM1401 | 9955 | ---------------------------------------------------------------------------------------------------- | 9954 |
| NCYC3594 | 9753 | ---------------------------------------------------------------------------------------------------- | 9752 |
| YJM1078 | 9627 | ---------------------------------------------------------------------------------------------------- | 9626 |
| YJM1439 | 10357 | ---------------------------------------------------------------------------------------------------- | 10356 |
| consensus | 12101 | ---------------------------------------------------------------------------------------------------- | 12200 |
|
| S288C | 9862 | ---------------------------------------------------------------------------------------------------- | 9861 |
| YJM1388 | 10036 | CTCCCGTGGCCGGGCCCCGGAACTATTAATATAAATAATGATAATTATTTTATCATTATTAACTATTAATATATTAATCTATTAATATTCTTTGATATTT | 10135 |
| YJM789 | 9349 | ---------------------------------------------------------------------------------------------------- | 9348 |
| YJM1273 | 9479 | ---------------------------------------------------------------------------------------------------- | 9478 |
| NCYC3585 | 9510 | ---------------------------------------------------------------------------------------------------- | 9509 |
| YJM1401 | 9955 | ---------------------------------------------------------------------------------------------------- | 9954 |
| NCYC3594 | 9753 | ---------------------------------------------------------------------------------------------------- | 9752 |
| YJM1078 | 9627 | ---------------------------------------------------------------------------------------------------- | 9626 |
| YJM1439 | 10357 | ---------------------------------------------------------------------------------------------------- | 10356 |
| consensus | 12201 | ---------------------------------------------------------------------------------------------------- | 12300 |
|
| S288C | 9862 | ---------------------------------------------------------------------------------------------------- | 9861 |
| YJM1388 | 10136 | ATTTTTATTTAATGAACATCTAATTTAATTTAATTTAATAAAATAGTCCGGTCCGCCCCGCGGAGGGGGCGGACCTCGAAGGAGATATGAATCTTTAATA | 10235 |
| YJM789 | 9349 | ---------------------------------------------------------------------------------------------------- | 9348 |
| YJM1273 | 9479 | ---------------------------------------------------------------------------------------------------- | 9478 |
| NCYC3585 | 9510 | ---------------------------------------------------------------------------------------------------- | 9509 |
| YJM1401 | 9955 | ---------------------------------------------------------------------------------------------------- | 9954 |
| NCYC3594 | 9753 | ---------------------------------------------------------------------------------------------------- | 9752 |
| YJM1078 | 9627 | ---------------------------------------------------------------------------------------------------- | 9626 |
| YJM1439 | 10357 | ---------------------------------------------------------------------------------------------------- | 10356 |
| consensus | 12301 | ---------------------------------------------------------------------------------------------------- | 12400 |
|
| S288C | 9862 | ---------------------------------------------------------------------------------------------------- | 9861 |
| YJM1388 | 10236 | TAAATAATCATAAATTCTAAATATTAGAATTATTATTTTATCTTTTTATTTATTAATAATTAAATATAAATTATTATTAATAATAATTATAATATTCTCC | 10335 |
| YJM789 | 9349 | ---------------------------------------------------------------------------------------------------- | 9348 |
| YJM1273 | 9479 | ---------------------------------------------------------------------------------------------------- | 9478 |
| NCYC3585 | 9510 | ---------------------------------------------------------------------------------------------------- | 9509 |
| YJM1401 | 9955 | ---------------------------------------------------------------------------------------------------- | 9954 |
| NCYC3594 | 9753 | ---------------------------------------------------------------------------------------------------- | 9752 |
| YJM1078 | 9627 | ---------------------------------------------------------------------------------------------------- | 9626 |
| YJM1439 | 10357 | ---------------------------------------------------------------------------------------------------- | 10356 |
| consensus | 12401 | ---------------------------------------------------------------------------------------------------- | 12500 |
|
| S288C | 9862 | ----T----------------------------------------------AAAAATAATATTAAT-----------------AAAAAT----------- | 9883 |
| YJM1388 | 10336 | TTTCT----------------------------------------------TAAAATAATATTAATA----------------AAAAAT----------- | 10362 |
| YJM789 | 9349 | ----AAAGGAGTGAGGGACCCCCTCCCGTTAGGGAGGGGGACCGAACCCCGAAGGAGAAA---------------------------------------- | 9404 |
| YJM1273 | 9479 | ----A----------------------------------------------AAGGAGAAA---------------------------------------- | 9488 |
| NCYC3585 | 9510 | ----A----------------------------------------------AAGGAGAAA---------------------------------------- | 9519 |
| YJM1401 | 9955 | ----A----------------------------------------------AAGGAGAAA---------------------------------------- | 9964 |
| NCYC3594 | 9753 | ----TT---------------------------------------------AAAAATAATATTAAT-----------------AAA-------------- | 9772 |
| YJM1078 | 9627 | ----TT---------------------------------------------AAAAATAATATTAAT-----------------AAA-------------- | 9646 |
| YJM1439 | 10357 | ----T----------------------------------------------AAAAATAATATTAATAGTGAACACCTTTATTTAAAGGTGTGAACCAATC | 10406 |
| consensus | 12501 | ----..---------------------------------------------.\*..\*.\*\*.--------------------------..------------ | 12600 |
|
| S288C | 9884 | -------------------------------------------------TATACAATAATAATAATAAA--------------TATTCATTATTATTTAA | 9920 |
| YJM1388 | 10363 | -------------------------------------------------TATACAATAATAATAATAAA--------------TATTCATTATTATTTAA | 10399 |
| YJM789 | 9405 | ---------------------------------------------------------------------------------------TATTATAATATAT | 9417 |
| YJM1273 | 9489 | ---------------------------------------------------------------------------------------TATTATAATATAT | 9501 |
| NCYC3585 | 9520 | ---------------------------------------------------------------------------------------TATTATAATATAT | 9532 |
| YJM1401 | 9965 | ---------------------------------------------------------------------------------------TATTATAATATAT | 9977 |
| NCYC3594 | 9773 | ------------------------------------------------------------AATTATACAATAATAATAATTAATATTCATTATTATTTAA | 9812 |
| YJM1078 | 9647 | ------------------------------------------------------------AATTATACAATAATAATAATTAATATTCATTATTATTTAA | 9686 |
| YJM1439 | 10407 | CCGCAAGGCAAGGGAGGAGTTCCGGGGCCCGGCCACGGGAGCCGGAACCCCGAAAGGAGAAATTATACAATAATAATAATAAATATTCATTATTATTTAA | 10506 |
| consensus | 12601 | -------------------------------------------------...-.-..-..---.---.------------.------.\*\*\*\*\*.\*\*.\*\*. | 12700 |
|
| S288C | 9921 | TTAATATCTCCTTTA-CTTCTTTTTCCTCCGTTGAGGACTTATTATTAAGTATATTATTATATACTACTTAAGATTATATATATAATATATATATATATA | 10019 |
| YJM1388 | 10400 | TTAATATCTCCTTTA-CTTATTTTTCCTCCGTTAGGGACTTATTATTAAGTATATTATTATATACTACTTAAGATTATATATATA--ATATATATATATA | 10496 |
| YJM789 | 9418 | TTAATAT----TTTA-TTTTTTTT--------------------------TATTTTAATTTAT------------------------------------- | 9449 |
| YJM1273 | 9502 | TTAATAT----TTTA-TTTTTTTT--------------------------TATTTTAATTTAT------------------------------------- | 9533 |
| NCYC3585 | 9533 | TTAATAT----TTTA--TTTTTTT--------------------------TATTTTAATTTAT------------------------------------- | 9563 |
| YJM1401 | 9978 | TTAATAT----TTTATTTTTTTTT--------------------------TATTTTAATTTAT------------------------------------- | 10010 |
| NCYC3594 | 9813 | TTAATATCTCCTTTA-CTTATTTTTCCTCCGTTAGGGACTTATTATTAAGTATATTATTATATACTACTTAAGATTATATATATAATATATATATATATT | 9911 |
| YJM1078 | 9687 | TTAATATCTCCTTTA-CTTATTTTTCCTCCGTTAGGGACTTATTATTAAGTATATTATTATATACTACTTAAGATTATATATATAATATATATATATATT | 9785 |
| YJM1439 | 10507 | TTAATATCTCCTTTA-CTTATTTTTCCTCCGTTAAGGACTTATTATTAAGTATATTATTATATACTACTTAAGATTATATATATAATATATATATATATT | 10605 |
| consensus | 12701 | \*\*\*\*\*\*\*----\*\*\*\*-.\*\*.\*\*\*\*---------..---------------\*\*\*.\*\*\*.\*.\*\*\*------------------------------------. | 12800 |
|
| S288C | 10020 | TTATATATAAAATATAAATATATAAATAATATAAAAATTAATAAAATAAATAAAATAAATTAGTCCGATCGAA--TCCCCT------------------- | 10098 |
| YJM1388 | 10497 | TTATATATAAAATATAAATATATAAATAATATAAAAATTAATAAAATAAATAAAATAAATTAGTCCGATCGAA--TCCCCT------------------- | 10575 |
| YJM789 | 9450 | ----------------------TAAATAATTTAATAATTAAATAAATTAATGATTTGATTTTATTCTATT-AT--TATTAT------------------- | 9505 |
| YJM1273 | 9534 | ----------------------TAAATAATTTAATAATTAAATAAATTAATGATTTGATTTTATTCTATT-AT--TATTAT------------------- | 9589 |
| NCYC3585 | 9564 | ----------------------TAAATAATTTAATAATTAAATAAATTAATGATTTAATTTTATTCTATT-AT--TATTAT------------------- | 9619 |
| YJM1401 | 10011 | ----------------------TAAATAATTTAATAATTAAATAAATTAATGATTTGATTTTATTCTATT-AT--TATTAT------------------- | 10066 |
| NCYC3594 | 9912 | ATATATA-AAAATATAAATATATAAATAATATAAAAATTAATAAAATAAATAAAATAAATTAGTCCGATCGAA--TCCCCTATTAAATTAAATTAAATTA | 10008 |
| YJM1078 | 9786 | ATATATA-AAAATATAAATATATAAATAATATAAAAATTAATAAAATAAATAAAATAAATTAGTCCGATCGAACCCCCCCTATTAAATTTAATTTAATTT | 9884 |
| YJM1439 | 10606 | ATATAT--AAAATATAAATATATAAATAATATAAAAATTAATAAAATAAATAAAATAAATTAGTCCGATCGAA--TCCCCT------------------- | 10682 |
| consensus | 12801 | .---------------------\*\*\*\*\*\*\*\*.\*\*\*.\*\*\*\*\*\*..\*\*\*\*.\*\*\*.\*..\*.\*.\*\*..\*.\*.\*\*.-\*.--.....\*--------.----.----. | 12900 |
|
| S288C | 10099 | ----------------ATTT---------------AATTAAATTAAA----------------------------------------------------- | 10114 |
| YJM1388 | 10576 | ----------------ATTTAATTAAATTAAATTAAATTAAATTAAA----------------------------------------------------- | 10606 |
| YJM789 | 9506 | ----------------ATTT---------------TATTATATAAAA----------------------------------------------------- | 9521 |
| YJM1273 | 9590 | ----------------ATTT---------------TATTATATAAAA----------------------------------------------------- | 9605 |
| NCYC3585 | 9620 | ----------------ATTT---------------TATTATATAAAA----------------------------------------------------- | 9635 |
| YJM1401 | 10067 | ----------------ATTT---------------TATTATATAAAA----------------------------------------------------- | 10082 |
| NCYC3594 | 10009 | AATTAAATTAAATTAAATTA---------------AATTAAATTAAA----------------------------------------------------- | 10040 |
| YJM1078 | 9885 | AATTAAATTTAATTTAATTA---------------AGAAGGATTGAGGGACCCCCTCCCTATACTTACGGGAGGGGGGACCGAACCCCAAAGGAGTGCGG | 9969 |
| YJM1439 | 10683 | -ATTAAATTAAATTAAATTA---------------AATTAAATTAAA----------------------------------------------------- | 10713 |
| consensus | 12901 | ---------.----.-\*\*\*.---------------......\*\*..\*.----------------------------------------------------- | 13000 |
|
| S288C | 10115 | ----------------------TTAAATTAAGAA-AGAGATAAATTTATATAAAATATTATTTATAATTAATTATAATTAAATTATAATATAATATAATA | 10191 |
| YJM1388 | 10607 | ----------------------TTAAATTAAGAA-AGAGATAAATTTATATAAAATATTATTTATAATTAATTATAATTAAATTATAATATAATATAATA | 10683 |
| YJM789 | 9522 | ----------------------ATAA-------------------------------------ATAATAAATAATAA-------ATAGTATGATACAAT- | 9554 |
| YJM1273 | 9606 | ----------------------ATAA-------------------------------------ATAATAAATAATAA-------ATAGTATGATACAAT- | 9638 |
| NCYC3585 | 9636 | ----------------------ATAA-------------------------------------ATAATAAATAATAA-------ATAGTATGATACAAT- | 9668 |
| YJM1401 | 10083 | ----------------------ATAA-------------------------------------ATAATAAATAATAA-------ATAGTATGATACAAT- | 10115 |
| NCYC3594 | 10041 | ----------------------TTAAATTAAGAA-AGAGATAAATTTATATAAAATATTATTTATAATTAATTATAATTAAATTATAATATAATATA--- | 10114 |
| YJM1078 | 9970 | GACCCCATGGGAACCCCTTTTTTATTCTTAAGAAGGGGGATAAATTTATATAAAATATTATTTATAATTAATTATAATTAAATTATAATATAATATA--- | 10066 |
| YJM1439 | 10714 | ----------------------TTAAATTAAGAA-AGAGATAAATTTATATAAAATATTATTTATAATTAATTATAATTAAATTATAATATAATATAATA | 10790 |
| consensus | 13001 | ----------------------.....--------.-.-------------------------\*\*\*\*\*.\*\*\*.\*\*\*\*-------\*\*\*.\*\*\*.\*\*\*.\*--- | 13100 |
|
| S288C | 10192 | TAAATAATAATATAATAAAAATAAAAATAAAATAATATTAGATTATATTATATAATTTATATAATTTTTTAATAATAA---------------------- | 10269 |
| YJM1388 | 10684 | TAAATAATAATAT------AATAAAAATAAAATAATATTAGATTATATTATATAATTTATATAATTTTTTAATAATAA---------------------- | 10755 |
| YJM789 | 9555 | TTATTAATAATATAATTA--------------------TAAATTATAAATAATAATTTA-------TTTTAATAATTC---------------------- | 9605 |
| YJM1273 | 9639 | TTATTAATAATATAATTA--------------------TAAATTATAAATAATAATTTA-------TTTTAATAATTC---------------------- | 9689 |
| NCYC3585 | 9669 | TTATTAATAATATAATTA--------------------TAAATTATAAATAATAATTTA-------TTTTAATAATTC---------------------- | 9719 |
| YJM1401 | 10116 | TTATTAATAATATAATTA--------------------TAAATTATAAATAATAATTTA-------TTTTAATAATTCTCCTTTCGGGGTTCCGGCTCCC | 10188 |
| NCYC3594 | 10115 | --AATAATAATATAATAAAAATAAAATAATAT------TAGATTATATTATATAATTTATATAAT-TTTTAATAATAA---------------------- | 10183 |
| YJM1078 | 10067 | --AATAATAATATAATAAAAATAAAATAATAT------TAGATTATATTATATAATTTATATAAT-TTTTAATAATAA---------------------- | 10135 |
| YJM1439 | 10791 | TAAATAATAATATAATAAAAATAAAATAATAT------TAGATTATATTATATAATTTATATAAT-TTTTAATAATAA---------------------- | 10861 |
| consensus | 13101 | -.\*.\*\*\*\*\*\*\*\*\*---.---------..-.-.------\*\*.\*\*\*\*\*\*....\*\*\*\*\*\*\*\*-------\*\*\*\*\*\*\*\*\*\*..---------------------- | 13200 |
|
| S288C | 10270 | ---------------------TAATAAATAAGTTTATTTATAATTATAAATATAAATATAAATATAAATAAAGA-AGGTATTATATT-TTATAAAATATA | 10346 |
| YJM1388 | 10756 | ---------------------TAATAAATAAGTTTATTTATAAT------TATAAATATAAATATAAATAAAGA-AGGTATTATATT-TTATAAAATATA | 10826 |
| YJM789 | 9606 | ---------------------T--------------------------------------AATATAAATAATGATAATAATTTTATCATTATGAACTATT | 9646 |
| YJM1273 | 9690 | ---------------------T--------------------------------------AATATAAATAATGATAATTATTTTATCATTATGAACTATT | 9730 |
| NCYC3585 | 9720 | ---------------------T--------------------------------------AATATAAATAATGATAATAATTTTATCATTATGAACTATT | 9760 |
| YJM1401 | 10189 | GTGGCCGGGCCCCGGAACTATT--------------------------------------AATATAAATAATGATAATTATTTTATCATTATGAACTATT | 10250 |
| NCYC3594 | 10184 | ---------------------TA----------------------------------------ATAAATAAGTTTA-----TTTATAATTA-TAAATATA | 10216 |
| YJM1078 | 10136 | ---------------------TA----------------------------------------ATAAATAAGTTTA-----TTTATAATTA-TAAATATA | 10168 |
| YJM1439 | 10862 | ---------------------TA----------------------------------------ATAAATA------------------------------ | 10870 |
| consensus | 13201 | ---------------------\*-----------------------------------------\*\*\*\*\*\*\*-...--...---.---.-----.--.---. | 13300 |
|
| S288C | 10347 | ------ATAATAATACAAAATTTATAT-------------------TTTAATAAAT-------------------------------------------- | 10377 |
| YJM1388 | 10827 | ------ATAATAATACAAAATTTATAT-------------------TTTAATAAAT-------------------------------------------- | 10857 |
| YJM789 | 9647 | ------AATATATTAATCTATTAATATTCTTTGATATTTATTTTTATTTAATGAACATC-----TAATTTAATTTAA----------TAAAATAGTCCGG | 9725 |
| YJM1273 | 9731 | ------AATATATTAATATATTAATATTCTTTGATATTTATTTTTATTTAATGAACATC-----TAATTTAATTTAATTTAATTTAATAAAATAGTCCGG | 9819 |
| NCYC3585 | 9761 | ------AATATATTAATCTATTAATATTCTTTGATATTTATTTTTATTTAATGAACATCTAATTTAATTTAATTTAA----------TAAAATAGTCCGG | 9844 |
| YJM1401 | 10251 | ------AATATATTAATCTATTAATATTCTTTGATATTTATTTTTATTTAATGAACATCTAATTTAATTTAATTTAA----------TAAAATAGTCCGG | 10334 |
| NCYC3594 | 10217 | ------AATATAAATAAAGA-------------------------------------------------------------------------------- | 10230 |
| YJM1078 | 10169 | AATATAAATATAAATAAAGA-------------------------------------------------------------------------------- | 10188 |
| YJM1439 | 10871 | ----------------------------------------------------------------------------------------------GTCCGG | 10876 |
| consensus | 13301 | -------..---.......---.-----------------------------.--.-------------------------------------------- | 13400 |
|
| S288C | 10378 | ---------------------------------------------------------------------------------------------------- | 10377 |
| YJM1388 | 10858 | ---------------------------------------------------------------------------------------------------- | 10857 |
| YJM789 | 9726 | TCCGCCCC-GTGGAGGGGGC-GACCTCGAAGGAGATATGAATCTTTAATATAAATAATCATAAATTCTAAATATTAGAATTATTATTTTATCTTTTTATT | 9823 |
| YJM1273 | 9820 | TCCGCCCC-GTGGAGGGGGCGGACCTCGAAGGAGATATGAATCTTTAATATAAATAATCATAAATTCTAAATATTAGAATTATTATTTTATCTTTTTATT | 9918 |
| NCYC3585 | 9845 | TCCGCCCC-GCGGAGGGGGCGGACCTCGAAGGAGATATGAATCTTTAATATAAATAATCATAAATTCTAAATATTAGAATTATTATTTTATCTTTTTATT | 9943 |
| YJM1401 | 10335 | TCCGCCCC-GCGGAGGGGGCGGACCTCGAAGGAGATATGAATCTTTAATATAAATAATCATAAATTCTAAATATTAGAATTATTATTTTATCTTTTTATT | 10433 |
| NCYC3594 | 10231 | ---------------------------------------------------------------------------------------------------- | 10230 |
| YJM1078 | 10189 | ---------------------------------------------------------------------------------------------------- | 10188 |
| YJM1439 | 10877 | CCCGCCCCCGCGGGGGGGGCGGACCCCGAAGGAGTGAGGGACC--------------------------------------------------------- | 10919 |
| consensus | 13401 | .---------.--.-----------.--------..-.-.-.---------------------------------------------------------- | 13500 |
|
| S288C | 10378 | ---------------------------------------------------------------------------------------------------- | 10377 |
| YJM1388 | 10858 | ---------------------------------------------------------------------------------------------------- | 10857 |
| YJM789 | 9824 | TATTAATAATTAAATATAAATTATTATTAATAATAATTATAATATTCTCCTTTCGGGGTTCCGGCTCCCGTGGCCGGGCCCCGGAACTTAAAAATAATAT | 9923 |
| YJM1273 | 9919 | TATTAATAATTAAATATAAATTATTATTAATAATAATTATAATATTCTCCTTTCGGGGTTCCGGCTCCCGTGGCCGGGCCCCGGAACTTTAAAATAATAT | 10018 |
| NCYC3585 | 9944 | TATTAATAATTAAATATAAATTATTATTAATAATAATTATAATATT----------------------------------------CTTTAAAATAATAT | 10003 |
| YJM1401 | 10434 | TATTAATAATTAAATATAAATTATTATTAATAATAATTATAATATTCTCCTTTC----------------------------------TTAAAATAATAT | 10499 |
| NCYC3594 | 10231 | ---------------------------------------------------------------------------------------------------- | 10230 |
| YJM1078 | 10189 | ---------------------------------------------------------------------------------------------------- | 10188 |
| YJM1439 | 10920 | ---------------------------------------------------------------------------------------------------- | 10919 |
| consensus | 13501 | -----------------------------------------------------------------------------------------.---------- | 13600 |
|
| S288C | 10378 | ---------------------------------------------------------------------------------------------------- | 10377 |
| YJM1388 | 10858 | ---------------------------------------------------------------------------------------------------- | 10857 |
| YJM789 | 9924 | TAATAAAAAATTATACAATAATAATAATAAATATTCATTATTATTTAATTAATATCTCCTTTACTTATTTTTCCTCCGTTAGGGACTTATTATTAAGTAT | 10023 |
| YJM1273 | 10019 | TAAT-AAAAATTATACAATAATAATAATAAATATTCATTATTATTTAATTAATATCTCCTTTACTTATTTTTCCTCCGTTAGGGACTTATTATTAAGTAT | 10117 |
| NCYC3585 | 10004 | TAAT-AAAAATTATACAATAATAATAATTAATATTCATTATTATTTAATTAATATCTCCTTTACTTATTTTTCCTCCGTTGAGGACTTATTATTAAGTAT | 10102 |
| YJM1401 | 10500 | TAATAAAAAATTATACAATAATAATAATAAATATTCATTATTATTTAATTAATATCTCCTTTACTTATTTTTCCTCCGTTAGGGACTTATTATTAAGTAT | 10599 |
| NCYC3594 | 10231 | ---------------------------------------------------------------------------------------------------- | 10230 |
| YJM1078 | 10189 | ---------------------------------------------------------------------------------------------------- | 10188 |
| YJM1439 | 10920 | ------------------------------------------------------------------------CCTCCCTAACGGG--------------- | 10932 |
| consensus | 13601 | ----------------------------.------------------------------------------------.-...--.--------------- | 13700 |
|
| S288C | 10378 | ---------------------------------------------------------------------------------------------------- | 10377 |
| YJM1388 | 10858 | ---------------------------------------------------------------------------------------------------- | 10857 |
| YJM789 | 10024 | ATTATTATATACTACTTAAGATTATATATATA--ATATATATATATATTATATATAAAATATAAATATATAAATAATATAAAAATTAATAAAATAAATAA | 10121 |
| YJM1273 | 10118 | ATTATTATATACTACTTAAGATTATATATATA--ATATATATATATATTATATATAAAATATAAATATATAAATAATATAAAAATTAATAAAATAAATAA | 10215 |
| NCYC3585 | 10103 | ATTATTATATACTACTTAAGATTATATATATAATATATATATATATATTATATATAAAATATAAATATATAAATAATATAAAAATTAATAAAATAAATAA | 10202 |
| YJM1401 | 10600 | ATTATTATATACTACTTAAGATTATATATATA--ATATATATATATATTATATATAAAATATAAATATATAAATAATATAAAAATTAATAAAATAAATAA | 10697 |
| NCYC3594 | 10231 | ---------------------------------------------------------------------------------------------------- | 10230 |
| YJM1078 | 10189 | ---------------------------------------------------------------------------------------------------- | 10188 |
| YJM1439 | 10933 | ---------------------------------------------------------------------------------------------------- | 10932 |
| consensus | 13701 | ---------------------------------------------------------------------------------------------------- | 13800 |
|
| S288C | 10378 | ---------------------------------------------------------------------------------------------------- | 10377 |
| YJM1388 | 10858 | ---------------------------------------------------------------------------------------------------- | 10857 |
| YJM789 | 10122 | AATAAATTAGTCCGATCGAATCCCCTATTTAATTAAATTAAATTAAATTAAATTAAATTAAATTAAATTAAATTAAATTAAATTAAATTAAGAAAGAGAT | 10221 |
| YJM1273 | 10216 | AATAAATTAGTCCGATCGAATCCCCT-----------------------------------ATTAAATTAAATTAAATTAAATTAAATTAAGAAAGAGAT | 10280 |
| NCYC3585 | 10203 | AATAAATTAGTCCGATCGAATCCCCT--------------------ATTAAATTAAATTAAATTAAATTAAATTAAATTAAATTAAATTAAGAAAGAGAT | 10282 |
| YJM1401 | 10698 | AATAAATTAGTCCGATCGAATCCCCT------------------------------ATTTAATTAAATTAAATTAAATTAAATTAAATTAAGAAAGAGAT | 10767 |
| NCYC3594 | 10231 | ---------------------------------------------------------------------------------------------------- | 10230 |
| YJM1078 | 10189 | ---------------------------------------------------------------------------------------------------- | 10188 |
| YJM1439 | 10933 | --------AGGGGGACCGAACCCCTTT--------------------------------------------------TTATCTTTAATTAAGAAGGA--- | 10971 |
| consensus | 13801 | ----------...--.----.---.-.--------------------------------.--------------------..--.---------.----- | 13900 |
|
| S288C | 10378 | ---------------------------------------------------------------------------------------------------- | 10377 |
| YJM1388 | 10858 | ---------------------------------------------------------------------------------------------------- | 10857 |
| YJM789 | 10222 | AAATTTATATAAAATATTATTTATAATTAATTATAATTAAATTATAATATAATATAATATAAATAATAATATAATAAAAATAAAATAATATTAGATTATA | 10321 |
| YJM1273 | 10281 | AAATTTATATAAAATATTATTTATAATTAATTATAATTAAAT-----TATAATATAATATAAATAATAATATAATAAAAATAAAATAATATTAGATTATA | 10375 |
| NCYC3585 | 10283 | AAATTTATATAAAATATTATTTATAATTAATTATAATTAAATTATAATATAATATAATATAAATAATAATATAATAAAAATAAAATAATATTAGATTATA | 10382 |
| YJM1401 | 10768 | AAATTTATATAAAATATTATTTATAATTAATTATAATTAAATTATAATATAATATAATATAAATAATAATATAATAAAAATAAAATAATATTAGATTATA | 10867 |
| NCYC3594 | 10231 | ---------------------------------------------------------------------------------------------------- | 10230 |
| YJM1078 | 10189 | ---------------------------------------------------------------------------------------------------- | 10188 |
| YJM1439 | 10972 | ---------------------------------------------------------------------------------------------------- | 10971 |
| consensus | 13901 | ---------------------------------------------------------------------------------------------------- | 14000 |
|
| S288C | 10378 | ---------------------------------------------------------------------------------------------------- | 10377 |
| YJM1388 | 10858 | ---------------------------------------------------------------------------------------------------- | 10857 |
| YJM789 | 10322 | TTATATAATTTATATAA-TTTTTAATAATAATAATAAATAAGTTTATTTATAAT------------TATAAATATAAATAAAGAAGGTATTATATTTTAT | 10408 |
| YJM1273 | 10376 | TTATATAATTTATATAATTTTTTAATAATAATAATAAATAAGTTTATTTATAATT------ATAAATATAAATATAAATAAAGAAGGTATTATATTTTAT | 10469 |
| NCYC3585 | 10383 | TTATATAATTTATATAA-TTTTTAATAATAATAATAAATAAGTTTATTTATAATT------ATAAATATAAATATAAATAAAGAAGGTATTATATTTTAT | 10475 |
| YJM1401 | 10868 | TTATATAATTTATATAATTTTTTAATAATAATAATAAATAAGTTTATTTATAATTATAAATATAAATATAAATATAAATAAAGAAGGTATTATATTTTAT | 10967 |
| NCYC3594 | 10231 | ------------------------------------------------------------------------------------AGGTATTATATTTTAT | 10246 |
| YJM1078 | 10189 | ------------------------------------------------------------------------------------AGGTATTATATTTTAT | 10204 |
| YJM1439 | 10972 | -----------------------------------------GTTTATTTATAATT------ATAAATATAAATATAAATAAAGAAGGTATTATATTTTAT | 11024 |
| consensus | 14001 | ---------------------------------------------------------------------------------------------------- | 14100 |
|
| S288C | 10378 | ---------------------------------------ATTAATATAAGTTTAAAGTTCC--GGGGCCCGG-CACGGGAGCCGGAACCCCGAAAGGAGA | 10435 |
| YJM1388 | 10858 | ---------------------------------------ATTAATATAAGTTTAAAGTTCC--GGGGCCCGGCCACGGGAGCCGGAACCCCGAAAGGAGA | 10916 |
| YJM789 | 10409 | AAAATATAATAATAATACAAAATTTATATTTTAATAAATATTAATATAAGTTTAAAGTTCC--GGGGCCCGGCCACGGGAGCCGGAACCCCGAAAGGAGA | 10506 |
| YJM1273 | 10470 | AAAATATAATAATAATACAAAATTTATATTTTAATAAATATTAATATAAGTTTAAAGTTCC--GGGGGCCGGCCACGGGAGCCGGAACCCCGAAAGGAGA | 10567 |
| NCYC3585 | 10476 | AAAATATAATAATAATACAAAATTTATATTTTAATAAATATTAATATAAGTTTAAAGTTCC--GGGGGCCGGCCACGGGAGCCGGAACCCCGAAAGGAGA | 10573 |
| YJM1401 | 10968 | AAAATATAATAATAATACAAAATTTATATTTTAATAAATATTAATATAAGTTTAAAGTTCC--GGGGGCCGGCCACGGGAGCCGGAACCCCGAAAGGAGA | 11065 |
| NCYC3594 | 10247 | AAAATATAATAATAATACAAAATTTATATTTTAATAAATATTAATATAAGTTTAAAGTTCCGGGGGGCCCGGCCACGGGAGCCGGAACCCCGAAAGGAGA | 10346 |
| YJM1078 | 10205 | AAAATATAATAATAATACAAAATTTATATTTTAATAAATATTAATATAAGTTTAAAGTTCCGGGGGGCCCGGCCACGGGAGCCGGAACCCCGAAAGGAGA | 10304 |
| YJM1439 | 11025 | AAAATATAATAATAATACAAAATTTATATTTTAATAAATATTAATATAAGTTTAAAGTTCC--GGGGGCCGGCCACGGGAGCCGGAACCCCGAAAGGAGA | 11122 |
| consensus | 14101 | ---------------------------------------\*\*\*\*\*\*\*\*\*\*\*\*\*\*\*\*\*\*\*\*\*\*--\*\*\*\*.\*\*\*\*-\*\*\*\*\*\*\*\*\*\*\*\*\*\*\*\*\*\*\*\*\*\*\*\*\*\*\* | 14200 |
|
| S288C | 10436 | AATAAATAATATATTTATAAAAAATTAAATAAATAAATATTATCTATTTAAAAATAAATATAATATAATATAATATAATAATTCTAAATATAAATAATAT | 10535 |
| YJM1388 | 10917 | AATAAATAATATATTTATAAAAAATTAAATAAATAAATATTATCTATTTAAAAATA-----AATATAATATAATATAATAATTCTAAATATAAATAATAT | 11011 |
| YJM789 | 10507 | AATAAATAATATATTTATAAAAAATTAAATAAATAAATATTATCTATTTAAAAATAAATATAATATAATATAATATAATAATTCTAAATATAAATAATAT | 10606 |
| YJM1273 | 10568 | AATAAATAATATATTTATAAAAAATTAAATAAATAAATATTATCTATTTAAAAATAAATATAATATAATATAATATAATAATTCTAAATATAAATAATAT | 10667 |
| NCYC3585 | 10574 | AATAAATAATATATTTATAAAAAATTAAATAAATAAATATTATCTATTTAAAAATAAATATAATATAATATAATATAATAATTCTAAATATAAATAATAT | 10673 |
| YJM1401 | 11066 | AATAAATAATATATTTATAAAAAATTAAATAAATAAATATTATCTATTTAAAAATA-----AATATAATATAATATAATAATTCTAAATATAAATAATAT | 11160 |
| NCYC3594 | 10347 | AATAAATAATATATTTATAAAAAATTAAATAAATAAATATTATCTATTT-CAAATAAATATAATATAATATAATATAATAATTCTAAATATAAATAATAT | 10445 |
| YJM1078 | 10305 | AATAAATAATATATTTATAAAAAATTAAATAAATAAATATTATCTATTT-CAAATAAATATAATATAATATAATATAATAATTCTAAATATAAATAATAT | 10403 |
| YJM1439 | 11123 | AATAAATAATATATTTATAAAAAATTAAATAAATAAATATTATCTATTTAAAAATAAATATAATATAATATAATATAATAATTCTAAATATAAATAATAT | 11222 |
| consensus | 14201 | \*\*\*\*\*\*\*\*\*\*\*\*\*\*\*\*\*\*\*\*\*\*\*\*\*\*\*\*\*\*\*\*\*\*\*\*\*\*\*\*\*\*\*\*\*\*\*\*\*-.\*\*\*\*\*-----\*\*\*\*\*\*\*\*\*\*\*\*\*\*\*\*\*\*\*\*\*\*\*\*\*\*\*\*\*\*\*\*\*\*\*\*\*\*\* | 14300 |
|
| S288C | 10536 | TTATTATAATTATTATAATAATTGTATTATTTATTAATAATATATATAATTATATTAAAACTAATATTACATTATTTTGTATATTTAAACAATTAAATTG | 10635 |
| YJM1388 | 11012 | TTATTATAATTATTATAATAATTGTATTATTTATTAATAATATATATAATTATATTAAAACTAATATTACATTATTTTGTATATTTAAACAATTAAATTG | 11111 |
| YJM789 | 10607 | TTATTATAATTATTATAATAATTGTATTATTTATTAATAATATATATAATTATATTAAAACTAATATTACATTATTTTGTATATTTAAACAATTAAATTG | 10706 |
| YJM1273 | 10668 | TTATTATAATTATTATAATAATTGTATTATTTATTAATAATATATATAATTATATTAAAACTAATATTACATTATTTTGTATATTTAAACAATTAAATTG | 10767 |
| NCYC3585 | 10674 | TTATTATAATTATTATAATAATTGTATTATTTATTAATAATATATATAATTATATTAAAACTAATATTACATTATTTTGTATATTTAAACAATTAAATTG | 10773 |
| YJM1401 | 11161 | TTATTATAATTATTATAATAATTGTATTATTTATTAATAATATATATAATTATATTAAAACTAATATTACATTATTTTGTATATTTAAACAATTAAATTG | 11260 |
| NCYC3594 | 10446 | TTATTATAATTATTATAATAATTGTATTATTTATTAATAATATATATAATTATATTAAAACTAATATTACATTATTTTGTATATTTAAACAATTAAATTG | 10545 |
| YJM1078 | 10404 | TTATTATAATTATTATAATAATTGTATTATTTATTAATAATATATATAATTATATTAAAACTAATATTACATTATTTTGTATATTTAAACAATTAAATTG | 10503 |
| YJM1439 | 11223 | TTATTATAATTATTATAATAATTGTATTATTTATTAATAATATATATAATTATATTAAAACTAATATTACATTATTTTGTATATTTAAACAATTAAATTG | 11322 |
| consensus | 14301 | \*\*\*\*\*\*\*\*\*\*\*\*\*\*\*\*\*\*\*\*\*\*\*\*\*\*\*\*\*\*\*\*\*\*\*\*\*\*\*\*\*\*\*\*\*\*\*\*\*\*\*\*\*\*\*\*\*\*\*\*\*\*\*\*\*\*\*\*\*\*\*\*\*\*\*\*\*\*\*\*\*\*\*\*\*\*\*\*\*\*\*\*\*\*\*\*\*\*\*\* | 14400 |
|
| S288C | 10636 | ATTATTCTTATTTGTAATCTTTATTTATTTTATTATATCTTATTAATGAT-AAATTATAATTATTATTAAAATAATAATTTACTTCTTTTGATATAAAAA | 10734 |
| YJM1388 | 11112 | ATTATTCTTATTTGTAATCTTTATTTATTTTATTATATCTTATTAATGAT-AAATTATAATTATTATTAAAATAATAATTTACTTCTTTTGATATAAAAA | 11210 |
| YJM789 | 10707 | ATTATTCTTATTTGTAATCTTTATTTATTTTATTATATCTTATTAATGAT-AAATTATAATTATTATTAAAATAATAATTTACTTCTTTTGATATAAAAA | 10805 |
| YJM1273 | 10768 | ATTATTCTTATTTGTAATCTTTATTTATTTTATTATATCTTATTAATGAT-AAATTATAATTATTATTAAAATAATAATTTACTTCTTTTGATATAAAAA | 10866 |
| NCYC3585 | 10774 | ATTATTCTTATTTGTAATCTTTATTTATTTTATTATATCTTATTAATGAT-AAATTATAATTATTATTAAAATAATAATTTACTTCTTTTGATATAAAAA | 10872 |
| YJM1401 | 11261 | ATTATTCTTATTTGTAATCTTTATTTATTTTATTATATCTTATTAATGAT-AAATTATAATTATTATTAAAATAATAATTTACTTCTTTTGATATAAAAA | 11359 |
| NCYC3594 | 10546 | ATTATTCTTATTTGTAATCTTTATTTATTTTATTATATCTTATTAATGATAAAATTATAATTATTATTAAAATAATAATTTACTTCTTTTGATATAAAAA | 10645 |
| YJM1078 | 10504 | ATTATTCTTATTTGTAATCTTTATTTATTTTATTATATCTTATTAATGATAAAATTATAATTATTATTAAAATAATAATTTACTTCTTTTGATATAAAAA | 10603 |
| YJM1439 | 11323 | ATTATTCTTATTTGTAATCTTTATTTATTTTATTATATCTTATTAATGAT-AAATTATAATTATTATTAAAATAATAATTTACTTCTTTTGATATAAAAA | 11421 |
| consensus | 14401 | \*\*\*\*\*\*\*\*\*\*\*\*\*\*\*\*\*\*\*\*\*\*\*\*\*\*\*\*\*\*\*\*\*\*\*\*\*\*\*\*\*\*\*\*\*\*\*\*\*\*-\*\*\*\*\*\*\*\*\*\*\*\*\*\*\*\*\*\*\*\*\*\*\*\*\*\*\*\*\*\*\*\*\*\*\*\*\*\*\*\*\*\*\*\*\*\*\*\*\* | 14500 |
|
| S288C | 10735 | TAAAATAATATAGTTCCGGGGCCCGGCCACGGGAGCCGGAACCCCGGAAGGAGATAAATATATTATATTTTTATTCCTACCTATTAAAGGTAAAGACTCG | 10834 |
| YJM1388 | 11211 | TAAAATAATATAGTTCCGGGGCCCGGCCACGGGAGCCGGAACCCCGGAAGGAGATAAATATATTATATTTTTATTCCTACCTATTAAAGGTAAAGACTCG | 11310 |
| YJM789 | 10806 | TAAAATAATATAGTTCCGGGGCCCGGCCACGGGAGCCGGAACCCCGGAAGGAGATAAATATATTATATTTTTATTCCTTCCTATTAAAGGTAAAGACTCG | 10905 |
| YJM1273 | 10867 | TAAAATAATAT----------------------------------------TAGAAAATATATTATATTTTTATTCCTTCCTATTAAAGGTAAAGACTCG | 10926 |
| NCYC3585 | 10873 | TAAAATAATAT---------------------------------------TAGA-AAATATATTATATTTTTACTCCTTCCTATTAAAGGTAAAGACTCG | 10932 |
| YJM1401 | 11360 | TAAAATAATATAGTTCCGGGGCCCGGCCACGGGAGCCGGAACCCCGGAAGGAGATAAATATATTATATTTTTATTCCTTCCTATTAAAGGTAAAGACTCG | 11459 |
| NCYC3594 | 10646 | TAAAATAATATTA----------------------------------------GAAAATATATTATATTTTTACTCCTTCCTATTGAAAGTGAAGACTCA | 10705 |
| YJM1078 | 10604 | TAAAATAATATTA----------------------------------------GAAAATATATTATATTTTTACTCCTTCCTATTGAAAGTGAAGACTCA | 10663 |
| YJM1439 | 11422 | TAAAATAATATAGTTCCGGGGCCCGGCCACGGGAGCCGGAACCCCGGAAGGAGATAAATATATTATATTTTTATTCCTTCCTATTAAAGGTAAAGACTCG | 11521 |
| consensus | 14501 | \*\*\*\*\*\*\*\*\*\*\*..-------------------------------------.....\*\*\*\*\*\*\*\*\*\*\*\*\*\*\*\*\*\*.\*\*\*\*.\*\*\*\*\*\*.\*\*.\*\*.\*\*\*\*\*\*\*. | 14600 |
|
| S288C | 10835 | ATTCTCATAATTAAATTTATATCCTTCGGCCGGATTAATTTATTT------------TATTTATATTTATATT--------------------------- | 10895 |
| YJM1388 | 11311 | ATTCTCATAATTAAATTTATATCCTTCGGCCGGATTAATTTATTT------------TATTTATATTTATATT--------------------------- | 11371 |
| YJM789 | 10906 | ATTCTCATAATTAAATTTATATCCTTCGGCCGGATTAATTTATTT------------TATTTATATTTATATT--------------------------- | 10966 |
| YJM1273 | 10927 | ATTCTCATAATTAAATTTATATCCTTCGGCCGGATTAATTTATTT------------TATTTATATTTATATT--------------------------- | 10987 |
| NCYC3585 | 10933 | ACTCTCATAATTAAATTTATATCCTTCGGCCGGATTAATTTATTTTATTTATATTTATATTTATATTTATATT--------------------------- | 11005 |
| YJM1401 | 11460 | ATTCTCATAATTAAATTTATATCCTTCGGCCGGATTAATTTATTTTATTTA------TATTTATATTTATATT--------------------------- | 11526 |
| NCYC3594 | 10706 | ATTCTCATAATTAAATTTATATCCTTCGGCCGGATTAATTTATTA------------TATTTATATTTATATT--------------------------- | 10766 |
| YJM1078 | 10664 | ATTCTCATAATTAAATTTATATCCTTCGGCCGGATTAATTTATTA------------TATTTATATTTATATTTCTCCTTCCGGGGTTCCGGCTCCCGTG | 10751 |
| YJM1439 | 11522 | ACTCTCATAATTAAATTTATATCCTTCGGCCGGATTAATTTATTTTATTTA------TATTTATATTTATATT--------------------------- | 11588 |
| consensus | 14601 | \*.\*\*\*\*\*\*\*\*\*\*\*\*\*\*\*\*\*\*\*\*\*\*\*\*\*\*\*\*\*\*\*\*\*\*\*\*\*\*\*\*\*\*.------------\*\*\*\*\*\*\*\*\*\*\*\*\*\*\*\*--------------------------- | 14700 |
|
| S288C | 10896 | -------------------------------------------TATAGTGAATACCT-------------TTTTTAATATTTATTTTTAATA-TTTATTT | 10938 |
| YJM1388 | 11372 | -------------------------------------------TATAGTGAATACCT-------------TTTTTAATATTTATTTTTAATATTTTATTT | 11415 |
| YJM789 | 10967 | -------------------------------------------TATAGTGAATACCTTTTTTAATATTTATTTTTAATATTTATTTTTAATA-TTTATTT | 11022 |
| YJM1273 | 10988 | -------------------------------------------TATAGTGAATACCTTTTTTAATATTTATTTTTAATATTTATTTTTAATA-TTTATTT | 11043 |
| NCYC3585 | 11006 | -------------------------------------------TATAGTGAATACCTTTTTTAATATTTATTTTTAATATTTATTTTTAATA-TTTATTT | 11061 |
| YJM1401 | 11527 | -------------------------------------------TATAGTGAATACCTTTTTTAATATTTATTTTTAATATTTATTTTTAATA-TTTATTT | 11582 |
| NCYC3594 | 10767 | ---------------------------------------------------------------------------------------------------- | 10766 |
| YJM1078 | 10752 | GCCGGGCCCCGGAACTATTTATATTTATATTTATATTTATATTTATAGTGAATACCTTTTTTAATATTTATTTTTAATATTTATTTTTAATA-TTTATTT | 10850 |
| YJM1439 | 11589 | -------------------------------------TATATTTATAGTGAATACCTTTTTTAATATTTATTTTTAATATTTATTTTTAATA-TTTATTT | 11650 |
| consensus | 14701 | ---------------------------------------------------------------------------------------------------- | 14800 |
|
| S288C | 10939 | TTAATATTTTATTTTTAATAAAATATAATCTTGTAAGTAAGAAAAGAATTTCGGTGATTGGAACCTTGAAAGGATAAATTTCTTATT-----TATTATAA | 11033 |
| YJM1388 | 11416 | TTAATATTTTATTTTTAATAAAATATAATCTTGTAAGTAAGAAAAGAATTTCGGTGATTGGAACCTTGAAAGGATAAATTTCTTATT-----TATTATAA | 11510 |
| YJM789 | 11023 | TTAATATTTTATTTTTAATAAAATATAATCTTGTAAGTAAGAAAAGAATTTCGGTGATTGGAACCTTGAAAGGATAAATTTCTTATT-----TATTATAA | 11117 |
| YJM1273 | 11044 | TTAATATTTTATTTTTAATAAAATATAATCTTGTAAGTAAGAAAAGAATTTCGGTGATTGGAACCTTGAAAGGATAAATTTCTTATT-----TATTATAA | 11138 |
| NCYC3585 | 11062 | TTAATATTTTATTTTTAATAAAATATAATCTTGTAAGTAAGAAAAGAATTTCGGTGATTGGAACCTTGAAAGGATAAATTTCTTATT-----TATTATAA | 11156 |
| YJM1401 | 11583 | TTAATATTTTATTTTTAATAAAATATAATCTTGTAAGTAAGAAAAGAATTTCGGTGATTGGAACCTTGAAAGGATAAATTTCTTATT-----TATTATAA | 11677 |
| NCYC3594 | 10767 | ---------------------------------------------------------------------------------------------------- | 10766 |
| YJM1078 | 10851 | TTAATA-TTTATTTTTAATAAAATATAATCTTGTAAGTAAGAAAAGAATTTTGGTGATTGGAACCTTGAAAGGATAAATTTCTTATTTATTATATTATAA | 10949 |
| YJM1439 | 11651 | TTAATATTTTATTTTTAATAAAATATAATCTTGTAAGTAAGAAAAGAATTTCGGTGATTGGAACCTTGAAAGGATAAATTTCTTATT-----TATTATAA | 11745 |
| consensus | 14801 | ---------------------------------------------------.------------------------------------------------ | 14900 |
|
| S288C | 11034 | TATTTATATTAATAGTTCCGGGGCCCGGCCACGGGAGCCGGAACCCCGAAAGGAGTATTATTAAACATTTAATATATTATATTAATATTTAATTTAAATG | 11133 |
| YJM1388 | 11511 | TATTTATATTAATAGTTCCGGGGCCCGGCCACGGGAGCCGGAACCCCGAAAGGAGTATTATTAAACATTTAATATATTATATTAATATTTAATTTAAATG | 11610 |
| YJM789 | 11118 | TATTTATATTAATAGTTCCGGGGCCCGGCCACGGGAGCCGGAACCCCGAAAGGAGTATTATTAAACATTTAATATATTATATTAATATTTAATTTAAATG | 11217 |
| YJM1273 | 11139 | TATTTATATTA-----------------------------------------TAGTATTATTAAACATTTAATATATTATATTAATATTTAATTTAAATG | 11197 |
| NCYC3585 | 11157 | TATTTATATTAATAGTTCCGGGGCCCGGCCACGGGAGCCGGAACCCCGAAAGGAGTATTATTAAACATTTAATATATTATATTAATATTTAATTTAAATG | 11256 |
| YJM1401 | 11678 | TATTTATATTAATAGTTCCGGGGGCCGGCCACGGGAGCCGGAACCCCGAAAGGAGTATTATTAAACATTTAATATATTATATTAATATTTAATTTAAATG | 11777 |
| NCYC3594 | 10767 | ---------------------------------------------------------------------------------------------------- | 10766 |
| YJM1078 | 10950 | TATTTATATTA-----------------------------------------TGGTATTATTAAACATTTAATATATTATATTAATATTTAATTTAAATG | 11008 |
| YJM1439 | 11746 | TATTTATATTA-----------------------------------------TAGTATTATTAAACATTTAATATATTATATTAATATTTAATTTAAATG | 11804 |
| consensus | 14901 | -----------------------.----------------------------..---------------------------------------------- | 15000 |
|
| S288C | 11134 | ATTAATATATTATTATAATAATATTTATTTTATATTAAAATATTATAATTAATATATATATATTTATTTTAATAA------TATTATTATTATTATTATT | 11227 |
| YJM1388 | 11611 | ATTAATATATTATTATAATAATATTTATTTTATATTAAAATATTATAATTAATATATATATATTTATTTTAATAA------TATTATTATTATTATTATT | 11704 |
| YJM789 | 11218 | ATTAATATATTATTATAATAATATTTATTTTATATTAAAATATTATAATTAATATATATATATTTATTTTAATAATATTATTATTATTATTATTATTATT | 11317 |
| YJM1273 | 11198 | ATTAATATATTATTATAATAATATTTATTTTATATTAAAATATTATAATTAATATATATATATTTATTTTAATAATATTATTATTATTATTATTATTATT | 11297 |
| NCYC3585 | 11257 | ATTAATATATTATTATAATAATATTTATTTTATATTAAAATATTATAATTAATATATATATATTTATTTTAATAA------TATTATTATTATTATTATT | 11350 |
| YJM1401 | 11778 | ATTAATATATTATTATAATAATATTTATTTTATATTAAAATATTATAATTAATATATATATATTTATTTTAATAA------TATTATTATTATTATTATT | 11871 |
| NCYC3594 | 10767 | ---------------------------------------------------------------------------------------------------- | 10766 |
| YJM1078 | 11009 | ATTAATATATTATTATAATAATATTTATTTTATATTAAAATATTATAATTAATATATATATATTTATTTTAATAA------TATTATTATTATTATTATT | 11102 |
| YJM1439 | 11805 | ATTAATATATTATTATAATAATATTTATTTTATATTAAAATATTATAATTAATATATATATATTTATTTTAATAA------TATTATTATTATTATTATT | 11898 |
| consensus | 15001 | ---------------------------------------------------------------------------------------------------- | 15100 |
|
| S288C | 11228 | AAAATTATTATTTTTATAA----ATATATATATATATATATATATATTATTTTTATTCTTATATAAATTATAT-AAAAAAAATATATATAATATATAATT | 11322 |
| YJM1388 | 11705 | AAAATTATTATTTTTATAA----ATATATATATATATATATATATATTATTTTTATTCTTATATAAATTATATAAAAAAAAATATATATAATATATAATT | 11800 |
| YJM789 | 11318 | AAAATTATTATTTTTATAA----ATATATATATATATATATATATATTATTTTTATTCTTATATAAATTATAT---AAAAAATATATATAATATATAATT | 11410 |
| YJM1273 | 11298 | AAAATTATTATTTTTATAA----ATATATATATATATATATATATATTATTTTTATTCTTATATAAATTATAT---AAAAAATATATATAATATATAATT | 11390 |
| NCYC3585 | 11351 | AAAATTATTATTTTTATAA----ATATATATATATATATATATATATTATTTTTATTCTTATATAAATTATAT---AAAAAATATATATAATATATAATT | 11443 |
| YJM1401 | 11872 | AAAATTATTATTTTTATAAATATATATATATATATATATATATATATTATTTTTATTCTTATATAAATTATAT-AAAAAAAATATATATAATATATAATT | 11970 |
| NCYC3594 | 10767 | ---------------------------------------------------------------------------------------------------- | 10766 |
| YJM1078 | 11103 | AAAATTATTATTTTTATAA----ATATATATATATATATATATATATTATTTTTATTCTTATATAAATTATAT---ATAAAATATATATAATATATAATT | 11195 |
| YJM1439 | 11899 | AAAATTATTATTTTTATAA----ATATATATATATATATATATATATTATTTTTATTCTTATATAAATTATAT-AAAAAAAATATATATAATATATAATT | 11993 |
| consensus | 15101 | -----------------------------------------------------------------------------.---------------------- | 15200 |
|
| S288C | 11323 | AATTAATATATATTATTTAAATTATATATTATTTAAAATACTTT-----TTATATTATATCTTCTTTAAATTAAAATATAATTATTATTTATATTATAAT | 11417 |
| YJM1388 | 11801 | AATTAATATATATTATTTAAATTATATATTATTTAAAATACTTT-----TTATATTATATCTTCTTTAAATTAAAATATAATTATTATTTATATTATAAT | 11895 |
| YJM789 | 11411 | AATTAATATATATTATTTAAATTATATATTATTTAAAATACTTT-----TTATATTATATCTTCTTTAGATTAAAATATAATTATTATTTATATTATAAT | 11505 |
| YJM1273 | 11391 | AATTAATATATATTATTTAAATTATATATTATTTAAAATACTTT-----TTATATTATATCTTCTTTAGATTAAAATATAATTATTATTTATATTATAAT | 11485 |
| NCYC3585 | 11444 | AATTAATATATATTATTTAAATTATATATTATTTAAAATACTTT-----TTATATTATATCTTCTTTAGATTAAAATATAATTATTATTTATATTATAAT | 11538 |
| YJM1401 | 11971 | AATTAATATATATTATTTAAATTATATATTATTTAAAATACTTT-----TTATATTATATCTTCTTTAAATTAAAATATAATTATTATTTATATTATAAT | 12065 |
| NCYC3594 | 10767 | ---------------------------------------------------------------------------------------------------- | 10766 |
| YJM1078 | 11196 | AATTAATATATATTATTTAAATTATATATTATTTAAAATACTTTTTATATTATATTATATCTCCTTTAAATTAAAATATAATTATTATTTATATTATAAT | 11295 |
| YJM1439 | 11994 | AATTAATATATATTATTTAAATTATATATTATTTAAAATACTTT-----TTATATTATATCTTCTTTAAATTAAAATATAATTATTATTTATATTATAAT | 12088 |
| consensus | 15201 | --------------------------------------------------------------.-----.------------------------------- | 15300 |
|
| S288C | 11418 | TATTTATGAAATATTATTATTAAAATAAAAAAGAGGTTTAGACTATATATTTATTATTTATAAACTTATTATATTATTTATTATTAATAGT--------- | 11508 |
| YJM1388 | 11896 | TATTTATGAAATATTATTATTAAAATAAAAAAGAGGTTTAGACTATATATTTATTATTTATAAACTTATTATATTATTTATTATTAATAGT--------- | 11986 |
| YJM789 | 11506 | TATTTATGAAATATTATTCTTAAAATAAAAAAGAGGTTTAGACTATATATTTATTATTTATAAACTTATTATATTATTTATTATTAATAGT--------- | 11596 |
| YJM1273 | 11486 | TATTTATGAAATATTATTATTAAAATAAAAAAGAGGTTTAGACTATATATTTATTATTTATAAACTTATTATATTATTTATTATTAATAGT--------- | 11576 |
| NCYC3585 | 11539 | TATTTATGAAATATTATTATTAAAATAAAAAAGAGGTTTAGACTATATATTTATTATTTATAAACTTATTATATTATTTATTATTAATAGT--------- | 11629 |
| YJM1401 | 12066 | TATTTATGAAATATTATTATTAAAATAAAAAAGAGGTTTAGACTATATATTTATTATTTATAAACTTATTATATTATTTATTATTAATAGT--------- | 12156 |
| NCYC3594 | 10767 | ---------------------------------------------------------------------------------------------------- | 10766 |
| YJM1078 | 11296 | TATTTATGAAATATTATTATTAAAATAAATAAGAGGTTTAGACTATATATTTATTATTTATAAACTTATTATATTATTTATTATTAATAGT--------- | 11386 |
| YJM1439 | 12089 | TATTTATGAAATATTATTCTTAAAATAAAAAAGAGGTTTAGACTATATATTTATTATTTATAAACTTATTATATTATTTATTATTAATAGTGAACACCTT | 12188 |
| consensus | 15301 | ------------------.----------.---------------------------------------------------------------------- | 15400 |
|
| S288C | 11509 | --------------------------------TCCGGGGCCCGGCCACGGGAGCCGGAACCCCGAAAGGAGAAATAAATAAAATAAAAAA-TAATAAATA | 11575 |
| YJM1388 | 11987 | --------------------------------TCCGGGGGCCGGCCACGGGAGCCGGAACCCCGAAAGGAGAAATAAATAAAATAAAAAA-TAATAAATA | 12053 |
| YJM789 | 11597 | --------------------------------TCCGGGGCCCGGCCACGGGAGCCGGAACCCCGAAAGGAGAAATAAATAAAATAAAAAA-TAATAAATA | 11663 |
| YJM1273 | 11577 | --------------------------------TCCGGGGCCCGGCCACGGGAGCCGGAACCCCGAAAGGAGAAATAAATAAAATAAAAAA-TAATAAATA | 11643 |
| NCYC3585 | 11630 | --------------------------------TCCGGGGCCCGGCCACGGGAGCCGGAACCCCGAAAGGAGAAATAAATAAAATAAAAAA-TAATAAATA | 11696 |
| YJM1401 | 12157 | --------------------------------TCCGGGGCCCGGCCACGGGAGCCGGAACCCCGAAAGGAGAAATAAATAAAATAAAAAA-TAATAAATA | 12223 |
| NCYC3594 | 10767 | -------------------------------------------------------------------------ATAAATAAAATAAAAAAGTAATAAATA | 10793 |
| YJM1078 | 11387 | --------------------------------TCCGGGGCCCGGCCACGGGAGCCGGAACCCCGAAAGGAGAAATAAATAAAATAAAAAAGTAATAAATA | 11454 |
| YJM1439 | 12189 | TATTTAAAGGTGTGAACCCCGCAAGGGAGGAGTTCCGGGCCCGGCCACGGGAGCCGGAACCCCGAAAGGAGAAATAAATAAAATAAAAAA-TAATAAATA | 12287 |
| consensus | 15401 | ---------------------------------.-.---.---------------------------------\*\*\*\*\*\*\*\*\*\*\*\*\*\*\*\*\*-\*\*\*\*\*\*\*\*\* | 15500 |
|
| S288C | 11576 | TTAATATTATTAAATATTAT--------------------------------TTATAATAAATATTAATATTATTAAATATTATTCATATTAATAAATTT | 11643 |
| YJM1388 | 12054 | TTAATATTATTAAATATTAT--------------------------------TTATAATAAATATTAATATTATTAAATATTATTCATATTAATAAATTT | 12121 |
| YJM789 | 11664 | TTAATATTATTAAATATTAT--------------------------------TTATAATAAATATTAATATTATTAAATATTATTCATATTAATAAATTT | 11731 |
| YJM1273 | 11644 | TTAATATTATTAAATATTAT--------------------------------TTATAATAAATATTAATATTATTAAATATTATTCATATTAATAAATTT | 11711 |
| NCYC3585 | 11697 | TTAATATTATTAAATATTAT--------------------------------TTATAATAAATATTAATATTATTAAATATTATTCATATTAATAAATTT | 11764 |
| YJM1401 | 12224 | TTAATATTATTAAATATTATTTATAATAAATATTAATATTATTAAATATTATTTATAATAAATATTAATATTATTAAATATTATTCATATTAATAAATTT | 12323 |
| NCYC3594 | 10794 | TTAATATTATTAAATATTAT--------------------------------TTATAATAAATATTAATATTATTAAATATTATTTATATTAATAAATTT | 10861 |
| YJM1078 | 11455 | TTAATATTATTAAATATTAT--------------------------------TTATAATAAATATTAATATTATTAAATATTATTTATATTAATAAATTT | 11522 |
| YJM1439 | 12288 | TTAATATTATTAAATATTAT--------------------------------TTATAATAAATATTAATATTATTAAATATTATTCATATTAATAAATTT | 12355 |
| consensus | 15501 | \*\*\*\*\*\*\*\*\*\*\*\*\*\*\*\*\*\*\*\*--------------------------------\*\*\*\*\*\*\*\*\*\*\*\*\*\*\*\*\*\*\*\*\*\*\*\*\*\*\*\*\*\*\*\*\*.\*\*\*\*\*\*\*\*\*\*\*\*\*\* | 15600 |
|
| S288C | 11644 | TATTATTATTTGTAATATATTAAATATTAATAATATATATATTATTTATTATAATGAAAACCTATCCTATATTATCCTATCATATAATATCATATCATAT | 11743 |
| YJM1388 | 12122 | TATTATTATTTGTAATATATTAAATATTAATAATATATATATTATTTATTATAATGAAAACCTATCCTAAATTATCCTATCATATAATATCATATCATAT | 12221 |
| YJM789 | 11732 | TATTATTATTTGTAATATATTAAATATTAATAATATATATATTATTTATTATAATGAAAACCTATCCTAAATTATCCTATCATATAATATCATATCATAT | 11831 |
| YJM1273 | 11712 | TATTATTATTTGTAATATATTAAATATTAATAATATATATATTATTTATTATAATGAAAACCTATCCTAAATTATCCTATCATATAATATCATATCATAT | 11811 |
| NCYC3585 | 11765 | TATTATTATTTGTAATATATTAAATATTAATAATATATATATTATTTATTATAATGAAAACCTATCCTAAATTATCCTATCATATAATATCATATCATAT | 11864 |
| YJM1401 | 12324 | TATTATTATTTGTAATATATTAAATATTAATAATATATATATTATTTATTATAATGAAAACCTATCCTAAATTATCCTATCATATAATATCATATCATAT | 12423 |
| NCYC3594 | 10862 | TATTATTATTTGTAATATATTAAATATTAATAATATATATATTATTTATTATAATGAAAACCTATCATAAATTATCCTATCATATAATATCATATCATAT | 10961 |
| YJM1078 | 11523 | TATTATTATTTGTAATATATTAAATATTAATAATATATATATTATTTATTATAATGAAAACCTATCATAAATTATCCTATCATATAATATCATATCATAT | 11622 |
| YJM1439 | 12356 | TATTATTATTTGTAATATATTAAATATTAATAATATATATATTATTTATTATAATGAAAACCTATCCTAAATTATCCTATCATATAATATCATATCATAT | 12455 |
| consensus | 15601 | \*\*\*\*\*\*\*\*\*\*\*\*\*\*\*\*\*\*\*\*\*\*\*\*\*\*\*\*\*\*\*\*\*\*\*\*\*\*\*\*\*\*\*\*\*\*\*\*\*\*\*\*\*\*\*\*\*\*\*\*\*\*\*\*\*\*.\*\*.\*\*\*\*\*\*\*\*\*\*\*\*\*\*\*\*\*\*\*\*\*\*\*\*\*\*\*\*\*\* | 15700 |
|
| S288C | 11744 | TATATTATATCTTATTATATGATATATAAAGTATTCACTCTATATGAGGTTATGATTATTATATAAATCTTATTTTATTTT----TATTTTTATTTGGAC | 11839 |
| YJM1388 | 12222 | TATATTATATCTTATTATATGATATATAAAGTATTCACTCTATATGAGGTTATGATTATTATATAAATCTTATTTTATTTT----TATTTTTATTTGGAC | 12317 |
| YJM789 | 11832 | TATATCATATCTTATTATATGATATATAAAGTATTCACTCTATATGAAGTTATGATTATTATATAAATCTTATTTTATTTT----TATTTTTATTTGGAC | 11927 |
| YJM1273 | 11812 | TATATCATATCTTATTATATGATATATAAAGTATTCACTCTATATGAAGTTATGATTATTATATAAATCTTATTTTATTTT----TATTTTTATTTGGAC | 11907 |
| NCYC3585 | 11865 | TATATTATATCTTATTATATGATATATAAAGTATTCACTCTATATGAGGTTATGATTATTATATAAATCTTATTTTA-TTT----TATTTTTATTTGGAC | 11959 |
| YJM1401 | 12424 | TATATTATATCTTATTATATGATATATAAAGTATTCACTCTATATGAGGTTATGATTATTATATAAATCTTATTTTATTTT----TATTTTTATTTGGAC | 12519 |
| NCYC3594 | 10962 | TATATCATATCTTATTATATGATATATAAAGTATTCACTCTATATGAAGTTATGATTATTATATAAATCTTATTTTATTTTA---TTTTTTTATTTGGAC | 11058 |
| YJM1078 | 11623 | TATATCATATCTTATTATATGATATATAAAGTATTCACTCTATATGAAGTTATGATTATTATATAAATCTTATCTTATTTTA---TTTTTTTATTTGGAC | 11719 |
| YJM1439 | 12456 | TATATTATATCTTATTATATGATATATAAAGTATTCACTCTATATGAGGTTATGATTATTATATAAATCTTATTTTATTTTATTTTATTTTTATTTGGAC | 12555 |
| consensus | 15701 | \*\*\*\*\*.\*\*\*\*\*\*\*\*\*\*\*\*\*\*\*\*\*\*\*\*\*\*\*\*\*\*\*\*\*\*\*\*\*\*\*\*\*\*\*\*\*.\*\*\*\*\*\*\*\*\*\*\*\*\*\*\*\*\*\*\*\*\*\*\*\*\*.\*\*\*-\*\*\*----\*.\*\*\*\*\*\*\*\*\*\*\*\*\* | 15800 |
|
| S288C | 11840 | TAATAATAATTATAATAATAATTATTGATATGTTCTAATATTAATAAATACATATTTATATTATAATATAAATATTCATTTCTTACTAATTAATAAAAAG | 11939 |
| YJM1388 | 12318 | TAATAATAATTATAATAATAATTATTGATATGTTCTAATATTAATAAATACATATTTATATTATAATATAAATATTCATTTCTTACTAATTAATAAAAAG | 12417 |
| YJM789 | 11928 | TAATAATAATTATAATAATAATTATTGATATGTTCTAATATTAATAAATACATATCTATATTATAATATAAATATTCATTTCTTAC----TAATAAAAAG | 12023 |
| YJM1273 | 11908 | TAATAATAATTATAATAATAATTATTGATATGTTCTAATATTAATAAATACATATCTATATTATAATATAAATATTCATTTCTTAC----TAATAAAAAG | 12003 |
| NCYC3585 | 11960 | TAATAATAATTATAATAATAATTATTGATATGTTCTAATATTAATAAATACATATTTATATTATAATATAAATATTCATTTCTTAC----TAATAAAAAG | 12055 |
| YJM1401 | 12520 | TAATAATAATTATAATAATAATTATTGATATGTTCTAATATTAATAAATACATATTTATATTATAATATAAATATTCATTTCTTACTAATTAATAAAAAG | 12619 |
| NCYC3594 | 11059 | TAATAATAATTATAATAATAATTATTGATATGTTCTAATATTAATAAATAAATATTTATATTATAATATAGATATTCATTTCTTACT----AATAAAAAG | 11154 |
| YJM1078 | 11720 | TAATAATAATTATAATAATAATTATTGATATGTTCTAATATTAATAAATAAATATTTATATTATAATATAGATATTCATTTCTTACT----AATAAAAAG | 11815 |
| YJM1439 | 12556 | TAATAATAATTATAATAATAATTATTGATATGTTCTAATATTAATAAATACATATTTATATTATAATATAAATATTCATTTCTTACT----AATAAAAAG | 12651 |
| consensus | 15801 | \*\*\*\*\*\*\*\*\*\*\*\*\*\*\*\*\*\*\*\*\*\*\*\*\*\*\*\*\*\*\*\*\*\*\*\*\*\*\*\*\*\*\*\*\*\*\*\*\*\*.\*\*\*\*.\*\*\*\*\*\*\*\*\*\*\*\*\*\*.\*\*\*\*\*\*\*\*\*\*\*\*\*\*\*-----\*\*\*\*\*\*\*\*\* | 15900 |
|
| S288C | 11940 | TTTTTATATTCATTATAATATAAATATATAAATATATATAAATATTTTAATAATTATAATTATATTAAGATATTATAAATATATATTTA---TTTTTTTT | 12036 |
| YJM1388 | 12418 | TTTTTATATTCATTATAATATAAATATATAAATATATATAAATATTTTAATAATTATAATTATATTAAGATATTATAAATATATATTTATTTTTTTTTTT | 12517 |
| YJM789 | 12024 | TTTTTATATTCATTATAATATAAATATATAAATATATATAAATATTTTAATAATTATAATTATATTAAGATATTATAAATATATATTTA---T-TTTTTT | 12119 |
| YJM1273 | 12004 | TTTTTATATTCATTATAATATAAATATATAAATATATATAAATATTTTAATAATTATAATTATATTAAGATATTATAAATATATATTTA---T-TTTTTT | 12099 |
| NCYC3585 | 12056 | TTTTTATATTCATTATAATATAAATATATAAATATATATAAATATTTTAATAATTATAATTATATTAATATATTATAAATATATATTTA-----TTTTTT | 12150 |
| YJM1401 | 12620 | TTTTTATATTCATTATAATATAAATATATAAATATATATAAATATTTTAATAATTATAATTATATTAAGATATTATAAATATATATTTAT-TT-TTTTTT | 12717 |
| NCYC3594 | 11155 | TTTTTATATTCATTATAATATAAATATATAAATATATATAAATA-TTTAATGATTATAATTATATTAAGATATTATAAATATATATTTA---TTTTTTAT | 11250 |
| YJM1078 | 11816 | TTTTTATATTCATTATAATATAAATATATAAATATATATAAATA-TTTAATGATTATAATTATATTAAGATATTATAAATATATATTTA---TTTTTTAT | 11911 |
| YJM1439 | 12652 | TTTTTATATTCATTATAATATAAATATATAAATATATATAAATATTTTAATAATTATAATTATATTAAGATATTATAAATATATATTTA--TTTTTTTTT | 12749 |
| consensus | 15901 | \*\*\*\*\*\*\*\*\*\*\*\*\*\*\*\*\*\*\*\*\*\*\*\*\*\*\*\*\*\*\*\*\*\*\*\*\*\*\*\*\*\*\*\*-\*\*\*\*\*\*.\*\*\*\*\*\*\*\*\*\*\*\*\*\*\*\*.\*\*\*\*\*\*\*\*\*\*\*\*\*\*\*\*\*\*\*\*-----\*\*\*\*.\* | 16000 |
|
| S288C | 12037 | TATA-----AAATAAATAAATAAATAAATAATTAATATTTTTATATTATAACTTATTTTT------ATAATAATAATAAGTATTTTAT------------ | 12113 |
| YJM1388 | 12518 | TATA-----AAATAAATAAATAAATAATTAATTAATATTTTTATATTATAACTTATTTTT------ATAATAATAATAAGTATTTTA------------- | 12593 |
| YJM789 | 12120 | TATA-----AAATAAATAAATAAATAATTAATTAATATTTTTATATTATAACTTATTTTT------ATAATAATAATAAGTATTTTA------------- | 12195 |
| YJM1273 | 12100 | TATA-----AAATAAATAAATAAATAATTAATTAATATTTTTATATTATAACTTATTTTT------ATAATAATAATAAGTATTTTA------------- | 12175 |
| NCYC3585 | 12151 | TATA-----AAATAAATAAATAAATAATTAATTAATATTTTTATATTATAACTTATTTTT------ATAATAATAATAAGTATTTTA------------- | 12226 |
| YJM1401 | 12718 | TATA-----AAATAAATAAATAAATAATTAATTAATATTTTTATATTATAACTTATTTTT------ATAATAATAATAAGTATTTTA------------- | 12793 |
| NCYC3594 | 11251 | AAAATAAATAAATAAATAAATAAATAATTAATTAATATTTTTATATTATAACTTATTTTTATAATAATAATAATAATAAGTATTTTATTTTTATTATATT | 11350 |
| YJM1078 | 11912 | AAAATAAATAAATAAATAAATAAATAATTAATTAATATTTTTATATTATAACTTATTTTTATAATAATAATAATAATAAGTATTTTATTTTTATTATATT | 12011 |
| YJM1439 | 12750 | TATA-----AAATAAATAAATAAATAAATAATTAATATTTTTATATTATAACTTATTTTT------ATAATAATAATAAGTATTTTAT------------ | 12826 |
| consensus | 16001 | .\*.\*-----\*\*\*\*\*\*\*\*\*\*\*\*\*\*\*\*\*\*.\*\*\*\*\*\*\*\*\*\*\*\*\*\*\*\*\*\*\*\*\*\*\*\*\*\*\*\*\*\*\*\*------\*\*\*\*\*\*\*\*\*\*\*\*\*\*\*\*\*\*\*\*\*------------- | 16100 |
|
| S288C | 12114 | ------------------------------------------TTTTTATTATATTATTAT-TTATATAAT--TATATATATATTAATTTCAATTTAATTA | 12168 |
| YJM1388 | 12594 | ------------------------------------------TTTTTATTATATTATTAT-TTATATAAT--TATATATATATTAATTTCAATTTAATTA | 12648 |
| YJM789 | 12196 | ------------------------------------------TTTTTATTATATTATTATATTATATAATTATATATATATATTAATTTTAATTTAATTA | 12253 |
| YJM1273 | 12176 | ------------------------------------------TTTTTATTATATTATTATATTATATAATTATATATATATATTAATTTTAATTTAATTA | 12233 |
| NCYC3585 | 12227 | ------------------------------------------TTTTTATTATATTATTAT-TTATATAATTATATATATATATTAATTTCAATTTAATTA | 12283 |
| YJM1401 | 12794 | ------------------------------------------TTTTTATTATATTATTAT-TTATATAAT--TATATATATATTAATTTCAATTTAATTA | 12848 |
| NCYC3594 | 11351 | ATTATATTATAACTTATTTTTATAATAATAATAAGTATTTTATTTTTATTATATTATTATATTATATAATTATATATATATATTAATTTCAATTTAATTA | 11450 |
| YJM1078 | 12012 | ATTATATTATAACTTATTTTTATAATAATAATAAGTATTTTATTTTTATTATATTATTATATTATATAATTATATATATATATTAATTTCAATTTAATTA | 12111 |
| YJM1439 | 12827 | ------------------------------------------TTTTTATTATATTATTAT-TTATATAAT--TATATATATATTAATTTCAATTTAATTA | 12881 |
| consensus | 16101 | ------------------------------------------\*\*\*\*\*\*\*\*\*\*\*\*\*\*\*\*\*\*-\*\*\*\*\*\*\*\*\*--\*\*\*\*\*\*\*\*\*\*\*\*\*\*\*\*\*.\*\*\*\*\*\*\*\*\*\* | 16200 |
|
| S288C | 12169 | ATTAATTAATTGGTATTTGGCATATAATATCAATTAATTGTAATTCTTATAAGAATTAATTAATTAATATGCTTTTTATATAATTTATACTTTTATATTT | 12268 |
| YJM1388 | 12649 | ATTAATTAATTGGTATTTGGCATATAATATCAATTAATTGTAATTCTTATAAGAATTAATTAATTAATATGCTTTTTATATAATTTATACTTTTATATTT | 12748 |
| YJM789 | 12254 | ATTAATTAATTGGTATTTGGCATATAATATCAATTAATTGTAATTCTTATAAG----AATTAATTAATATGCTTTTTATATAATTTATACTTTTATATTT | 12349 |
| YJM1273 | 12234 | ATTAATTAATTGGTATTTGGCATATAATATCAATTAATTGTAATTCTTATAAG----AATTAATTAATATGCTTTTTATATAATTTATACTTTTATATTT | 12329 |
| NCYC3585 | 12284 | ATTAATTAATTGGTATTTGGCATATGAGATCAATTAATTGTAATTCTTATAAG----AATTAATTAATATGCTTTTTATATAATTTATACTTTTATATTT | 12379 |
| YJM1401 | 12849 | ATTAATTAATTGGTATTTGGCATATAATATCAATTAATTGTAATTCTTATAAGAATTAATTAATTAATATGCTTTTTATATAATTTATACTTTTATATTT | 12948 |
| NCYC3594 | 11451 | ATTAATTAATTGGTATTTAGCATATGAGATCAATTAATTGTAATTCTTATAAG----AATTAATTAATATGCTTTTTATATAATTTATACTTTTATATTT | 11546 |
| YJM1078 | 12112 | ATTAATTAATTGGTATTTAGCATATGAGATCAATTAATTGTAATTCTTATAAG----AATTAATTAATATGCTTTTTATATAATTTATACTTTTATATTT | 12207 |
| YJM1439 | 12882 | ATTAATTAATTGGTATTTGGCATATGAGATCAATTAATTGTAATTCTTATAAG----AATTAATTAATATGCTTTTTATATAATTTATACTTTTATATTT | 12977 |
| consensus | 16201 | \*\*\*\*\*\*\*\*\*\*\*\*\*\*\*\*\*\*.\*\*\*\*\*\*.\*.\*\*\*\*\*\*\*\*\*\*\*\*\*\*\*\*\*\*\*\*\*\*\*\*\*----\*\*\*\*\*\*\*\*\*\*\*\*\*\*\*\*\*\*\*\*\*\*\*\*\*\*\*\*\*\*\*\*\*\*\*\*\*\*\*\*\*\*\* | 16300 |
|
| S288C | 12269 | CTCCTTCCGGGGTTCCGGCTCCCGTGGCCGGGCCCCGGAACTATTATTATTATT----T-TTATTTATTTATTATTAAAATATAATAATAAATAGTCCGG | 12363 |
| YJM1388 | 12749 | ----------------------------------------ATATTATTATTATT----TATTATTTATTTATTATTAAAATATAATAATAAATAGTCCGG | 12804 |
| YJM789 | 12350 | A----------------------------------------TATTATTATTATT----T-TTATTTATTTATTATTAAAATATAATAATAAATAGTCCGG | 12404 |
| YJM1273 | 12330 | A----------------------------------------TATTATTATTATT----T-TTATTTATTTATTATTAAAATATAATAATAAAT------- | 12377 |
| NCYC3585 | 12380 | A----------------------------------------TATTATTATTATT----T-TTATTTATTTATTATTAAAATATAATAATAAATAGTCCGG | 12434 |
| YJM1401 | 12949 | A----------------------------------------TATTATTATTATT----T-TTATTTATTTATTATTAAAATATAATAATAAATAGTCCGG | 13003 |
| NCYC3594 | 11547 | CTCCTTCCGGGGTTCCGGCTCCCGTGGCCGGGCCCCGGAACTATTATTATTATTTTTAT-TTATTTATTTATTATTAAAATATAATAATAAATA------ | 11639 |
| YJM1078 | 12208 | CTCCTTCCGGGGTTCCGGCTCCCGTGGCCGGGCCCCGGAACTATTATTATTATTTTTAT-TTATTTATTTATTATTAAAATATAATAATAAATA------ | 12300 |
| YJM1439 | 12978 | CTCCTTTCGGGGTTCCGGCTCCCGTGGCCGGG-GCCGGAACTATTATTATTATT----T-TTATTTATTTATTATTAAAATATAATAATAAATA------ | 13065 |
| consensus | 16301 | .-----.--------------------------.------.\*\*\*\*\*\*\*\*\*\*\*\*\*----\*-\*\*\*\*\*\*\*\*\*\*\*\*\*\*\*\*\*\*\*\*\*\*\*\*\*\*\*\*\*\*\*\*\*------- | 16400 |
|
| S288C | 12364 | CCCGCCCCGCGGGGCGGACGCCGGAGGAGAATTATATTTTTATATAATAATTTATATTTCTATATATATATATATATATTATATATAAATATTATTATAT | 12463 |
| YJM1388 | 12805 | CCCGCCCCGCGGGGCGGACGCCGGAGGAGAATTATATTTTTATATAATAATTTATATTTCTATATATATATATATATATTATATATAAATATTATTATAT | 12904 |
| YJM789 | 12405 | CCCGCCCCGCGGGGCGGACGCCGGAGGAGAATTATATTTTTATATAATAATTTATATTTCTATATATATATATATATATTATATATAAATATTATTATAT | 12504 |
| YJM1273 | 12378 | ---------------------------AAAATTATATTTTTATATAATAATTTATATTTCTATATATATATATATATATTATATATAAATATTATTATAT | 12450 |
| NCYC3585 | 12435 | CCCGCCCCGCGGGGCGGACGCCGGAGGAGAATTATATTTTTATATAATAATTTATATTTCTATATATATATATATATATTATATATAAATATTATTATAT | 12534 |
| YJM1401 | 13004 | CCCGCCCCGCGGGGCGGACGCCGGAGGAGAATTATATTTTTATATAATAATTTATATTTCTATATATATATATATATATTATATATAAATATTATTATAT | 13103 |
| NCYC3594 | 11640 | ------------------------AAATTATATATATTTTTATATAATAATTTATATTTC--TATATATATATATATATTATATATAAATATTATTATAT | 11713 |
| YJM1078 | 12301 | ------------------------AAATTATATATATTTTTATATAATAATTTATATTTC--TATATATATATATATATTATATATAAATATTATTATAT | 12374 |
| YJM1439 | 13066 | ----------------------------AAATTATATTTTTATATAATAATTTATATTTCTATATATATATATATATATTATATATAAATATTATTATAT | 13137 |
| consensus | 16401 | -------------------------....\*..\*\*\*\*\*\*\*\*\*\*\*\*\*\*\*\*\*\*\*\*\*\*\*\*\*\*\*\*--\*\*\*\*\*\*\*\*\*\*\*\*\*\*\*\*\*\*\*\*\*\*\*\*\*\*\*\*\*\*\*\*\*\*\*\*\*\* | 16500 |
|
| S288C | 12464 | ATATTTTTATATATATTATAATTATATTCATTAATATTTTATTATAGTGGT--GGGGTCCCAATTATTATTTTCAATAATAATTTATCATGGGACCCGGA | 12561 |
| YJM1388 | 12905 | ATATTTTTATATATATTATAATTATATTCATTAATATTTTATTATAGTGGTG-GGGGTCCCAATTATTATTTTCAATAATAATTTATCATGGGACCCGGA | 13003 |
| YJM789 | 12505 | ATATTTTTATATATATTATAATTATATTCATTAATATTTTATTATAGTGGTG-GGGGTCCCAATTATTATTTTCAATAATAATTTATCATGGGACCCGGA | 12603 |
| YJM1273 | 12451 | ATATTTTTATATATATTATAATTATATTCATTAATATTTTATTATAGTGGTG-GGGGTCCCAATTATTATTTTCAATAATAATTTATCATGGGACCCGGA | 12549 |
| NCYC3585 | 12535 | ATATTTTTATATATATTATAATTATATTCATTAATATTTTATTATAATGGTG-GGGGTCCCAATTATTATTTTCAATAATAATTTATCAAGGGACCCGGA | 12633 |
| YJM1401 | 13104 | ATATTTTTATATATATTATAATTATATTCATTAATATTTTATTATAGTGGT--GGGGTCCCAATTATTATTTTCAATAATAATTTATCATGGGACCCGGA | 13201 |
| NCYC3594 | 11714 | ATATTTTTATATATATTATAATTATATTCATTAATATTTTATTATAGTGGTGGGGGGTCCCAATTATTATTTTCAATAATAATTTATCATGGGACCCGGA | 11813 |
| YJM1078 | 12375 | ATATTTTTATATATATTATAATTATATTCATTAATATTTTATTATAGTGGT-GGGGGTCCCAATTATTATTTTCAATAATAATTTATCAAGGGACCCGGA | 12473 |
| YJM1439 | 13138 | ATATTTTTATATATATTATAATTATATTCATTAATATTTTATTATAGTGGTG-GGGGGCCCAATTATTATTTTCAATAATAATTTATCATGGGACCCGGA | 13236 |
| consensus | 16501 | \*\*\*\*\*\*\*\*\*\*\*\*\*\*\*\*\*\*\*\*\*\*\*\*\*\*\*\*\*\*\*\*\*\*\*\*\*\*\*\*\*\*\*\*\*\*.\*\*\*\*--\*\*\*\*.\*\*\*\*\*\*\*\*\*\*\*\*\*\*\*\*\*\*\*\*\*\*\*\*\*\*\*\*\*\*\*.\*\*\*\*\*\*\*\*\*\* | 16600 |
|
| S288C | 12562 | TATCTTCTTGTTTTTATTTATTATTTTATTAAATTTATTTTAATTATTTATTTATAATTTATATTAT--------------------------------- | 12628 |
| YJM1388 | 13004 | TATCTTCTTGTTTTTATTTATTATTTTATTAAATTTATTTTAATTATTTATTTATAATTTATATTAT--------------------------------- | 13070 |
| YJM789 | 12604 | TATCTTCTTGTTTTTATTTATTATTTTATTAAATTTATTTTAATTATTTATTTATAATTTATATTAT--------------------------------- | 12670 |
| YJM1273 | 12550 | TATCTTCTTGTTTTTATTTATTATTTTATTAAATTTATTTTAATTATTTATTTATAATTTATATTAT--------------------------------- | 12616 |
| NCYC3585 | 12634 | TATCTTCTTGTTTTTATTTATTATTTTATTAAATTTATTTTAATTATTTATTTATAATTTATATTAT--------------------------------- | 12700 |
| YJM1401 | 13202 | TATCTTCTTGTTTTTATTTATTATTTTTTTAAATTTATTTTAATTATTTATTTATAATTTATATTAT--------------------------------- | 13268 |
| NCYC3594 | 11814 | TATCTTCTTGTTTTTATTTATTATTTTATTAAATTTATTTTAATTATTTATTTATAATTTATATTAT--------------------------------- | 11880 |
| YJM1078 | 12474 | TATCTTCTTGTTTTTATTTATTATTTTATTAAATTTATTTTAATTATTTATTTATAATTTATATTAT--------------------------------- | 12540 |
| YJM1439 | 13237 | TATCTTCTTGTTTTTATTTATTATTTTATTAAATTTATTTTAATTATTTATTTATAATTTATATTATACTCCTTCTTAATTAAAGATAAAAAGGGGTTCG | 13336 |
| consensus | 16601 | \*\*\*\*\*\*\*\*\*\*\*\*\*\*\*\*\*\*\*\*\*\*\*\*\*\*\*.\*\*\*\*\*\*\*\*\*\*\*\*\*\*\*\*\*\*\*\*\*\*\*\*\*\*\*\*\*\*\*\*\*\*\*\*\*\*\*--------------------------------- | 16700 |
|
| S288C | 12629 | --------------------------------------------------------------------ACAATTTATTATTTCGTTAATACCTTTATTTA | 12660 |
| YJM1388 | 13071 | --------------------------------------------------------------------ACAATTTATTATTTCGTTAATACCTTTATTTA | 13102 |
| YJM789 | 12671 | --------------------------------------------------------------------ACAATTTATTATTTCGTTAATACCTTTATTTA | 12702 |
| YJM1273 | 12617 | --------------------------------------------------------------------ACAATTTATTATTTCGTTAATACCTTTATTTA | 12648 |
| NCYC3585 | 12701 | --------------------------------------------------------------------ACAATTTATTATTTCGTTAATACCTTTATTTA | 12732 |
| YJM1401 | 13269 | --------------------------------------------------------------------ACAATTTATTATTTCGTTAATACCTTTATTTA | 13300 |
| NCYC3594 | 11881 | --------------------------------------------------------------------ACAATTTATTATTTCGTTAATACCTTTATTTA | 11912 |
| YJM1078 | 12541 | --------------------------------------------------------------------ACAATTTATTATTTCGTTAATATCTTTATTTA | 12572 |
| YJM1439 | 13337 | GTCCCCCTCCCGTTAGGGAGGGGTCCCTCACTCCTTCGGGGTCCGCCCCCCCCGCGGGGGCGGGCCGGACTATTTATTATTTCGTTAATACCTTTATTTA | 13436 |
| consensus | 16701 | --------------------------------------------------------------------\*\*.\*\*\*\*\*\*\*\*\*\*\*\*\*\*\*\*\*\*\*.\*\*\*\*\*\*\*\*\* | 16800 |
|
| S288C | 12661 | TATTATATAATATATTATATTATTATAATA-TATTTATTGATTATATTAATACATTTAACTAATGTGTGCTCTATATTTATTGAATAGTTTGGTTCTTAT | 12759 |
| YJM1388 | 13103 | TATTATATAATATATTATATTATTATAATA-TATTTATTGATTATATTAATACATTTAACTAATGTGTGCTCTATATTTATTGAATAGTTTGGTTATTAT | 13201 |
| YJM789 | 12703 | TATTATATAATATATTATATTATTATAATATTATTTATTGATTATATTAATACATTTAACTAATGTGTGCTCTATATTTATTGAATAGTTTGGTTCTTAT | 12802 |
| YJM1273 | 12649 | TATTATATAATATATTATATTATTATAATATTATTTATTGATTATATTAATACATTTAACTAATGTGTGCTCTATATTTATTGAATAGTTTGGTTCTTAT | 12748 |
| NCYC3585 | 12733 | TATTATATAATATATTATATTATTATAATA-TATTTATTGATTATATTAATACATTTAACTAATGTGTGCTCTATATTTATTGAATAGTTTGGTTATTAT | 12831 |
| YJM1401 | 13301 | TATTATATAATATATTATATTATTATAATA-TATTTATTGATTATATTAATACATTTAACTAATGTGTGCTCTATATTTATTGAATAGTTTGGTTCTTAT | 13399 |
| NCYC3594 | 11913 | TATTATATAATATATTATATTATTATAATA-TATTTATTGATTATATTAATACATTTAACTAATGTGTGCTCTATATTTATTGAATAGTTTGGTTCTTAT | 12011 |
| YJM1078 | 12573 | TATTATATAATATATTATATTATTATAATA-TATTTATTGATTATATTAATACATTTAACTAATGTGTGCTCTATATTTATTGAATAGTTTGGTTCTTAT | 12671 |
| YJM1439 | 13437 | TATTATATAATATATTATATTATTATAATA-TATTTATTGATTATATTAATACATTTAACTAATGTGTGCTCTATATTTATTGAATAGTTTGGTTCTTAT | 13535 |
| consensus | 16801 | \*\*\*\*\*\*\*\*\*\*\*\*\*\*\*\*\*\*\*\*\*\*\*\*\*\*\*\*\*\*-\*\*\*\*\*\*\*\*\*\*\*\*\*\*\*\*\*\*\*\*\*\*\*\*\*\*\*\*\*\*\*\*\*\*\*\*\*\*\*\*\*\*\*\*\*\*\*\*\*\*\*\*\*\*\*\*\*\*\*\*\*\*\*\*.\*\*\*\* | 16900 |
|
| S288C | 12760 | CACCCACCCCCTCCCCCTATT-----------------------------------ACGTCTCCGAGGTCCCGGTTTCGTAAGAAACCGGGACTTATATA | 12824 |
| YJM1388 | 13202 | CACCCACCCCCTCCCCCTATT-----------------------------------ACGTCTCCGAGGTCCCGGTTTCGTAAGAAACCGGGACTTATATA | 13266 |
| YJM789 | 12803 | CACCCACCCCCTCCCCCTATT-----------------------------------A-----------------------------------CTTATATA | 12832 |
| YJM1273 | 12749 | CACCCACCCCCTCCCCCTATT-----------------------------------A-----------------------------------CTTATATA | 12778 |
| NCYC3585 | 12832 | CACCCACCCCCTCCCCCTATT-----------------------------------A-----------------------------------CTTATATA | 12861 |
| YJM1401 | 13400 | CACCCACCCCCTCCCCCTATTACGTCTCCGAGGTCCCGGTTTCGTAAGAAACCGGGA-----------------------------------CTTATATA | 13464 |
| NCYC3594 | 12012 | CACCCACCCCCTCCCCCTATT-----------------------------------AC-----------------------------------TTATATA | 12041 |
| YJM1078 | 12672 | CACCCACCCCCTATTACGTCT------CCGAGGTCCCGGTTTCGTAAGAAACCGGGAC-----------------------------------TTATATA | 12730 |
| YJM1439 | 13536 | CACCCACCCCCTCCCCCTATT-----------------------------------ACGTCTCCGAGGTCCCGGTTTCGTAAGAAACCGGGACTTATATA | 13600 |
| consensus | 16901 | \*\*\*\*\*\*\*\*\*\*\*\*....\*...\*-----------------------------------\*------------------------------------\*\*\*\*\*\*\* | 17000 |
|
| S288C | 12825 | TTTAATACTAAAAATATAACTACATTAC---TTTTTTAATATATATAACAATATATATATATATATATATTAATTATATAAAATATAAT----------- | 12910 |
| YJM1388 | 13267 | TTTAATACTAAAAATATAACTACATTAC---TTTTTTAATATATATAACAATATATATATATATATATATTAATTATATAAAATATAAT----------- | 13352 |
| YJM789 | 12833 | TTTAATACTAAAAATATAACTACATTAC---TTTTTTAATATATATAACAATATATATATATATATATATTAATTATATAAAATATAAT----------- | 12918 |
| YJM1273 | 12779 | TTTAATACTAAAAATATAACTACATTAC---TTTTTTAATATATATAACAATATATATATATATATATATTAATTATATAAAATATAAT----------- | 12864 |
| NCYC3585 | 12862 | TTTAATACTAAAAATATAACTACATTAC---TTTTTTAATATATATAACAATATATATATATATATATATTAATTATATAAAATATAAT----------- | 12947 |
| YJM1401 | 13465 | TTTAATACTAAAAATATAACTACATTAC---TTTTTTAATATATATAACAATATATATATATATATATATTAATTATATAAAATATAAT----------- | 13550 |
| NCYC3594 | 12042 | TTTAATACTAAAAATATAACTACATTACTTTTTTTTTAATATATATAAC-ATATATATATATATATATATTAATTATATAAAATATAAT----------- | 12129 |
| YJM1078 | 12731 | TTTAATACTAAAAATATAACTACATTACTTTTTTTTTTA-ATATATATA-ACATATATATATATATATATTAATTATATAAAATATAAT----------- | 12817 |
| YJM1439 | 13601 | TTTAATACTAAAAATATAACTACATTAC---TTTTTTAATATATATATA-----ATATATATATATATATTAATTATATAAAATATAATACTCCTTCGGG | 13692 |
| consensus | 17001 | \*\*\*\*\*\*\*\*\*\*\*\*\*\*\*\*\*\*\*\*\*\*\*\*\*\*\*\*---\*\*\*\*\*\*.\*-\*\*\*\*\*\*\*..--.--\*\*\*\*\*\*\*\*\*\*\*\*\*\*\*\*\*\*\*\*\*\*\*\*\*\*\*\*\*\*\*\*\*\*\*----------- | 17100 |
|
| S288C | 12911 | -----------------ACTCTATATTAAATATTATTTTTATCAATATTTATTTATATATATAATAATAATAATAATAATCAATATTAATTATTTATATA | 12993 |
| YJM1388 | 13353 | -----------------ACTCTATATTAAATATTATTTTTATCAATATTTATTTATATATATAATAATAATAATAATAATCAATATTAATTATTTATATA | 13435 |
| YJM789 | 12919 | -----------------ACTCTATATTAAATATTATTTTTATCAATATTTATTTAT-------ATAATAATAATAATAATCAATATTAATTATTTACATA | 12994 |
| YJM1273 | 12865 | -----------------ACTCTATATTAAATATTATTTTTATCAATATTTATTTAT-------ATAATAATAATAATAATCAATATTAATTATTTACATA | 12940 |
| NCYC3585 | 12948 | -----------------ACTCTATATTAAATATTATTTTTATCAATATTTATTTATATATATAATAATAATAATAATAATCAATATTAATTATTTATATA | 13030 |
| YJM1401 | 13551 | -----------------ACTCTATATTAAATATTATTTTTATCAATATTTATTTATATATATAATAATAATAATAATAATCAATATTAATTATTTATATA | 13633 |
| NCYC3594 | 12130 | -----------------ACTCTATATTAAATATTATTTTTATCAATATTTATTTATATATATAATAATAATAATAATAATCAATATTAATTATTTATATA | 12212 |
| YJM1078 | 12818 | -----------------ACTCTATATTAAATATTA-TTTTATCAATATTTATTAATATATATAATAATAATAATAATAATCAATATTAATTATTTATATA | 12899 |
| YJM1439 | 13693 | GTCCCGCCGGGGCGGGGACTCTATATTAAATATTATTTTTATCAATATTTATTTAT-------ATAATAATAATAATAATCAATATTAATTATTTACATA | 13785 |
| consensus | 17101 | -----------------\*\*\*\*\*\*\*\*\*\*\*\*\*\*\*\*\*\*-\*\*\*\*\*\*\*\*\*\*\*\*\*\*\*\*\*.\*\*-------\*\*\*\*\*\*\*\*\*\*\*\*\*\*\*\*\*\*\*\*\*\*\*\*\*\*\*\*\*\*\*\*\*.\*\*\* | 17200 |
|
| S288C | 12994 | TATAAGATTAATATTATTTAATATATTATGAATAATTTAATTAATAAATCTTTAAATATTATCATAAAAA-TATAAATTAAATAATTTCTTATTTATAAT | 13092 |
| YJM1388 | 13436 | TATAAGATTAATATTATTAAATATATTATGAATAATTTAATTAATAAATCTTTAAATATTATCATAAAAA-TATAAATTAAATAATTTCTTATTTATAAT | 13534 |
| YJM789 | 12995 | TATAAGATTAATATTATTTAATATATTATGAATAATTTAATTAATAAATCTTTAAATATTATCATAAAAA-TATAAATTAAATAATTTCTTATTTATAAT | 13093 |
| YJM1273 | 12941 | TATAAGATTAATATTATTTAATATATTATGAATAATTTAATTAATAAATCTTTAAATATTATCATAAAAA-TATAAATTAAATAATTTCTTATTTATAAT | 13039 |
| NCYC3585 | 13031 | TATAAGATTAATATTATTTAATATATTATGAATAATTTAATTAATAAATCTTTAAATATTATCATAAAAA-TCTAAATTAAATAATTTCTTATTTATAAT | 13129 |
| YJM1401 | 13634 | TATAAGATTAATATTATTTAATATATTATGAATAATTTAATTAATAAATCTTTAAATATTATCATAAAAA-TATAAATTAAATAATTTCTTATTTATAAT | 13732 |
| NCYC3594 | 12213 | TATAAGATTAATATTATTTAATATATTATGAATAATTTAATTAATAAATCTTTAAATATTATCATAAAAA-TATAAATTAAATAATTTCTTATTTATAAT | 12311 |
| YJM1078 | 12900 | TATAAGATTAATATTATTTAATATATTATGAATAATTTAATTAATAAATCTTTAAATATTATTATAAAAAGTATAAATTAAATAACTTCTTATTTATAAT | 12999 |
| YJM1439 | 13786 | TATAAGATTAATATTATTTAATATATTATGAATAATTTAATTAATAAATCTTTAAATATTATCATAAAAA-TATAAATTAAATAACTTCTTATTTATAAT | 13884 |
| consensus | 17201 | \*\*\*\*\*\*\*\*\*\*\*\*\*\*\*\*\*\*.\*\*\*\*\*\*\*\*\*\*\*\*\*\*\*\*\*\*\*\*\*\*\*\*\*\*\*\*\*\*\*\*\*\*\*\*\*\*\*\*\*\*\*.\*\*\*\*\*\*\*-\*.\*\*\*\*\*\*\*\*\*\*\*\*.\*\*\*\*\*\*\*\*\*\*\*\*\*\* | 17300 |
|
| S288C | 13093 | AAAGAATAATA--------ATATATATAAATATAA-------TAAAGAATGTAAATAATATATATATAATATAATATAATATAAAAAATATATATATATA | 13177 |
| YJM1388 | 13535 | AAAGAATAATA--------ATATATATAAATATAA-------TAAAGAATGTAAATAATATATATATAATATAATATAATATAAAAAATATATATATATA | 13619 |
| YJM789 | 13094 | AAAGAATAATA--ATATATATATATATAAATATAA-------TAAAGAATGTAAATAATATAT-----ATATAATATAATATAAAAAATATATATATATA | 13179 |
| YJM1273 | 13040 | AAAGAATAATA--ATATATATATATATAAATATAA-------TAAAGAATGTAAATAATATAT-----ATATAATATAATATAAAAAATATATATATATA | 13125 |
| NCYC3585 | 13130 | AAAGAATAATAATATATATATATATATAAATATAA-------TAAAGAATGTAAATAATATAT-----ATATAATATAATATAAAAAATATATATATATA | 13217 |
| YJM1401 | 13733 | AAAGAATAATA--------ATATATATAAATATAA-------TAAAGAATGTAAATAATATATATATAATATAATATAATATAAAAAATATATATATATA | 13817 |
| NCYC3594 | 12312 | AAAGAATAATA--------ATATATATAAATATAA-------TAAAGAATGTAAATAATATATA-----TATAATATAATATAAAAAATATATATATATA | 12391 |
| YJM1078 | 13000 | AAAGAATAATA--------ATATATATATATATAAATATAATTAAAGAATGTAAATAATATATA-----TATAATATAATATAAAAAATATATATATATA | 13086 |
| YJM1439 | 13885 | AAAGAATAATA--ATATATATATATATAAATATAA-------TAAAGAATGTAAATAATATATA-----TATAATATAATATAAAAAATATATATATATA | 13970 |
| consensus | 17301 | \*\*\*\*\*\*\*\*\*\*\*--------\*\*\*\*\*\*\*\*\*.\*\*\*\*\*\*-------\*\*\*\*\*\*\*\*\*\*\*\*\*\*\*\*\*\*\*\*\*------\*\*\*\*\*\*\*\*\*\*\*\*\*\*\*\*\*\*\*\*\*\*\*\*\*\*\*\*\*\*\* | 17400 |
|
| S288C | 13178 | TAAATATAT----------ATATAATATATAGATAATAATATTTTTATA-TAATTTATTTTATTATTA-------------------------------- | 13234 |
| YJM1388 | 13620 | TAAATATATATATA-----ATATAATATATAGATAATAATATTTTTATATTAATTTATTTTATTATTA-------------------------------- | 13682 |
| YJM789 | 13180 | TAAATATATATATA-----ATATAATATATAGATAATAATATTTTTATATTAATTTATTTTATTATTAATTCCGGGGCCCGGCCACGGGAGCCGGAACCC | 13274 |
| YJM1273 | 13126 | TAAATATATATATA-----ATATAATATATAGATAATAATATTTTTATATTAATTTATTTTATTATTA-------------------------------- | 13188 |
| NCYC3585 | 13218 | TAAATATATATATA-----ATATAATATATAGATAATAATATTTTTATATTAATTTATTTTATTATTA-------------------------------- | 13280 |
| YJM1401 | 13818 | TAAATATAT----------ATATAATATATAGATAATAATATTTTTATA-TAATTTATTTTATTATTA-------------------------------- | 13874 |
| NCYC3594 | 12392 | TAAATATATATATAATATAATATAATATATAGATAATAATATTTTTATATTAATTTATTTTATTATTA-------------------------------- | 12459 |
| YJM1078 | 13087 | TA-----ATATAT------ATATAATATATAGATAATAATATTTTTATA-TAATTTATTTTATTATTA-------------------------------- | 13142 |
| YJM1439 | 13971 | TAAATATATATATA-----ATATAATATATAGATAATAATATTTTTATATTAATTTATTTTATTATTAATTCCGGGGCCCGGCCACGGGAGCCGGAACCC | 14065 |
| consensus | 17401 | \*\*-----\*\*----------\*\*\*\*\*\*\*\*\*\*\*\*\*\*\*\*\*\*\*\*\*\*\*\*\*\*\*\*\*\*-\*\*\*\*\*\*\*\*\*\*\*\*\*\*\*\*\*\*-------------------------------- | 17500 |
|
| S288C | 13235 | -------AGTAATAAATAATAAAAAAATCAATATATTAAATAATATATTTATATTAGTTCGGTTTAGTTGGTA-TTTTGTAATGAGTAAAAA-GTAATAT | 13325 |
| YJM1388 | 13683 | -------AGTAATGAATAAT-ATAAAATCAATATATTAAATAATATATTTATATTAGTTCGGTTTAGTTGGTA-TTTTGTAATGAGTAAAAA-GTAATAT | 13772 |
| YJM789 | 13275 | CGAAAGGAGTAATGAATAATA-TAAAATCAATATATTAAATAATATATTTATATTAGTTCGGTTTAGTTGGTA-TTTTGTAATGAGTAAAAA-GTAATAT | 13371 |
| YJM1273 | 13189 | -------AGTAATGAATAATA-TAAAATCAATATATTAAATAATATATTTATATTAGTTCGGTTTAGTTGGTA-TTTTGTAATGAGTAAAAA-GTAATAT | 13278 |
| NCYC3585 | 13281 | -------AGTAATGAATAATA-TAAAATCAATATATTAAATAATATATTTATATTAGTTCGGTTTAGTTGGTA-TTTTGTAATGAGTAAAAA-GTAATAT | 13370 |
| YJM1401 | 13875 | -------AGTAATAAATAATA-AAAAATCAATATATTAAATAATATATTTATATTAGTTCGGTTTAGTTGGTA-TTTTGTAATGAGTAAAAA-GTAATAT | 13964 |
| NCYC3594 | 12460 | -------AGTAATGAATAATA-TAAAATCAATATATTAAATAATATATTTATATTAGTTCGGTTTAGTTGGTA-TTTTGTAATGAGTAAAAA-GTAATAT | 12549 |
| YJM1078 | 13143 | -------AGTAATGAATAATA-TAAAATCAATATATTAAATAATATATTTATATTAGTTCGATCTAGTTGGGATTTTTGTAATGAGTAAAAAGGTAATAT | 13234 |
| YJM1439 | 14066 | CGAAAGGAGTAATGAATAATA-TAAAATCAATATATTAAATAATATATTTATATTAGTTCGGTCTAATTGGTA-TTTTGTAATGAGTAAAAA-GTAATAT | 14162 |
| consensus | 17501 | -------\*\*\*\*\*\*.\*\*\*\*\*\*--.\*\*\*\*\*\*\*\*\*\*\*\*\*\*\*\*\*\*\*\*\*\*\*\*\*\*\*\*\*\*\*\*\*\*\*\*\*\*.\*.\*\*.\*\*\*\*.\*-\*\*\*\*\*\*\*\*\*\*\*\*\*\*\*\*\*\*-\*\*\*\*\*\*\* | 17600 |
|
| S288C | 13326 | ATAATATTAAATAATAAGTATTGATATAAGTAATAGATATAATAATAATATTA-TTAATATTTTATATAAATAATATTAATAATATAGATTATGAAAGAG | 13424 |
| YJM1388 | 13773 | ATAATATTAAATAATAAGTATTGATATAAGTAATAGATATAATAATAATATTA-TTAATATTTTATATAAATAATATTAATAATATAGATTATGAAAGAG | 13871 |
| YJM789 | 13372 | ATAATATTAAATAATAAGTATTGATATAAGTAATAGATATAATAATAATATTA-TTAATATTTTATATAAATAATATTAATAATATAGATTATGAAAGAG | 13470 |
| YJM1273 | 13279 | ATAATATTAAATAATAAGTATTGATATAAGTAATAGATATAATAATAATATTA-TTAATATTTTATATAAATAATATTAATAATATAGATTATGAAAGAG | 13377 |
| NCYC3585 | 13371 | ATAATATTAAATAATAAGTATTGATATAAGTAATAGATATAATAATAATATTA-TTAATATTTTATATAAATAATATTAATAATATAGATTATGAAAGAG | 13469 |
| YJM1401 | 13965 | ATAATATTAAATAATAAGTATTGATATAAGTAATAGATATAATAATAATATTA-TTAATATTTTATATAAATAATATTAATAATATAGATTATGAAAGAG | 14063 |
| NCYC3594 | 12550 | ATAATATTAAATAATAAGTATTGATATAAGTAATAGATATAATAATAATATTATTTAATATTATATATAAATAATATTAATAATATAGATTATGAAAGAG | 12649 |
| YJM1078 | 13235 | ATAATATTAAATAATAAGTATTGATATAAGTAATAGATATAATAATAATATTATTTAATATTATATATAAATAATATTAATAATATAGATTATGAAAGAG | 13334 |
| YJM1439 | 14163 | ATAATATTAAATAATAAGTATTGATATAAGTAATAGATATAATAATAATATTA-TTAATATTTTATATAAATAATATTAATAATATAGATTATGAAAGAG | 14261 |
| consensus | 17601 | \*\*\*\*\*\*\*\*\*\*\*\*\*\*\*\*\*\*\*\*\*\*\*\*\*\*\*\*\*\*\*\*\*\*\*\*\*\*\*\*\*\*\*\*\*\*\*\*\*\*\*\*\*-\*\*\*\*\*\*\*\*.\*\*\*\*\*\*\*\*\*\*\*\*\*\*\*\*\*\*\*\*\*\*\*\*\*\*\*\*\*\*\*\*\*\*\*\*\* | 17700 |
|
| S288C | 13425 | AGTATTAATATCATTAAATATATATATATGTTATATAATTTAAATGATTTTAATATATATATATATATTATATTATAGATTATGATACATTTATATAAAT | 13524 |
| YJM1388 | 13872 | AGTATTAATATCATTAAATATATATATATGTTATATAATTTAAATGATTTTAATATATATATATATATTATATTATAGATTATGATACATTTATATAAAT | 13971 |
| YJM789 | 13471 | AGTATTAATATCATTAAATATATATATATGTTATATAATTTAAATGATTTTAATATATATATATATATTATATTATAGATTATGATACATTTATATAAAT | 13570 |
| YJM1273 | 13378 | AGTATTAATATCATTAAATATATATATATGTTATATAATTTAAATGATTTTAATATATATATATATATTATATTATAGATTATGATACATTTATATAAAT | 13477 |
| NCYC3585 | 13470 | AGTATTAATATCATTAAATATATATATATGTTATATAATTTAAATGATTTTAATATATATATATATATTATATTATAGATTATGATACATTTATATAAAT | 13569 |
| YJM1401 | 14064 | AGTATTAATATCATTAAATATATATATATGTTATATAATTTAAATGATTTTAATATATATATATATATTATATTATAGATTATGATACATTTATATAAAT | 14163 |
| NCYC3594 | 12650 | AGTATTAATATCATTAAATATATATATATGTTATATAATTTAAATGATTTTAATATATATATATATATTATATTATAGATTATGATACATTTATATAAAT | 12749 |
| YJM1078 | 13335 | AGTATTAATATCATTAAATATATATATATGTTATATAATTTAAATGATTTTAATATATATATATATATTATATTATAGATTATGATACATTTATATAAAT | 13434 |
| YJM1439 | 14262 | AGTATTAATATCATTAAATATATATATATGTTATATAATTTAAATGATTTTAATATATATATATATATTATATTATAGATTATGATACATTTATATAAAT | 14361 |
| consensus | 17701 | \*\*\*\*\*\*\*\*\*\*\*\*\*\*\*\*\*\*\*\*\*\*\*\*\*\*\*\*\*\*\*\*\*\*\*\*\*\*\*\*\*\*\*\*\*\*\*\*\*\*\*\*\*\*\*\*\*\*\*\*\*\*\*\*\*\*\*\*\*\*\*\*\*\*\*\*\*\*\*\*\*\*\*\*\*\*\*\*\*\*\*\*\*\*\*\*\*\*\*\* | 17800 |
|
| S288C | 13525 | AATATATATATAAAAATTAATTATACTATTACTTTATAATATAATAATATTTATTTATAAAGATATAAAAGAATTGTTTAAAGTTATAACTAAAATATTA | 13624 |
| YJM1388 | 13972 | AATATATATATAAAAATTAATTATACTATTACTTTATAATATAATAATATTTATTTATAAAGATATAAAAGAATTGTTTAAAGTTATAACTAAAATATTA | 14071 |
| YJM789 | 13571 | AATATATATATAAAAATTAATTATACTATTACTTTATAATATAATAATATTTATTTATAAAGATATAAAAGAATTGTTTAAAGTTATAACTAAAATATTA | 13670 |
| YJM1273 | 13478 | AATATATATATAAAAATTAATTATACTATTACTTTATAATATAATAATATTTATTTATAAAGATATAAAAGAATTGTTTAAAGTTATAACTAAAATATTA | 13577 |
| NCYC3585 | 13570 | AATATATATATAAAAATTAATTATACTATTACTTTATAATATAATAATATTTATTTATAAAGATATAAAAGAATTGTTTAAAGTTATAACTAAAATATTA | 13669 |
| YJM1401 | 14164 | AATATATATATAAAAATTAATTATACTATTACTTTATAATATAATAATATTTATTTATAAAGATATAAAAGAATTGTTTAAAGTTATAACTAAAATATTA | 14263 |
| NCYC3594 | 12750 | AATATATATATAAAAATTAATTATACTATTACTTTATAATATAATAATATTTATTTATAAAGATATAAAAGAATTGTTTAAAGTTATAACTAAAATATTA | 12849 |
| YJM1078 | 13435 | AATATATATATAAAAATTAATTATACTATTACTTTATAATATAATAATATTTATTTATAAAGATATAAAAGAATTGTTTAAAGTTATAACTAAAATATTA | 13534 |
| YJM1439 | 14362 | AATATATATATAAAAATTAATTATACTATTACTTTATAATATAATAATATTTATTTATAAAGATATAAAAGAATTGTTTAAAGTTATAACTAAAATATTA | 14461 |
| consensus | 17801 | \*\*\*\*\*\*\*\*\*\*\*\*\*\*\*\*\*\*\*\*\*\*\*\*\*\*\*\*\*\*\*\*\*\*\*\*\*\*\*\*\*\*\*\*\*\*\*\*\*\*\*\*\*\*\*\*\*\*\*\*\*\*\*\*\*\*\*\*\*\*\*\*\*\*\*\*\*\*\*\*\*\*\*\*\*\*\*\*\*\*\*\*\*\*\*\*\*\*\*\* | 17900 |
|
| S288C | 13625 | TAT------------------------------------------AGTATTCATTAATAATTAATATTATAAATTCAACTATTGTTATATTTATAAATAG | 13682 |
| YJM1388 | 14072 | TAT------------------------------------------AGTATTCATTAATAATTAATATTATAAATTCAACTATTGTTATATTTATAAATAG | 14129 |
| YJM789 | 13671 | TATAGTCCGGCCCGCCCCCGCGGGGCGGACCCC-------GAAGGAGTATTCATTAATAATTAATATTATAAATTCAACTATTGTTATATTTATAAATAG | 13763 |
| YJM1273 | 13578 | TAT------------------------------------------AGTATTCATTAATAATTAATATTATAAATTCAACTATTGTTATATTTATAAATAG | 13635 |
| NCYC3585 | 13670 | TAT------------------------------------------AGTATTCATTAATAATTAATATTATAAATTCAACTATTGTTATATTTATAAATAG | 13727 |
| YJM1401 | 14264 | TAT------------------------------------------AGTATTCATTAATAATTAATATTATAAATTCAACTATTGTTATATTTATAAATAG | 14321 |
| NCYC3594 | 12850 | TAT------------------------------------------AGTATTCATTAATAATTAATATTATAAATTCAACTATTGTTATATTTATAAATAG | 12907 |
| YJM1078 | 13535 | TAT------------------------------------------AGTATTCATTAATAATTAATATTATAAATTCAACTATTGTTATATTTATAAATAG | 13592 |
| YJM1439 | 14462 | TATAGTCCGGCCCGCCCCCGCGGGGCGGACCCCTTTTTAAGAAGGAGTATTCATTAATAATAAATATTATAAATTCAACTATTGTTATATTTATAAATAG | 14561 |
| consensus | 17901 | \*\*\*------------------------------------------\*\*\*\*\*\*\*\*\*\*\*\*\*\*\*\*.\*\*\*\*\*\*\*\*\*\*\*\*\*\*\*\*\*\*\*\*\*\*\*\*\*\*\*\*\*\*\*\*\*\*\*\*\*\* | 18000 |
|
| S288C | 13683 | AATAATATATTATTATCCTTTAAGATATAACAATAATTATTTAAATTAAATTAAATTAAATTTAAT-TAATTTTTTTTTTTAATGAATATAATAATAATA | 13781 |
| YJM1388 | 14130 | AATAATATATTATTATCCTTTAAAATATAACAATAATTATTTAAATTAAATTAAATTTAATTAA------------TTTTTAATGAATATAATAATAATA | 14217 |
| YJM789 | 13764 | AATAATATATTATTATCCTTTAAGATATAACAATAATTATTTAAATTAAATTAAATTTAATTAA------------TTTTTAATGAATATAATAATAATA | 13851 |
| YJM1273 | 13636 | AATAATATATTATTATCCTTTAAAATATAACAATAATTATTTAAATTAAATTAAATTTAATTAA------------TTTTTAATGAATATAATAATAATA | 13723 |
| NCYC3585 | 13728 | AATAATATATTATTATCCTTTAAAATATAACAATAATTATTTAAATTAAATTAAATTTAATTAATT-TT-------TTTTTAATGAATATAATAATAATA | 13819 |
| YJM1401 | 14322 | AATAATATATTATTATCCTTTAAGATATAACAATAATTATTTAAATTAAATTAAATTTAATTAATTTTT-------TTTTTAATGAATATAATAATAATA | 14414 |
| NCYC3594 | 12908 | AATAATATATTATTATCCTTAAGGATATAACAATAAT-----------AAAAAAATTTAATAA--------------TTTTAATGAATATAATAATAATA | 12982 |
| YJM1078 | 13593 | AATAATATATTATTATCCTTAAGGATATAACAATAAT-----------AAAAAAATTTAATAA--------------TTTTAATGAATATAATAATAATA | 13667 |
| YJM1439 | 14562 | AATAATATATTATTATCATTTAAGATATAACAATAATTATTTAAATTAAATTAAATTTAATTAATT--------TTTTTTTAATGAATATAATAATAATA | 14653 |
| consensus | 18001 | \*\*\*\*\*\*\*\*\*\*\*\*\*\*\*\*\*.\*\*.\*..\*\*\*\*\*\*\*\*\*\*\*\*\*-----------\*\*..\*\*\*\*\*.\*\*\*..-.---.--------\*\*\*\*\*\*\*\*\*\*\*\*\*\*\*\*\*\*\*\*\*\*\* | 18100 |
|
| S288C | 13782 | ATATTATTAAAATTAATATATAAAAAAAAAGTAAAAATGGTACAAAGATGATTATATTCAACAAATGCAAAAGATATTGCAGTATTATATTTTATGTTAG | 13881 |
| YJM1388 | 14218 | ATATTATTAAAATTAATATATAAAAAAAAAGTAAAAATGGTACAAAGATGATTATATTCAACAAATGCAAAAGATATTGCAGTATTATATTTTATGTTAG | 14317 |
| YJM789 | 13852 | ATATTATTAAAATTAATATATAAAAAAAAAGTAAAAATGGTACAAAGATGATTATATTCAACAAATGCAAAAGATATTGCAGTATTATATTTTATGTTAG | 13951 |
| YJM1273 | 13724 | ATATTATTAAAATTAATATATAAAAAAAAAGTAAAAATGGTACAAAGATGATTATATTCAACAAATGCAAAAGATATTGCAGTATTATATTTTATGTTAG | 13823 |
| NCYC3585 | 13820 | ATATTATTAAAATTAATATATAAAAAAAAAGTAAAAATGGTACAAAGATGATTATATTCAACAAATGCAAAAGATATTGCAGTATTATATTTTATGTTAG | 13919 |
| YJM1401 | 14415 | ATATTATTAAAATTAATATATAAAAAAAAAGTAAAAATGGTACAAAGATGATTATATTCAACAAATGCAAAAGATATTGCAGTATTATATTTTATGTTAG | 14514 |
| NCYC3594 | 12983 | ATATTATTAAAATTAATATAT--AAAAAAAGTAAAAATGGTACAAAGATGATTATATTCAACAAATGCAAAAGATATTGCAGTATTATATTTTATGTTAG | 13080 |
| YJM1078 | 13668 | ATATTATTAAAATTAATATAT--AAAAAAAGTAAAAATGGTACAAAGATGATTATATTCAACAAATGCAAAAGATATTGCAGTATTATATTTTATGTTAG | 13765 |
| YJM1439 | 14654 | ATATTATTAAAATTAATATAT-AAAAAAAAGTAAAAATGGTACAAAGATGATTATATTCAACAAATGCAAAAGATATTGCAGTATTATATTTTATGTTAG | 14752 |
| consensus | 18101 | \*\*\*\*\*\*\*\*\*\*\*\*\*\*\*\*\*\*\*\*\*--\*\*\*\*\*\*\*\*\*\*\*\*\*\*\*\*\*\*\*\*\*\*\*\*\*\*\*\*\*\*\*\*\*\*\*\*\*\*\*\*\*\*\*\*\*\*\*\*\*\*\*\*\*\*\*\*\*\*\*\*\*\*\*\*\*\*\*\*\*\*\*\*\*\*\*\*\* | 18200 |
|
| S288C | 13882 | CTATTTTTAGTGGTATGGCAGGAACAGCAATGTCTTTAATCATTAGATTAGAATTAGCTGCACCTGGTTCACAATATTTACATGGTAATTCACAATTATT | 13981 |
| YJM1388 | 14318 | CTATTTTTAGTGGTATGGCAGGAACAGCAATGTCTTTAATCATTAGATTAGAATTAGCTGCACCTGGTTCACAATATTTACATGGAAATTCACAGTTATT | 14417 |
| YJM789 | 13952 | CTATTTTTAGTGGTATGGCAGGAACAGCAATGTCTTTAATCATTAGATTAGAATTAGCTGCACCTGGTTCACAATATTTACATGGAAATTCACAATTATT | 14051 |
| YJM1273 | 13824 | CTATTTTTAGTGGTATGGCAGGAACAGCAATGTCTTTAATCATTAGATTAGAATTAGCTGCACCTGGTTCACAATATTTACATGGAAATTCACAATTATT | 13923 |
| NCYC3585 | 13920 | CTATTTTTAGTGGTATGGCAGGAACAGCAATGTCTTTAATCATTAGATTAGAATTAGCTGCACCTGGTTCACAATATTTACATGGAAATTCACAATTATT | 14019 |
| YJM1401 | 14515 | CTATTTTTAGTGGTATGGCAGGAACAGCAATGTCTTTAATCATTAGATTAGAATTAGCTGCACCTGGTTCACAATATTTACATGGAAATTCACAGTTATT | 14614 |
| NCYC3594 | 13081 | CTATTTTTAGTGGTATGGCAGGAACAGCAATGTCTTTAATCATTAGATTAGAATTAGCTGCACCTGGTTCACAATATTTACATGGAAATTCACAATTATA | 13180 |
| YJM1078 | 13766 | CTATTTTTAGTGGTATGGCAGGAACAGCAATGTCTTTAATCATTAGATTAGAATTAGCTGCACCTGGTTCACAATATTTACATGGAAATTCACAATTATT | 13865 |
| YJM1439 | 14753 | CTATTTTTAGTGGTATGGCAGGAACAGCAATGTCTTTAATCATTAGATTAGAATTAGCTGCACCTGGTTCACAATATTTACATGGTAATTCACAGTTATT | 14852 |
| consensus | 18201 | \*\*\*\*\*\*\*\*\*\*\*\*\*\*\*\*\*\*\*\*\*\*\*\*\*\*\*\*\*\*\*\*\*\*\*\*\*\*\*\*\*\*\*\*\*\*\*\*\*\*\*\*\*\*\*\*\*\*\*\*\*\*\*\*\*\*\*\*\*\*\*\*\*\*\*\*\*\*\*\*\*\*\*\*\*.\*\*\*\*\*\*\*\*.\*\*\*\*. | 18300 |
|
| S288C | 13982 | TAATGGTGCGCCTCTCAGTGCGTATATTTCGTTGATGCGTCTAGCATTAGTATTATGAATCATCAATAGATACTTAAAACATATGACTAACTCAGTAGGG | 14081 |
| YJM1388 | 14418 | TAATGGTGCGCCTCTCAGTGCGTATATTTCGTTGATGCGTCTAGCATTAGTATTATGAATCATCAATAGATACTTAAAACATATGACTAACTCAGTAGGG | 14517 |
| YJM789 | 14052 | TAATGGTGCGCCTCTCAGTGCGTATATTTCGTTGATGCGTCTAGCATTAGTATTATGAATCATCAATAGATACTTAAAACATATGACTAACTCAGTAGGG | 14151 |
| YJM1273 | 13924 | TAATG----------------------------------------------------------------------------------------------- | 13928 |
| NCYC3585 | 14020 | TAATG----------------------------------------------------------------------------------------------- | 14024 |
| YJM1401 | 14615 | TAATG----------------------------------------------------------------------------------------------- | 14619 |
| NCYC3594 | 13181 | TAATG----------------------------------------------------------------------------------------------- | 13185 |
| YJM1078 | 13866 | TAATGGTGCGCCTCTCAGTGCGTATATTTCGTTGATGCGTCTAGCATTAGTATTATGAATCATCAATAGATACTTAAAACATATGACTAACTCAGTAGGG | 13965 |
| YJM1439 | 14853 | TAATGGTGCGCCTCTCAGTGCGTATATTTCGTTGATGCGTCTAGCATTAGTATTATGAATCATCAATAGATACTTAAAACATATGACTAACTCAGTAGGG | 14952 |
| consensus | 18301 | \*\*\*\*\*----------------------------------------------------------------------------------------------- | 18400 |
|
| S288C | 14082 | GCTAACTTTACGGGGACAATAGCATGTCATAAAACACCTATGATTAGTGTAGGTGGAGTTAAGTGTTACATGGTTAGGTTAACGAACTTCTTACAAGTCT | 14181 |
| YJM1388 | 14518 | GCTAACTTTACGGGGACAATAGCATGTCATAAAACACCTATGATTAGTGTAGGTGGAGTTAAGTGTTACATGGTTAGGTTAACGAACTTCTTACAAGTCT | 14617 |
| YJM789 | 14152 | GCTAACTTTACGGGGACAATAGCATGTCATAAAACACCTATGATTAGTGTAGGTGGAGTTAAGTGTTACATGGTTAGGTTAACGAACTTCTTACAAGTCT | 14251 |
| YJM1273 | 13929 | ---------------------------------------------------------------------------------------------------- | 13928 |
| NCYC3585 | 14025 | ---------------------------------------------------------------------------------------------------- | 14024 |
| YJM1401 | 14620 | ---------------------------------------------------------------------------------------------------- | 14619 |
| NCYC3594 | 13186 | ---------------------------------------------------------------------------------------------------- | 13185 |
| YJM1078 | 13966 | GCTAACTTTACGGGGACAATAGCATGTCATAAAACACCTCTGATTAGTGTAGGTGGAGTTAAGTGTTACATGGTTAGGTTAACGAACTTCTTACAAGTCT | 14065 |
| YJM1439 | 14953 | GCTAACTTTACGGGGACAATAGCATGTCATAAAACACCTATGATTAGTGTAGGTGGAGTTAAGTGTTACATGGTTAGGTTAACGAACTTCTTACAAGTCT | 15052 |
| consensus | 18401 | ---------------------------------------.------------------------------------------------------------ | 18500 |
|
| S288C | 14182 | TTATCAGGATTACAATTTCCTCTTATCATTTGGATATAGTAAAACAAGTTTGATTATTTTACGTTGAGGTAATCAGATTATGATTCATTGTTTTAGATAG | 14281 |
| YJM1388 | 14618 | TTATCAGGATTACAATTTCCTCTTATCATTTGGATATAGTAAAACAAGTTTGATTATTTTACGTTGAGGTAATCAGATTATGATTCATTGTTTTAGATAG | 14717 |
| YJM789 | 14252 | TTATCAGGATTACAATTTCCTCTTATCATTTGGATATAGTAAAACAAGTTTGATTATTTTACGTTGAGGTAATCAGATTATGATTCATTGTTTTAGATAG | 14351 |
| YJM1273 | 13929 | ---------------------------------------------------------------------------------------------------- | 13928 |
| NCYC3585 | 14025 | ---------------------------------------------------------------------------------------------------- | 14024 |
| YJM1401 | 14620 | ---------------------------------------------------------------------------------------------------- | 14619 |
| NCYC3594 | 13186 | ---------------------------------------------------------------------------------------------------- | 13185 |
| YJM1078 | 14066 | TTATCAGGATTACAATTTCCTCTTATCATTTGGATATAGTAAAACAAGTTTGATTATTTTACGTTGAGGTAATCAGATTATGATTCATTGTTTTAGATAG | 14165 |
| YJM1439 | 15053 | TTATCAGGATTACAATTTCCTCTTATCATTTGGATATAGTAAAACAAGTTTGATTATTTTACGTTGAGGTAATCAGATTATGATTCATTGTTTTAGATAG | 15152 |
| consensus | 18501 | ---------------------------------------------------------------------------------------------------- | 18600 |
|
| S288C | 14282 | CACAGGCAGTGTGAAAAAGATGAAGGACCTAAATAACACAAAAGGAAATACGAAAAGTGAGGGATCAACTGAAAGAGGAAACTCTGGAGTTGACAGAGGT | 14381 |
| YJM1388 | 14718 | CACAGGCAGTGTGAAAAAGATGAAGGACCTAAATAACACAAAAGGAAATACGAAAAGTGAGGGATCAACTGAAAGAGGAAACTCTGGAGTTGACAGAGGT | 14817 |
| YJM789 | 14352 | CACAGGCAGTGTGAAAAAGATGAAGGACCTAAATAACACAAAAGGAAATACGAAAAGTGAGGGATCAACTGAAAGAGGAAACTCTGGAGTTGACAGAGGT | 14451 |
| YJM1273 | 13929 | ---------------------------------------------------------------------------------------------------- | 13928 |
| NCYC3585 | 14025 | ---------------------------------------------------------------------------------------------------- | 14024 |
| YJM1401 | 14620 | ---------------------------------------------------------------------------------------------------- | 14619 |
| NCYC3594 | 13186 | ---------------------------------------------------------------------------------------------------- | 13185 |
| YJM1078 | 14166 | CACAGGCAGTGTGAAAAAGATGAAGGACCTAAATAACACAAAAGGAAATACGAAAAGTGAGGGATCAACTGAAAGAGGAAACTCTGGAGTTGACAGAGGT | 14265 |
| YJM1439 | 15153 | CACAGGCAGTGTGAAAAAGATGAAGGACCTAAATAACACAAAAGGAAATACGAAAAGTGAGGGATCAACTGAAAGAGGAAACTCTTGAGTTGACAGAGGT | 15252 |
| consensus | 18601 | -------------------------------------------------------------------------------------.-------------- | 18700 |
|
| S288C | 14382 | ATAGTAGTACCGAATACTCAAATAAAAATGAGATTTTTAAATCAAGTTAGATACTATTCAGTAAATAATAATTTAAAAATAGGGAAGGATACCAATATTG | 14481 |
| YJM1388 | 14818 | ATAGTAGTACCGAATACTCAAATAAAAATGAGATTTTTAAATCAAGTTAGATACTATTCAGTAAATAATAATTTAAAAATAGGGAAGGATACCAATATTG | 14917 |
| YJM789 | 14452 | ATAGTAGTACCGAATACTCAAATAAAAATGAGATTTTTAAATCAAGTTAGATACTATTCAGTAAATAATAATTTAAAAATAGGGAAGGATACCAATATTG | 14551 |
| YJM1273 | 13929 | ---------------------------------------------------------------------------------------------------- | 13928 |
| NCYC3585 | 14025 | ---------------------------------------------------------------------------------------------------- | 14024 |
| YJM1401 | 14620 | ---------------------------------------------------------------------------------------------------- | 14619 |
| NCYC3594 | 13186 | ---------------------------------------------------------------------------------------------------- | 13185 |
| YJM1078 | 14266 | ATAGTAGTACCGAATACTCAAATAAAAATGAGATTTTTAAATCAAGTTAGATACTATTCAGTAAATAATAATTTAAAAATAGGGAAGGATACCAATATTG | 14365 |
| YJM1439 | 15253 | ATAGTAGTACCGAATACTCAAATAAAAATGAGATTTTTAAATCAAGTTAGATACTATTCAGTAAATAATAATTTAAAAATAGGGAAGGATACCAATATTG | 15352 |
| consensus | 18701 | ---------------------------------------------------------------------------------------------------- | 18800 |
|
| S288C | 14482 | AGTTATCAAAAGATACAAGTACTTCGGACTTGTTAGAATTTGAGAAATTAGTAATAGATAATATAAATGAGGAAAATATAAATAATAATTTATTAAGTAT | 14581 |
| YJM1388 | 14918 | AGTTATCAAAAGATACAAGTACTTCGGACTTGTTAGAATTTGAGAAATTAGTAATAGATAATATAAATGAGGAAAATATAAATAATAATTTATTAAGTAT | 15017 |
| YJM789 | 14552 | AGTTATCAAAAGATACAAGTACTTCGGACTTGTTAGAATTTGAGAAATTAGTAATAGATAATATAAATAAGGAAAATATAAATAATAATTTATTAAGTAT | 14651 |
| YJM1273 | 13929 | ---------------------------------------------------------------------------------------------------- | 13928 |
| NCYC3585 | 14025 | ---------------------------------------------------------------------------------------------------- | 14024 |
| YJM1401 | 14620 | ---------------------------------------------------------------------------------------------------- | 14619 |
| NCYC3594 | 13186 | ---------------------------------------------------------------------------------------------------- | 13185 |
| YJM1078 | 14366 | AGTTATCAAAAGATACAAGTACTTCGGACTTGTTAGAATTTGAGAAATTAGTAATAGATAATATAAATAAGGAAAATATAAATAATAATTTATTAAGTAT | 14465 |
| YJM1439 | 15353 | AGTTATCAAAAGATACAAGTACTTCGGACTTGTTAGAATTAGAGAAATTAGTAATAGATAATATAAATGAGGAAAATATAAATAATAATTTATTAAGTAT | 15452 |
| consensus | 18801 | ----------------------------------------.---------------------------.------------------------------- | 18900 |
|
| S288C | 14582 | TATAAAAAACGTAGATATATTAATATTAGCATATAATAGAATTAAGAGTAAACCTGGTAATATAACTCCAGGTACAACATTAGAAACATTAGATGGTATA | 14681 |
| YJM1388 | 15018 | TATAAAAAACGTAGATATATTAATATTAGCATATAATAGAATTAAGAGTAAACCTGGTAATATAACTCCAGGTACAACATTAGAAACATTAGATGGTATA | 15117 |
| YJM789 | 14652 | TATAAAAAACGTAGATATATTAATATTAGCATATAATAGAATTAAGAGTAAACCTGGTAATATAACTCCAGGTACAACATTAGAAACATTAGATGGTATA | 14751 |
| YJM1273 | 13929 | ---------------------------------------------------------------------------------------------------- | 13928 |
| NCYC3585 | 14025 | ---------------------------------------------------------------------------------------------------- | 14024 |
| YJM1401 | 14620 | ---------------------------------------------------------------------------------------------------- | 14619 |
| NCYC3594 | 13186 | ---------------------------------------------------------------------------------------------------- | 13185 |
| YJM1078 | 14466 | TATAAAAAACGTAGATATATTAATATTAGCATATAATAGAATTAAGAGTAAACCTGGTAATATAACTCCAGGTACAACATTAGAAACATTAGATGGTATA | 14565 |
| YJM1439 | 15453 | TATAAAAAACGTAGATATATTAATATTAGCATATAATAGAATTAAGAGTAAACCTGGTAATATAACTCCAGGTACAACATTAGAAACATTAGATGGTATA | 15552 |
| consensus | 18901 | ---------------------------------------------------------------------------------------------------- | 19000 |
|
| S288C | 14682 | AATATAATATATTTAAATAAATTATCAAATGAATTAGGAACAGGTAAATTCAAATTTAAACCCATGAGAATAGTTAATATTCCTAAACCTAAAGGTGGTA | 14781 |
| YJM1388 | 15118 | AATATAATATATTTAAATAAATTATCAAATGAATTAGGAACAGGTAAATTCAAATTTAAACCCATGAGAATAGTTAATATTCCTAAACCTAAAGGTGGTA | 15217 |
| YJM789 | 14752 | AATATAATATATTTAAATAAATTATCAAATGAATTAGGAACAGGTAAATTCAAATTTAAACCCATGAGAATAGTTAATATTCCTAAACCTAAAGGTGGTA | 14851 |
| YJM1273 | 13929 | ---------------------------------------------------------------------------------------------------- | 13928 |
| NCYC3585 | 14025 | ---------------------------------------------------------------------------------------------------- | 14024 |
| YJM1401 | 14620 | ---------------------------------------------------------------------------------------------------- | 14619 |
| NCYC3594 | 13186 | ---------------------------------------------------------------------------------------------------- | 13185 |
| YJM1078 | 14566 | AATATAATATATTTAAATAAATTATCAAATGAATTAGGAACAGGTAAATTCAAATTTAAACCCATGAGAATAGTTAATATTCCTAAACCTAAAGGTGGTA | 14665 |
| YJM1439 | 15553 | AATATAATATATTTAAATAAATTATCAAATGAATTAGGAACAGGTAAATTCAAATTTAAACCCATGAGAATAGTTAATATTCCTAAACCTAAAGGTGGTA | 15652 |
| consensus | 19001 | ---------------------------------------------------------------------------------------------------- | 19100 |
|
| S288C | 14782 | TAAGACCTTTAAGTGTAGGTAATCCAAGAGATAAAATTGTACAAGAAGTTATAAGAATAATTTTAGATACAATTTTTGATAAAAAGATATCAACACATTC | 14881 |
| YJM1388 | 15218 | TAAGACCTTTAAGTGTAGGTAATCCAAGAGATAAAATTGTACAAGAAGTTATAAGAATAATTTTAGATACAATTTTTGATAAAAAGATATCAACACATTC | 15317 |
| YJM789 | 14852 | TAAGACCTTTAAGTGTAGGTAATCCAAGAGATAAAATTGTACAAGAAGTTATAAGAATAATTTTAGATACAATTTTTGATAAAAAGATATCAACACATTC | 14951 |
| YJM1273 | 13929 | ---------------------------------------------------------------------------------------------------- | 13928 |
| NCYC3585 | 14025 | ---------------------------------------------------------------------------------------------------- | 14024 |
| YJM1401 | 14620 | ---------------------------------------------------------------------------------------------------- | 14619 |
| NCYC3594 | 13186 | ---------------------------------------------------------------------------------------------------- | 13185 |
| YJM1078 | 14666 | TAAGACCTTTAAGTGTAGGTAATCCAAGAGATAAAATTGTACAAGAAGTTATAAGAATAATTTTAGATACAATTTTTGATAAAAAGATATCAGCACATTC | 14765 |
| YJM1439 | 15653 | TAAGACCTTTAAGTGTAGGTAATCCAAGAGATAAAATTGTACAAGAAGTTATAAGAATAATTTTAGATACAATTTTTGATAAAAAGATATCAACACATTC | 15752 |
| consensus | 19101 | --------------------------------------------------------------------------------------------.------- | 19200 |
|
| S288C | 14882 | ACATGGTTTTAGAAAGAATATAAGTTGTCAAACAGCAATTTGAGAAGTTAGAAATATATTTGGTGGAAGTAATTGATTTATTGAAGTAGACTTAAAAAAA | 14981 |
| YJM1388 | 15318 | ACATGGTTTTAGAAAGAATATAAGTTGTCAAACAGCAATTTGAGAAGTTAGAAATATATTTGGTGGAAGTAATTGATTTATTGAAGTAGACTTAAAAAAA | 15417 |
| YJM789 | 14952 | ACATGGTTTTAGAAAGAATATAAGTTGTCAAACAGCAATTTGAGAAGTTAGAAATATATTTGGTGGAAGTAATTGATTTATTGAAGTAGACTTAAAAAAA | 15051 |
| YJM1273 | 13929 | ---------------------------------------------------------------------------------------------------- | 13928 |
| NCYC3585 | 14025 | ---------------------------------------------------------------------------------------------------- | 14024 |
| YJM1401 | 14620 | ---------------------------------------------------------------------------------------------------- | 14619 |
| NCYC3594 | 13186 | ---------------------------------------------------------------------------------------------------- | 13185 |
| YJM1078 | 14766 | ACATGGTTTTATAAAGAATATAAGTTGTCAAACAGCAATTTGAGAAGTTAGAAATATATTTGGTGGAAGTAATTGATTTATTGAAGTAGACTTAAAAAAA | 14865 |
| YJM1439 | 15753 | ACATGGTTTTAGAAAGAATATAAGTTGTCAAACAGCAATTTGAGAAGTTAGAAATATATTTGGTGGAAGTAATTGATTTATTGAAGTAGACTTAAAAAAA | 15852 |
| consensus | 19201 | -----------.---------------------------------------------------------------------------------------- | 19300 |
|
| S288C | 14982 | TGTTTTGATACAATTTCTCATGATTTAATTATTAAAGAATTAAAAAGATATATTTCAGATAAAGGTTTTATTGATTTAGTATATAAATTATTAAGAGCTG | 15081 |
| YJM1388 | 15418 | TGTTTTGATACAATTTCTCATGATTTAATTATTAAAGAATTAAAAAGATATATTTCAGATAAAGGTTTTATTGATTTAGTATATAAATTATTAAGAGCTG | 15517 |
| YJM789 | 15052 | TGTTTTGATACAATTTCTCATGATTTAATTATTAAAGAATTAAAAAGATATATTTCAGATAAAGGTTTTATTGATTTAGTATATAAATTATTAAGAGCTG | 15151 |
| YJM1273 | 13929 | ---------------------------------------------------------------------------------------------------- | 13928 |
| NCYC3585 | 14025 | ---------------------------------------------------------------------------------------------------- | 14024 |
| YJM1401 | 14620 | ---------------------------------------------------------------------------------------------------- | 14619 |
| NCYC3594 | 13186 | ---------------------------------------------------------------------------------------------------- | 13185 |
| YJM1078 | 14866 | TGTTTTGATACAATTTCTCATGATTTAATTATTAAAGAATTAAAAAGATATATTTCAGATAAAGGTTTTATTGATTTAGTATATAAATTATTAAGAGCTG | 14965 |
| YJM1439 | 15853 | TGTTTTGATACAATTTCTCATGATTTAATTATTAAAGAATTAAAAAGATATATTTCAGATAAAGGTTTTATTGATTTAGTATATAAATTATTAAGAGCTG | 15952 |
| consensus | 19301 | ---------------------------------------------------------------------------------------------------- | 19400 |
|
| S288C | 15082 | GTTATATTGATGAGAAAGGAACTTATCATAAACCTATATTAGGTTTACCTCAAGGATCATTAATTAGTCCTATCTTATGTAATATTGTAATAACATTGGT | 15181 |
| YJM1388 | 15518 | GTTATATTGATGAGAAAGGAACTTATCATAAACCTATATTAGGTTTACCTCAAGGATCATTAATTAGTCCTATCTTATGTAATATTGTAATAACATTGGT | 15617 |
| YJM789 | 15152 | GTTATATTGATGAGAAAGGAACTTATCATAAACCTATATTAGGTTTACCTCAAGGATCATTAATTAGTCCTATCTTATGTAATATTGTAATAACATTGGT | 15251 |
| YJM1273 | 13929 | ---------------------------------------------------------------------------------------------------- | 13928 |
| NCYC3585 | 14025 | ---------------------------------------------------------------------------------------------------- | 14024 |
| YJM1401 | 14620 | ---------------------------------------------------------------------------------------------------- | 14619 |
| NCYC3594 | 13186 | ---------------------------------------------------------------------------------------------------- | 13185 |
| YJM1078 | 14966 | GTTATATTGATGAGAAAGGAACTTATCATAAACCTATATTAGGTTTACCTCAAGGATCATTAATTAGTCCTATCTTATGTAATATTGTAATAACATTGGT | 15065 |
| YJM1439 | 15953 | GTTATATTGATGAGAAAGGAACTTATCATAAACCTATATTAGGTTTACCTCAAGGATCATTAATTAGTCCTATCTTATGTAATATTGTAATAACATTGGT | 16052 |
| consensus | 19401 | ---------------------------------------------------------------------------------------------------- | 19500 |
|
| S288C | 15182 | AGATAATTGATTAGAAGATTATATTAATTTATATAATAAAGGTAAAGTTAAAAAACAACATCCTACATATAAAAAATTATCAAGAATAATTGCAAAAGCT | 15281 |
| YJM1388 | 15618 | AGATAATTGATTAGAAGATTATATTAATTTATATAATAAAGGTAAAGTTAAAAAACAACATCCTACATATAAAAAATTATCAAGAATAATTGCAAAAGCT | 15717 |
| YJM789 | 15252 | AGATAATTGATTAGAAGATTATATTAATTTATATAATAAAGGTAAAGTTAAAAAACAACATCCTACATATAAAAAATTATCAAGAATAATTGCAAAAGCT | 15351 |
| YJM1273 | 13929 | ---------------------------------------------------------------------------------------------------- | 13928 |
| NCYC3585 | 14025 | ---------------------------------------------------------------------------------------------------- | 14024 |
| YJM1401 | 14620 | ---------------------------------------------------------------------------------------------------- | 14619 |
| NCYC3594 | 13186 | ---------------------------------------------------------------------------------------------------- | 13185 |
| YJM1078 | 15066 | AGATAATTGATTAGAAGATTATATTAATTTATATAATAAAGGTAAAGTTAAAAAACAACATCCTACATATAAAAAATTATCAAGAATAATAGCAAAAGCT | 15165 |
| YJM1439 | 16053 | AGATAATTGATTAGAAGATTATATTAATTTATATAATAAAGGTAAAGTTAAAAAACAACATCCTACATATAAAAAATTATCAAGAATAATTGCAAAAGCT | 16152 |
| consensus | 19501 | ------------------------------------------------------------------------------------------.--------- | 19600 |
|
| S288C | 15282 | AAAATATTTTCGACAAGATTAAAATTACATAAAGAAAGAGCTAAAGGCCCACTATTTATTTATAATGATCCTAATTTCAAGAGAATAAAATACGTTAGAT | 15381 |
| YJM1388 | 15718 | AAAATATTTTCGACAAGATTAAAATTACATAAAGAAAGAGCTAAAGGCCCACTATTTATTTATAATGATCCTAATTTCAAGAGAATAAAATACGTTAGAT | 15817 |
| YJM789 | 15352 | AAAATATTTTCGACAAGATTAAAATTACATAAAGAAAGAGCTAAAGGCCTACTATTTATTTATAATGATCCTAATTTCAAGAGAATAAAATACGTTAGAT | 15451 |
| YJM1273 | 13929 | ---------------------------------------------------------------------------------------------------- | 13928 |
| NCYC3585 | 14025 | ---------------------------------------------------------------------------------------------------- | 14024 |
| YJM1401 | 14620 | ---------------------------------------------------------------------------------------------------- | 14619 |
| NCYC3594 | 13186 | ---------------------------------------------------------------------------------------------------- | 13185 |
| YJM1078 | 15166 | AAAATATTTTCGACAAGATTAAAATTACATAAAGAAAGAGCTAAAGGCCTACTATTTATTTATAATGATCCTAATTTCAAGAGAATAAAATACGTTAGAT | 15265 |
| YJM1439 | 16153 | AAAATATTTTCGACAAGATTAAAATTACATAAAGAAAGAGCTAAAGGGCCACTATTTATTTATAATGATCCTAATTTCAAGAGAATAAAATACGTTAGAT | 16252 |
| consensus | 19601 | -----------------------------------------------.-.-------------------------------------------------- | 19700 |
|
| S288C | 15382 | ATGCAGATGATATTTTAATTGGGGTATTAGGTTCAAAAAATGATTGTAAAATAATCAAAAGAGATTTAAACAATTTTTTAAATTCATTAGGTTTAACTAT | 15481 |
| YJM1388 | 15818 | ATGCAGATGATATTTTAATTGGGGTATTAGGTTCAAAAAATGATTGTAAAATAATCAAAAGAGATTTAAACAATTTTTTAAATTCATTAGGTTTAACTAT | 15917 |
| YJM789 | 15452 | ATGCAGATGATATTTTAATTGGGGTATTAGGTTCAAAAAATGATTGTAAAATAATCAAAAGAGATTTAAACAATTTTTTAAATTCATTAGGTTTAACTAT | 15551 |
| YJM1273 | 13929 | ---------------------------------------------------------------------------------------------------- | 13928 |
| NCYC3585 | 14025 | ---------------------------------------------------------------------------------------------------- | 14024 |
| YJM1401 | 14620 | ---------------------------------------------------------------------------------------------------- | 14619 |
| NCYC3594 | 13186 | ---------------------------------------------------------------------------------------------------- | 13185 |
| YJM1078 | 15266 | ATGCAGATGATATTTTAATTGGGGTATTAGGTTCAAAAAATGATTGTAAAATAATCAAAAGAGATTTAAACAATTTTTTAAATTCATTAGGTTTAACTAT | 15365 |
| YJM1439 | 16253 | ATGCAGATGATATTTTAATTGGGGTATTAGGTTCAAAAAATGATTGTAAAATAATCAAAAGAGATTTAAACAATTTTTTAAATTCATTAGGTTTAACTAT | 16352 |
| consensus | 19701 | ---------------------------------------------------------------------------------------------------- | 19800 |
|
| S288C | 15482 | AAATGAAGAAAAAACTTTAATTACTTGTGCAACTGAACTACCAGCAAGATTTTTAGGTTATAATATTTCAATTACACCTTTAAAAAGAATACCTACAGTT | 15581 |
| YJM1388 | 15918 | AAATGAAGAAAAAACTTTAATTACTTGTGCAACTGAACTACCAGCAAGATTTTTAGGTTATAATATTTCAATTACACCTTTAAAAAGAATACCTACAGTT | 16017 |
| YJM789 | 15552 | AAATGAAGAAAAAACTTTAATTACTTGTGCAACTGAACTACCAGCAAGATTTTTAGGTTATAATATTTCAATTACACCTTTAAAAAGAATACCTACAGTT | 15651 |
| YJM1273 | 13929 | ---------------------------------------------------------------------------------------------------- | 13928 |
| NCYC3585 | 14025 | ---------------------------------------------------------------------------------------------------- | 14024 |
| YJM1401 | 14620 | ---------------------------------------------------------------------------------------------------- | 14619 |
| NCYC3594 | 13186 | ---------------------------------------------------------------------------------------------------- | 13185 |
| YJM1078 | 15366 | AAATGAAGAAAAAACTTTAATTACTTGTGCAACTGAACTACCAGCAAGATTTTTAGGTTATAATATTTCAATTACACCTTTAAAAAGAATACCTACAGTT | 15465 |
| YJM1439 | 16353 | AAATGAAGAAAAAACTTTAATTACTTGTGCAACTGAACTACCAGCAAGATTTTTAGGTTATAATATTTCAATTACACCTTTAAAAAGAATACCTACAGTT | 16452 |
| consensus | 19801 | ---------------------------------------------------------------------------------------------------- | 19900 |
|
| S288C | 15582 | ACTAAACTAATTAGAGGTAAACTTATTAGAAGTAGAAATACAACTAGACCTATTATTAATGCACCAATTAGAGATATTATCAATAAATTAGCTACTAATG | 15681 |
| YJM1388 | 16018 | ACTAAACTAATTAGAGGTAAACTTATTAGAAGTAGAAATACAACTAGACCTATTATTAATGCACCAATTAGAGATATTATCAATAAATTAGCTACTAATG | 16117 |
| YJM789 | 15652 | ACTAAACTAATTAGAGGTAAACTTATTAGAAGTAGAAATACAACTAGACCTATTATTAATGCACCAATTAGAGATATTATCAATAAATTAGCTACTAATG | 15751 |
| YJM1273 | 13929 | ---------------------------------------------------------------------------------------------------- | 13928 |
| NCYC3585 | 14025 | ---------------------------------------------------------------------------------------------------- | 14024 |
| YJM1401 | 14620 | ---------------------------------------------------------------------------------------------------- | 14619 |
| NCYC3594 | 13186 | ---------------------------------------------------------------------------------------------------- | 13185 |
| YJM1078 | 15466 | ACTAAACTAATTAGAGGTAAACTTATTAGAAGTAGAAATACAACTAGACCTATTATTAATGCACCAATTAGAGATATTATCAATAAATTAGCTACTAATG | 15565 |
| YJM1439 | 16453 | ACTAAACTAATTAGAGGTAAACTTATTAGAAGTAGAAATACAACTAGACCTATTATTAATGCACCAATTAGAGATATTATCAATAAATTAGCTACTAATG | 16552 |
| consensus | 19901 | ---------------------------------------------------------------------------------------------------- | 20000 |
|
| S288C | 15682 | GATATTGTAAGCATAATAAAAATGGTAGAATAGGAGTGCCTACAAGAGTAGGTAGATGACTATATGAAGAACCTAGAACAATTATTAATAATTATAAAGC | 15781 |
| YJM1388 | 16118 | GATATTGTAAGCATAATAAAAATGGTAGAATAGGAGTGCCTACAAGAGTAGGTAGATGACTATATGAAGAACCTAGAACAATTATTAATAATTATAAAGC | 16217 |
| YJM789 | 15752 | GATATTGTAAGCATAATAAAAATGGTAGAATAGGAGTGCCTACAAGAGTAGGTAGATGACTATATGAAGAACCTAGAACAATTATTAATAATTATAAAGC | 15851 |
| YJM1273 | 13929 | ---------------------------------------------------------------------------------------------------- | 13928 |
| NCYC3585 | 14025 | ---------------------------------------------------------------------------------------------------- | 14024 |
| YJM1401 | 14620 | ---------------------------------------------------------------------------------------------------- | 14619 |
| NCYC3594 | 13186 | ---------------------------------------------------------------------------------------------------- | 13185 |
| YJM1078 | 15566 | GATATTGTAAGCATAATAAAAATGGTAGAATAGGAGTGCCTACAAGAGTAGGTAGATGACTATATGAAGAACCTAGAACAATTATTAATAATTATAAAGC | 15665 |
| YJM1439 | 16553 | GATATTGTAAGCATAATAAAAATGGTAGAATAGGAGTGCCTACAAGAGTAGGTAGATGACTATATGAAGAACCTAGAACAATTATTAATAATTATAAAGC | 16652 |
| consensus | 20001 | ---------------------------------------------------------------------------------------------------- | 20100 |
|
| S288C | 15782 | GTTAGGTAGAGGTATCTTAAATTATTATAAATTAGCTACTAATTATAAAAGATTAAGAGAAAGAATCTATTACGTATTATATTATTCATGTGTATTAACT | 15881 |
| YJM1388 | 16218 | GTTAGGTAGAGGTATCTTAAATTATTATAAATTAGCTACTAATTATAAAAGATTAAGAGAAAGAATCTATTACGTATTATATTATTCATGTGTATTAACT | 16317 |
| YJM789 | 15852 | GTTAGGTAGAGGTATCTTAAATTATTATAAATTAGCTACTAATTATAAAAGATTAAGAGAAAGAATCTATTACGTATTATATTATTCATGTGTATTAACT | 15951 |
| YJM1273 | 13929 | ---------------------------------------------------------------------------------------------------- | 13928 |
| NCYC3585 | 14025 | ---------------------------------------------------------------------------------------------------- | 14024 |
| YJM1401 | 14620 | ---------------------------------------------------------------------------------------------------- | 14619 |
| NCYC3594 | 13186 | ---------------------------------------------------------------------------------------------------- | 13185 |
| YJM1078 | 15666 | GTTAGGTAGAGGTATCTTAAATTATTATAAATTAGCTACTAATTATAAAAGATTAAGAGAAAGAATCTATTACGTATTATATTATTCATGTGTATTAACT | 15765 |
| YJM1439 | 16653 | GTTAGGTAGAGGTATCTTAAATTATTATAAATTAGCTACTAATTATAAAAGATTAAGAGAAAGAATCTATTACGTATTATATTATTCATGTGTATTAACT | 16752 |
| consensus | 20101 | ---------------------------------------------------------------------------------------------------- | 20200 |
|
| S288C | 15882 | TTAGCTAGTAAATATAGATTAAAAACAATAAGTAAAACTATTAAAAAATTTGGTTATAATTTAAATATTATTGAAAATGATAAATTAATTGCCAATTTTC | 15981 |
| YJM1388 | 16318 | TTAGCTAGTAAATATAGATTAAAAACAATAAGTAAAACTATTAAAAAATTTGGTTATAATTTAAATATTATTGAAAATGATAAATTAATTGTCAATTTTC | 16417 |
| YJM789 | 15952 | TTAGCTAGTAAATATAGATTAAAAACAATAAGTAAAACTATTAAAAAATTTGGTTATAATTTAAATATTATTGAAAATGATAAATTAATTGCCAATTTTC | 16051 |
| YJM1273 | 13929 | ---------------------------------------------------------------------------------------------------- | 13928 |
| NCYC3585 | 14025 | ---------------------------------------------------------------------------------------------------- | 14024 |
| YJM1401 | 14620 | ---------------------------------------------------------------------------------------------------- | 14619 |
| NCYC3594 | 13186 | ---------------------------------------------------------------------------------------------------- | 13185 |
| YJM1078 | 15766 | TTAGCTAGTAAATATAGATTAAAAACAATAAGTAAAACTATTAAAAAATTTGGTTATAATTTAAATATTATTGAAAATGATAAATTAATTGCCAATTTTC | 15865 |
| YJM1439 | 16753 | TTAGCTAGTAAATATAGATTAAAAACAATAAGTAAAACTATTAAAAAATTTGGTTATAATTTAAATATTATTGAAAATGATAAATTAATTGCCAATTTTC | 16852 |
| consensus | 20201 | -------------------------------------------------------------------------------------------.-------- | 20300 |
|
| S288C | 15982 | CAAGAAATACTTTTGATAATATCAAAAAAATTGAAAATCATGGTATATTTATATATATATCAGAAGCTAAAGTAACTGATCCTTTTGAATATATCGATTC | 16081 |
| YJM1388 | 16418 | CAAGAAATACTTTTGATAATATCAAAAAAATTGAAAATCATGGTATATTTATATATATATCAGAAGCTAAAGTAACTGATCCTTTTGAATATATCGATTC | 16517 |
| YJM789 | 16052 | CAAGAAATACTTTTGATAATATCAAAAAAATTGAAAATCATGGTATATTTATATATATATCAGAAGCTAAAGTAACTGATCCTTTTGAATATATCGATTC | 16151 |
| YJM1273 | 13929 | ---------------------------------------------------------------------------------------------------- | 13928 |
| NCYC3585 | 14025 | ---------------------------------------------------------------------------------------------------- | 14024 |
| YJM1401 | 14620 | ---------------------------------------------------------------------------------------------------- | 14619 |
| NCYC3594 | 13186 | ---------------------------------------------------------------------------------------------------- | 13185 |
| YJM1078 | 15866 | CAAGAAATACTTTTGATAATATCAAAAAAATTGAAAATCATGGTATATTTATATATATATCAGAAGCTAAAGTAACTGATCCTTTTGAATATATCGATTC | 15965 |
| YJM1439 | 16853 | CAAGAAATACTTTTGATAATATCAAAAAAATTGAAAATCATGGTATATTTATATATATATCAGAAGCTAAAGTAACTGATCCTTTTGAATATATCGATTC | 16952 |
| consensus | 20301 | ---------------------------------------------------------------------------------------------------- | 20400 |
|
| S288C | 16082 | AATTAAATATATATTACCTACAGCTAAAGCTAATTTTAATAAACCTTGTAGTATTTGTAATTCAACTATTGATGTAGAAATACATCATGTTAAACAATTA | 16181 |
| YJM1388 | 16518 | AATTAAATATATATTACCTACAGCTAAAGCTAATTTTAATAAACCTTGTAGTATTTGTAATTCAACTATTGATGTAGAAATACATCATGTTAAACAATTA | 16617 |
| YJM789 | 16152 | AATTAAATATATATTACCTACAGCTAAAGCTAATTTTAATAAACCTTGTAGTATTTGTAATTCAACTATTGATGTAGAAATACATCATGTTAAACAATTA | 16251 |
| YJM1273 | 13929 | ---------------------------------------------------------------------------------------------------- | 13928 |
| NCYC3585 | 14025 | ---------------------------------------------------------------------------------------------------- | 14024 |
| YJM1401 | 14620 | ---------------------------------------------------------------------------------------------------- | 14619 |
| NCYC3594 | 13186 | ---------------------------------------------------------------------------------------------------- | 13185 |
| YJM1078 | 15966 | AATTAAATATATATTACCTACAGCTAAAGCTAATTTTAATAAACCTTGTAGTATTTGTAATTCAACTATTGATGTAGAAATACATCATGTTAAACAATTA | 16065 |
| YJM1439 | 16953 | AATTAAATATATATTACCTACAGCTAAAGCTAATTTTAATAAACCTTGTAGTATTTGTAATTCAACTATTGATGTAGAAATACATCATGTTAAACAATTA | 17052 |
| consensus | 20401 | ---------------------------------------------------------------------------------------------------- | 20500 |
|
| S288C | 16182 | CATAGAGGTATATTAAAAGCACTTAAAGATTATATTCTAGGTAGAATAATTACCATAAACAGAAAACAAATTCCATTATGTAAACAATGTCATATTAAAA | 16281 |
| YJM1388 | 16618 | CATAGAGGTATATTAAAAGCACTTAAAGATTATATTCTAGGTAGAATAATTACCATAAACAGAAAACAAATTCCATTATGTAAACAATGTCATATTAAAA | 16717 |
| YJM789 | 16252 | CATAGAGGTATATTAAAAGCACTTAAAGATTATATTTTAGGTAGAATAATTACCATAAACAGAAAACAAATTCCATTATGTAAACAATGTCATATTAAAA | 16351 |
| YJM1273 | 13929 | ---------------------------------------------------------------------------------------------------- | 13928 |
| NCYC3585 | 14025 | ---------------------------------------------------------------------------------------------------- | 14024 |
| YJM1401 | 14620 | ---------------------------------------------------------------------------------------------------- | 14619 |
| NCYC3594 | 13186 | ---------------------------------------------------------------------------------------------------- | 13185 |
| YJM1078 | 16066 | CATAGAGGTATATTAAAAGCACTTAAAGATTATATTTTAGGTAGAATAATTACCATAAACAGAAAACAAATTCCATTATGTAAACAATGTCATATTAAAA | 16165 |
| YJM1439 | 17053 | CATAGAGGTATATTAAAAGCACTTAAAGATTATATTCTAGGTAGAATAATTACCATAAACAGAAAACAAATTCCATTATGTAAACAATGTCATATTAAAA | 17152 |
| consensus | 20501 | ------------------------------------.--------------------------------------------------------------- | 20600 |
|
| S288C | 16282 | CACATAAAAATAAATTTAAAAATATAGGACCTGGTATATAAAATCTATTATTAATGATACTCAATATGGAAAGCCGTATGATGGGAAACTATCACGTACG | 16381 |
| YJM1388 | 16718 | CACATAAAAATAAATTTAAAAATATAGGACCTGGTATATAAAATCTATTATTAATGATACTCAATATGGAAAGCCGTATGATGGGAAACTATCACGTACG | 16817 |
| YJM789 | 16352 | CACATAAAAATAAATTTAAAAATATAGGACCTGGTATATAAAATCTATTATTAATGATACTCAATATGGAAAGCCGTATGATGGGAAACTATCACGTACG | 16451 |
| YJM1273 | 13929 | ---------------------------------------------------------------------------------------------------- | 13928 |
| NCYC3585 | 14025 | ---------------------------------------------------------------------------------------------------- | 14024 |
| YJM1401 | 14620 | ---------------------------------------------------------------------------------------------------- | 14619 |
| NCYC3594 | 13186 | ---------------------------------------------------------------------------------------------------- | 13185 |
| YJM1078 | 16166 | CACATAAAAATAAATTTAAAAATATAGGACCTGGTATATAAAATCTATTATTAATGATACTCAATATGGAAAGCCGTATGATGGGAAACTATCACGTACG | 16265 |
| YJM1439 | 17153 | CACATAAAAATAAATTTAAAAATATAGGACCTGGTATATAAAATCTATTATTAATGATACTCAATATGGAAAGCCGTATGATGGGAAACTATCACGTACG | 17252 |
| consensus | 20601 | ---------------------------------------------------------------------------------------------------- | 20700 |
|
| S288C | 16382 | GTTTGGGAAAGGCTCTTTAACACGTGGCAACATAGGTTAATTTGCTATTTCATTTTTAGTAGTTGGTCATGCTGTATTAATGATTTTCTGTGCGCCGTTT | 16481 |
| YJM1388 | 16818 | GTTTGGGAAAGGCTCTTTAACACGTGGCAACATAGGTTAATTTGCTATTTCATTTTTAGTAGTTGGTCATGCTGTATTAATGATTTTCTGTGCGCCGTTT | 16917 |
| YJM789 | 16452 | GTTTGGGAAAGGCTCTTTAACACGTGGCAACATAGGTTAATTTGCTATTTCATTTTTAGTAGTTGGTCATGCTGTATTAATGATTTTCTGTGCGCCGTTT | 16551 |
| YJM1273 | 13929 | -----------------------------------------------------TTTTAGTAGTTGGTCATGCTGTATTAATGATTTTCTGTGCGCCGTTT | 13975 |
| NCYC3585 | 14025 | -----------------------------------------------------TTTTAGTAGTTGGTCATGCTGTATTAATAATTTTCT----------- | 14060 |
| YJM1401 | 14620 | -----------------------------------------------------TTTTAGTAGTTGGTCATGCTGTATTAATAATTTTCT----------- | 14655 |
| NCYC3594 | 13186 | -----------------------------------------------------TTTTAGTAGTTGGTCATGCTGTATTAATGATTTTCTGTGCGCCGTTT | 13232 |
| YJM1078 | 16266 | GTTTGGGAAAGGCTCTTTAACACGTGGCAACATAGGTTAATTTGCTATTTCATTTTTAGTAGTTGGTCATGCTGTATTAATAATTTTCT----------- | 16354 |
| YJM1439 | 17253 | GTTTGGGAAAGGCTCTTTAACACGTGGCAACATAGGTTAATTTGCTATTTCATTTTTAGTACTTGGTCATGCTGTATTAATAATTTTCT----------- | 17341 |
| consensus | 20701 | -----------------------------------------------------\*\*\*\*\*\*\*\*.\*\*\*\*\*\*\*\*\*\*\*\*\*\*\*\*\*\*\*.\*\*\*\*\*\*\*----------- | 20800 |
|
| S288C | 16482 | CGCTTAATTTATCACTGTATTGAAGTGTTAATTGATAAACATATCTCTGTTTATTCAATTAATGAAAACTTTACCGTATCATTTTGGTTCTGATTATTAG | 16581 |
| YJM1388 | 16918 | CGCTTAATTTATCACTGTATTGAAGTGTTAATTGATAAACATATCTCTGTTTATTCAATTAATGAAAACTTTACCGTATCATTTTGGTTCTGATTATTAG | 17017 |
| YJM789 | 16552 | CGCTTAATTTATCACTGTATTGAAGTGTTAATTGATAAACATATCTCTGTTTATTCAATTAATGAAAACTTTACCGTATCATTTTGGTTCTGATTATTAG | 16651 |
| YJM1273 | 13976 | CGCTTAATTTATCACTGTATTGAAGTGTTAATTGATAAACATATCTCTGTTTATTCAATTAATGAAAACTTTACCGTATCATTTTGGTTCTGATTATTAG | 14075 |
| NCYC3585 | 14061 | ---------------------------------------------------------------------------------------------------- | 14060 |
| YJM1401 | 14656 | ---------------------------------------------------------------------------------------------------- | 14655 |
| NCYC3594 | 13233 | CGCTTAATTTATCACTGTATTGAAGTGTTAATTGATAAACATATCTCTGTTTATTCAATTAATGAAAACTTTACCGTATCATTTTGGTTCTGATTATTAG | 13332 |
| YJM1078 | 16355 | ---------------------------------------------------------------------------------------------------- | 16354 |
| YJM1439 | 17342 | ---------------------------------------------------------------------------------------------------- | 17341 |
| consensus | 20801 | ---------------------------------------------------------------------------------------------------- | 20900 |
|
| S288C | 16582 | TAGTAACATACATAGTATTTAGATACGTAAACCATATGGCTTACCCAGTTGGGGCCAACTCAACGGGGACAATAGCATGCCATAAAAGCGCTGGAGTAAA | 16681 |
| YJM1388 | 17018 | TAGTAACATACATAGTATTTAGATACGTAAACCATATGGCTTACCCAGTTGGGGCCAACTCAACGGGGACAATAGCATGCCATAAAAGCGCTGGAGTAAA | 17117 |
| YJM789 | 16652 | TAGTAACATACATAGTATTTAGATACGTAAACCATATGGCTTACCCAGTTGGGGCCAACTCAACGGGGACAATAGCATGCCATAAAAGCGCTGGAGTAAA | 16751 |
| YJM1273 | 14076 | TAGTAACATACATAGTATTTAGATACGTAAACCATATGGCTTACCCAGTTGGGGCCAACTCAACGGGGACAATAGCATGCCATAAAAGCGCTGGAGTAAA | 14175 |
| NCYC3585 | 14061 | ---------------------------------------------------------------------------------------------------- | 14060 |
| YJM1401 | 14656 | ---------------------------------------------------------------------------------------------------- | 14655 |
| NCYC3594 | 13333 | TAGTAACATACATAGTATTTAGATACGTAAACCATATGGCTTACCCAGTTGGGGCCAACTCAACGGGGACAATAGCATGCCATAAAAGCGCTGGAGTAAA | 13432 |
| YJM1078 | 16355 | ---------------------------------------------------------------------------------------------------- | 16354 |
| YJM1439 | 17342 | ---------------------------------------------------------------------------------------------------- | 17341 |
| consensus | 20901 | ---------------------------------------------------------------------------------------------------- | 21000 |
|
| S288C | 16682 | ACAGCCAGCGCAAGGTAAGAACTGTCCGATGGCTAGGTTAACGAATTCCTGTAAAGAATGTTTAGGGTTCTCATTAACTCCTTCCCACTTGGGGATTGTG | 16781 |
| YJM1388 | 17118 | ACAGCCAGCGCAAGGTAAGAACTGTCCGATGGCTAGGTTAACGAATTCCTGTAAAGAATGTTTAGGGTTCTCATTAACTCCTTCCCACTTGGGGATTGTG | 17217 |
| YJM789 | 16752 | ACAGCCAGCGCAAGGTAAGAACTGTCCGATGGCTAGGTTAACGAATTCCTGTAAAGAATGTTTAGGGTTCTCATTAACTCCTTCCCACTTGGGGATTGTG | 16851 |
| YJM1273 | 14176 | ACAGCCAGCGCAAGGTAAGAACTGTCCGATGGCTAGGTTAACGAATTCCTGTAAAGAATGTTTAGGGTTCTCATTAACTCCTTCCCACTTGGGGATTGTG | 14275 |
| NCYC3585 | 14061 | ---------------------------------------------------------------------------------------------------- | 14060 |
| YJM1401 | 14656 | ---------------------------------------------------------------------------------------------------- | 14655 |
| NCYC3594 | 13433 | ACAGCCAGCGCAAGGTAAGAACTGTCCGATGGCTAGGTTAACGAATTCCTGTAAAGAATGTTTAGGGTTCTCATTAACTCCTTCCCACTTGGGGATTGTG | 13532 |
| YJM1078 | 16355 | ---------------------------------------------------------------------------------------------------- | 16354 |
| YJM1439 | 17342 | ---------------------------------------------------------------------------------------------------- | 17341 |
| consensus | 21001 | ---------------------------------------------------------------------------------------------------- | 21100 |
|
| S288C | 16782 | ATTCATGCTTATGTATTGGAAGAAGAGGTACACGAGTTAACCAAAAATGAATCATTAGCTTTAAGTAAAAGTTGACATTTGGAGGGCTGTACGAGTTCAA | 16881 |
| YJM1388 | 17218 | ATTCATGCTTATGTATTGGAAGAAGAGGTACACGAGTTAACCAAAAATGAATCATTAGCTTTAAGTAAAAGTTGACATTTGGAGGGCTGTACGAGTTCAA | 17317 |
| YJM789 | 16852 | ATTCATGCTTATGTATTGGAAGAAGAGGTACACGAGTTAACCAAAAATGAATCATTAGCTTTAAGTAAAAGTTGACATTCGGAGGGCTGTACGAGTTCAA | 16951 |
| YJM1273 | 14276 | ATTCATGCTTATGTATTGGAAGAAGAGGTACACGAGTTAACCAAAAATGAATCATTAGCTTTAAGTAAAAGTTGACATTCGGAGGGCTGTACGAGTTCAA | 14375 |
| NCYC3585 | 14061 | ---------------------------------------------------------------------------------------------------- | 14060 |
| YJM1401 | 14656 | ---------------------------------------------------------------------------------------------------- | 14655 |
| NCYC3594 | 13533 | ATTCATGCTTATGTATTGGAAGAAGAGGTACACGAGTTAACCAAAAATGAATCATTAGCTTTAAGTAAAAGTTGACATTCGGAGGGCTGTACGAGTTCAA | 13632 |
| YJM1078 | 16355 | ---------------------------------------------------------------------------------------------------- | 16354 |
| YJM1439 | 17342 | ---------------------------------------------------------------------------------------------------- | 17341 |
| consensus | 21101 | -------------------------------------------------------------------------------.-------------------- | 21200 |
|
| S288C | 16882 | ATGGAAAATTAAGAAATACGGGATTGTCCGAAAGGGGAAACCCTGGGGATAACGGAGTCTTCATAGTACCCAAATTTAATTTAAATAAAGTGAGATACTT | 16981 |
| YJM1388 | 17318 | ATGGAAAATTAAGAAATACGGGATTGTCCGAAAGGGGAAACCCTGGGGATAACGGAGTCTTCATAGTACCCAAATTTAATTTAAATAAAGTGAGATACTT | 17417 |
| YJM789 | 16952 | ATGGAAAATTAAGAAATACGGGATTGTCCGAAAGGGGAAACCCTGGGGATAACGGAGTCTTCATAGTACCCAAATTTAATTTAAATAAAGTGAGATACTT | 17051 |
| YJM1273 | 14376 | ATGGAAAATTAAGAAATACGGGATTGTCCGAAAGGGGAAACCCTGGGGATAACGGAGTCTTCATAGTACCCAAATTTAATTTAAATAAAGTGAGATACTT | 14475 |
| NCYC3585 | 14061 | ---------------------------------------------------------------------------------------------------- | 14060 |
| YJM1401 | 14656 | ---------------------------------------------------------------------------------------------------- | 14655 |
| NCYC3594 | 13633 | ATGGAAAATTAAGAAATACGGGATTGTCCGAAAGGGGAAACCCTGGGGATAACGGAGTCTTCATAGTACCCAAATTTAATTTAAATAAAGTGAGATACTT | 13732 |
| YJM1078 | 16355 | ---------------------------------------------------------------------------------------------------- | 16354 |
| YJM1439 | 17342 | ---------------------------------------------------------------------------------------------------- | 17341 |
| consensus | 21201 | ---------------------------------------------------------------------------------------------------- | 21300 |
|
| S288C | 16982 | TAGTACTTTATCTAAATTAAATGCAAGGAAGGAAGACAGTTTAGCGTATTTAACAAAGATTAATACTACGGATTTTTCCGAGTTAAATAAATTAATAGAA | 17081 |
| YJM1388 | 17418 | TAGTACTTTATCTAAATTAAATGCAAGGAAGGAAGACAGTTTAGCGTATTTAACAAAGATTAATACTACGGATTTTTCCGAGTTAAATAAATTAATAGAA | 17517 |
| YJM789 | 17052 | TAGTACTTTATCTAAATTAAATGCAAGGAAGGAAGACAGTTTAGCGTATTTAACAAAGATTAATACTACGGATTTTTCCGAGTTAAATAAATTAATAGAA | 17151 |
| YJM1273 | 14476 | TAGTACTTTATCTAAATTAAATGCAAGGAAGGAAGACAGTTTAGCGTATTTAACAAAGATTAATACTACGGATTTTTCCGAGTTAAATAAATTAATAGAA | 14575 |
| NCYC3585 | 14061 | ---------------------------------------------------------------------------------------------------- | 14060 |
| YJM1401 | 14656 | ---------------------------------------------------------------------------------------------------- | 14655 |
| NCYC3594 | 13733 | TAGTACTTTATCTAAATTAAATGCAAGGAAGGAAGACAGTTTAGCGTATTTAACAAAGATTAATACTACGGATTTTTCCGAGTTAAATAAATTAATAGAA | 13832 |
| YJM1078 | 16355 | ---------------------------------------------------------------------------------------------------- | 16354 |
| YJM1439 | 17342 | ---------------------------------------------------------------------------------------------------- | 17341 |
| consensus | 21301 | ---------------------------------------------------------------------------------------------------- | 21400 |
|
| S288C | 17082 | AATAATCATAATAAACTTGAAACCATTAATACTAGAATTTTAAAATTAATGTCAGATATTAGAATGTTATTAATTGCTTATAATAAAATTAAAAGTAAGA | 17181 |
| YJM1388 | 17518 | AATAATCATAATAAACTTGAAACCATTAATACTAGAATTTTAAAATTAATGTCAGATATTAGAATGTTATTAATTGCTTATAATAAAATTAAAAGTAAGA | 17617 |
| YJM789 | 17152 | AATAATCATAATAAACTTGAAACCATTAATACTAGAATTTTAAAATTAATGTCAGATATTAGAATGTTATTAATTGCTTATAATAAAATTAAAAGTAAGA | 17251 |
| YJM1273 | 14576 | AATAATCATAATAAACTTGAAACCATTAATACTAGAATTTTAAAATTAATGTCAGATATTAGAATGTTATTAATTGCTTATAATAAAATTAAAAGTAAGA | 14675 |
| NCYC3585 | 14061 | ---------------------------------------------------------------------------------------------------- | 14060 |
| YJM1401 | 14656 | ---------------------------------------------------------------------------------------------------- | 14655 |
| NCYC3594 | 13833 | AATAATCATAATAAACTTGAAACCATTAATACTAGAATTTTAAAATTAATGTCAGATATTAGAATGTTATTAATTGCTTATAATAAAATTAAAAGTAAGA | 13932 |
| YJM1078 | 16355 | ---------------------------------------------------------------------------------------------------- | 16354 |
| YJM1439 | 17342 | ---------------------------------------------------------------------------------------------------- | 17341 |
| consensus | 21401 | ---------------------------------------------------------------------------------------------------- | 21500 |
|
| S288C | 17182 | AAGGTAATATATCTAAAGGTTCTAATAATATTACCTTAGATGGGATTAATATTTCATATTTAAATAAATTATCTAAAGATATTAACACTAATATGTTTAA | 17281 |
| YJM1388 | 17618 | AAGGTAATATATCTAAAGGTTCTAATAATATTACCTTAGATGGGATTAATATTTCATATTTAAATAAATTATCTAAAGATATTAACACTAATATGTTTAA | 17717 |
| YJM789 | 17252 | AAGGTAATATATCTAAAGGTTCTAATAATATTACCTTAGATGGGATTAATATTTCATATTTAAATAAATTATCTAAAGATATTAACACTAATATGTTTAA | 17351 |
| YJM1273 | 14676 | AAGGTAATATATCTAAAGGTTCTAATAATATTACCTTAGATGGGATTAATATTTCATATTTAAATAAATTATCTAAAGATATTAACACTAATATGTTTAA | 14775 |
| NCYC3585 | 14061 | ---------------------------------------------------------------------------------------------------- | 14060 |
| YJM1401 | 14656 | ---------------------------------------------------------------------------------------------------- | 14655 |
| NCYC3594 | 13933 | AAGGTAATATATCTAAAGGTTCTAATAATATTACCTTAGATGGGATTAATATTTCATATTTAAATAAATTATCTAAAGATATTAACACTAATATGTTTAA | 14032 |
| YJM1078 | 16355 | ---------------------------------------------------------------------------------------------------- | 16354 |
| YJM1439 | 17342 | ---------------------------------------------------------------------------------------------------- | 17341 |
| consensus | 21501 | ---------------------------------------------------------------------------------------------------- | 21600 |
|
| S288C | 17282 | ATTTTCTCCGGTTAGAAGAGTTGAAATTCCTAAAACATCTGGAGGATTTAGACCTTTAAGTGTTGGAAATCCTAGAGAAAAAATTGTACAAGAAAGTATG | 17381 |
| YJM1388 | 17718 | ATTTTCTCCGGTTAGAAGAGTTGAAATTCCTAAAACATCTGGAGGATTTAGACCTTTAAGTGTTGGAAATCCTAGAGAAAAAATTGTACAAGAAAGTATG | 17817 |
| YJM789 | 17352 | ATTTTCTCCGGTTAGAAGAGTTGAAATTCCTAAAACATCTGGAGGATTTAGACCTTTAAGTGTTGGAAATCCTAGAGAAAAAATTGTACAAGAAAGTATG | 17451 |
| YJM1273 | 14776 | ATTTTCTCCGGTT---AGAGTTGAAATTCCTAAAACATCTGGAGGATTTAGACCTTTAAGTGTTGGAAATCCTAGAGAAAAAATTGTACAAGAAAGTATG | 14872 |
| NCYC3585 | 14061 | ---------------------------------------------------------------------------------------------------- | 14060 |
| YJM1401 | 14656 | ---------------------------------------------------------------------------------------------------- | 14655 |
| NCYC3594 | 14033 | ATTTTCTCCGGTTAGAAGAGTTGAAATTCCTAAAACATCTGGAGGATTTAGACCTTTAAGTGTTGGAAATCCTAGAGAAAAAATTGTACAAGAAAGTATG | 14132 |
| YJM1078 | 16355 | ---------------------------------------------------------------------------------------------------- | 16354 |
| YJM1439 | 17342 | ---------------------------------------------------------------------------------------------------- | 17341 |
| consensus | 21601 | ---------------------------------------------------------------------------------------------------- | 21700 |
|
| S288C | 17382 | AGAATAATATTAGAAATTATCTATAATAATAGTTTCTCTTATTATTCTCATGGATTTAGACCTAACTTATCTTGTTTAACAGCTATTATTCAATGTAAAA | 17481 |
| YJM1388 | 17818 | AGAATAATATTAGAAATTATCTATAATAATAGTTTCTCTTATTATTCTCATGGATTTAGACCTAACTTATCTTGTTTAACAGCTATTATTCAATGTAAAA | 17917 |
| YJM789 | 17452 | AGAATAATATTAGAAATTATCTATAATAATAGTTTCTCTTATTATTCTCATGGATTTAGACCTAACTTATCTTGTTTAACAGCTATTATTCAATGTAAAA | 17551 |
| YJM1273 | 14873 | AGAATAATATTAGAAATTATCTATAATAATAGTTTCTCTTATTATTCTCATGGATTTAGACCTAACTTATCTTGTTTAACAGCTATTATTCAATGTAAAA | 14972 |
| NCYC3585 | 14061 | ---------------------------------------------------------------------------------------------------- | 14060 |
| YJM1401 | 14656 | ---------------------------------------------------------------------------------------------------- | 14655 |
| NCYC3594 | 14133 | AGAATAATATTAGAAATTATCTATAATAATAGTTTCTCTTATTATTCTCATGGATTTAGACCTAACTTATCTTGTTTAACAGCTATTATTCAATGTAAAA | 14232 |
| YJM1078 | 16355 | ---------------------------------------------------------------------------------------------------- | 16354 |
| YJM1439 | 17342 | ---------------------------------------------------------------------------------------------------- | 17341 |
| consensus | 21701 | ---------------------------------------------------------------------------------------------------- | 21800 |
|
| S288C | 17482 | ATTATATGCAATACTGTAATTGATTTATTAAAGTAGATTTAAATAAATGCTTTGATACAATTCCACATAATATGTTAATTAATGTATTAAATGAGAGAAT | 17581 |
| YJM1388 | 17918 | ATTATATGCAATACTGTAATTGATTTATTAAAGTAGATTTAAATAAATGCTTTGATACAATTCCACATAATATGTTAATTAATGTATTAAATGAGAGAAT | 18017 |
| YJM789 | 17552 | ATTATATGCAATACTGTAATTGATTTATTAAAGTAGATTTAAATAAATGCTTTGATACAATTCCACATAATATGTTAATTAATGTATTAAATGAGAGAAT | 17651 |
| YJM1273 | 14973 | ATTATATGCAATACTGTAATTGATTTATTAAAGTAGATTTAAATAAATGCTTTGATACAATTCCACATAATATGTTAATTAATGTATTAAATGAGAGAAT | 15072 |
| NCYC3585 | 14061 | ---------------------------------------------------------------------------------------------------- | 14060 |
| YJM1401 | 14656 | ---------------------------------------------------------------------------------------------------- | 14655 |
| NCYC3594 | 14233 | ATTATATGCAATACTGTAATTGATTTATTAAAGTAGATTTAAATAAATGCTTTGATACAATTCCACATAATATGTTAATTAATGTATTAAATGAGAGAAT | 14332 |
| YJM1078 | 16355 | ---------------------------------------------------------------------------------------------------- | 16354 |
| YJM1439 | 17342 | ---------------------------------------------------------------------------------------------------- | 17341 |
| consensus | 21801 | ---------------------------------------------------------------------------------------------------- | 21900 |
|
| S288C | 17582 | CAAAGATAAAGGTTTCATAGACTTATTATATAAATTATTAAGAGCTGGATATGTTGATAAAAATAATAATTATCATAATACAACTTTAGGAATTCCTCAA | 17681 |
| YJM1388 | 18018 | CAAAGATAAAGGTTTCATAGACTTATTATATAAATTATTAAGAGCTGGATATGTTGATAAAAATAATAATTATCATAATACAACTTTAGGAATTCCTCAA | 18117 |
| YJM789 | 17652 | CAAAGATAAAGGTTTCATAGACTTATTATATAAATTATTAAGAGCTGGATATGTTGATAAAAATAATAATTATCATAATACAACTTTAGGAATCCCTCAA | 17751 |
| YJM1273 | 15073 | CAAAGATAAAGGTTTCATAGACTTATTATATAAATTATTAAGAGCTGGATATGTTGATAAAAATAATAATTATCATAATACAACTTTAGGAATCCCTCAA | 15172 |
| NCYC3585 | 14061 | ---------------------------------------------------------------------------------------------------- | 14060 |
| YJM1401 | 14656 | ---------------------------------------------------------------------------------------------------- | 14655 |
| NCYC3594 | 14333 | CAAAGATAAAGGTTTCATAGACTTATTATATAAATTATTAAGAGCTGGATATGTTGATAAAAATAATAATTATCATAATACAACTTTAGGAATCCCTCAA | 14432 |
| YJM1078 | 16355 | ---------------------------------------------------------------------------------------------------- | 16354 |
| YJM1439 | 17342 | ---------------------------------------------------------------------------------------------------- | 17341 |
| consensus | 21901 | ---------------------------------------------------------------------------------------------.------ | 22000 |
|
| S288C | 17682 | GGTAGTGTTGTCAGTCCTATTTTATGTAATATTTTTTTAGATAAATTAGATAAATATTTAGAAAATAAATTTGAGAATGAATTCAATACTGGAAATATGT | 17781 |
| YJM1388 | 18118 | GGTAGTGTTGTCAGTCCTATTTTATGTAATATTTTTTTAGATAAATTAGATAAATATTTAGAAAATAAATTTGAGAATGAATTCAATACTGGAAATATGT | 18217 |
| YJM789 | 17752 | GGTAGTGTTGTCAGTCCTATTTTATGTAATATTTTTTTAGATAAATTAGATAAATATTTAGAAAATAAATTTGAGAATGAATTCAATACTGGAAATATGT | 17851 |
| YJM1273 | 15173 | GGTAGTGTTGTCAGTCCTATTTTATGTAATATTTTTTTAGATAAATTAGATAAATATTTAGAAAATAAATTTGAGAATGAATTCAATACTGGAAATATGT | 15272 |
| NCYC3585 | 14061 | ---------------------------------------------------------------------------------------------------- | 14060 |
| YJM1401 | 14656 | ---------------------------------------------------------------------------------------------------- | 14655 |
| NCYC3594 | 14433 | GGTAGTGTTGTCAGTCCTATTTTATGTAATATTTTTTTAGATAAATTAGATAAATATTTAGAAAATAAATTTGAGAATGAATTCAATACTGGAAATATGT | 14532 |
| YJM1078 | 16355 | ---------------------------------------------------------------------------------------------------- | 16354 |
| YJM1439 | 17342 | ---------------------------------------------------------------------------------------------------- | 17341 |
| consensus | 22001 | ---------------------------------------------------------------------------------------------------- | 22100 |
|
| S288C | 17782 | CTAATAGAGGTAGAAATCCAATTTATAATAGTTTATCATCTAAAATTTATAGATGTAAATTATTATCTGAAAAATTAAAATTGATTAGATTAAGAGACCA | 17881 |
| YJM1388 | 18218 | CTAATAGAGGTAGAAATCCAATTTATAATAGTTTATCATCTAAAATTTATAGATGTAAATTATTATCTGAAAAATTAAAATTGATTAGATTAAGAGACCA | 18317 |
| YJM789 | 17852 | CTAATAGAGGTAGAAATCCAATTTATAATAGTTTATCATCTAAAATTTATAGATGTAAATTATTATCTGAAAAATTAAAATTGATTAGATTAAGAGACCA | 17951 |
| YJM1273 | 15273 | CTAATAGAGGTAGAAATCCAATTTATAATAGTTTATCATCTAAAATTTATAGATGTAAATTATTATCTGAAAAATTAAAATTGATTAGATTAAGAGACCA | 15372 |
| NCYC3585 | 14061 | ---------------------------------------------------------------------------------------------------- | 14060 |
| YJM1401 | 14656 | ---------------------------------------------------------------------------------------------------- | 14655 |
| NCYC3594 | 14533 | CTAATAGAGGTAGAAATCCAATTTATAATAGTTTATCATCTAAAATTTATAGATGTAAATTATTATCTGAAAAATTAAAATTGATTAGATTAAGAGACCA | 14632 |
| YJM1078 | 16355 | ---------------------------------------------------------------------------------------------------- | 16354 |
| YJM1439 | 17342 | ---------------------------------------------------------------------------------------------------- | 17341 |
| consensus | 22101 | ---------------------------------------------------------------------------------------------------- | 22200 |
|
| S288C | 17882 | TTACCAAAGAAATATGGGATCTGATAAAAGTTTTAAAAGAGCTTATTTTGTTAGATATGCTGATGATATTATCATTGGTGTAATGGGTTCTCATAATGAT | 17981 |
| YJM1388 | 18318 | TTACCAAAGAAATATGGGATCTGATAAAAGTTTTAAAAGAGCTTATTTTGTTAGATATGCTGATGATATTATCATTGGTGTAATGGGTTCTCATAATGAT | 18417 |
| YJM789 | 17952 | TTACCAAAGAAATATGGGATCTGATAAAAGTTTTAAAAGAGCTTATTTTGTTAGATATGCTGATGATATTATCATTGGTGTAATGGGTTCTCATAATGAT | 18051 |
| YJM1273 | 15373 | TTACCAAAGAAATATGGGATCTGATAAAAGTTTTAAAAGAGCTTATTTTGTTAGATATGCTGATGATATTATCATTGGTGTAATGGGTTCTCATAATGAT | 15472 |
| NCYC3585 | 14061 | ---------------------------------------------------------------------------------------------------- | 14060 |
| YJM1401 | 14656 | ---------------------------------------------------------------------------------------------------- | 14655 |
| NCYC3594 | 14633 | TTACCAAAGAAATATGGGATCTGATAAAAGTTTTAAAAGAGCTTATTTTGTTAGATATGCTGATGATATTATCATTGGTGTAATGGGTTCTCATAATGAT | 14732 |
| YJM1078 | 16355 | ---------------------------------------------------------------------------------------------------- | 16354 |
| YJM1439 | 17342 | ---------------------------------------------------------------------------------------------------- | 17341 |
| consensus | 22201 | ---------------------------------------------------------------------------------------------------- | 22300 |
|
| S288C | 17982 | TGTAAAAATATTTTAAACGATATTAATAACTTCTTAAAAGAAAATTTAGGTATGTCAATTAATATAGATAAATCCGTTATTAAACATTCTAAAGAAGGAG | 18081 |
| YJM1388 | 18418 | TGTAAAAATATTTTAAACGATATTAATAACTTCTTAAAAGAAAATTTAGGTATGTCAATTAATATAGATAAATCCGTTATTAAACATTCTAAAGAAGGAG | 18517 |
| YJM789 | 18052 | TGTAAAAATATTTTAAACGATATTAATAACTTCTTAAAAGAAAATTTAGGTATGTCAATTAATATAGATAAATCCGTTATTAAACATTCTAAAGAAGGAG | 18151 |
| YJM1273 | 15473 | TGTAAAAATATTTTAAACGATATTAATAACTTCTTAAAAGAAAATTTAGGTATGTCAATTAATATAGATAAATCCGTTATTAAACATTCTAAAGAAGGAG | 15572 |
| NCYC3585 | 14061 | ---------------------------------------------------------------------------------------------------- | 14060 |
| YJM1401 | 14656 | ---------------------------------------------------------------------------------------------------- | 14655 |
| NCYC3594 | 14733 | TGTAAAAATATTTTAAACGATATTAATAACTTCTTAAAAGAAAATTTAGGTATGTCAATTAATATAGATAAATCCGTTATTAAACATTCTAAAGAAGGAG | 14832 |
| YJM1078 | 16355 | ---------------------------------------------------------------------------------------------------- | 16354 |
| YJM1439 | 17342 | ---------------------------------------------------------------------------------------------------- | 17341 |
| consensus | 22301 | ---------------------------------------------------------------------------------------------------- | 22400 |
|
| S288C | 18082 | TTAGTTTTTTAGGGTATGATGTAAAAGTTACACCTTGAGAAAAAAGACCTTATAGAATGATTAAAAAAGGTGATAATTTTATTAGGGTTAGACATCATAC | 18181 |
| YJM1388 | 18518 | TTAGTTTTTTAGGGTATGATGTAAAAGTTACACCTTGAGAAAAAAGACCTTATAGAATGATTAAAAAAGGTGATAATTTTATTAGGGTTAGACATCATAC | 18617 |
| YJM789 | 18152 | TTAGTTTTTTAGGGTATGATGTAAAAGTTACACCTTGAGAAAAAAGACCTTATAGAATGATTAAAAAAGGTGATAATTTTATTAGGGTTAGACATCATAC | 18251 |
| YJM1273 | 15573 | TTAGTTTTTTAGGGTATGATGTAAAAGTTACACCTTGAGAAAAAAGACCTTATAGAATGATTAAAAAAGGTGATAATTTTATTAGGGTTAGACATCATAC | 15672 |
| NCYC3585 | 14061 | ---------------------------------------------------------------------------------------------------- | 14060 |
| YJM1401 | 14656 | ---------------------------------------------------------------------------------------------------- | 14655 |
| NCYC3594 | 14833 | TTAGTTTTTTAGGGTATGATGTAAAAGTTACACCTTGAGAAAAAAGACCTTATAGAATGATTAAAAAAGGTGATAATTTTATTAGGGTTAGACATCATAC | 14932 |
| YJM1078 | 16355 | ---------------------------------------------------------------------------------------------------- | 16354 |
| YJM1439 | 17342 | ---------------------------------------------------------------------------------------------------- | 17341 |
| consensus | 22401 | ---------------------------------------------------------------------------------------------------- | 22500 |
|
| S288C | 18182 | TAGTTTAGTTGTTAATGCCCCTATTAGAAGTATTGTAATAAAATTAAATAAACATGGCTATTGTTCTCATGGTATTTTAGGAAAACCCAGAGGGGTTGGA | 18281 |
| YJM1388 | 18618 | TAGTTTAGTTGTTAATGCCCCTATTAGAAGTATTGTAATAAAATTAAATAAACATGGCTATTGTTCTCATGGTATTTTAGGAAAACCCAGAGGGGTTGGA | 18717 |
| YJM789 | 18252 | TAGTTTAGTTGTTAATGCCCCTATTAGAAGTATTGTAATAAAATTAAATAAACATGGCTATTGTTCTCATGGTATTTTAGGAAAACCCAGAGGGGTTGGA | 18351 |
| YJM1273 | 15673 | TAGTTTAGTTGTTAATGCCCCTATTAGAAGTATTGTAATAAAATTAAATAAACATGGCTATTGTTCTCATGGTATTTTAGGAAAACCCAGAGGGGTTGGA | 15772 |
| NCYC3585 | 14061 | ---------------------------------------------------------------------------------------------------- | 14060 |
| YJM1401 | 14656 | ---------------------------------------------------------------------------------------------------- | 14655 |
| NCYC3594 | 14933 | TAGTTTAGTTGTTAATGCCCCTATTAGAAGTATTGTAATAAAATTAAATAAACATGGCTATTGTTCTCATGGTATTTTAGGAAAACCCAGAGGGGTTGGA | 15032 |
| YJM1078 | 16355 | ---------------------------------------------------------------------------------------------------- | 16354 |
| YJM1439 | 17342 | ---------------------------------------------------------------------------------------------------- | 17341 |
| consensus | 22501 | ---------------------------------------------------------------------------------------------------- | 22600 |
|
| S288C | 18282 | AGATTAATTCATGAAGAAATGAAAACCATTTTAATGCATTACTTAGCTGTTGGTAGAGGTATTATAAACTATTATAGATTAGCTACCAATTTTACCACAT | 18381 |
| YJM1388 | 18718 | AGATTAATTCATGAAGAAATGAAAACCATTTTAATGCATTACTTAGCTGTTGGTAGAGGTATTATAAACTATTATAGATTAGCTACCAATTTTACCACAT | 18817 |
| YJM789 | 18352 | AGATTAATTCATGAAGAAATGAAAACCATTTTAATGCATTACTTAGCTGTTGGTAGAGGTATTATAAACTATTATAGATTAGCTACCAATTTTACCACAT | 18451 |
| YJM1273 | 15773 | AGATTAATTCATGAAGAAATGAAAACCATTTTAATGCATTACTTAGCTGTTGGTAGAGGTATTATAAACTATTATAGATTAGCTACCAATTTTACCACAT | 15872 |
| NCYC3585 | 14061 | ---------------------------------------------------------------------------------------------------- | 14060 |
| YJM1401 | 14656 | ---------------------------------------------------------------------------------------------------- | 14655 |
| NCYC3594 | 15033 | AGATTAATTCATGAAGAAATGAAAACCATTTTAATGCATTACTTAGCTGTTGGTAGAGGTATTATAAACTATTATAGATTAGCTACCAATTTTACCACAT | 15132 |
| YJM1078 | 16355 | ---------------------------------------------------------------------------------------------------- | 16354 |
| YJM1439 | 17342 | ---------------------------------------------------------------------------------------------------- | 17341 |
| consensus | 22601 | ---------------------------------------------------------------------------------------------------- | 22700 |
|
| S288C | 18382 | TAAGAGGTAGAATTACATACATTTTATTTTATTCATGTTGTTTAACATTAGCAAGAAAATTTAAATTAAATACTGTTAAGAAAGTTATTTTAAAATTCGG | 18481 |
| YJM1388 | 18818 | TAAGAGGTAGAATTACATACATTTTATTTTATTCATGTTGTTTAACATTAGCAAGAAAATTTAAATTAAATACTGTTAAGAAAGTTATTTTAAAATTCGG | 18917 |
| YJM789 | 18452 | TAAGAGGTAGAATTACATACATTTTATTTTATTCATGTTGTTTAACATTAGCAAGAAAATTTAAATTAAATACTGTTAAGAAAGTTATTTTAAAATTCGG | 18551 |
| YJM1273 | 15873 | TAAGAGGTAGAATTACATACATTTTATTTTATTCATGTTGTTTAACATTAGCAAGAAAATTTAAATTAAATACTGTTAAGAAAGTTATTTTAAAATTCGG | 15972 |
| NCYC3585 | 14061 | ---------------------------------------------------------------------------------------------------- | 14060 |
| YJM1401 | 14656 | ---------------------------------------------------------------------------------------------------- | 14655 |
| NCYC3594 | 15133 | TAAGAGGTAGAATTACATACATTTTATTTTATTCATGTTGTTTAACATTAGCAAGAAAATTTAAATTAAATACTGTTAAGAAAGTTATTTTAAAATTCGG | 15232 |
| YJM1078 | 16355 | ---------------------------------------------------------------------------------------------------- | 16354 |
| YJM1439 | 17342 | ---------------------------------------------------------------------------------------------------- | 17341 |
| consensus | 22701 | ---------------------------------------------------------------------------------------------------- | 22800 |
|
| S288C | 18482 | TAAAGTATTAGTTGATCCTCATTCAAAAGTTAGTTTTAGTATTGATGATTTTAAAATTAGACATAAAATAAATATAACTGATTCTAATTATACACCTGAT | 18581 |
| YJM1388 | 18918 | TAAAGTATTAGTTGATCCTCATTCAAAAGTTAGTTTTAGTATTGATGATTTTAAAATTAGACATAAAATAAATATAACTGATTCTAATTATACACCTGAT | 19017 |
| YJM789 | 18552 | TAAAGTATTAGTTGATCCTCATTCAAAAGTTAGTTTTAGTATTGATGATTTTAAAATTAGACATAAAATAAATATAACTGATTCTAATTATACACCTGAT | 18651 |
| YJM1273 | 15973 | TAAAGTATTAGTTGATCCTCATTCAAAAGTTAGTTTTAGTATTGATGATTTTAAAATTAGACATAAAATAAATATAACTGATTCTAATTATACACCTGAT | 16072 |
| NCYC3585 | 14061 | ---------------------------------------------------------------------------------------------------- | 14060 |
| YJM1401 | 14656 | ---------------------------------------------------------------------------------------------------- | 14655 |
| NCYC3594 | 15233 | TAAAGTATTAGTTGATCCTCATTCAAAAGTTAGTTTTAGTATTGATGATTTTAAAATTAGACATAAAATAAATATAACTGATTCTAATTATACACCTGAT | 15332 |
| YJM1078 | 16355 | ---------------------------------------------------------------------------------------------------- | 16354 |
| YJM1439 | 17342 | ---------------------------------------------------------------------------------------------------- | 17341 |
| consensus | 22801 | ---------------------------------------------------------------------------------------------------- | 22900 |
|
| S288C | 18582 | GAAATTTTAGATAGATATAAATATATGTTACCTAGATCTTTATCATTATTTAGTGGTATTTGTCAAATTTGTGGTTCTAAACATGATTTAGAAGTACATC | 18681 |
| YJM1388 | 19018 | GAAATTTTAGATAGATATAAATATATGTTACCTAGATCTTTATCATTATTTAGTGGTATTTGTCAAATTTGTGGTTCTAAACATGATTTAGAAGTACATC | 19117 |
| YJM789 | 18652 | GAAATTTTAGATAGATATAAATATATGTTACCTAGATCTTTATCATTATTTAGTGGTATTTGTCAAATTTGTGGTTCTAAACATGATTTAGAAGTACATC | 18751 |
| YJM1273 | 16073 | GAAATTTTAGATAGATATAAATATATGTTACCTAGATCTTTATCATTATTTAGTGGTATTTGTCAAATTTGTGGTTCTAAACATGATTTAGAAGTACATC | 16172 |
| NCYC3585 | 14061 | ---------------------------------------------------------------------------------------------------- | 14060 |
| YJM1401 | 14656 | ---------------------------------------------------------------------------------------------------- | 14655 |
| NCYC3594 | 15333 | GAAATTTTAGATAGATATAAATATATGTTACCTAGATCTTTATCATTATTTAGTGGTATTTGTCAAATTTGTGGTTCTAAACATGATTTAGAAGTACATC | 15432 |
| YJM1078 | 16355 | ---------------------------------------------------------------------------------------------------- | 16354 |
| YJM1439 | 17342 | ---------------------------------------------------------------------------------------------------- | 17341 |
| consensus | 22901 | ---------------------------------------------------------------------------------------------------- | 23000 |
|
| S288C | 18682 | ACGTAAGAACATTAAATAATGCTGCCAATAAAATTAAAGATGATTATTTATTAGGTAGAATGATTAAGATAAATAGAAAACAAATTACTATCTGTAAAAC | 18781 |
| YJM1388 | 19118 | ACGTAAGAACATTAAATAATGCTGCCAATAAAATTAAAGATGATTATTTATTAGGTAGAATGATTAAGATAAATAGAAAACAAATTACTATCTGTAAAAC | 19217 |
| YJM789 | 18752 | ACGTAAGAACATTAAATAATGCTGCCAATAAAATTAAAGATGATTATTTATTAGGTAGAATGATTAAGATAAATAGAAAACAAATTACTATCTGTAAAAC | 18851 |
| YJM1273 | 16173 | ACGTAAGAACATTAAATAATGCTGCCAATAAAATTAAAGATGATTATTTATTAGGTAGAATGATTAAGATAAATAGAAAACAAATTACTATCTGTAAAAC | 16272 |
| NCYC3585 | 14061 | ---------------------------------------------------------------------------------------------------- | 14060 |
| YJM1401 | 14656 | ---------------------------------------------------------------------------------------------------- | 14655 |
| NCYC3594 | 15433 | ACGTAAGAACATTAAATAATGCTGCCAATAAAATTAAAGATGATTATTTATTAGGTAGAATGATTAAGATAAATAGAAAACAAATTACTATCTGTAAAAC | 15532 |
| YJM1078 | 16355 | ---------------------------------------------------------------------------------------------------- | 16354 |
| YJM1439 | 17342 | ---------------------------------------------------------------------------------------------------- | 17341 |
| consensus | 23001 | ---------------------------------------------------------------------------------------------------- | 23100 |
|
| S288C | 18782 | ATGTCATTTTAAAGTTCATCAAGGTAAATATAATGGTCCAGGTTTATAATAATTATTATACTATTAAATATGCGTTAAATGGAGAGCCGTATGATATGAA | 18881 |
| YJM1388 | 19218 | ATGTCATTTTAAAGTTCATCAAGGTAAATATAATGGTCCAGGTTTATAATAATTATTATACTATTAAATATGCGTTAAATGGAGAGCCGTATGATATGAA | 19317 |
| YJM789 | 18852 | ATGTCATTTTAAAGTTCATCAAGGTAAATATAATGGTCCAGGTTTATAATAATTATTATACTATTAAATATGCGTTAAATGGAGAGCCGTATGATATGAA | 18951 |
| YJM1273 | 16273 | ATGTCATTTTAAAGTTCATCAAGGTAAATATAATGGTCCAGGTTTATAATAATTATTATACTATTAAATATGCGTTAAATGGAGAGCCGTATGATATGAA | 16372 |
| NCYC3585 | 14061 | ---------------------------------------------------------------------------------------------------- | 14060 |
| YJM1401 | 14656 | ---------------------------------------------------------------------------------------------------- | 14655 |
| NCYC3594 | 15533 | ATGTCATTTTAAAGTTCATCAAGGTAAATATAATGGTCCAGGTTTATAATAATTATTATACTATTAAATATGCGTTAAATGGAGAGCCGTATGATATGAA | 15632 |
| YJM1078 | 16355 | ---------------------------------------------------------------------------------------------------- | 16354 |
| YJM1439 | 17342 | ---------------------------------------------------------------------------------------------------- | 17341 |
| consensus | 23101 | ---------------------------------------------------------------------------------------------------- | 23200 |
|
| S288C | 18882 | AGTATCACGTACGGTTCGGAGAGGGCTCTTTTATATGAATGTTATTACATTCAGATAGGTTTGCTACTCTACTCTTAGTAATGCCTGCTTTAATTGGAGG | 18981 |
| YJM1388 | 19318 | AGTATCACGTACGGTTCGGAGAGGGCTCTTTTATATGAATGTTATTACATTCAGATAGGTTTGCTACTCTACTCTTAGTAATGCCTGCTTTAATTGGTGG | 19417 |
| YJM789 | 18952 | AGTATCACGTACGGTTCGGAGAGGGCTCTTTTATATGAATGTTATTACATTCAGATAGGTTTGCTACTCTACTCTTAGTAATGCCTGCTTTAATTGGAGG | 19051 |
| YJM1273 | 16373 | AGTATCACGTACGGTTCGGAGAGGGCTCTTTTATATGAATGTTATTACATTCAGATAGGTTTGCTACTCTACTCTTAGTAATGCCTGCTTTAATTGGTGG | 16472 |
| NCYC3585 | 14061 | ------------------------------------------------------------------------TCTTAGTAATGCCTGCTTTAATTGGTGG | 14088 |
| YJM1401 | 14656 | ------------------------------------------------------------------------TCTTAGTAATGCCTGCTTTAATTGGAGG | 14683 |
| NCYC3594 | 15633 | AGTATCACGTACGGTTCGGAGAGGGCTCTTTTATATGAATGTTATTACATTCAGATAGGTTTGCTACTCTACTCTTAGTAATGCCTGCTTTAATTGGTGG | 15732 |
| YJM1078 | 16355 | ------------------------------------------------------------------------TCTTAGTAATGCCTGCTTTAATTGGAGG | 16382 |
| YJM1439 | 17342 | ------------------------------------------------------------------------TCTTAGTAATGCCTGCTTTAATAGGAGG | 17369 |
| consensus | 23201 | ------------------------------------------------------------------------\*\*\*\*\*\*\*\*\*\*\*\*\*\*\*\*\*\*\*\*\*\*.\*\*.\*\* | 23300 |
|
| S288C | 18982 | TTTTGGTAACCAAAAAAGATATGAAAGTAATAATAATAA---TAATCAAGTAATAGAAAATAAAGAATA---------TAATTTAAAATTAAATTATGAT | 19069 |
| YJM1388 | 19418 | ATTTGGTAAAGATAAAA-ATATGAAAG---TAATAATAA---TAATCAAATAATAAAAAATAAATAATATAATAGTTTTAATCTAAAATAAAATTATGAT | 19510 |
| YJM789 | 19052 | TTTTGGTAACCAAAAAAGATATGAAAGTAATAATAATAA---TAATCAAGTAATAGAAAATAAAGAATA---------TAATTTAAAATTAAATTATGAT | 19139 |
| YJM1273 | 16473 | ATTTGGTAAC------------------------------------------------------------------------------------------ | 16482 |
| NCYC3585 | 14089 | ATTTGGTAAAGATAAAA-ATATGAAAG---TAATAATAA---TAATCAAATAATAAAAAATAAATAATATAATAGTTTTAATCTAAAATAAAATTATGAT | 14181 |
| YJM1401 | 14684 | TTTTGGTAACCAAAAAAGATATGAAAGTAATAATAATAA---TAATCAAGTAATAGAAAATAAAGAATA---------TAATTTAAAATTAAATTATGAT | 14771 |
| NCYC3594 | 15733 | ATTTGGTAAAGATAAAA-ATATGAAAGT---AATAATAA---TAATCAAATAATAAAAAATAAATAATATAATAGTTTTAATCTAAAATAAAATTATGAT | 15825 |
| YJM1078 | 16383 | TTTTGGTAACCAAAAAAGATATGAAAGT---AATAATAATAATAATCAAGTAATAGAAAATAAAGAATA---------TAATTTAAAATTAAATTATGAT | 16470 |
| YJM1439 | 17370 | TTTTGGTAACCAAAAAAGATATGAAAGTAATAATAATAA---TAATCAAGTAATAGAAAATAAAGAATA---------TAATTTAAAATTAAATTATGAT | 17457 |
| consensus | 23301 | .\*\*\*\*\*\*\*\*..-.------------------------------------.-----.--------.-----------------.------.---------- | 23400 |
|
| S288C | 19070 | AAGTTGGGACCTTATTTAGCTGGATTAATTGAAGGTGATGGAACTATTCTAGTTCAAAATTCATCTTCAATAA--AAAAATCT-----AAATATAGACCG | 19162 |
| YJM1388 | 19511 | AATTTAGAATCTTATTTAGTTGAATTAATTGAAGGTGATGGAACTATTCTAGTTCAAGATTCATTAACAAAAAGTAAACAAAA-----AAATATAGATCT | 19605 |
| YJM789 | 19140 | AAGTTGGGACCTTATTTAGCTGGATTAATTGAAGGTGATGGAACTATTCTAGTTCAAAATTCA---TCTTCAA--TAA--AAAAATCTAAATATAGACCG | 19232 |
| YJM1273 | 16483 | ---------------------------------------------------------------------------------------------------- | 16482 |
| NCYC3585 | 14182 | AATTTAGAATCTTATTTAGTTGAATTAATTGAAGGTGATGGAACTATTCTAGTTCAAGATTCA---TTAACAA--AAAGTAAACAAAAAAATATAGATCT | 14276 |
| YJM1401 | 14772 | AAGTTGGGACCTTATTTAGCTGGATTAATTGAAGGTGATGGAACTATTCTAGTTCAAAATTCA---TCTTCAA--TAA--AAAAATCTAAATATAGACCG | 14864 |
| NCYC3594 | 15826 | AATTTAGAATCTTATTTAGTTGAATTAATTGAAGGTGATGGAACTATTCTAGTTCAAGATTCA---TTAACAA--AAAGTAAACAAAAAAATATAGATCT | 15920 |
| YJM1078 | 16471 | AAGTTGGGACCTTATTTAGCTGGATTAATTGAAGGTGATGGAACTATTCTAGTTCAAAATTCA---TCTTCAA--TAA--AAAAATCTAAATATAGACCG | 16563 |
| YJM1439 | 17458 | AAGTTGGGACCTTATTTAGCTGGATTAATTGAAGGTGATGGAACTATTCTAGTTCAAAATTCA---TCTTCAA--TAA--AAAAATCTAAATATAGACCG | 17550 |
| consensus | 23401 | --.--.-.-.---------.--.----------------------------------.------.......----.--......-...---------.-. | 23500 |
|
| S288C | 19163 | TTAATTGTTGTAGTATTTAAATTAGAAGATTTAGAATTAGCTAATTATTTATGTAATTTAACTAAATGTGGAAAAGTGTATAAAAAAATTAATCGTAATT | 19262 |
| YJM1388 | 19606 | ATAATTATTCTAATATTAAAATTATAATATTTTTAATTAGCTAATTATTTATATAATTTAATTAAATGTGGTAAAGTATAT-AAAAAATTAATTGTAATT | 19704 |
| YJM789 | 19233 | TTAATTGTTGTAGTATTTAAATTAGAAGATTTAGAATTAGCTAATTATTTATGTAATTTAACTAAATGTGGGAAAGTGTATAAAAAAATTAATCGTAATT | 19332 |
| YJM1273 | 16483 | ---------------------------------------------------------------------------------------------------- | 16482 |
| NCYC3585 | 14277 | ATAATTATTCTAATATTAAAATTATAATATTTTTAATTAGCTAATTATTTATATAATTTAATTAAATGTGGTAAAGTATAT-AAAAAATTAATTGTAATT | 14375 |
| YJM1401 | 14865 | TTAATTGTTGTAGTATTTAAATTAGAAGATTTAGAATTAGCTAATTATTTATGTAATTTAACTAAATGTGGAAAAGTGTATAAAAAAATTAATCGTAATT | 14964 |
| NCYC3594 | 15921 | ATAATTATTCTAATATTAAAATTATAATATTTTTAATTAGCTAATTATTTATATAATTTAATTAAATGTGGTAAAGTATAT-AAAAAATTAATTGTAATT | 16019 |
| YJM1078 | 16564 | TTAATTGTTGTAGTATTTAAATTAGAAGATTTAGAATTAGCTAATTATTTATGTAATTTAACTAAATGTGGGAAAGTGTATAAAAAAATTAATCGTAATT | 16663 |
| YJM1439 | 17551 | TTAATTGTTGTAGTATTTAAATTAGAAGATTTAGAATTAGCTAATTATTTATGTAATTTAACTAAATGTGG-AAAGTGTATAAAAAAATTAATCGTAATT | 17649 |
| consensus | 23501 | .-----.--.--.----.------.--.----..------------------.--------.---------.-----.---------------.------ | 23600 |
|
| S288C | 19263 | ATGTATTATGACTTATTCATGATTTAAAAGGTGTATATACATTATTAAATATTATTAATGGATATATGAGAACACCTAAATATGAAGCATTTGTTAGAGG | 19362 |
| YJM1388 | 19705 | ATGTATTATGACTTATTTATGATTTAAAAGGTGTATATACATTATTAAATATTATTAATGGATATATAAAAACACCTAAATATGAAACATTTGTAAGAAG | 19804 |
| YJM789 | 19333 | ATGTATTATGACTTATTCATGATTTAAAAGGTGTATATACATTATTAAATATTATTAATGGATATATGAGAACACCTAAATATGAAGCATTTGTTAGAGG | 19432 |
| YJM1273 | 16483 | ---------------------------------------------------------------------------------------------------- | 16482 |
| NCYC3585 | 14376 | ATGTATTATGACTTATTTATGATTTAAAAGGTGTATATACATTATTAAATATTATTAATGGATATATAAAAACACCTAAATATGAAACATTTGTAAGAAG | 14475 |
| YJM1401 | 14965 | ATGTATTATGACTTATTCATGATTTAAAAGGTGTATATACATTATTAAATATTATTAATGGATATATGAGAACACCTAAATATGAAGCATTTGTTAGAGG | 15064 |
| NCYC3594 | 16020 | ATGTATTATGACTTATTTATGATTTAAAAGGTGTATATACATTATTAAATATTATTAATGGATATATAAAAACACCTAAATATGAAACATTTGTAAGAAG | 16119 |
| YJM1078 | 16664 | ATGTATTATGACTTATTCATGATTTAAAAGGTGTATATACATTATTAAATATTATTAATGGATATATGAGAACACCTAAATATGAAGCATTTGTTAGAGG | 16763 |
| YJM1439 | 17650 | ATGTATTATGACTTATTCATGATTTAAAAGGTGTATATACATTATTAAATATTATTAATGGATATATGAGAACACCTAAATATGAAACATTTGTTAGAGG | 17749 |
| consensus | 23601 | -----------------.-------------------------------------------------.-.----------------.-------.---.- | 23700 |
|
| S288C | 19363 | TGCTGAATTTATAAATAATTATATTAATTCAAC---------------------------------------------------------------AACA | 19399 |
| YJM1388 | 19805 | TGTTTAATTTATAAATAATTATATTAATTCTCCTTTCGGGGTTCCGGCTCCCGTGGCCGGAACTCCGTAACTATAAAAATTCATCTATCTTATGGGATGA | 19904 |
| YJM789 | 19433 | TGCTGAATTTATAAATAATTATATTAATT-----------------------------------------CAACAACAATT------------------- | 19472 |
| YJM1273 | 16483 | ---------------------------------------------------------------------------------------------------- | 16482 |
| NCYC3585 | 14476 | TGTTTAATTTATAAATAATTATATTAATTCTC---------------------------------CGTAACTATAAAAATTCATCTATCTTATGGGATGA | 14542 |
| YJM1401 | 15065 | TGCTGAATTTATAAATAATTATATTAATT-----------------------------------------CAACAACAATT------------------- | 15104 |
| NCYC3594 | 16120 | TGTTTAATTTATAAATAATTATATTAATTCTCCTTTCGGGGTTCCGGCTCCCGTGGCCGGAACTCCGTAACTATAAAAATTCATCTATCTTATGGGATGA | 16219 |
| YJM1078 | 16764 | TGCTGAATTTATAAATAATTATATTAATTCAAC---------------------------------------------------------------AACA | 16800 |
| YJM1439 | 17750 | TGCTGAATTTATAAATAATTATATTAATTCAAC---------------------------------------------------------------AACA | 17786 |
| consensus | 23701 | --.-.-------------------------..---------------------------------------.-.--.--------------------..- | 23800 |
|
| S288C | 19400 | ATTCTACATAATAAATTAAAAAATATAGATAATATTAAAATTAAACCATTAGATACATCAGATATTGGTTCAAACGCTTGATTAGCTGGTATGACAGATG | 19499 |
| YJM1388 | 19905 | AGACTAAATAATAAATAAAAAAATATAAATAATATTAAAATTAAACCATTAGATATATTAGATATTGGTTCAAACGCTTAATTAGATGGTATGACTGATG | 20004 |
| YJM789 | 19473 | ---CTACATAATAAATTAAAAAATATAGATAATATTAAAATTAAACCATTAGATACATCAGATATTGGTTCAAACGCTTGATTAGCTGGTATGACAGATG | 19569 |
| YJM1273 | 16483 | ---------------------------------------------------------------------------------------------------- | 16482 |
| NCYC3585 | 14543 | AGACTAAATAATAAATAAAAAAATATAAATAATATTAAAATTAAACCATTAGATATATTAGATATTGGTTCAAACGCTTAATTAGATGGTATGACTGATG | 14642 |
| YJM1401 | 15105 | ---CTACATAATAAATTAAAAAATATAGATAATATTAAAATTAAACCATTAGATACATCAGATATTGGTTCAAACGCTTGATTAGCTGGTATGACAGATG | 15201 |
| NCYC3594 | 16220 | AGACTAAATAATAAATAAAAAAATATAAATAATATTAAAATTAAACCATTAGATATATTAGATATTGGTTCAAACGCTTAATTAGATGGTATGACTGATG | 16319 |
| YJM1078 | 16801 | ATTCTACATAATAAATTAAAAAATATAGATAATATTAAAATTAAACCATTAGATACATCAGATATTGGTTCAAACGCTTGATTAGCTGGTATGACAGATG | 16900 |
| YJM1439 | 17787 | ATTCTACATAATAAATTAAAAAATATAGATAATATTAAAATTAAACCATTAGATACATTAGATATTGGTTCAAACGCTTGATTAGCTGGTATGACAGATG | 17886 |
| consensus | 23801 | -..---.---------.----------.---------------------------.--.--------------------.-----.---------.---- | 23900 |
|
| S288C | 19500 | CAGATGGTAA-TTTTTCTATTAATTTAATAAATGGTAAAAATCGTTCTAGTAGAGCAATGCCTTATTATTGTTTAGAATTAAGAC--AAAATTATCAAAA | 19596 |
| YJM1388 | 20005 | CGGATGGTAATTTTTTCTATTAATTTAATAAATAGT-AAAATCGTTCTAATAAAGCAATGCCTTATTATTGTTTATAATTAAAAC--TTATTATTACTAA | 20101 |
| YJM789 | 19570 | CAGATGGTAA-TTTTTCTATTAATTTAATAAATGGTAAAAATCGTTCTAGTAGAGCAATGCCTTATTATTGTTTAGAATTAAGAC--AAAATTATCAAAA | 19666 |
| YJM1273 | 16483 | ---------------------------------------------------------------------------------------------------- | 16482 |
| NCYC3585 | 14643 | CGGATGGTAA-TTTTTCTATTAATTTAATAAATAGT-AAAATCGTTCTAATAAAGCAATGCCTTATTATTGTTTATAATTAAAACTTATTATTACTAAA- | 14739 |
| YJM1401 | 15202 | CAGATGGTAA-TTTTTCTATTAATTTAATAAATGGTAAAAATCGTTCTAGTAGAGCAATGCCTTATTATTGTTTAGAATTAAGAC--AAAATTATCAAAA | 15298 |
| NCYC3594 | 16320 | CGGATGGTAA-TTTTTCTATTAATTTAATAAATAGT-AAAATCGTTCTAATAAAGCAATGCCTTATTATTGTTTATAATTAAAAC---TTATTATTACTA | 16414 |
| YJM1078 | 16901 | CAGATGGTAA-TTTTTCTATTAATTTAATAAATGGTAAAAATCGTTCTAGTAGAGCAATGCCTTATTATTGTTTAGAATTAAGAC---AAAATTATCAAA | 16996 |
| YJM1439 | 17887 | CAGATGGTAA-TTTTTCTATTAATTTAATAAATGGTAAAAATCGTTCTAGTAGAGCAATGCCTTATTATTGTTTAGAATTAAGAC--AAAATAATCATAA | 17983 |
| consensus | 23901 | -.-------------------------------.---------------.--.----------------------.------.----............- | 24000 |
|
| S288C | 19597 | AA------ATTCTAATAATAATAATATTAATTTTTCTTATTTTTATATTATGTCTGCAATTGCACTATATTTTAATGTTAATTTATATAGTAGAGAACGT | 19690 |
| YJM1388 | 20102 | A------------AATAATAATAATATTAATTTATCATATTTTAATATTATGTTTATAATTGCACTATATTTTAATGTTAATTTATATAATAGAGAACGT | 20189 |
| YJM789 | 19667 | AA------ATTCTAATAATAATAATATTAATTTTTCTTATTTTTATATTATGTCTGCAATTGCACTATATTTTAATGTTAATTTATATAGTAGAGAACGT | 19760 |
| YJM1273 | 16483 | ---------------------------------------------------------------------------------------------------- | 16482 |
| NCYC3585 | 14740 | -------------AATAATAATAATATTAATTTATCATATTTTAATATTATGTTTATAATTGCACTATATTTTAATGTTAATTTATATAATAGAGAACGT | 14826 |
| YJM1401 | 15299 | AA------ATTCTAATAATAATAATATTAATTTTTCTTATTTTTATATTATGTCTGCAATTGCACTATATTTTAATGTTAATTTATATAGTAGAGAACGT | 15392 |
| NCYC3594 | 16415 | AA------A-----ATAATAATAATATTAATTTATCATATTTTAATATTATGTTTATAATTGCACTATATTTTAATGTTAATTTATATAATAGAGAACGT | 16503 |
| YJM1078 | 16997 | AAAATTCTA-----ATAATAATAATATTAATTTTTCTTATTTTTATATTATGTCTGCAATTGCACTATATTTTAATGTTAATTTATATAGTAGAGAACGT | 17091 |
| YJM1439 | 17984 | AA------ATTCTAATAATAATAATATTAATTTTTCTTATTTTTATATTATGTCTGCAATTGCACTATATTTTAATGTTAATTTATATAGTAGAGAACGT | 18077 |
| consensus | 24001 | ---------------------------------.--.------.---------.-..--------------------------------.---------- | 24100 |
|
| S288C | 19691 | AATTTAAATTTATTAGTATCTCTTAATAA-TACGTATAAACTATATTATAGTTATAAAGTAATAGTGGCTAATCTATATAAAAATATTAAAGTAATAGAA | 19789 |
| YJM1388 | 20190 | AATTTAAATTTACAATCTTCTCTAAAAAA-GTACATATAACTATATTATAGTTATAAAGTAATATTAGCTAATCTATATAAAAATATTAAAGTTATAAAA | 20288 |
| YJM789 | 19761 | AATTTAAATTTATTAGTATCTCTTAATAA-TACGTATAAACTATATTATAGTTATAAAGTAATAGTGGCTAATCTATATAAAAATATTAAAGTAATAGAA | 19859 |
| YJM1273 | 16483 | ---------------------------------------------------------------------------------------------------- | 16482 |
| NCYC3585 | 14827 | AATTTAAATTTACAATCTTCTCTAAAAAAGTACATAT-AACTATATTATAGTTATAAAGTAATATTAGCTAATCTATATAAAAATATTAAAGTTATAAAA | 14925 |
| YJM1401 | 15393 | AATTTAAATTTATTAGTATCTCTTAATAA-TACGTATAAACTATATTATAGTTATAAAGTAATAGTGGCTAATCTATATAAAAATATTAAAGTAATAGAA | 15491 |
| NCYC3594 | 16504 | AATTTAAATTTACAATCTTCTCTAAAAAAGTACATAT-AACTATATTATAGTTATAAAGTAATATTAGCTAATCTATATAAAAATATTAAAGTTATAAAA | 16602 |
| YJM1078 | 17092 | AATTTAAATTTATTAGTATCTCTTAATAATACGTATA-AACTATATTATAGTTATAAAGTAATAGTGGCTAATCTATATAAAAATATTAAAGTAATAGAA | 17190 |
| YJM1439 | 18078 | AATTTAAATTTATTAGTATCTCTTAATAA-TACGTATAAACTATATTATAGTTATAAAGTAATAGTGGCTAATCTATATAAAAATATTAAAGTAATAGAA | 18176 |
| consensus | 24101 | ------------..-...-----.--.--.........--------------------------.-.--------------------------.---.-- | 24200 |
|
| S288C | 19790 | TACTTTAATAAATATTCTTTATTATCATCTAAACACTTAGATTTTTTAGATTGATCTAAATTAGTTATTTTAATTAATAATGAGGGTCAAAGTAT-AAAA | 19888 |
| YJM1388 | 20289 | TACTTTAATAAATATTTTTTATTATCATCTAAACATTTAGATTATTTATATTGATTTAGATTAGTTATTTTAATTTATAATGAAGGTCAAAGTAT--AAA | 20386 |
| YJM789 | 19860 | TACTTTAATAAATATTCTTTATTATCATCTAAACACTTAGATTTTTTAGATTGATCTAAATTAGTTATTTTAATTAATAATGAGGGTCAAAGTAT-AAAA | 19958 |
| YJM1273 | 16483 | ---------------------------------------------------------------------------------------------------- | 16482 |
| NCYC3585 | 14926 | TACTTTAATAAATATTTTTTATTATCATCTAAACATTTAGATTATTTATATTGATTTAGATTAGTTATTTTAATTTATAATGAAGGTCAAAGTAT--AAA | 15023 |
| YJM1401 | 15492 | TACTTTAATAAATATTCTTTATTATCATCTAAACACTTAGATTTTTTAGATTGATCTAAATTAGTTATTTTAATTAATAATGAGGGTCAAAGTAT-AAAA | 15590 |
| NCYC3594 | 16603 | TACTTTAATAAATATTTTTTATTATCATCTAAACATTTAGATTATTTATATTGATTTAGATTAGTTATTTTAATTTATAATGAAGGTCAAAGTAT-AA-A | 16700 |
| YJM1078 | 17191 | TACTTTAATAAATATTCTTTATTATCATCTAAACACTTAGATTTTTTAGATTGATCTAAATTAGTTATTTTAATTAATAATGAGGGTCAAAGTATAAA-A | 17289 |
| YJM1439 | 18177 | TACTTTAATAAATATTCTTTATTATCATCTAAACACTTAGATTTTTTAGATTGATCTAAATTAGTTATTTTAATTAATAATGAGGGTCAAAGTAT-AAAA | 18275 |
| consensus | 24201 | ----------------.------------------.-------.----.------.--.----------------.-------.---------------- | 24300 |
|
| S288C | 19889 | CTTAATGGTAGTTGAGAATTAGGTATAAATTTACGTAAAGATTATAATAAAACTAGAACTACGTTTACTTGATCTCATTTAAAAAATACATATTTAGAAA | 19988 |
| YJM1388 | 20387 | CTTAATGGTAGTTAAGAATTAGGTCTAAATTTACGTAAAAATTATAATAAAACTATAACTACATTTAATTAATTAGATAAAAAAAATACATATTTATAAA | 20486 |
| YJM789 | 19959 | CTTAATGGTAGTTGAGAATTAGGTATAAATTTACGTAAAGATTATAATAAAACTAGAACTACGTTTACTTGATCTCATTTAAAAAATACATATTTAGAAA | 20058 |
| YJM1273 | 16483 | ---------------------------------------------------------------------------------------------------- | 16482 |
| NCYC3585 | 15024 | CTTAATGGTAGTTAAGAATTAGGTCTAAATTTACGTAAAAATTATAATAAAACTATAACTACATTTAATTAATTAGATAAAAAAAATACATATTTATAAA | 15123 |
| YJM1401 | 15591 | CTTAATGGTAGTTGAGAATTAGGTATAAATTTACGTAAAGATTATAATAAAACTAGAACTACGTTTACTTGATCTCATTTAAAAAATACATATTTAGAAA | 15690 |
| NCYC3594 | 16701 | CTTAATGGTAGTTAAGAATTAGGTCTAAATTTACGTAAAAATTATAATAAAACTATAACTACATTTAATTAATTAGATAAAAAAAATACATATTTATAAA | 16800 |
| YJM1078 | 17290 | CTTAATGGTAGTTGAGAATTAGGTATAAATTTACGTAAAGATTATAATAAAACTAGAACTACGTTTACTTGATCTCATTTAAAAAATACATATTTAGAAA | 17389 |
| YJM1439 | 18276 | CTTAATGGTAGTTGAGAATTAGGTATAAATTTACGTAAAGATTATAATAAAACTAGAACTACGTTTACTTGATCTCATTAAAAAAAAACATATTTAGAAA | 18375 |
| consensus | 24301 | -------------.----------.--------------.---------------.------.----.--.--...--..------.---------.--- | 24400 |
|
| S288C | 19989 | ATAAAT-AAATAA-----------------ATTATTATTAC-----------------TTTC-TTCCCCTCCGAATCCG----T----AATATATTTACG | 20044 |
| YJM1388 | 20487 | ATAAAT-AATATATTTTCTATTTTATGGTTTTTATTATTAC-----------------TTT--TTCTCCACCAAACTTGTTAAT----AATATAATAACA | 20562 |
| YJM789 | 20059 | ATAAATAAATAAA-----------------------------------TTATTATTACTTTC-TTCCCCTCCGAATCCG--------TAATATATTTACG | 20114 |
| YJM1273 | 16483 | ---------------------------------------------------------------------------------------------------- | 16482 |
| NCYC3585 | 15124 | ATAAAT-AATATA-----------------TTTTCTATTTTATGGTTTTTATTATTACTTT--TTCTCCACCAAACTTG----TTAATAATATAATAACA | 15199 |
| YJM1401 | 15691 | ATAAATAAATAAA-----------------------------------TTATTATTACTTTC-TTCCCCTCCGAATCCG--------TAATATATTTACG | 15746 |
| NCYC3594 | 16801 | ATAAAT-AATATA-----------------TTTTCTATTTTATGGTTTTTATTATTAC-TTT-TTCTCCACCAAACTTG----TTAATAATATAATAACA | 16876 |
| YJM1078 | 17390 | ATAAAT-AAATAA----------------------------------ATTATTATTAC-TTTCTTCCCCTCCGAATCCG--------TAATATATTTACG | 17445 |
| YJM1439 | 18376 | ATAAATAAATAAA-----------------------------------TTATTATTACTTTC-TTCCCCTCCGAATCCG--------TAATATATTTACG | 18431 |
| consensus | 24401 | ---------...------------------.--..----..------.-------------.----.--.--.--...----------------.-.--. | 24500 |
|
| S288C | 20045 | GATATATAATCTCGTAGTGTAAAAGGTGTAACGAGATTATT-----AATAA-----GTTGCCGTAATATATTGTAAAATATATTATTATTACAACACTAT | 20134 |
| YJM1388 | 20563 | AGTCAATAATTTCGTAATAT-AAAGATGTAACGAAATTATTAATAAAATAA-----GTTGCCGTAATATATTGTAAAATATATTATTATTACAACACTAT | 20656 |
| YJM789 | 20115 | GATATATAATCTCGTAGTGTAAAAGGTGTAACGAGATTATT----------AATAAGTTGCCGTAATATATTGTAAAATATATTATTATTACAACACTAT | 20204 |
| YJM1273 | 16483 | ---------------------------------------------------------------------------------------------------- | 16482 |
| NCYC3585 | 15200 | AGTCAATAATTTCGTAATAT-AAAGATGTAACGAAATTATT-----AATAAAATAAGTTGCCGTAATATATTGTAAAATATATTATTATTACAACACTAT | 15293 |
| YJM1401 | 15747 | GATATATAATCTCGTAGTGTAAAAGGTGTAACGAGATTATT----------AATAAGTTGCCGTAATATATTGTAAAATATATTATTATTACAACACTAT | 15836 |
| NCYC3594 | 16877 | AGTCAATAATTTCGTAATAT-AAAGATGTAACGAAATTATT-----AATAAAATAAGTTGCCGTAATATATTGTAAAATATATTATTATTACAACACTAT | 16970 |
| YJM1078 | 17446 | GATATATAATCTCGTAGTGTAAAAGGTGTAACGAGATTATT----------AATAAGTTGCCGTAATATATTGTAAAATATATTATTATTACAACACTAT | 17535 |
| YJM1439 | 18432 | GATATATAATCTCGTAGTGTAAAAGGTGTAACGAGATTATT----------AATAAGTTGCCGTAATATATTGTAAAATATATTATTATTACAACACTAT | 18521 |
| consensus | 24501 | ..-..-----.-----.-.------.--------.----------------------------------------------------------------- | 24600 |
|
| S288C | 20135 | ATGCGGGAAAACCCTAAAGTCATAATATAATATTATCCCCACGAGGGCCACACATGTGTGGCCCTCGCGGGGTATGGTAAATTTAATTAAGTTATAAATG | 20234 |
| YJM1388 | 20657 | ATGCGGGAAAATCCTAAAGTTATAATATAATATT-------------------------------------TTTTGGAAA-TTCAATTAAGTTATGAATG | 20718 |
| YJM789 | 20205 | ATGCGGGAAAACCCTAAAGTCATAATATAATATTATCCCCACGAGGGCCACACATGTGTGGCCCTCGCGGGGTATGGAAAATTTAATTAAGTTATAAATG | 20304 |
| YJM1273 | 16483 | ---------------------------------------------------------------------------------------------------- | 16482 |
| NCYC3585 | 15294 | ATGCGGGAAAATCCTAAAGTTATAATATAATAT---------------------TTTGT----------------GGAAA-TTCAATTAAGTTATGAATG | 15355 |
| YJM1401 | 15837 | ATGCGGGAAAACCCTAAAGTCATAATATAATATTATCCCCACGAGGGCCACACATGTGTGGCCCTCGCGGGGTATGGAAAATTTAATTAAGTTATAAATG | 15936 |
| NCYC3594 | 16971 | ATGCGGGAAAATCCTAAAGTTATAATATAATATTT---------------------TGT----------------GGAAA-TTCAATTAAGTTATGAATG | 17032 |
| YJM1078 | 17536 | ATGCGGGAAAACCCTAAAGTCATAATATAATATTATCCCCACGAGGGCCACACATGTGTGGCCCTCGCGGGGTATGGAAAATTTAATTAAGTTATAAATG | 17635 |
| YJM1439 | 18522 | ATGCGGGAAAACCCTAAAGTCATAATATAATATTATCCCCACGAGGGCCACACATGTGTGACCCTCGCGGGGTATGGAAAATTTAATTAAGTTATAAATG | 18621 |
| consensus | 24601 | -----------.--------.-------------.--------------------.----.----------.-.---.-----.-----------.---- | 24700 |
|
| S288C | 20235 | TACTATAGTATTAAAAATTATTATGAATAATTTC-------------------CCC-----------------------ACCCCCATGCGAAGCAT---- | 20288 |
| YJM1388 | 20719 | TACTATAATATTAAAAATTATTATAAATAATTAT-------------------CCTCATTAATTATAATTATAATTATAATTCTAATTATAATATA---- | 20795 |
| YJM789 | 20305 | TACTATAGTATTAAAAATTATTATGAATAATTTCCCC------------------------------------------ACCCCCATGCGAAGCAT---- | 20358 |
| YJM1273 | 16483 | ---------------------------------------------------------------------------------------------------- | 16482 |
| NCYC3585 | 15356 | TACTATAATATTAAAAATTATTATAAATAATTATCCTCATTAATTATAATTA--TA-----------------------ATTATAATTCTAATTATAATA | 15430 |
| YJM1401 | 15937 | TACTATAGTATTAAAAATTATTATGAATAATTTCCCC------------------------------------------ACCCCCATGCGAAGCAT---- | 15990 |
| NCYC3594 | 17033 | TACTATAATATTAAAAATTATTATAAATAATTATCCTCATTAATTATAATTAT--A-----------------------ATTATAATTCTAATTATAATA | 17107 |
| YJM1078 | 17636 | TACTATAGTATTAAAAATTATTATGAATAATTTCCCC------------------------------------------ACCCCCATGCGAAGCAT---- | 17689 |
| YJM1439 | 18622 | TACTATAGTATTAAAAATTATTATGAATAATTTCCCC------------------------------------------ACCCCCATGCGAAGCAT---- | 18675 |
| consensus | 24701 | -------.----------------.-------..--.-----------------..------------------------.....--...--....---- | 24800 |
|
| S288C | 20289 | --GGGGGGGGGTATAAGTATGGACAATCCGCAGGAAACCAAATAATAATTAAT-----------------ATCCTGAA-----ACAAAGTAAGTGAAGGA | 20364 |
| YJM1388 | 20796 | --AAAAGTTAGGATAAATATGGACAATCCGCAGGAAACCAAATAATAATAGGA-----------------AT-----------ATATATTAAGATATATA | 20865 |
| YJM789 | 20359 | --GGGGGGGGGTATAAGTATGGACAATCCGCAGGAAACCAAATAATAAATAATATCCTGAACAAAGTAAGTGAAGGAG-----ATATCTTAAAATATATA | 20451 |
| YJM1273 | 16483 | ---------------------------------------------------------------------------------------------------- | 16482 |
| NCYC3585 | 15431 | TAAAAAGTTAGGATAAATATGGACAATCCGCAGGAAACCAAATAATAA-----------------------TAGGAAT-----ATATATTAAGATATATA | 15502 |
| YJM1401 | 15991 | --GGGGGGGGGTATAAGTATGGACAATCCGCAGGAAACCAAATAATAATTAATATCCTGAACAAAGTAAGTGAAGGAG-----ATATCTTAAAATATATA | 16083 |
| NCYC3594 | 17108 | TAAAAAGTTAGGATAAATATGGACAATCCGCAGGAAACCAAATAATAA-----------------------TAGGAAT-----ATATATTAAGATATATA | 17179 |
| YJM1078 | 17690 | ---GGGGGGGGTATAAGTATGGACAATCCGCAGGAAACCAAATAATAA-----------------------ATAATATCCTGAACAAAGTAAGTGAAGGA | 17763 |
| YJM1439 | 18676 | --GGGGGTGGGTATAAGTATGGACAATCCGCAGGAAACCAAATAATAATTAATATCCTGA-----------------------ACAAAGTAAGTGAAGGA | 18750 |
| consensus | 24801 | --....-...-.----.-------------------------------.....-----------------......-.------.-...---...-...- | 24900 |
|
| S288C | 20365 | GATATCTTAAAATATATA-------------------------------------------------TAATATATATTTTATAAATTATTATGTAGGATC | 20415 |
| YJM1388 | 20866 | TATATATATTTATATATATTCT-TTTTTTTTTCAATATATTATATTTAGTATTATTGGAAGAGGGGGTAATATATATTT----AATTATTACGTAGGATC | 20960 |
| YJM789 | 20452 | TA---------ATATATATTT---------------------------------------------------TATA-------AATTATTACGTAGGATC | 20484 |
| YJM1273 | 16483 | ---------------------------------------------------------------------------------------------------- | 16482 |
| NCYC3585 | 15503 | TATATATATTTATATATATTCTTTTTTTTTTTCAATATATTATATTTAGTATTATTGGAAGAGGGGGTAATATATATTT----AATTATTACGTAGGATC | 15598 |
| YJM1401 | 16084 | TA---------ATATATATTT---------------------------------------------------TATA-------AATTATTACGTAGGATC | 16116 |
| NCYC3594 | 17180 | TATATATATTTATATATATTCTTTTTTTTTTTCAATATATTATATTTAGTATTATTGGAAGAGGGGGTAATATATATTT----AATTATTACGTAGGATC | 17275 |
| YJM1078 | 17764 | GATATCTTAAAATATATA-------------------------------------------------TAATATATATTTTATAAATTATTACGTAGGATC | 17814 |
| YJM1439 | 18751 | GATATCTAAAAATATATA-------------------------------------------------TAATATATATTTTATAAATTATTACGTAGGATC | 18801 |
| consensus | 24901 | .----.-....---------.----------------------------------------------------------------------.-------- | 25000 |
|
| S288C | 20416 | CTCAGAGACTACACGTGTTGCACCCATTATATTA-----------------------------TGTATAATGGGTTGAAGATATAGTCCAAATATAATTG | 20486 |
| YJM1388 | 20961 | CTCAGAGACTACACGTGTTGCACCCATTATATTAATATTATATTATATTTT------------ATTATAATGGGTTGAAGATATAGTCCAAATATAATTG | 21048 |
| YJM789 | 20485 | CTCAGAGACTACACGTGTTGCACCCATTATATTA-----------------------------TGTATAATGGGTTGAAGATATAGTCCAAATATAATTG | 20555 |
| YJM1273 | 16483 | ---------------------------------------------------------------------------------------------------- | 16482 |
| NCYC3585 | 15599 | CTCAGAGACTACACGTGTTGCACCCATTATATTAATATTAATATTAATATTATATTATATTTTATTATAATGGGTTGAAGATATAGTCCAAATATAATTG | 15698 |
| YJM1401 | 16117 | CTCAGAGACTACACGTGTTGCACCCATTATATTA-----------------------------TGTATAATGGGTTGAAGATATAGTCCAAATATAATTG | 16187 |
| NCYC3594 | 17276 | CTCAGAGACTACACGTGTTGCACCCATTATATTAATATTATATTATATTTTA------------TTATAATGGGTTGAAGATATAGTCCAAATATAATTG | 17363 |
| YJM1078 | 17815 | CTCAGAGACTACACGTGTTGCACCCATTATATTA-----------------T------------GTATAATGGGTTGAAGATATAGTCCAAATATAATTG | 17885 |
| YJM1439 | 18802 | CTCAGAGACTACACGTGTTGCACCCATTATATTA-----------------------------TGTATAATGGGTTGAAGATATAGTCCAAATATAATTG | 18872 |
| consensus | 25001 | ----------------------------------------...-..--.--.-----------..----------------------------------- | 25100 |
|
| S288C | 20487 | AAAGATTATAAT-AAAATGAACTATTTATTACCATTAATAATTGGAGCTACAGATACAGCATTTCCAAGAATTAATAACATTGCTTTTTGAGTATTACCT | 20585 |
| YJM1388 | 21049 | AAAGATTATAATAAAAATGAACTATTTATTACCATTAATGATTGGAGCTACAGATACAGCATTTCCAAGAATTAATAACATTGCTTTTTGAGTATTACCT | 21148 |
| YJM789 | 20556 | AAAGATTATAAT-AAAATGAACTATTTATTACCATTAATAATTGGAGCTACAGATACAGCATTTCCAAGAATTAATAACATTGCTTTTTGAGTATTACCT | 20654 |
| YJM1273 | 16483 | ----------------------TATTTATTACCATTAATAATTGGAGCTACAGATACAGCATTTCCAAGAATTAATAACATTGCTTTTTGAGTATTACCT | 16560 |
| NCYC3585 | 15699 | AAAGATTATAATAAAAATGAACTATTTATTACCATTAATAATTGGAGCTACAGATACAGCATTTCCAAGAATTAATAACATTGCTTTTTGAGTATTACCT | 15798 |
| YJM1401 | 16188 | AAAGATTATAAT-AAAATGAACTATTTATTACCATTAATAATTGGAGCTACAGATACAGCATTTCCAAGAATTAATAACATTGCTTTTTGAGTATTACCT | 16286 |
| NCYC3594 | 17364 | AAAGATTATAATAAAAATGAACTATTTATTACCATTAATGATTGGAGCTACAGATACAGCATTTCCAAGAATTAATAACATTGCTTTTTGAGTATTACCT | 17463 |
| YJM1078 | 17886 | AAAGATTATAAT-AAAATGAACTATTTATTACCATTAATAATTGGAGCTACAGATACAGCATTTCCAAGAATTAATAACATTGCTTTTTGAGTATTACCT | 17984 |
| YJM1439 | 18873 | AAAGATTATAAT-AAAATGAACTATTTATTACCATTAATGATTGGAGCTACAGATACAGCATTTCCAAGAATTAATAACATTGCTTTTTGAGTATTACCT | 18971 |
| consensus | 25101 | ----------------------\*\*\*\*\*\*\*\*\*\*\*\*\*\*\*\*\*.\*\*\*\*\*\*\*\*\*\*\*\*\*\*\*\*\*\*\*\*\*\*\*\*\*\*\*\*\*\*\*\*\*\*\*\*\*\*\*\*\*\*\*\*\*\*\*\*\*\*\*\*\*\*\*\*\*\*\*\* | 25200 |
|
| S288C | 20586 | ATGGGGTTAGTATGTTTAGTTACATCAACTTTAGTAGAATCAGGTGCTGGTACAGGGTGAACTGTCTATCCACCATTATCATCTATTCAGGCACATTCAG | 20685 |
| YJM1388 | 21149 | ATGGGGTTAGTATGTTTAGTTACATCAACTTTAGTAGAATCAGGTGCTGGTACAGGGTGAACTGTCTATCCACCATTATCATCTATTCAGGCACATTCAG | 21248 |
| YJM789 | 20655 | ATGGGGTTAGTATGTTTAGTTACATCAACTTTAGTAGAATCAGGTGCTGGTACAGGGTGAACTGTCTATCCACCATTATCATCTATTCAGGCACATTCAG | 20754 |
| YJM1273 | 16561 | ATGGGGTTAGTATGTTTAGTTACATCAACTTTAGTAGAATCAGGTGCTGGTACAGGGTGAACTGTCTATCCACCATTATCATCTATTCAGGCACATTCAG | 16660 |
| NCYC3585 | 15799 | ATGGGGTTAGTATGTTTAGTTACATCAACTTTAGTAGAATCAGGTGCTGGTACAGGGTGAACTGTCTATCCACCATTATCATCTATTCAGGCACATTCAG | 15898 |
| YJM1401 | 16287 | ATGGGGTTAGTATGTTTAGTTACATCAACTTTAGTAGAATCAGGTGCTGGTACAGGGTGAACTGTCTATCCACCATTATCATCTATTCAGGCACATTCAG | 16386 |
| NCYC3594 | 17464 | ATGGGGTTAGTATGTTTAGTTACATCAACTTTAGTAGAATCAGGTGCTGGTACAGGGTGAACTGTCTATCCACCATTATCATCTATTCAGGCACATTCAG | 17563 |
| YJM1078 | 17985 | ATGGGGTTAGTATGTTTAGTTACATCAACTTTAGTAGAATCAGGTGCTGGTACAGGGTGAACTGTCTATCCACCATTATCATCTATTCAGGCACATTCAG | 18084 |
| YJM1439 | 18972 | ATGGGGTTAGTATGTTTAGTTACATCAACTTTAGTAGAATCAGGTGCTGGTACAGGGTGAACTGTCTATCCACCATTATCATCTATTCAGGCACATTCAG | 19071 |
| consensus | 25201 | \*\*\*\*\*\*\*\*\*\*\*\*\*\*\*\*\*\*\*\*\*\*\*\*\*\*\*\*\*\*\*\*\*\*\*\*\*\*\*\*\*\*\*\*\*\*\*\*\*\*\*\*\*\*\*\*\*\*\*\*\*\*\*\*\*\*\*\*\*\*\*\*\*\*\*\*\*\*\*\*\*\*\*\*\*\*\*\*\*\*\*\*\*\*\*\*\*\*\*\* | 25300 |
|
| S288C | 20686 | GACCTAGTGTAGATTTAGCAATTTTTGCATTACATTTAACATCAATTTCATCATTATTAGGTGCTATTAATTTCATTGTAACAACATTAAATATGAGAAC | 20785 |
| YJM1388 | 21249 | GACCTAGTGTAGATTTAGCAATTTTTGCATTACATTTAACATCAATTTCATCATTATTAGGTGCTATTAATTTCATTGTAACAACATTAAATATGAGAAC | 21348 |
| YJM789 | 20755 | GACCTAGTGTAGATTTAGCAATTTTTGCATTACATTTAACATCAATTTCATCATTATTAGGTGCTATTAATTTCATTGTAACAACATTAAATATGAGAAC | 20854 |
| YJM1273 | 16661 | GACCTAGTGTAGATTTAGCAATTTTTGCATTACATTTAACATCAATTTCATCATTATTAGGTGCTATTAATTTCATTGTAACAACATTAAATATGAGAAC | 16760 |
| NCYC3585 | 15899 | GACCTAGTGTAGATTTAGCAATTTTTGCATTACATTTAACATCAATTTCATCATTATTAGGTGCTATTAATTTCATTGTAACAACATTAAATATGAGAAC | 15998 |
| YJM1401 | 16387 | GACCTAGTGTAGATTTAGCAATTTTTGCATTACATTTAACATCAATTTCATCATTATTAGGTGCTATTAATTTCATTGTAACAACATTAAATATGAGAAC | 16486 |
| NCYC3594 | 17564 | GACCTAGTGTAGATTTAGCAATTTTTGCATTACATTTAACATCAATTTCATCATTATTAGGTGCTATTAATTTCATTGTAACAACATTAAATATGAGAAC | 17663 |
| YJM1078 | 18085 | GACCTAGTGTAGATTTAGCAATTTTTGCATTACATTTAACATCAATTTCATCATTATTAGGTGCTATTAATTTCATTGTAACAACATTAAATATGAGAAC | 18184 |
| YJM1439 | 19072 | GACCTAGTGTAGATTTAGCAATTTTTGCATTACATTTAACATCAATTTCATCATTATTAGGTGCTATTAATTTCATTGTAACAACATTAAATATGAGAAC | 19171 |
| consensus | 25301 | \*\*\*\*\*\*\*\*\*\*\*\*\*\*\*\*\*\*\*\*\*\*\*\*\*\*\*\*\*\*\*\*\*\*\*\*\*\*\*\*\*\*\*\*\*\*\*\*\*\*\*\*\*\*\*\*\*\*\*\*\*\*\*\*\*\*\*\*\*\*\*\*\*\*\*\*\*\*\*\*\*\*\*\*\*\*\*\*\*\*\*\*\*\*\*\*\*\*\*\* | 25400 |
|
| S288C | 20786 | AAATGGTATGACAATGCATAAATTACCATTATTTGTATGATCAATTTTCATTACAGCGTTCTTATTATTATTATCATTACCTGTATTATCTGCTGGTATT | 20885 |
| YJM1388 | 21349 | AAATGGTATGACAATGCATAAATTACCATTATTTGTATGATCAATTTTCATTACAGCGTTCTTATTATTATTATCATTACCTGTATTATCTGCTGGTATT | 21448 |
| YJM789 | 20855 | AAATGGTATGACAATGCATAAATTACCATTATTTGTATGATCAATTTTCATTACAGCGTTCTTATTATTATTATCATTACCTGTATTATCTGCTGGTATT | 20954 |
| YJM1273 | 16761 | AAATGGTATGACAATGCATAAATTACCATTATTTGTATGATCAATTTTCATTACAGCGTTCTTATTATTATTATCATTACCTGTATTATCTGCTGGTATT | 16860 |
| NCYC3585 | 15999 | AAATGGTATGACAATGCATAAATTACCATTATTTGTATGATCAATTTTCATTACAGCGTTCTTATTATTATTATCATTACCTGTATTATCTGCTGGTATT | 16098 |
| YJM1401 | 16487 | AAATGGTATGACAATGCATAAATTACCATTATTTGTATGATCAATTTTCATTACAGCGTTCTTATTATTATTATCATTACCTGTATTATCTGCTGGTATT | 16586 |
| NCYC3594 | 17664 | AAATGGTATGACAATGCATAAATTACCATTATTTGTATGATCAATTTTCATTACAGCGTTCTTATTATTATTATCATTACCTGTATTATCTGCTGGTATT | 17763 |
| YJM1078 | 18185 | AAATGGTATGACAATGCATAAATTACCATTATTTGTATGATCAATTTTCATTACAGCGTTCTTATTATTATTATCATTACCTGTATTATCTGCTGGTATT | 18284 |
| YJM1439 | 19172 | AAATGGTATGACAATGCATAAATTACCATTATTTGTATGATCAATTTTCATTACAGCGTTCTTATTATTATTATCATTACCTGTATTATCTGCTGGTATT | 19271 |
| consensus | 25401 | \*\*\*\*\*\*\*\*\*\*\*\*\*\*\*\*\*\*\*\*\*\*\*\*\*\*\*\*\*\*\*\*\*\*\*\*\*\*\*\*\*\*\*\*\*\*\*\*\*\*\*\*\*\*\*\*\*\*\*\*\*\*\*\*\*\*\*\*\*\*\*\*\*\*\*\*\*\*\*\*\*\*\*\*\*\*\*\*\*\*\*\*\*\*\*\*\*\*\*\* | 25500 |
|
| S288C | 20886 | ACAATGTTATTATTAGATAGAAACTTCAATACTTCATTCTTTGAAGTATCAGGAGGTGGTGACCCAATCTTATACGAGCATTTATTTT------------ | 20973 |
| YJM1388 | 21449 | ACAATGTTATTATTAGATAGAAACTTCAATACTTCATTCTTTGAAGTAGCAGGAGGTGGTGACCCAATCTTATATGAGCATTTATTTT------------ | 21536 |
| YJM789 | 20955 | ACAATGTTATTATTAGATAGAAACTTCAATACTTCATTCTTTGAAGTAGCAGGAGGTGGTGACCCAATCTTATATGAGCATTTATTTTACAAAGGCTACA | 21054 |
| YJM1273 | 16861 | ACAATGTTATTATTAGATAGAAACTTCAATACTTCATTCTTTGAAGTAGCAGGAGGTGGTGACCCAATCTTATATGAGCATTTATTTT------------ | 16948 |
| NCYC3585 | 16099 | ACAATGTTATTATTAGATAGAAACTTCAATACTTCATTCTTTGAAGTAGCAGGAGGTGGTGACCCAATCTTATACGAGCATTTATTTT------------ | 16186 |
| YJM1401 | 16587 | ACAATGTTATTATTAGATAGAAACTTCAATACTTCATTCTTTGAAGTAGCAGGAGGTGGTGACCCAATCTTATACGAGCATTTATTTT------------ | 16674 |
| NCYC3594 | 17764 | ACAATGTTATTATTAGATAGAAACTTCAATACTTCATTCTTTGAAGTAGCAGGAGGTGGTGACCCAATCTTATATGAGCATTTATTTT------------ | 17851 |
| YJM1078 | 18285 | ACAATGTTATTATTAGATAGAAACTTCAATACTTCATTCTTTGAAGTAGCAGGAGGTGGTGACCCAATCTTATATGAGCATTTATTTTACAAAGGCTACA | 18384 |
| YJM1439 | 19272 | ACAATGTTATTATTAGATAGAAACTTCAATACTTCATTCTTTGAAGTAGCAGGAGGTGGTGACCCAATCTTATACGAGCATTTATTTT------------ | 19359 |
| consensus | 25501 | \*\*\*\*\*\*\*\*\*\*\*\*\*\*\*\*\*\*\*\*\*\*\*\*\*\*\*\*\*\*\*\*\*\*\*\*\*\*\*\*\*\*\*\*\*\*\*\*.\*\*\*\*\*\*\*\*\*\*\*\*\*\*\*\*\*\*\*\*\*\*\*\*\*.\*\*\*\*\*\*\*\*\*\*\*\*\*------------ | 25600 |
|
| S288C | 20974 | ---------------------------------------------------------------------------------------------------- | 20973 |
| YJM1388 | 21537 | ---------------------------------------------------------------------------------------------------- | 21536 |
| YJM789 | 21055 | TAATAAATAATAATGTTATTTTAAATTTCTTACCAATAATATTATTATTATTAAATAATGATTCTTATATCATAATAATAATAAAATTAACAATATTATT | 21154 |
| YJM1273 | 16949 | ---------------------------------------------------------------------------------------------------- | 16948 |
| NCYC3585 | 16187 | ---------------------------------------------------------------------------------------------------- | 16186 |
| YJM1401 | 16675 | ---------------------------------------------------------------------------------------------------- | 16674 |
| NCYC3594 | 17852 | ---------------------------------------------------------------------------------------------------- | 17851 |
| YJM1078 | 18385 | TAATAAATAATAATGTTATTTTAAATTTCTTACCAATAATATTATTATTATTAAATAATGATTCTTATATCATAATAATAATAAAATTAACAATATTATT | 18484 |
| YJM1439 | 19360 | ---------------------------------------------------------------------------------------------------- | 19359 |
| consensus | 25601 | ---------------------------------------------------------------------------------------------------- | 25700 |
|
| S288C | 20974 | ---------------------------------------------------------------------------------------------------- | 20973 |
| YJM1388 | 21537 | ---------------------------------------------------------------------------------------------------- | 21536 |
| YJM789 | 21155 | ATCTAATAATCATCTATTATTATTATCTTCATCTA------TAAATAATAAAGATAAACTATCAATAAAATTAGATACTCCTTTTCGGGGTTTCGGTTCC | 21248 |
| YJM1273 | 16949 | ---------------------------------------------------------------------------------------------------- | 16948 |
| NCYC3585 | 16187 | ---------------------------------------------------------------------------------------------------- | 16186 |
| YJM1401 | 16675 | ---------------------------------------------------------------------------------------------------- | 16674 |
| NCYC3594 | 17852 | ---------------------------------------------------------------------------------------------------- | 17851 |
| YJM1078 | 18485 | ATCTAATAATCATCTATTATTATTATCTTCATCTATAAATATAAATAATAAAGATAAACTATCAATAAAATTAGAAACTCCTTTTCGGGGTTTCGGTTCC | 18584 |
| YJM1439 | 19360 | ---------------------------------------------------------------------------------------------------- | 19359 |
| consensus | 25701 | ---------------------------------------------------------------------------.------------------------ | 25800 |
|
| S288C | 20974 | ---------------------------------------------------------------------------------------------------- | 20973 |
| YJM1388 | 21537 | ---------------------------------------------------------------------------------------------------- | 21536 |
| YJM789 | 21249 | GTGTCGGACCCCAAAACTTCTAAAGATACTTTAAAATCATTATTAGATTTAACAAATAATGACATTAATAAATATAATTGAGAATGAGATAATAATAATT | 21348 |
| YJM1273 | 16949 | ---------------------------------------------------------------------------------------------------- | 16948 |
| NCYC3585 | 16187 | ---------------------------------------------------------------------------------------------------- | 16186 |
| YJM1401 | 16675 | ---------------------------------------------------------------------------------------------------- | 16674 |
| NCYC3594 | 17852 | ---------------------------------------------------------------------------------------------------- | 17851 |
| YJM1078 | 18585 | GTGTCGGACCCAAAAACTTCTAAAGATACTATAAAATCATTATTAGATTTAACAAATAATGACATTAATAAATATAATTGAGAATGAGATAATAATAATT | 18684 |
| YJM1439 | 19360 | ---------------------------------------------------------------------------------------------------- | 19359 |
| consensus | 25801 | -----------.------------------.--------------------------------------------------------------------- | 25900 |
|
| S288C | 20974 | ---------------------------------------------------------------------------------------------------- | 20973 |
| YJM1388 | 21537 | ---------------------------------------------------------------------------------------------------- | 21536 |
| YJM789 | 21349 | TTAATTTTGATAAATTTTATAAAGAATTTAAAAAAGTTAAACCTAATAATAAATTACCATCTAAAGAATTTTTAGAATGATTTATTGGTTTTTTTGAAGC | 21448 |
| YJM1273 | 16949 | ---------------------------------------------------------------------------------------------------- | 16948 |
| NCYC3585 | 16187 | ---------------------------------------------------------------------------------------------------- | 16186 |
| YJM1401 | 16675 | ---------------------------------------------------------------------------------------------------- | 16674 |
| NCYC3594 | 17852 | ---------------------------------------------------------------------------------------------------- | 17851 |
| YJM1078 | 18685 | TTAATTTTGATAAATTTTATAAAGAATTTAAAAAAGTTAAACCTAATAATAAATTACCATCTAAAGAATTTTTAGAATGATTTATTGGTTTTTTTGAAGC | 18784 |
| YJM1439 | 19360 | ---------------------------------------------------------------------------------------------------- | 19359 |
| consensus | 25901 | ---------------------------------------------------------------------------------------------------- | 26000 |
|
| S288C | 20974 | ---------------------------------------------------------------------------------------------------- | 20973 |
| YJM1388 | 21537 | ---------------------------------------------------------------------------------------------------- | 21536 |
| YJM789 | 21449 | TGATGGTTGTTTACTTATTCCTAAAAATAAACAATTATATGCTATTATTCTTTCTAATAGTAAAGATTTAAATCTATTAAATTATATTAAAGATAATATA | 21548 |
| YJM1273 | 16949 | ---------------------------------------------------------------------------------------------------- | 16948 |
| NCYC3585 | 16187 | ---------------------------------------------------------------------------------------------------- | 16186 |
| YJM1401 | 16675 | ---------------------------------------------------------------------------------------------------- | 16674 |
| NCYC3594 | 17852 | ---------------------------------------------------------------------------------------------------- | 17851 |
| YJM1078 | 18785 | TGATGGTTGTTTACTTATTCCTAAAAATAAACAATTATATGCTATTATTCTTTCTAATAATAAAGATTTAAATCTATTAAATTATATTAAAGATAATATA | 18884 |
| YJM1439 | 19360 | ---------------------------------------------------------------------------------------------------- | 19359 |
| consensus | 26001 | -----------------------------------------------------------.---------------------------------------- | 26100 |
|
| S288C | 20974 | ---------------------------------------------------------------------------------------------------- | 20973 |
| YJM1388 | 21537 | ---------------------------------------------------------------------------------------------------- | 21536 |
| YJM789 | 21549 | ACATTTGGTAATGTACTATATCATTCAAAAAAATTAAATACTTATAGATGAGTAGTATATAATGAAACAGATATTTTATTATTAATTCATTTATTTAATG | 21648 |
| YJM1273 | 16949 | ---------------------------------------------------------------------------------------------------- | 16948 |
| NCYC3585 | 16187 | ---------------------------------------------------------------------------------------------------- | 16186 |
| YJM1401 | 16675 | ---------------------------------------------------------------------------------------------------- | 16674 |
| NCYC3594 | 17852 | ---------------------------------------------------------------------------------------------------- | 17851 |
| YJM1078 | 18885 | ACATTTGGTAATGTACTATATCATTCAAAAAAATTAAATACTTATAGATGAGTAGTATATAATGAAACAGATATTTTATTATTAATTCATTTATTTAATG | 18984 |
| YJM1439 | 19360 | ---------------------------------------------------------------------------------------------------- | 19359 |
| consensus | 26101 | ---------------------------------------------------------------------------------------------------- | 26200 |
|
| S288C | 20974 | ---------------------------------------------------------------------------------------------------- | 20973 |
| YJM1388 | 21537 | ---------------------------------------------------------------------------------------------------- | 21536 |
| YJM789 | 21649 | GTAATCTAGTATTACCTGTAAGATATGTAAAATTAGAAATATTTATTTCTAATATAAATATAAAATTATTAAAAAATAATAAACTTATTATTAAATTAAT | 21748 |
| YJM1273 | 16949 | ---------------------------------------------------------------------------------------------------- | 16948 |
| NCYC3585 | 16187 | ---------------------------------------------------------------------------------------------------- | 16186 |
| YJM1401 | 16675 | ---------------------------------------------------------------------------------------------------- | 16674 |
| NCYC3594 | 17852 | ---------------------------------------------------------------------------------------------------- | 17851 |
| YJM1078 | 18985 | GTAATCTAGTATTACCTGTAAGATATGTAAAATTAGAAATATTTATTTCTAATATAAATATAAAATTATTAAAAAATAATAAACTTATTATTAAATTAAT | 19084 |
| YJM1439 | 19360 | ---------------------------------------------------------------------------------------------------- | 19359 |
| consensus | 26201 | ---------------------------------------------------------------------------------------------------- | 26300 |
|
| S288C | 20974 | ---------------------------------------------------------------------------------------------------- | 20973 |
| YJM1388 | 21537 | ---------------------------------------------------------------------------------------------------- | 21536 |
| YJM789 | 21749 | TAATAAATGTAAAATACCTAAATTAAATAATGCTTGATTAGCAGGATTTACGGATGGTGAAGGTTGTTTTTATGTAGGTAAAACTCAATCTTTTTATCGT | 21848 |
| YJM1273 | 16949 | ---------------------------------------------------------------------------------------------------- | 16948 |
| NCYC3585 | 16187 | ---------------------------------------------------------------------------------------------------- | 16186 |
| YJM1401 | 16675 | ---------------------------------------------------------------------------------------------------- | 16674 |
| NCYC3594 | 17852 | ---------------------------------------------------------------------------------------------------- | 17851 |
| YJM1078 | 19085 | TAATAAATGTAAAATACCTAAATTAAATAATGCTTGATTAGCAGGATTTACGGATGGTGAAGGTTGTTTTTATGTAGGTAAAACTCAATCTTTTTATCGT | 19184 |
| YJM1439 | 19360 | ---------------------------------------------------------------------------------------------------- | 19359 |
| consensus | 26301 | ---------------------------------------------------------------------------------------------------- | 26400 |
|
| S288C | 20974 | ---------------------------------------------------------------------------------------------------- | 20973 |
| YJM1388 | 21537 | ---------------------------------------------------------------------------------------------------- | 21536 |
| YJM789 | 21849 | CTAAGTTATATTATTACTCAAAAATATTTAGCTAATAAAATTGTTTTTGATATATTATTATTATTATTACAAAATATAATTAATATTAAATCTGGTGGTG | 21948 |
| YJM1273 | 16949 | ---------------------------------------------------------------------------------------------------- | 16948 |
| NCYC3585 | 16187 | ---------------------------------------------------------------------------------------------------- | 16186 |
| YJM1401 | 16675 | ---------------------------------------------------------------------------------------------------- | 16674 |
| NCYC3594 | 17852 | ---------------------------------------------------------------------------------------------------- | 17851 |
| YJM1078 | 19185 | CTAAGTTATATTATTACTCAAAAATATTTAGCTAATAAAATTGTTTTTGATATATTATTATTATTATTACAAAATATAATTAATATTAAATCTGGTGGTG | 19284 |
| YJM1439 | 19360 | ---------------------------------------------------------------------------------------------------- | 19359 |
| consensus | 26401 | ---------------------------------------------------------------------------------------------------- | 26500 |
|
| S288C | 20974 | ---------------------------------------------------------------------------------------------------- | 20973 |
| YJM1388 | 21537 | ---------------------------------------------------------------------------------------------------- | 21536 |
| YJM789 | 21949 | TTAATAATCATTCTAAAGATAATCTTTATGTTTTACGTATTAGTAGTTTAGAAGCTTGTTCTAAATTAAAATTATATTTTGATAAATATCCTTTAAGAAG | 22048 |
| YJM1273 | 16949 | ---------------------------------------------------------------------------------------------------- | 16948 |
| NCYC3585 | 16187 | ---------------------------------------------------------------------------------------------------- | 16186 |
| YJM1401 | 16675 | ---------------------------------------------------------------------------------------------------- | 16674 |
| NCYC3594 | 17852 | ---------------------------------------------------------------------------------------------------- | 17851 |
| YJM1078 | 19285 | TTAATAATCATTCTAAAGATAATCTTTATGTTTTACGTATTAGTAGTTTAGAAGCTTGTTCTAAATTAAAATTATATTTTGATAAATATCCTTTAAGAAG | 19384 |
| YJM1439 | 19360 | ---------------------------------------------------------------------------------------------------- | 19359 |
| consensus | 26501 | ---------------------------------------------------------------------------------------------------- | 26600 |
|
| S288C | 20974 | ---------------------------------------------------------------------------------------------------- | 20973 |
| YJM1388 | 21537 | ---------------------------------------------------------------------------------------------------- | 21536 |
| YJM789 | 22049 | TTATAAATTATTAATCTATAAAGATTGATTAAATTTTATTGATATAGCTATAAATGATAATTTATCAAAAAATATTAGAAAAGTTAATTTAATAAAAATA | 22148 |
| YJM1273 | 16949 | ---------------------------------------------------------------------------------------------------- | 16948 |
| NCYC3585 | 16187 | ---------------------------------------------------------------------------------------------------- | 16186 |
| YJM1401 | 16675 | ---------------------------------------------------------------------------------------------------- | 16674 |
| NCYC3594 | 17852 | ---------------------------------------------------------------------------------------------------- | 17851 |
| YJM1078 | 19385 | TTATAAATTATTAATCTATAAAGATTGATTAAATTTTATTGATATAGCTATAAATGATAATTTATCAAAAAATATTAGAAAAGTTAATTTAATAAAAATA | 19484 |
| YJM1439 | 19360 | ---------------------------------------------------------------------------------------------------- | 19359 |
| consensus | 26601 | ---------------------------------------------------------------------------------------------------- | 26700 |
|
| S288C | 20974 | ---------------------------------------------------------------------------------------------------- | 20973 |
| YJM1388 | 21537 | ---------------------------------------------------------------------------------------------------- | 21536 |
| YJM789 | 22149 | TTAGATACTATT---------------------------------------AGAAATAAAAAGAAATTATTATAATTTATAA-AAAAATTTTTTTAATTT | 22208 |
| YJM1273 | 16949 | ---------------------------------------------------------------------------------------------------- | 16948 |
| NCYC3585 | 16187 | ---------------------------------------------------------------------------------------------------- | 16186 |
| YJM1401 | 16675 | ---------------------------------------------------------------------------------------------------- | 16674 |
| NCYC3594 | 17852 | ---------------------------------------------------------------------------------------------------- | 17851 |
| YJM1078 | 19485 | TTAGATATTATTAGTTCCGGGGCCCGGCACGGGAGCCAGAACCCCGGACGGAGAAATAAAAAGAAATTATTATAATTTATAAAAAAAAATTTTTTAATTT | 19584 |
| YJM1439 | 19360 | ---------------------------------------------------------------------------------------------------- | 19359 |
| consensus | 26701 | -------.--------------------------------------------------------------------------------.----------- | 26800 |
|
| S288C | 20974 | ---------------------------------------------------------------------------------------------------- | 20973 |
| YJM1388 | 21537 | ---------------------------------------------------------------------------------------------------- | 21536 |
| YJM789 | 22209 | ATTATGTAAAGAGAAATAAAAAATATAATTAAATGTAAATTTATATATAAATATATATTATTTTATTATTCTTTATTTATTAAAGAAATAATAATAATAT | 22308 |
| YJM1273 | 16949 | ---------------------------------------------------------------------------------------------------- | 16948 |
| NCYC3585 | 16187 | ---------------------------------------------------------------------------------------------------- | 16186 |
| YJM1401 | 16675 | ---------------------------------------------------------------------------------------------------- | 16674 |
| NCYC3594 | 17852 | ---------------------------------------------------------------------------------------------------- | 17851 |
| YJM1078 | 19585 | ATTATGTAAAGAGAAATAAAAAATATAATTAAATGTAAATTTATATATAAATATATATTATTTTATTATTCTTTATTTATTAAAGAAATAATAATAATAT | 19684 |
| YJM1439 | 19360 | ---------------------------------------------------------------------------------------------------- | 19359 |
| consensus | 26801 | ---------------------------------------------------------------------------------------------------- | 26900 |
|
| S288C | 20974 | ---------------------------------------------------------------------------------------------------- | 20973 |
| YJM1388 | 21537 | ---------------------------------------------------------------------------------------------------- | 21536 |
| YJM789 | 22309 | TTATATAATATTTTTTATATAGTTTTATATCTTTTATTATTTGAAACTTTTTATTTATTAATAGTTCCGGGGCCCGGCCACGGGAGCCGGAACCCCGAAA | 22408 |
| YJM1273 | 16949 | ---------------------------------------------------------------------------------------------------- | 16948 |
| NCYC3585 | 16187 | ---------------------------------------------------------------------------------------------------- | 16186 |
| YJM1401 | 16675 | ---------------------------------------------------------------------------------------------------- | 16674 |
| NCYC3594 | 17852 | ---------------------------------------------------------------------------------------------------- | 17851 |
| YJM1078 | 19685 | TTATATAATATTTTTTATATAGTTTTATATCTTTTATTATTTGAAACTTTTTATTTATTAATAGTTCCGGGGCCCGGCCACGGGAGCCGGAACCCCGAAA | 19784 |
| YJM1439 | 19360 | ---------------------------------------------------------------------------------------------------- | 19359 |
| consensus | 26901 | ---------------------------------------------------------------------------------------------------- | 27000 |
|
| S288C | 20974 | ---------------------------------------------------------------------------------------------------- | 20973 |
| YJM1388 | 21537 | ---------------------------------------------------------------------------------------------------- | 21536 |
| YJM789 | 22409 | GGAGTTCATCTATTTAATTATATATGGACAATAATTAAATAATAGTATTTATTTATCTTAAAGATATATATAATAAATATAAAGAATATGTTATATTTTA | 22508 |
| YJM1273 | 16949 | ---------------------------------------------------------------------------------------------------- | 16948 |
| NCYC3585 | 16187 | ---------------------------------------------------------------------------------------------------- | 16186 |
| YJM1401 | 16675 | ---------------------------------------------------------------------------------------------------- | 16674 |
| NCYC3594 | 17852 | ---------------------------------------------------------------------------------------------------- | 17851 |
| YJM1078 | 19785 | GGAGTTCATCTATTTAATTATATATGGACAATAATTAAATAATAGTATTTATTTATCTTAAAGATATATATAATAAATATAAAGAATATGTTATATTTTA | 19884 |
| YJM1439 | 19360 | ---------------------------------------------------------------------------------------------------- | 19359 |
| consensus | 27001 | ---------------------------------------------------------------------------------------------------- | 27100 |
|
| S288C | 20974 | ---------------------------------------------------------------------------------------------------- | 20973 |
| YJM1388 | 21537 | ---------------------------------------------------------------------------------------------------- | 21536 |
| YJM789 | 22509 | GTTTTTATATATTATAATTATATATATACTTAATTTATATATATAACGTAAAAATTACAAAATGTAATTTTTATTGATAATTATCTTATTATTATTATAT | 22608 |
| YJM1273 | 16949 | ---------------------------------------------------------------------------------------------------- | 16948 |
| NCYC3585 | 16187 | ---------------------------------------------------------------------------------------------------- | 16186 |
| YJM1401 | 16675 | ---------------------------------------------------------------------------------------------------- | 16674 |
| NCYC3594 | 17852 | ---------------------------------------------------------------------------------------------------- | 17851 |
| YJM1078 | 19885 | GTTTTTATATATTATAATTATATATATACTTAATTTATATATATAACGTAAAAATTACAAAATGTAATTTTTATTGATAATTATCTTATTATTATTATAT | 19984 |
| YJM1439 | 19360 | ---------------------------------------------------------------------------------------------------- | 19359 |
| consensus | 27101 | ---------------------------------------------------------------------------------------------------- | 27200 |
|
| S288C | 20974 | ---------------------------------------------------------------------------------------------------- | 20973 |
| YJM1388 | 21537 | ---------------------------------------------------------------------------------------------------- | 21536 |
| YJM789 | 22609 | TAAATTATATAATATAATAAGTTTATATTAATAATAATTATTATAATATAAAGTATATAAAAGCGACACTGATAGTACGGTTAATAATCTTCTTTAGGAT | 22708 |
| YJM1273 | 16949 | ---------------------------------------------------------------------------------------------------- | 16948 |
| NCYC3585 | 16187 | ---------------------------------------------------------------------------------------------------- | 16186 |
| YJM1401 | 16675 | ---------------------------------------------------------------------------------------------------- | 16674 |
| NCYC3594 | 17852 | ---------------------------------------------------------------------------------------------------- | 17851 |
| YJM1078 | 19985 | TAAATTATATAATATAATAAGTTTATATTAATAATAATTATTATAATATAAAGTATATAAAAGCGACACTGATAGTACGGTTAATAATCTTCTTTAGGAT | 20084 |
| YJM1439 | 19360 | ---------------------------------------------------------------------------------------------------- | 19359 |
| consensus | 27201 | ---------------------------------------------------------------------------------------------------- | 27300 |
|
| S288C | 20974 | ---------------------------------------------------------------------------------------------------- | 20973 |
| YJM1388 | 21537 | ---------------------------------------------------------------------------------------------------- | 21536 |
| YJM789 | 22709 | CAAGACCGTCGGTTAATTAAGTGATCGCTACAGACTGCTTTATCGGTGGCTTTAATAATATATATATATATATATATATATATATATTATAAGGTTAATG | 22808 |
| YJM1273 | 16949 | ---------------------------------------------------------------------------------------------------- | 16948 |
| NCYC3585 | 16187 | ---------------------------------------------------------------------------------------------------- | 16186 |
| YJM1401 | 16675 | ---------------------------------------------------------------------------------------------------- | 16674 |
| NCYC3594 | 17852 | ---------------------------------------------------------------------------------------------------- | 17851 |
| YJM1078 | 20085 | CAAGACCGTCGGTTAATTAAGTGATCGCTACAGACTGCTTTATCGGTGGCTTTAATAATATATATATATATATATATATATATATATTATAAGGTTAATG | 20184 |
| YJM1439 | 19360 | ---------------------------------------------------------------------------------------------------- | 19359 |
| consensus | 27301 | ---------------------------------------------------------------------------------------------------- | 27400 |
|
| S288C | 20974 | ---------------------------------------------------------------------------------------------------- | 20973 |
| YJM1388 | 21537 | ---------------------------------------------------------------------------------------------------- | 21536 |
| YJM789 | 22809 | TACAGTCGGAACTCTCAATATATATATATATTTTTTTTTTTATTAAATAAATATATATATAAATTGGTTATTATTATTAAATAATAAATAAAATATATTG | 22908 |
| YJM1273 | 16949 | ---------------------------------------------------------------------------------------------------- | 16948 |
| NCYC3585 | 16187 | ---------------------------------------------------------------------------------------------------- | 16186 |
| YJM1401 | 16675 | ---------------------------------------------------------------------------------------------------- | 16674 |
| NCYC3594 | 17852 | ---------------------------------------------------------------------------------------------------- | 17851 |
| YJM1078 | 20185 | TACAGTCGGAACTCTCAATATATATATATA--TTTTTTTTTATTAAATAAATATATATATAAATTGGTTATTATTATTAAATAATAAATAAAATATATTG | 20282 |
| YJM1439 | 19360 | ---------------------------------------------------------------------------------------------------- | 19359 |
| consensus | 27401 | ---------------------------------------------------------------------------------------------------- | 27500 |
|
| S288C | 20974 | --------------------------------GATTCTTTGGTCAAACAGTGGCCCTTATTATTATATTAATAATATATAATGATATGCATTTTTCTAAA | 21041 |
| YJM1388 | 21537 | --------------------------------GATTCTTTGGT--------------------------------------------------------- | 21547 |
| YJM789 | 22909 | TAAGTGAAATATTTATATTTTATATGAGTTTGGATTCTTTGGTCAAACAGTGGCCCTTATTATTATATTAATAATATATAATGATATGCATTTTTCTAAA | 23008 |
| YJM1273 | 16949 | --------------------------------GATTCTTTGGT--------------------------------------------------------- | 16959 |
| NCYC3585 | 16187 | --------------------------------GATTCTTTGGTCAAACAGTGGCCCTTATTATTATATTAATAATATATAATGATATGCATTTTTCTAAA | 16254 |
| YJM1401 | 16675 | --------------------------------GATTCTTTGGTCAAACAGTGGCCCTTATTATTATATTAATAATATATAATGATATGCATTTTTCTAAA | 16742 |
| NCYC3594 | 17852 | --------------------------------GATTCTTTGGT--------------------------------------------------------- | 17862 |
| YJM1078 | 20283 | TAAGTGAAATATTTATATTTTATATGAGTTTGGATTCTTTGGT--------------------------------------------------------- | 20325 |
| YJM1439 | 19360 | --------------------------------GATTCTTTGGTCAAACAGTGGCCCTTATTATTATATTAATAATATATAATGATATGCATTTTTCTAAA | 19427 |
| consensus | 27501 | --------------------------------\*\*\*\*\*\*\*\*\*\*\*--------------------------------------------------------- | 27600 |
|
| S288C | 21042 | TGCTGGAAATTATTAAAAAAATGAATTACAAATATTATAAGTCTATTATTTAAAGCCTTATTTGTAAAAATATTCATATCTTATAATAATCAGCAGGATA | 21141 |
| YJM1388 | 21548 | ---------------------------------------------------------------------------------------------------- | 21547 |
| YJM789 | 23009 | TGCTGGAAATTATTAAAAAAATGAATTACAAATATTATAAGTCTATTATTTAAAGCCTTATTTGTAAAAATATTCATATCTTATAATAATCAGCAGGATA | 23108 |
| YJM1273 | 16960 | ---------------------------------------------------------------------------------------------------- | 16959 |
| NCYC3585 | 16255 | TGCTGGAAATTATTAAAAAAATGAATTACAAATATTATAAGTCTATTATTTAAAGCCTTATTTGTAAAAATATTCATATCTTATAATAATCAGCAGGATA | 16354 |
| YJM1401 | 16743 | TGCTGGAAATTATTAAAAAAATGAATTACAAATATTATAAGTCTATTATTTAAAGCCTTATTTGTAAAAATATTCATATCTTATAATAATCAGCAGGATA | 16842 |
| NCYC3594 | 17863 | ---------------------------------------------------------------------------------------------------- | 17862 |
| YJM1078 | 20326 | ---------------------------------------------------------------------------------------------------- | 20325 |
| YJM1439 | 19428 | TGCTGGAAATTATTAAAAAAATGAATTACAAATATTATAAGTCTATTATTTAAAGCCTTATTTGTAAAAATATTCATATCTTATAATAATCAGCAGGATA | 19527 |
| consensus | 27601 | ---------------------------------------------------------------------------------------------------- | 27700 |
|
| S288C | 21142 | AGATAATAAATAATCTTATATTAAAAAAAGATAATATTAAAAGATCCTCAGAGACTACAAGAAAAATATTAAATAATTCAATAAATAAAAAATTTAATCA | 21241 |
| YJM1388 | 21548 | ---------------------------------------------------------------------------------------------------- | 21547 |
| YJM789 | 23109 | AGATAATAAATAATCTTATATTAAAAAAAGATAATATTAAAAGATCCTCAGAGACTACAAGAAAAATATTAAATAATTCAATAAATAAAAAATTTAATCA | 23208 |
| YJM1273 | 16960 | ---------------------------------------------------------------------------------------------------- | 16959 |
| NCYC3585 | 16355 | AGATAATAAATAATCTTATATTAAAAAAAGATAATATTAAAAGATCCTCAGAGACTACAAGAAAAATATTAAATAATTCAATAAATAAAAAATTTAATCA | 16454 |
| YJM1401 | 16843 | AGATAATAAATAATCTTATATTAAAAAAAGATAATATTAAAAGATCCTCAGAGACTACAAGAAAAATATTAAATAATTCAATAAATAAAAAATTTAATCA | 16942 |
| NCYC3594 | 17863 | ---------------------------------------------------------------------------------------------------- | 17862 |
| YJM1078 | 20326 | ---------------------------------------------------------------------------------------------------- | 20325 |
| YJM1439 | 19528 | AGATAATAAATAATCTTATATTAAAAAAAGATAATATTAAAAGATCCTCAGAGACTACAAGAAAAATATTAAATAATTCAATAAATAAAAAATTTAATCA | 19627 |
| consensus | 27701 | ---------------------------------------------------------------------------------------------------- | 27800 |
|
| S288C | 21242 | ATGATTAGCTGGATTAATTGATGGTGATGGATATTTTGGTATTGTAAGTAAGAAATATGTATCATTAGAAATTCTAGTAGCATTAGAAGATGAAATAGCT | 21341 |
| YJM1388 | 21548 | ---------------------------------------------------------------------------------------------------- | 21547 |
| YJM789 | 23209 | ATGATTAGCTGGATTAATTGATGGTGATGGATATTTTGGTATTGTAAGTAAGAAATATGTATCATTAGAAATTCTAGTAGCATTAGAAGATGAAATAGCT | 23308 |
| YJM1273 | 16960 | ---------------------------------------------------------------------------------------------------- | 16959 |
| NCYC3585 | 16455 | ATGATTAGCTGGATTAATTGATGGTGATGGATATTTTGGTATTGTAAGTAAGAAATATGTATCATTAGAAATTCTAGTAGCATTAGAAGATGAAATAGCT | 16554 |
| YJM1401 | 16943 | ATGATTAGCTGGATTAATTGATGGTGATGGATATTTTGGTATTGTAAGTAAGAAATATGTATCATTAGAAATTCTAGTAGCATTAGAAGATGAAATAGCT | 17042 |
| NCYC3594 | 17863 | ---------------------------------------------------------------------------------------------------- | 17862 |
| YJM1078 | 20326 | ---------------------------------------------------------------------------------------------------- | 20325 |
| YJM1439 | 19628 | ATGATTAGCTGGATTAATTGATGGTGATGGATATTTTGGTATTGTAAGTAAGAAATATGTATCATTAGAAATTCTAGTAGCATTAGAAGATGAAATAGCT | 19727 |
| consensus | 27801 | ---------------------------------------------------------------------------------------------------- | 27900 |
|
| S288C | 21342 | TTAAAAGAAATTCAAAATAAATTTGGTGGTTCTATTAAATTAAGATCAGGTGTAAAAGCTATTAGATATAGATTACTTAATAAAACTGGTATAATTAAAT | 21441 |
| YJM1388 | 21548 | ---------------------------------------------------------------------------------------------------- | 21547 |
| YJM789 | 23309 | TTAAAAGAAATTCAAAATAAATTTGGTGGTTCTATTAAATTAAGATCAGGTGTAAAAGCTATTAGATATAGATTACTTAATAAAACTGGTATAATTAAAT | 23408 |
| YJM1273 | 16960 | ---------------------------------------------------------------------------------------------------- | 16959 |
| NCYC3585 | 16555 | TTAAAAGAAATTCAAAATAAATTTGGTGGTTCTATTAAATTAAGATCAGGTGTAAAAGCTATTAGATATAGATTACTTAATAAAACTGGTATAATTAAAT | 16654 |
| YJM1401 | 17043 | TTAAAAGAAATTCAAAATAAATTTGGTGGTTCTATTAAATTAAGATCAGGTGTAAAAGCTATTAGATATAGATTACTTAATAAAACTGGTATAATTAAAT | 17142 |
| NCYC3594 | 17863 | ---------------------------------------------------------------------------------------------------- | 17862 |
| YJM1078 | 20326 | ---------------------------------------------------------------------------------------------------- | 20325 |
| YJM1439 | 19728 | TTAAAAGAAATTCAAAATAAATTTGGTGGTTCTATTAAATTAAGATCAGGTGTAAAAGCTATTAGATATAGATTACTTAATAAAACTGGTATAATTAAAT | 19827 |
| consensus | 27901 | ---------------------------------------------------------------------------------------------------- | 28000 |
|
| S288C | 21442 | TAATTAATGCAGTTAATGGTAATATTAGAAATACTAAAAGATTAGTACAATTTAATAAAGTTTGTATTTTATTAGGTATTGATTTTATTTATCCAATTAA | 21541 |
| YJM1388 | 21548 | ---------------------------------------------------------------------------------------------------- | 21547 |
| YJM789 | 23409 | TAATTAATGCAGTTAATGGTAATATTAGAAATACTAAAAGATTAGTACAATTTAATAAAGTTTGTATTTTATTAGGTATTGATTTTATTTATCCAATTAA | 23508 |
| YJM1273 | 16960 | ---------------------------------------------------------------------------------------------------- | 16959 |
| NCYC3585 | 16655 | TAATTAATGCAGTTAATGGTAATATTAGAAATACTAAAAGATTAGTACAATTTAATAAAGTTTGTATTTTATTAGGTATTGATTTTATTTATCCAATTAA | 16754 |
| YJM1401 | 17143 | TAATTAATGCAGTTAATGGTAATATTAGAAATACTAAAAGATTAGTACAATTTAATAAAGTTTGTATTTTATTAGGTATTGATTTTATTTATCCAATTAA | 17242 |
| NCYC3594 | 17863 | ---------------------------------------------------------------------------------------------------- | 17862 |
| YJM1078 | 20326 | ---------------------------------------------------------------------------------------------------- | 20325 |
| YJM1439 | 19828 | TAATTAATGCAGTTAATGGTAATATTAGAAATACTAAAAGATTAGTACAATTTAATAAAGTTTGTATTTTATTAGGTATTGATTTTATTTATCCAATTAA | 19927 |
| consensus | 28001 | ---------------------------------------------------------------------------------------------------- | 28100 |
|
| S288C | 21542 | ATTAACTAAAGATAATAGTTGATTTGTTGGATTTTTTGATGCTGATGGTACAATTAATTATTCATTTAAAAATAATCATCCTCAATTAACAATTTCTGTA | 21641 |
| YJM1388 | 21548 | ---------------------------------------------------------------------------------------------------- | 21547 |
| YJM789 | 23509 | ATTAACTAAAGATAATAGTTGATTTGTTGGATTTTTTGATGCTGATGGTACAATTAATTATTCATTTAAAAATAATCATCCTCAATTAACAATTTCTGTA | 23608 |
| YJM1273 | 16960 | ---------------------------------------------------------------------------------------------------- | 16959 |
| NCYC3585 | 16755 | ATTAACTAAAGATAATAGTTGATTTGTTGGATTTTTTGATGCTGATGGTACAATTAATTATTCATTTAAAAATAATCATCCTCAATTAACAATTTCTGTA | 16854 |
| YJM1401 | 17243 | ATTAACTAAAGATAATAGTTGATTTGTTGGATTTTTTGATGCTGATGGTACAATTAATTATTCATTTAAAAATAATCATCCTCAATTAACAATTTCTGTA | 17342 |
| NCYC3594 | 17863 | ---------------------------------------------------------------------------------------------------- | 17862 |
| YJM1078 | 20326 | ---------------------------------------------------------------------------------------------------- | 20325 |
| YJM1439 | 19928 | ATTAACTAAAGATAATAGTTGATTTGTTGGATTTTTTGATGCTGATGGTACAATTAATTATTCATTTAAAAATAATCATCCTCAATTAACAATTTCTGTA | 20027 |
| consensus | 28101 | ---------------------------------------------------------------------------------------------------- | 28200 |
|
| S288C | 21642 | ACTAATAAATATTTACAAGATGTACAAGAATATAAAAATATTTTAGGTGGTAATATTTATTTTGATAAATCACAAAATGGTTATTATAAATGATCCATTC | 21741 |
| YJM1388 | 21548 | ---------------------------------------------------------------------------------------------------- | 21547 |
| YJM789 | 23609 | ACTAATAAATATTTACAAGATGTACAAGAATATAAAAATATTTTAGGTGGTAATATTTATTTTGATAAATCACAAAATGGTTATTATAAATGATCCATTC | 23708 |
| YJM1273 | 16960 | ---------------------------------------------------------------------------------------------------- | 16959 |
| NCYC3585 | 16855 | ACTAATAAATATTTACAAGATGTACAAGAATATAAAAATATTTTAGGTGGTAATATTTATTTTGATAAATCACAAAATGGTTATTATAAATGATCCATTC | 16954 |
| YJM1401 | 17343 | ACTAATAAATATTTACAAGATGTACAAGAATATAAAAATATTTTAGGTGGTAATATTTATTTTGATAAATCACAAAATGGTTATTATAAATGATCCATTC | 17442 |
| NCYC3594 | 17863 | ---------------------------------------------------------------------------------------------------- | 17862 |
| YJM1078 | 20326 | ---------------------------------------------------------------------------------------------------- | 20325 |
| YJM1439 | 20028 | ACTAATAAATATTTACAAGATGTACAAGAATATAAAAATATTTTAGGTGGTAATATTTATTTTGATAAATCACAAAATGGTTATTATAAATGATCCATTC | 20127 |
| consensus | 28201 | ---------------------------------------------------------------------------------------------------- | 28300 |
|
| S288C | 21742 | AATCAAAAGATATAGTATTAAATTTTATTAATGATTATATTAAAATAAATCCATCAAGAACACTAAAAATAAATAAATTATATTTAAGTAAAGAATTTTA | 21841 |
| YJM1388 | 21548 | ---------------------------------------------------------------------------------------------------- | 21547 |
| YJM789 | 23709 | AATCAAAAGATATAGTATTAAATTTTATTAATGATTATATTAAAATAAATCCATCAAGAACACTAAAAATAAATAAATTATATTTAAGTAAAGAATTTTA | 23808 |
| YJM1273 | 16960 | ---------------------------------------------------------------------------------------------------- | 16959 |
| NCYC3585 | 16955 | AATCAAAAGATATAGTATTAAATTTTATTAATGATTATATTAAAATAAATCCATCAAGAACACTAAAAATAAATAAATTATATTTAAGTAAAGAATTTTA | 17054 |
| YJM1401 | 17443 | AATCAAAAGATATAGTATTAAATTTTATTAATGATTATATTAAAATAAATCCATCAAGAACACTAAAAATAAATAAATTATATTTAAGTAAAGAATTTTA | 17542 |
| NCYC3594 | 17863 | ---------------------------------------------------------------------------------------------------- | 17862 |
| YJM1078 | 20326 | ---------------------------------------------------------------------------------------------------- | 20325 |
| YJM1439 | 20128 | AATCAAAAGATATAGTATTAAATTTTATTAATGATTATATTAAAATAAATCCATCAAGAACACTAAAAATAAATAAATTATATTTAAGTAAAGAATTTTA | 20227 |
| consensus | 28301 | ---------------------------------------------------------------------------------------------------- | 28400 |
|
| S288C | 21842 | TAATTTAAAAGAATTAAAAGCTTATAATAAATCTTCTGATTCAATACAATATAAAGCATGATTAAATTTTGAAAATAAATGAAAAAATAAATAAATTATT | 21941 |
| YJM1388 | 21548 | ---------------------------------------------------------------------------------------------------- | 21547 |
| YJM789 | 23809 | TAATTTAAAAGAATTAAAAGCTTATAATAAATCTTCTGATTCAATACAATATAAAGCATGATTAAATTTTGAAAATAAATGAAAAAATAAATAAATTATT | 23908 |
| YJM1273 | 16960 | ---------------------------------------------------------------------------------------------------- | 16959 |
| NCYC3585 | 17055 | TAATTTAAAAGAATTAAAAGCTTATAATAAATCTTCTGATTCAATACAATATAAAGCATGATTAAATTTTGAAAATAAATGAAAAAATAAATAAATTATT | 17154 |
| YJM1401 | 17543 | TAATTTAAAAGAATTAAAAGCTTATAATAAATCTTCTGATTCAATACAATATAAAGCATGATTAAATTTTGAAAATAAATGAAAAAATAAATAAATTATT | 17642 |
| NCYC3594 | 17863 | ---------------------------------------------------------------------------------------------------- | 17862 |
| YJM1078 | 20326 | ---------------------------------------------------------------------------------------------------- | 20325 |
| YJM1439 | 20228 | TAATTTAAAAGAATTAAAAGCTTATAATAAATCTTCTGATTCAATACAATATAAAGCATGATTAAATTTTGAAAATAAATGAAAAAATAAATAAATTATT | 20327 |
| consensus | 28401 | ---------------------------------------------------------------------------------------------------- | 28500 |
|
| S288C | 21942 | TAATAAAGATATAGTCCAAATTATATATATATAATATATATATATATAACAAGCACCCTGAAGTATATATTTTAATTATTCCTGGATTTGGTATTATTTC | 22041 |
| YJM1388 | 21548 | -----------------------------------------------------CATCCAGAAGTATATATTTTAATTATTCCTGGATTTGGTATTATTTC | 21594 |
| YJM789 | 23909 | TAATAAAGATATAGTCCAAATTATATATATATAATATATATATATATAACAAGCACCCTGAAGTATATATTTTAATTATTCCTGGATTTGGTATTATTTC | 24008 |
| YJM1273 | 16960 | -----------------------------------------------------CATCCAGAAGTATATATTTTAATTATCCCTGGATTTGGTATTATTTC | 17006 |
| NCYC3585 | 17155 | TAATAAAGATATAGTCCAAATTATATATATATAATATATATATATATAACAAGCACCCTGAAGTATATATTTTAATTATTCCTGGATTTGGTATTATTTC | 17254 |
| YJM1401 | 17643 | TAATAAAGATATAGTCCAAATTATATATATATAATATATATATATATAACAAGCACCCTGAAGTATATATTTTAATTATTCCTGGATTTGGTATTATTTC | 17742 |
| NCYC3594 | 17863 | -----------------------------------------------------CATCCAGAAGTATATATTTTAATTATTCCTGGATTTGGTATTATTTC | 17909 |
| YJM1078 | 20326 | -----------------------------------------------------CATCCAGAAGTATATATTTTAATTATTCCTGGATTTGGTATTATTTC | 20372 |
| YJM1439 | 20328 | TAATAAAGATATAGTCCAAATTATATATATATAATATATATATATATAACAAGCACCCTGAAGTATATATTTTAATTATTCCTGGATTTGGTATTATTTC | 20427 |
| consensus | 28501 | -----------------------------------------------------\*\*.\*\*.\*\*\*\*\*\*\*\*\*\*\*\*\*\*\*\*\*\*\*\*.\*\*\*\*\*\*\*\*\*\*\*\*\*\*\*\*\*\*\*\* | 28600 |
|
| S288C | 22042 | ACATGTAGTATCAACATATTCTAAAAAACCTGTATTTGGTGAAATTTCAATGGTATATGCTATGGCTTCAATTGGATTATTAGGATTCTTAGTATGATCA | 22141 |
| YJM1388 | 21595 | ACATGTAGTATCAACATATTCTAAAAAACCTGTATTTGGTGAAATTTCAATGGTATATGCTATGGCTTCAATTGGATTATTAGGATTCTTAGTATGATCA | 21694 |
| YJM789 | 24009 | ACATGTAGTATCAACATATTCTAAAAAACCTGTATTTGGTGAAATTTCAATGGTATATGCTATGGCTTCAATTGGATTATTAGGATTCTTAGTATGATCA | 24108 |
| YJM1273 | 17007 | ACATGTAGTATCAACATATTCTAAAAAACCTGTATTTGGTGAAATTTCAATGGTATATGCTATGGCTTCAATTGGATTATTAGGATTCTTAGTATGATCA | 17106 |
| NCYC3585 | 17255 | ACATGTAGTATCAACATATTCTAAAAAACCTGTATTTGGTGAAATTTCAATGGTATATGCTATGGCTTCAATTGGATTATTAGGATTCTTAGTATGATCA | 17354 |
| YJM1401 | 17743 | ACATGTAGTATCAACATATTCTAAAAAACCTGTATTTGGTGAAATTTCAATGGTATATGCTATGGCTTCAATTGGATTATTAGGATTTTTAGTATGATCA | 17842 |
| NCYC3594 | 17910 | ACATGTAGTATCAACATATTCTAAAAAACCTGTATTTGGTGAAATTTCAATGGTATATGCTATGGCTTCAATTGGATTATTAGGATTCTTAGTATGATCA | 18009 |
| YJM1078 | 20373 | ACATGTAGTATCAACATATTCTAAAAAACCTGTATTTGGTGAAATTTCAATGGTATATGCTATGGCTTCAATTGGATTATTAGGATTCTTAGTATGATCA | 20472 |
| YJM1439 | 20428 | ACATGTAGTATCAACATATTCTAAAAAACCTGTATTTGGTGAAATTTCAATGGTATATGCTATGGCTTCAATTGGATTATTAGGATTCTTAGTATGATCA | 20527 |
| consensus | 28601 | \*\*\*\*\*\*\*\*\*\*\*\*\*\*\*\*\*\*\*\*\*\*\*\*\*\*\*\*\*\*\*\*\*\*\*\*\*\*\*\*\*\*\*\*\*\*\*\*\*\*\*\*\*\*\*\*\*\*\*\*\*\*\*\*\*\*\*\*\*\*\*\*\*\*\*\*\*\*\*\*\*\*\*\*\*\*\*.\*\*\*\*\*\*\*\*\*\*\*\* | 28700 |
|
| S288C | 22142 | CATCATATGTATATTGTAGGATTAGATGCAGAT------------------------------------------------------------------- | 22174 |
| YJM1388 | 21695 | CATCATATGTATATTGTAGGATTAGATGCAGAT------------------------------------------------------------------- | 21727 |
| YJM789 | 24109 | CATCATATGTATATTGTAGGATTAGATGCAGATATGAAAGCTCAAGTGTCGTTATATATGGTAACGTATATATTATTATTTAACTGTATGCCGGAAACTT | 24208 |
| YJM1273 | 17107 | CATCATATGTATATTGTAGGATTAGATGCAGAT------------------------------------------------------------------- | 17139 |
| NCYC3585 | 17355 | CATCATATGTATATTGTAGGATTAGATGCAGATATGAAAGCTCAAGTGTCGTTATATATGGTAACGTATATATTATTATTTAACTGTATGCCGGAAACTT | 17454 |
| YJM1401 | 17843 | CATCATATGTATATTGTAGGATTAGATGCAGATATGAAAGCTCAAGTGTCGTTATATATGGTAACGTATATATTATTATTTAACTGTATGCCGGAAACTT | 17942 |
| NCYC3594 | 18010 | CATCATATGTATATTGTAGGATTAGATGCAGAT------------------------------------------------------------------- | 18042 |
| YJM1078 | 20473 | CATCATATGTATATTGTAGGATTAGATGCAGAT------------------------------------------------------------------- | 20505 |
| YJM1439 | 20528 | CATCATATGTATATTGTAGGATTAGATGCAGAT------------------------------------------------------------------- | 20560 |
| consensus | 28701 | \*\*\*\*\*\*\*\*\*\*\*\*\*\*\*\*\*\*\*\*\*\*\*\*\*\*\*\*\*\*\*\*\*------------------------------------------------------------------- | 28800 |
|
| S288C | 22175 | ---------------------------------------------------------------------------------------------------- | 22174 |
| YJM1388 | 21728 | ---------------------------------------------------------------------------------------------------- | 21727 |
| YJM789 | 24209 | TATACTATTTTAATAATAATGATAATATTATTAAAATAACTAAAATAATAGTCCGCTTATTATTAATATATTTGGAAAAATCTATTATTTTATTTAGATA | 24308 |
| YJM1273 | 17140 | ---------------------------------------------------------------------------------------------------- | 17139 |
| NCYC3585 | 17455 | TATACTATTTTAATAATAATGATAATATTATTAAAATAACTAAAATAATAGTCCGCTTATTATTAATATATTTGGAAAAATCTATTATTTTATTTAGATA | 17554 |
| YJM1401 | 17943 | TATACTATTTTAATAATAATGATAATATTATTAAAATAACTAAAATAATAGTCCGCTTATTATTAATATATTTGGAAAAATCTATTATTTTATTTAGATA | 18042 |
| NCYC3594 | 18043 | ---------------------------------------------------------------------------------------------------- | 18042 |
| YJM1078 | 20506 | ---------------------------------------------------------------------------------------------------- | 20505 |
| YJM1439 | 20561 | ---------------------------------------------------------------------------------------------------- | 20560 |
| consensus | 28801 | ---------------------------------------------------------------------------------------------------- | 28900 |
|
| S288C | 22175 | ---------------------------------------------------------------------------------------------------- | 22174 |
| YJM1388 | 21728 | ---------------------------------------------------------------------------------------------------- | 21727 |
| YJM789 | 24309 | TTTTATAACTAATAATAAACAATCGGCAAGTAACTTTTATTTTTCTTCTATAAAAATTAATAAAAAAACAATTCTTAAAAATCATTTAAATGATATAAAT | 24408 |
| YJM1273 | 17140 | ---------------------------------------------------------------------------------------------------- | 17139 |
| NCYC3585 | 17555 | TTTTATAACTAATAATAAACAATCGGCAAGTAACTTTTATTTTTCTTCTATAAAAATTAATAAAAAAACAATTCTTAAAAATCATTTAAATGATATAAAT | 17654 |
| YJM1401 | 18043 | TTTTATAACTAATAATAAACAATCGGCAAGTAACTTTTATTTTTCTTCTATAAAAATTAATAAAAAAACAATTCTTAAAAATCATTTAAATGATATAAAT | 18142 |
| NCYC3594 | 18043 | ---------------------------------------------------------------------------------------------------- | 18042 |
| YJM1078 | 20506 | ---------------------------------------------------------------------------------------------------- | 20505 |
| YJM1439 | 20561 | ---------------------------------------------------------------------------------------------------- | 20560 |
| consensus | 28901 | ---------------------------------------------------------------------------------------------------- | 29000 |
|
| S288C | 22175 | ---------------------------------------------------------------------------------------------------- | 22174 |
| YJM1388 | 21728 | ---------------------------------------------------------------------------------------------------- | 21727 |
| YJM789 | 24409 | GAAATAAAAATATCAGAACATAAACCTTTATATAAAAGATTAAAAGATGATGAAATATTAGGTTATTATTTAGCAGGTTTAATTGAAGGAGATGGTCATA | 24508 |
| YJM1273 | 17140 | ---------------------------------------------------------------------------------------------------- | 17139 |
| NCYC3585 | 17655 | GAAATAAAAATATCAGAACATAAACCTTTATATAAAAGATTAAAAGATGATGAAATATTAGGTTATTATTTAGCAGGTTTAATTGAAGGAGATGGTCATA | 17754 |
| YJM1401 | 18143 | GAAATAAAAATATCAGAACATAAACCTTTATATAAAAGATTAAAAGATGATGAAATATTAGGTTATTATTTAGCAGGTTTAATTGAAGGAGATGGTCATA | 18242 |
| NCYC3594 | 18043 | ---------------------------------------------------------------------------------------------------- | 18042 |
| YJM1078 | 20506 | ---------------------------------------------------------------------------------------------------- | 20505 |
| YJM1439 | 20561 | ---------------------------------------------------------------------------------------------------- | 20560 |
| consensus | 29001 | ---------------------------------------------------------------------------------------------------- | 29100 |
|
| S288C | 22175 | ---------------------------------------------------------------------------------------------------- | 22174 |
| YJM1388 | 21728 | ---------------------------------------------------------------------------------------------------- | 21727 |
| YJM789 | 24509 | TCGGCGCTAAATATATTACTATTGCTATTAATTATAAAGATATTAAAAATGCTTATTATTTAAAAAAATTAATTGGTTATGGTAATATTAGAAGATACTC | 24608 |
| YJM1273 | 17140 | ---------------------------------------------------------------------------------------------------- | 17139 |
| NCYC3585 | 17755 | TCGGCGCTAAATATATTACTATTGCTATTAATTATAAAGATATTAAAAATGCTTATTATTTAAAAAAATTAATTGGTTATGGTAATATTAGAAGATACTC | 17854 |
| YJM1401 | 18243 | TCGGCGCTAAATATATTACTATTGCTATTAATTATAAAGATATTAAAAATGCTTATTATTTAAAAAAATTAATTGGTTATGGTAATATTAGAAGATACTC | 18342 |
| NCYC3594 | 18043 | ---------------------------------------------------------------------------------------------------- | 18042 |
| YJM1078 | 20506 | ---------------------------------------------------------------------------------------------------- | 20505 |
| YJM1439 | 20561 | ---------------------------------------------------------------------------------------------------- | 20560 |
| consensus | 29101 | ---------------------------------------------------------------------------------------------------- | 29200 |
|
| S288C | 22175 | ---------------------------------------------------------------------------------------------------- | 22174 |
| YJM1388 | 21728 | ---------------------------------------------------------------------------------------------------- | 21727 |
| YJM789 | 24609 | TAATACTGATAAAGCTGTAAGTTTAAATTTTGATTCTAAAGCAGCTATATTAAGAGTATTTAATTTAATTAATGGTAAATTATTAGGCCCTTATAAGCAT | 24708 |
| YJM1273 | 17140 | ---------------------------------------------------------------------------------------------------- | 17139 |
| NCYC3585 | 17855 | TAATACTGATAAAGCTGTAAGTTTAAATTTTGATTCTAAAGCAGCTATATTAAGAGTATTTAATTTAATTAATGGTAAATTATTAGGCCCTTATAAGCAT | 17954 |
| YJM1401 | 18343 | TAATACTGATAAAGCTGTAAGTTTAAATTTTGATTCTAAAGCAGCTATATTAAGAGTATTTAATTTAATTAATGGTAAATTATTAGGCCCTTATAAGCAT | 18442 |
| NCYC3594 | 18043 | ---------------------------------------------------------------------------------------------------- | 18042 |
| YJM1078 | 20506 | ---------------------------------------------------------------------------------------------------- | 20505 |
| YJM1439 | 20561 | ---------------------------------------------------------------------------------------------------- | 20560 |
| consensus | 29201 | ---------------------------------------------------------------------------------------------------- | 29300 |
|
| S288C | 22175 | ---------------------------------------------------------------------------------------------------- | 22174 |
| YJM1388 | 21728 | ---------------------------------------------------------------------------------------------------- | 21727 |
| YJM789 | 24709 | AAACAATTAATTGATAATAAATACGATATTAAATTTAATACTTTAATTAAACCTATCGCTAATTTTAATTTATGAGATAACCCCTGATTAACTGGTTTTA | 24808 |
| YJM1273 | 17140 | ---------------------------------------------------------------------------------------------------- | 17139 |
| NCYC3585 | 17955 | AAACAATTAATTGATAATAAATACGATATTAAATTTAATACTTTAATTAAACCTATCGCTAATTTTAATTTATGAGATAACCCCTGATTAACTGGTTTTA | 18054 |
| YJM1401 | 18443 | AAACAATTAATTGATAATAAATACGATATTAAATTTAATACTTTAATTAAACCTATCGCTAATTTTAATTTATGAGATAACCCCTGATTAACTGGTTTTA | 18542 |
| NCYC3594 | 18043 | ---------------------------------------------------------------------------------------------------- | 18042 |
| YJM1078 | 20506 | ---------------------------------------------------------------------------------------------------- | 20505 |
| YJM1439 | 20561 | ---------------------------------------------------------------------------------------------------- | 20560 |
| consensus | 29301 | ---------------------------------------------------------------------------------------------------- | 29400 |
|
| S288C | 22175 | ---------------------------------------------------------------------------------------------------- | 22174 |
| YJM1388 | 21728 | ---------------------------------------------------------------------------------------------------- | 21727 |
| YJM789 | 24809 | CTGACGCTGATGGTAGTTTTGGAGTATATATTTATAAATCAAAAACTATAAAAATAGGATATAATGTAAAAATTATATTTAGAATTAAACAAAGATATGT | 24908 |
| YJM1273 | 17140 | ---------------------------------------------------------------------------------------------------- | 17139 |
| NCYC3585 | 18055 | CTGACGCTGATGGTAGTTTTGGAGTATATATTTATAAATCAAAAACTATAAAAATAGGATATAATGTAAAAATTATATTTAGAATTAAACAAAGACATGT | 18154 |
| YJM1401 | 18543 | CTGACGCTGATGGTAGTTTTGGAGTATATATTTATAAATCAAAAACTATAAAAATAGGATATAATGTAAAAATTATATTTAGAATTAAACAAAGACATGT | 18642 |
| NCYC3594 | 18043 | ---------------------------------------------------------------------------------------------------- | 18042 |
| YJM1078 | 20506 | ---------------------------------------------------------------------------------------------------- | 20505 |
| YJM1439 | 20561 | ---------------------------------------------------------------------------------------------------- | 20560 |
| consensus | 29401 | -----------------------------------------------------------------------------------------------.---- | 29500 |
|
| S288C | 22175 | ---------------------------------------------------------------------------------------------------- | 22174 |
| YJM1388 | 21728 | ---------------------------------------------------------------------------------------------------- | 21727 |
| YJM789 | 24909 | TGATTTATTAAAACATATTCAAAAAGCTTTAGATGGTAGTATTTCTTTATTTAAAATAAAATCAGTAGTTGGAGGTTATAATTATTCAGCTCTTAATTTT | 25008 |
| YJM1273 | 17140 | ---------------------------------------------------------------------------------------------------- | 17139 |
| NCYC3585 | 18155 | TGATTTATTAAAACATATTCAAAAAGCTTTAGATGGTAGTATTTCTTTATTTAAAATAAAATCAGTAGTTGGAGGTTATAATTATTCAGCTCTTAATTTT | 18254 |
| YJM1401 | 18643 | TGATTTATTAAAACATATTCAAAAAGCTTTAGATGGTAGTATTTCTTTATTTAAAATAAAATCAGTAGTTGGAGGTTATAATTATTCAGCTCTTAATTTT | 18742 |
| NCYC3594 | 18043 | ---------------------------------------------------------------------------------------------------- | 18042 |
| YJM1078 | 20506 | ---------------------------------------------------------------------------------------------------- | 20505 |
| YJM1439 | 20561 | ---------------------------------------------------------------------------------------------------- | 20560 |
| consensus | 29501 | ---------------------------------------------------------------------------------------------------- | 29600 |
|
| S288C | 22175 | ---------------------------------------------------------------------------------------------------- | 22174 |
| YJM1388 | 21728 | ---------------------------------------------------------------------------------------------------- | 21727 |
| YJM789 | 25009 | AATTCTATTAAAAATTGAATTAAATATTTTAGTTTATATCCACCATTGCATAATAGTAAATATATTCTTTTTATTAAATGATATAAAGTTTGTTATTTAT | 25108 |
| YJM1273 | 17140 | ---------------------------------------------------------------------------------------------------- | 17139 |
| NCYC3585 | 18255 | AATTCTATTAAAAATTGAATTAAATATTTTAGTTTATATCCACCATTGCATAATAGTAAATATATTCTTTTTATTAAATGATATAAAGTTTGTTATTTAT | 18354 |
[truncated: 1,087,626 more chars]
