# Supplementary figures and images for: Population structure of mitochondrial genomes in Saccharomyces cerevisiae
Source: BMC Genomics. 2015 Jun 11;16(1):451. doi: 10.1186/s12864-015-1664-4 (PMC4464245; doi:10.1186/s12864-015-1664-4)

**K=15**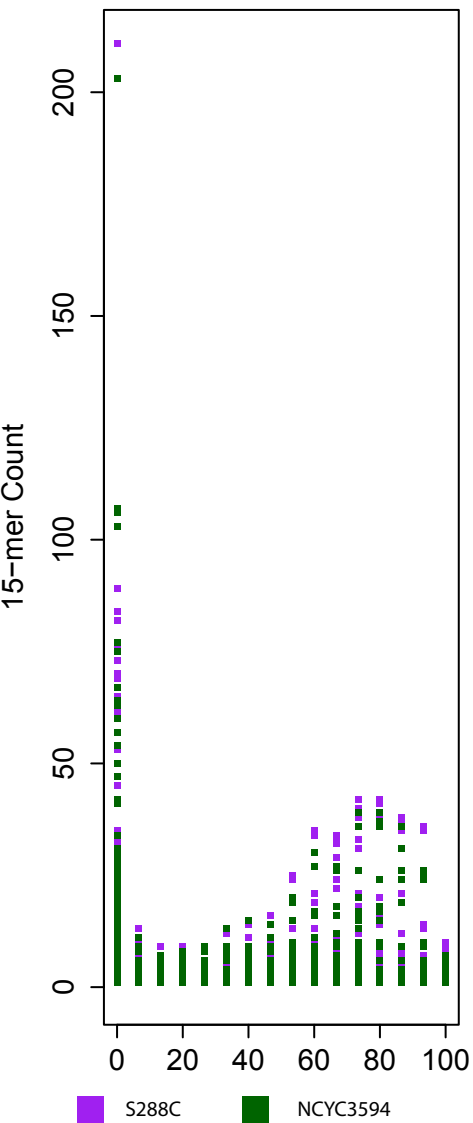**K=30**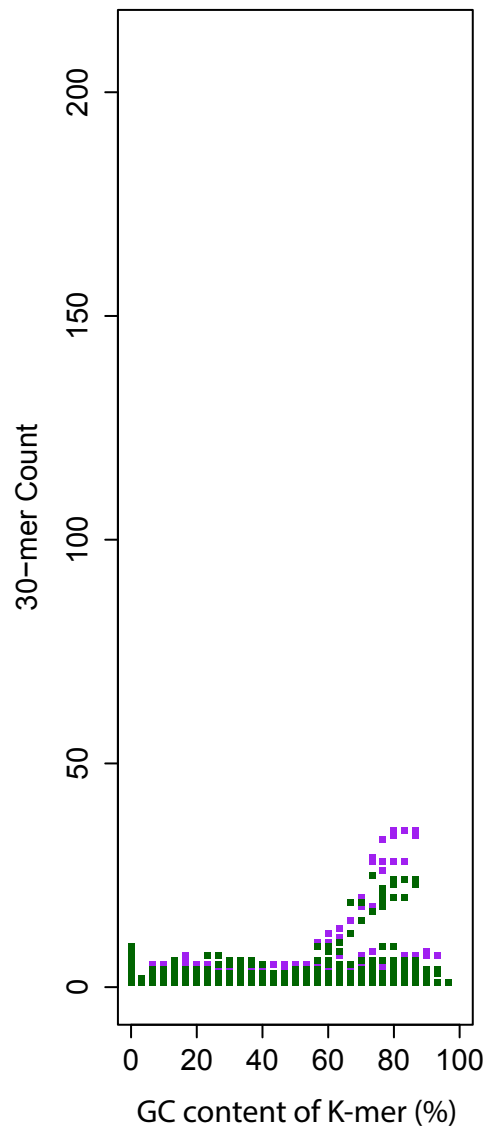**K=50**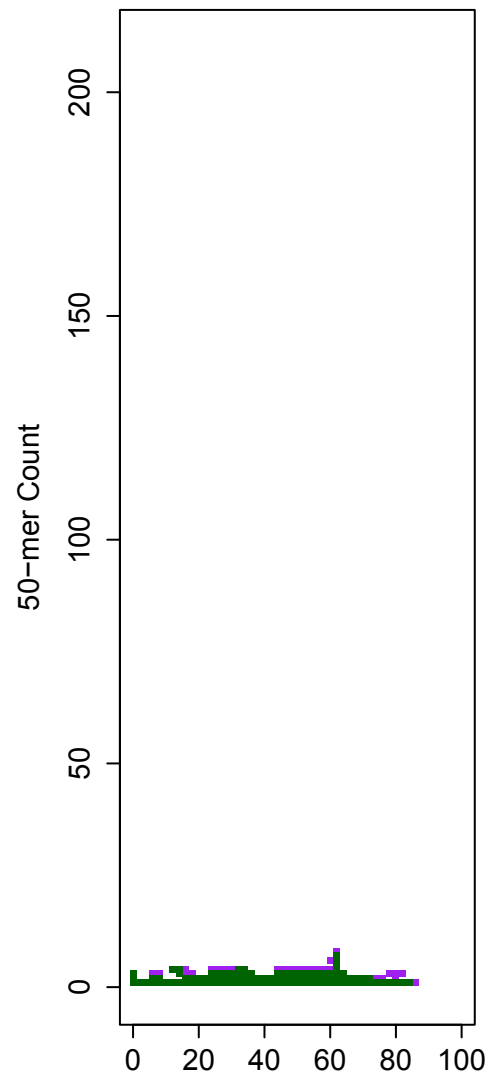

Supplement: Additional file 6: File S2. — Repetitive elements. A k-mer analysis of repetitive sequences in strains S2883 and NCYC3594. Each square represents a single, potentially overlapping, k-mer. [file 12864_2015_1664_MOESM6_ESM.pdf]

- mosaic-A
- mosaic-B
- wine/European
- North American
- sake
- Maylasian
- West African
- Unknown

— 0.1

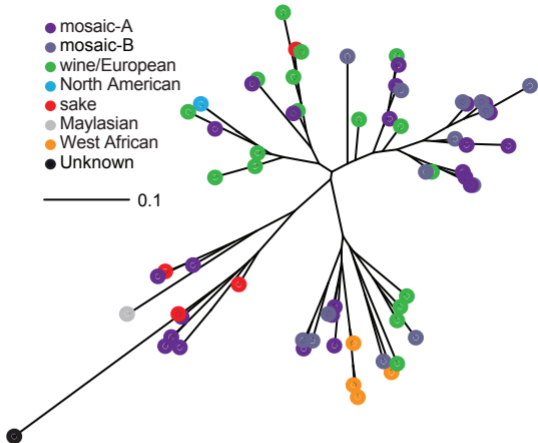

Supplement: Additional file 7: Figure S3. — Intron phylogenetics. A phylogenetic tree built from intron profiling, treated as binary character states. Any strain with a questionable intron profile was excluded. [file 12864_2015_1664_MOESM7_ESM.pdf]
